# Detecting Clinically Significant Prostate Cancer in PI-RADS 3 Lesions Using T2w-Derived Radiomics Feature Maps in 3T Prostate MRI

**DOI:** 10.3390/curroncol31110503

**Published:** 2024-11-01

**Authors:** Laura J. Jensen, Damon Kim, Thomas Elgeti, Ingo G. Steffen, Lars-Arne Schaafs, Matthias Haas, Lukas J. Kurz, Bernd Hamm, Sebastian N. Nagel

**Affiliations:** 1 Charité–Universitätsmedizin Berlin, Corporate Member of Freie Universität Berlin, Humboldt-Universität zu Berlin, and Berlin Institute of Health, Department of Radiology, Hindenburgdamm 30, 12203 Berlin, Germany; 2 Charité–Universitätsmedizin Berlin, Corporate Member of Freie Universität Berlin, Humboldt-Universität zu Berlin, and Berlin Institute of Health, Department of Urology, Hindenburgdamm 30, 12203 Berlin, Germany; 3 Bielefeld University, Medical School and University Medical Center East Westphalia-Lippe, Protestant Hospital of the Bethel Foundation, Academic Department of Diagnostic and Interventional Radiology and Paediatric Radiology, Burgsteig 13, 33617 Bielefeld, Germany

**Keywords:** prostate, magnetic resonance imaging, radiomics, prostatic neoplasms, prostatitis

## Abstract

Prostate Imaging Reporting and Data System version 2.1 (PI-RADS) category 3 lesions are a challenge in the clinical workflow. A better detection of the infrequently occurring clinically significant prostate cancer (csPCa) in PI-RADS 3 lesions is an important objective. The purpose of this study was to evaluate if feature maps calculated from T2-weighted (T2w) 3 Tesla (3T) MRI can help detect csPCa in PI-RADS category 3 lesions. In-house biparametric 3T prostate MRI examinations acquired between January 2019 and June 2023 because of elevated prostate-specific antigen (PSA) levels were retrospectively screened. Inclusion criteria were a PI-RADS 3 lesion and available results of an ultrasound-guided targeted and systematic biopsy. Exclusion criteria were a simultaneous PI-RADS category 4 or 5 lesion and hip replacement. Target lesions with the International Society of Urological Pathology (ISUP) grade group 1 were rated clinically insignificant PCa (ciPCa) and ≥2 csPCa. This resulted in 52 patients being included in the final analysis, of whom 11 (21.1%), 8 (15.4%), and 33 (63.5%) patients had csPCa, ciPCa, and no PCa, respectively, with the latter two groups being combined as non-csPCa. Eight of the csPCas were located in the peripheral zone (PZ) and three in the transition zone (TZ). In the non-csPCa group, 29 were located in the PZ and 12 in the TZ. Target lesions were marked with volumes of interest (VOIs) on axial T2w images. Axial T2w images were then converted to 93 feature maps. VOIs were copied into the maps, and feature quantity was retrieved directly. Features were tested for significant differences with the Mann–Whitney U-test. Univariate models for single feature performance and bivariate models implementing PSA density (PSAD) were calculated. Ten map-derived features differed significantly between the csPCa and non-csPCa groups (AUCs: 0.70–0.84). The diagnostic performance for TZ lesions (AUC: 0.83–1.00) was superior to PZ lesions (AUC: 0.74–0.85). In the bivariate models, performance in the PZ improved with AUCs >0.90 throughout. Parametric feature maps alone and as bivariate models with PSAD can (?) noninvasively identify csPCa in PI-RADS 3 lesions and could serve as a quantitative tool reducing ambiguity in PI-RADS 3 lesions.

## 1. Introduction

Prostate cancer (PCa) is among the most prevalent malignancies and the second most frequent cancer worldwide, increasing with the aging population [1,2,3]. While a considerable number of detected PCas are clinically insignificant (ciPCas) and usually remain without clinical consequences for a man’s lifetime, identifying the small percentage of aggressive, clinically significant PCas (csPCas) is crucial [4,5,6].

Besides clinical parameters such as serum prostate-specific antigen (PSA) levels, multiparametric (mp) MRI, including T2-weighted (T2w) images; diffusion-weighted imaging (DWI); and dynamic contrast-enhanced (DCE) sequences have been established as a standard diagnostic workup in men at risk for PCa [7,8,9]. mpMRI has proven beneficial in tumor detection and targeting of biopsies [2,10,11]. Striving to standardize and structure radiological image acquisition and reporting in prostate MRI, an expert panel launched the Prostate Imaging Reporting and Data System (PI-RADS) and updated it to version 2.1 in 2019 [12,13]. The score has proven useful and is crucial in clinical care [14,15,16,17,18]. While the PI-RADS scores 1 and 2 indicate very low and low likelihoods and 4 and 5 are high and very high likelihoods and warrant prostate biopsy, the PI-RADS score 3 is the most ambiguous. PI-RADS score 3 indicates intermediate risk with an equivocal suspicion of csPCa [12,19]. The optimal management and need for biopsy of a PI-RADS 3 lesion is contentious [4]. An improved imaging-based assessment of PI-RADS 3 lesions could prevent unnecessary biopsies and help select patients requiring prompt diagnostic workup.

The “radiomics” approach endeavors to noninvasively gain information on tissue properties from radiological images. Image features beyond visual perception are retrieved, resulting in high-dimensional data that are further processed (e.g., by modeling and deep learning) to establish imaging biomarkers [20,21,22,23]. Despite the abundant empirical findings on radiomics, application in clinical care remains scarce, with studies being criticized for lacking standardization, reproducibility, and transparency [23,24,25]. As one option to increase the transparency and reproducibility of radiomics, parametric feature maps were introduced [26,27,28]. With an automated software script, image stacks are dissembled into a grid of small volumes of interest (VOIs) and converted to parametric maps, one for each feature, maintaining spatial information and reflecting feature quantity in brightness [26]. The resulting maps allow the direct visualization and numerical retrieval of feature quantity [26]. This tool has been applied in MR images of the liver and lung but not to prostate MRI [28,29].

Since PI-RADS 3 lesions cannot be elucidated using conventional MR sequences, this pioneering study aimed to investigate if T2w-derived radiomic feature maps could help to noninvasively identify csPCa.

## 2. Materials and Methods

### 2.1. Patient Population and Target Lesions

**Figure 1:**
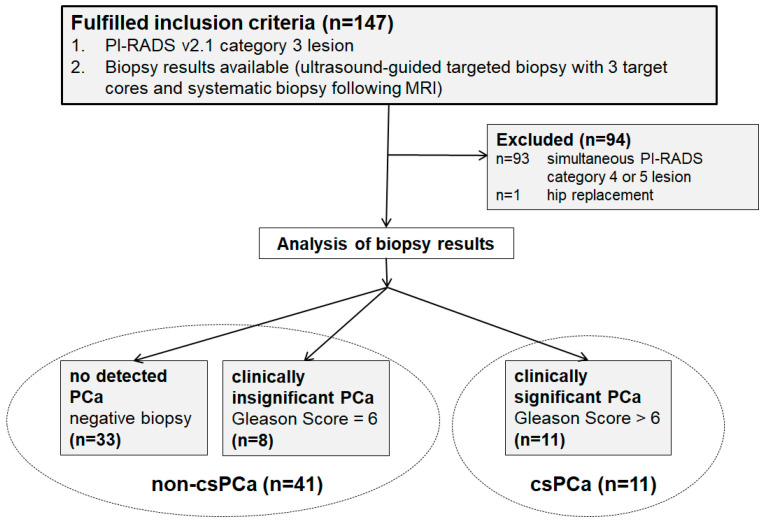
Flow diagram of patients. PCa: Prostate cancer.

This single-center study was approved by the institutional review board (EA1/104/19). In-house prostate exams acquired on one 3 Tesla MRI scanner (Magnetom Skyra, Siemens Healthineers, Erlangen, Germany) from January 2019 to June 2023 were screened retrospectively. Our institution specializes in urogenital imaging (tertiary/quaternary care). Prostate exams are reported by experienced and certified radiologists in prostate imaging within the dual control principle. Patients were enrolled consecutively. Inclusion criteria were (1) a PI-RADS 3 lesion and (2) available results of an ultrasound-guided targeted biopsy with 3 target cores and systematic biopsy following MRI. Exclusion criteria were (1) a simultaneous PI-RADS category 4 or 5 lesion, and (2) hip replacement because of image deterioration. If there was more than one PI-RADS 3 lesion in one patient, the most suspicious lesion (based on the T2w images in the TZ and the DWI in the PZ) was chosen as the target lesion. Histopathological results of the target lesion (3 target cores) served as the reference standard. According to the biopsy reports, the target lesions were grouped as csPCa (International Society of Urological Pathology (ISUP) grade group ≥ 2) and non-csPCa, combining ciPCa (ISUP grade group 1) and cases without the detection of PCa (negative biopsy). Since the definition of clinically significant PCa is not standardized, a Gleason Score > 6 was chosen as the cutoff for csPCa [19,30]. Figure 1 shows the patient cohort. Table 1 summarizes patient characteristics.

### 2.2. MRI Examination

All patients were examined on one 3 Tesla MRI scanner (Magnetom Skyra, Siemens Healthineers, Erlangen, Germany) in clinical care including an axial and coronal T2-weighted turbo spin echo (TSE) and an axial diffusion-weighted sequence (b-values of 0, 50, 400, and 1000) of the prostate, and furthermore an axial T1w gradient echo sequence and a sagittal T2w TSE sequence of the pelvis. The exam protocol followed PI-RADS standards and was identical for all patients [19]. Contrast-enhanced sequences were not included in the imaging protocol. PI-RADS scoring was accordingly based on biparametric MRI. Patients were asked to evacuate the urinary bladder and rectum before the MRI scan. If there were no contraindications or rejection by the patient, 40 mg Buscopan (intramuscular) was applied as an antispasmodic agent immediately before the scan. An endorectal coil was not used. Supplementary File S1 summarizes the technical details of the axial T2w TSE sequence.

### 2.3. Lesion Segmentation

The T2w axial images were retrieved from the picture archiving and communication system in the DICOM data format. Target lesions were delineated with volumes of interest (VOIs) using the open-source software 3D slicer (3D Slicer, Version 5.2.2, www.slicer.org). Segmentation was performed twice independently to ensure the correct marking of the target lesion and to assess the interrater agreement. Both readers are board-certified radiologists, one with over six years of experience (L.J.J.), and the other with over 11 years of experience (S.N.N.). The segmentation of reader 1 was considered for a further analysis. The readers were provided with the written report indicating the location of the PI-RADS 3 lesion and the correlating diffusion-weighted sequences. No further clinical data (e.g., the histopathological diagnosis) were apparent to the readers.

### 2.4. Calculation of Parametric Feature Maps and Feature Extraction

**Figure 2:**
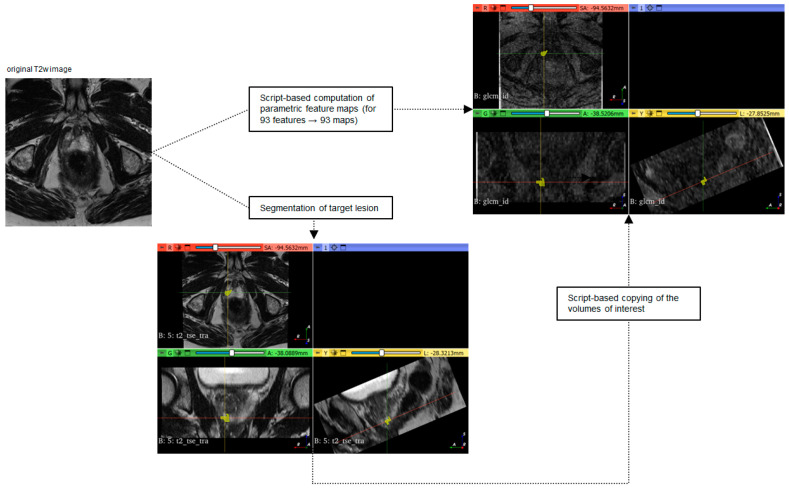
Workflow from the original images to the parametric feature maps. The original T2w images are computed into parametric maps using a script-based pipeline. A separate map is calculated for each of the 93 features, resulting in 93 feature maps per patient. As a parallel step, the target lesions are segmented manually in the original T2w images with volumes of interest (VOIs) using the open-source software 3D sclicer (www.slicer.org). In the software application, the axial (red window), coronal (green window), and sagittal (yellow window) reformation including the VOI is shown. In the next automated step, the script is used to copy the VOI into all the maps at the exact location as in the original image. The quantity of the respective feature can be determined by extracting the mean from the VOI.

**Figure 3:**
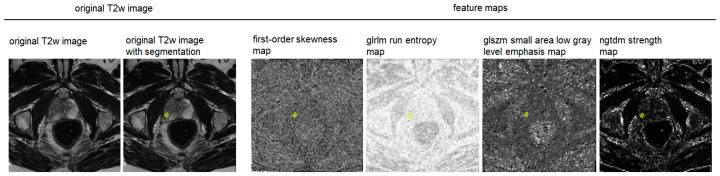
Examples of parametric feature maps. On the left, the original axial T2-weighted (T2w) image and the segmentation, i.e., the volume of interest (VOI, yellow) of the target lesion are shown, and on the right, exemplary parametric feature maps are shown. The feature maps carry the same spatial information as the original image and reflect the feature quantity in brightness. Quantitative information on the feature can be directly drawn from the map once it is computed by extracting the mean. The VOI is copied into the feature maps in an automated step of the software script.

Parametric feature maps were computed using a pretested software script [26,27]. A total of 93 parametric feature maps per patient were calculated based on the axial T2w image stack. After the initial analysis of the spatial resolution of the feature maps, the voxel size was adjusted to 2 mm (i.e., the software tool dissembled the original image into small VOIs of 2 × 2 × 3 mm with 3 mm representing the z-axis of the voxel matching the slice thickness of the original MR images). Supplementary File S2 contains the applied software script. Except for the shape feature group, maps of all features available by PyRadiomics were computed: 18 first-order features (energy, total energy, entropy, kurtosis, maximum, minimum, mean, median, interquartile range (IQR), skewness, range, mean absolute deviation (MAD), robust mean absolute deviation (RMAD), root mean squared (RMS), variance, uniformity, 10th percentile, and 90th percentile) and second -order features (24 gray-level co-occurrence matrix (GLCM) features, 14 gray-level dependence matrix (GLDM) features, 16 gray-level run-length matrix (GLRLM) features, 16 gray-level size zone matrix (GLSZM) features, and 5 neighboring gray tone difference matrix (NGTDM) features) [31]. VOIs were copied from the original images to the feature maps to maintain their location and the mean value at their position was extracted from each map, reflecting the respective feature quantity. Figure 2 illustrates the workflow from the original image to the feature extraction from the parametric maps. Figure 3 shows examples of parametric feature maps.

### 2.5. Statistical Analysis

**Figure 4:**
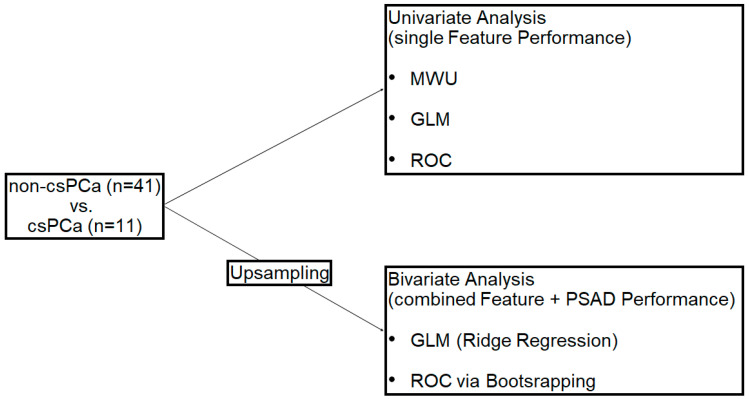
The steps in the data analysis are shown. Differences between the patients with clinically significant prostate cancer (csPCa) and patients without PCa and with clinically insignificant PCa, grouped as non-csPCa, were investigated. In the univariate analysis, significant differences between the two groups were analyzed using the Mann–Whitney U (MWU) test. Generalized linear models (GLMs) of the single map-derived features were built, and the performance was assessed using a receiver operating characteristic (ROC) curve analysis. In the bivariate analysis, data were upsampled, and GLMs combining the single map-derived features with serum prostate-specific antigen density (PSAD) were built applying ridge regression. Performance was assessed with ROC via bootstrapping.

The statistical analysis was performed using R (version 4.2.1, R Foundation for Statistical Computing) [32]. Differences between the csPCa and non-csPCa groups were tested for significant differences using the Mann–Whitney U (MWU) test of the R base package. For the univariate and bivariate analyses of imaging features and clinical parameters, logistic regression was performed. Univariate generalized linear models (GLMs) were calculated without upsampling using the glmnet package version 4.1-8 (refs. [33,34]). For the bivariate analysis with serum prostate-specific antigen density (PSAD), class imbalance was addressed using upsampling to avoid bias from unequal datasets using the caret package version 6.0-94 (ref. [35]). Bootstrapping (*n* = 1000) was applied to estimate odds ratios, confidence intervals, and *p*-values. Model performance was evaluated by calculating the AUC (Area Under the Curve) and compared using the DeLong test using the pROC package version 1.18.5 (ref. [36]). In the bivariate models, PSAD was considered (1) as a continuous variable, (2) with a cutoff of 0.15 (ng/mL)/cm^3^, and (3) with a cutoff of 0.20 (ng/mL)/cm^3^ in the GLMs. Figure 4 summarizes the steps in the data analysis. Data were considered for the peripheral and transition zone lesions and separated by zone (peripheral/transition). Diagnostic performance of the individual feature maps and models was determined by a receiver operating characteristic (ROC) curve analysis using the pROC package [36]. The AUCs were rated according to Koo and Li (70–80% is acceptable, 80–90% is excellent, and 90–100% is outstanding) [37]. Interrater agreement was assessed with intraclass correlation coefficients (ICCs) (ICC3 according to the Shrout and Fleiss Convention) using the psych package for R (Version 2.2.5) [38,39]. Interrater reliabilities were classified as poor to excellent (ICC < 0.5 is poor, 0.5 to 0.75 is moderate, 0.75 to 0.9 is good, and >0.9 is excellent) [37]. A *p*-value < 0.05 was generally considered to indicate statistical significance.

## 3. Results

### 3.1. Univariate Analysis

**Figure 5:**
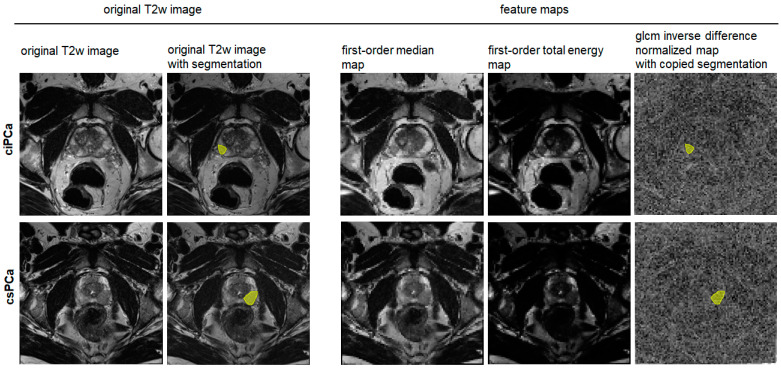
Exemplary feature maps of best-performing features in the peripheral zone. In the upper row, images of a patient with clinically insignificant prostate cancer (ciPCa, Gleason Score 3 + 3, prostate-specific antigen (PSA) density: 0.17 (ng/mL)/cm^3^) and in the lower row images of a patient with clinically significant prostate cancer (csPCa, Gleason Score 3 + 4, PSA density: 0.17 (ng/mL)/cm^3^) are shown. On the left, the original T2-weighted axial image and the segmented target lesion marked with a volume of interest (VOI, yellow) are displayed. On the right, the parametric feature maps of the first-order median, first-order total energy, and glcm inverse difference normalized are shown. The map-derived features first-order median and first-order total energy revealed excellent diagnostic performance in differentiating csPCa from non-csPCa (no PCa and clinically insignificant PCa). VOIs were copied based on software in the maps, as shown for the glcm inverse difference normalized map, to extract feature quantity directly.

**Figure 6:**
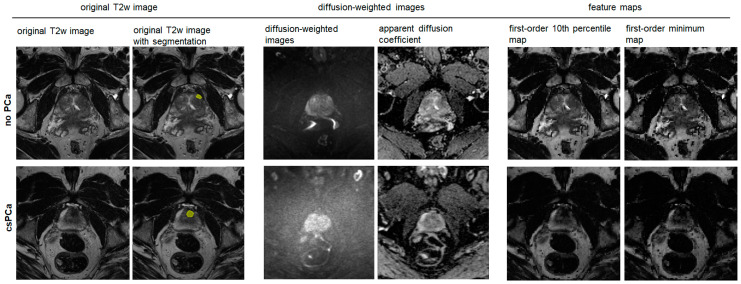
Feature maps with the best diagnostic performance in the transition zone. Both rows display images of a PI-RADS 3 lesion in the left anterior transition zone. Next to the original T2-weighted (T2w) images, the segmentation of the target lesion, i.e., the volume of interest (VOI, yellow) is shown. The patient in the upper row had a prostate-specific antigen (PSA) density of 0.09 (ng/mL)/cm^3^, and the biopsy revealed no prostate cancer (PCa). The lower row shows images of a patient with a PSA density of 0.16 (ng/mL)/cm^3^ and biopsy-proven clinically significant prostate cancer (csPCa) with a Gleason Score of 4 + 5. The features derived from the first-order 10th percentile and minimum map differed significantly between the csPCa and non-csPCa groups with excellent to outstanding diagnostic performance.

**Figure 7:**
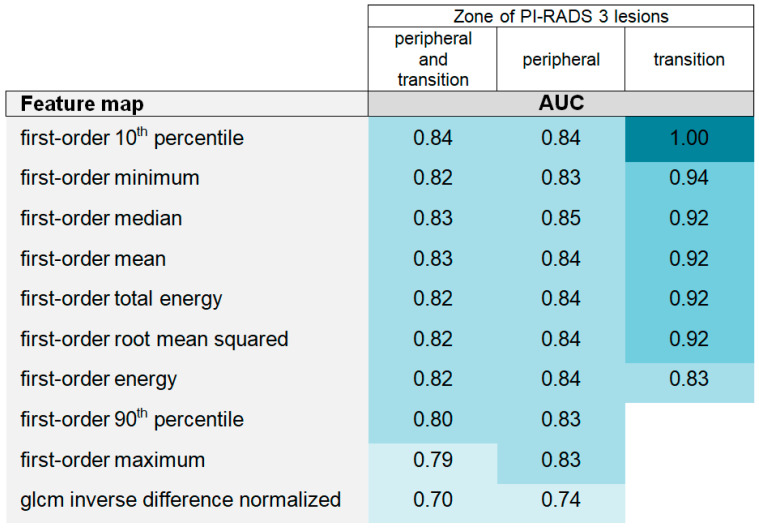
The heatmap of the single-feature performance separated by zone. Map-derived features that were significantly different between the clinically significant prostate cancer (csPCa) and non-csPCa (clinically insignificant prostate cancer and no prostate cancer) groups are listed on the left. Ten features differentiated the two groups across all prostatic zones with areas under the curve (AUCs) of 0.70–0.84. Considering only lesions localized in the peripheral zone, the ten exact features differed significantly but with slightly better diagnostic performance (AUCs: 0.74–0.85). When the analysis was limited to lesions in the transition zone, seven features differed significantly with excellent to outstanding diagnostic performance (AUCs: 0.83–0.10).

Assessing PI-RADS 3 lesions in the peripheral (*n* = 37) and transition (*n* = 15) zones together, 10 out of 93 map-derived features were significantly different between the csPCa and non-csPCa groups in the MWU test. In the ROC analysis of single-feature GLMs, diagnostic performance was excellent for eight map-derived features (AUC: 0.82–0.84) and acceptable for two map-derived features (AUC: 0.70–0.79). For PI-RADS 3 lesions in the peripheral zone, the same ten map-derived features differed significantly, with nine features showing excellent diagnostic performance (AUC: 0.83–0.85) and one with acceptable performance (AUC: 0.74). Feature maps of significantly different features in the peripheral zone are shown in Figure 5. Considering PI-RADS 3 lesions in the transition zone, the MWU test revealed seven significantly different map-derived features: six with outstanding diagnostic performance (AUC: 0.92–1.00) and one with excellent diagnostic performance (AUC: 0.83). Feature maps with the best diagnostic performance in the transition zone are shown in Figure 6. Figure 7 summarizes the results of the MWU test and ROC analysis. In the Supplementary Materials, detailed results of the MWU test (Supplementary File S3 for all PI-RADS 3 lesions, Supplementary File S4 for lesions in the peripheral zone only, and Supplementary File S5 for lesions in the transition zone only) and the ROC analysis including sensitivity, negative predictive value (NPV), specificity, and positive predictive value (PPV) (Supplementary File S6 for all PI-RADS 3 lesions, Supplementary File S7 for lesions in the peripheral zone only, and Supplementary File S8 for lesions in the transition zone only) are provided. Supplementary Files S9–S11 show the corresponding ROC curves for all features.

### 3.2. Bivariate Analysis

**Figure 8:**
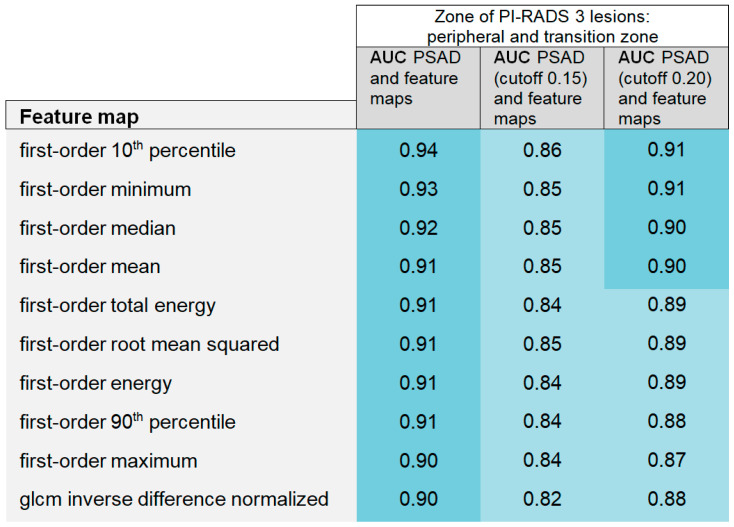
Areas under the curve (AUCs) of the generalized linear models combining the performance of feature maps and prostate-specific antigen density (PSAD) are summarized in a heatmap, considering PI-RADS 3 category 3 lesions in the peripheral and transition zones together. PSAD was implemented in the models as a continuous variable (AUC PSAD and feature maps) and with a cutoff of 0.15 (ng/mL)/cm^3^ and 0.20 (ng/mL)/cm^3^.

**Figure 9:**
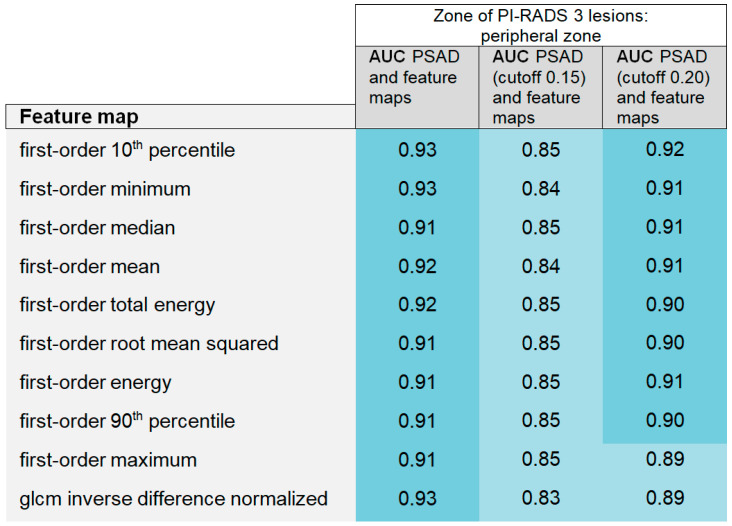
The heatmap shows the areas under the curve (AUCs) of the generalized linear models combining the map-derived features with the prostate-specific antigen density (PSAD), considering PI-RADS category 3 lesions only in the peripheral zone. Models were calculated with PSAD as a continuous variable (AUC PSAD and feature maps) and with a cutoff of 0.15 (ng/mL)/cm^3^ and 0.20 (ng/mL)/cm^3^.

AUCs of GLMs, including the PSAD as a continuous variable and with a cutoff of 0.15 and 0.20 (ng/mL)/cm^3^, were superior to the single-feature performance in the univariate analysis considering peripheral and transition zones together, as shown in Figure 8. Also, when only considering PI-RADS 3 lesions in the peripheral zone, the models implementing PSAD as a continuous variable yielded excellent diagnostic performance (AUC > 0.90) throughout, as shown in Figure 9. Results of the ROC analysis via bootstrapping (odds ratios, confidence intervals, and *p*-values) are provided in Supplementary File S12 for the peripheral and transition zones together and in Supplementary File S13 for the peripheral zone only. Given the small sample size of csPCa in the transition zone, the diagnostic power of the models for the transition zone is limited, and the results are presented in the Supplementary Files (see File S14).

### 3.3. Interrater Agreement

ICCs showed overall excellent interrater reliability for all features (ICCs ≥ 0.96; *p* < 0.001). ICCs per feature are provided in the Supplementary File S15.

## 4. Discussion

The presented study shows that radiomics from T2w MRI parametric feature maps help to noninvasively detect csPCa in PI-RADS 3 lesions with superior diagnostic performance in the transition zone. Performance in the peripheral zone was leveled up by building bivariate models combining feature maps with PSAD. Therefore, the software-based generation of T2w parametric feature maps alone and combined with PSAD in prediction models can be an additional diagnostic aid for PI-RADS 3 lesions. About 17% of prostate MRI reports in the clinical routine are scored with PI-RADS category 3, and only 15–19% of this group hold an ISUP grade group > 1 [40,41]. Because of this, a diagnostic tool based on subvisual quantitative criteria could contribute to a more appropriate and targeted management of PI-RADS category 3 cases. Otherwise, managing PI-RADS category 3 cases is still somewhat controversial, ranging from surveillance to immediate biopsy [42,43]. Given the fact that prostate biopsy is associated with possible complications like prostatitis, urinary tract infections, and sepsis [44], using parametric maps could facilitate the decision making about biopsy in multimorbid patients and, above all, avoid unnecessary biopsies. Parametric maps could also help to guide a follow-up biopsy when the histopathological diagnosis is doubted and enhance the low cancer detection rates in systematic transrectal ultrasound-guided biopsies (27–40%) [45].

When applying the parametric map approach, the entire image stack is converted to a feature map stack reflecting the quantity of the feature in brightness while maintaining the anatomical information of the images. By using an automated software-based pipeline, the original image is dissembled into a grid of voxels of a predefined size. Then, the feature is calculated for each voxel and the feature quantity is stored in the map with the same spatial information as in the original image, reflecting the feature’s quantity in brightness [26,27]. Thus, the feature quantity is directly visualized for the complete image stack, enabling quick assessment from any volume of interest (even beyond organ boundaries) by extracting the mean, similar to the use of quantitative apparent diffusion coefficient (ADC) metrics in prostate imaging [4]. The maps increase the transparency of the features’ behavior by the direct visualization of radiomic features, particularly for those with complex underlying mathematics [28].

Another benefit of parametric maps is the enhanced reproducibility of radiomics against different VOI sizes, which are known to have confounding effects on radiomic features [31,46,47]. Previous studies showed increased reproducibility of radiomics across different VOI sizes when features were derived from parametric maps instead of directly extracted from the original images [27,28]. This is because the feature quantity is calculated for voxels of the same size, which reduces confounding effects caused by different VOI sizes. Since PI-RADS 3 lesions are strongly variable in size, the computation of feature maps seems useful in related radiomics studies [48]. Also, other factors, such as imaging artifacts that could influence feature quantity, can be mitigated by calculating feature maps since the disassembly of the image in the voxel grid can smooth out single outliers as they only appear in single voxels but do not affect the whole-tumor VOI [27,28].

Other studies also attempted detecting csPCa in PI-RADS 3 lesions using texture analysis or radiomics combined with modeling, machine learning, or nomograms [44,49,50,51,52,53,54]. For example, Corsi et al. proposed a model combining PSA density and two radiomics features (texture regularity from T2w and size zone heterogeneity from ADC) to identify csPCa among 80 PI-RADS 3 lesions, obtaining a sensitivity of 80% and a specificity of 76% [44]. Another group performed radiomics-based machine learning to screen for csPCa in 263 PI-RADS 3 lesions (139 PZ and 124 TZ) with integrated radiomic features of T2w, diffusion-weighted, and ADC images and yielded excellent AUCs [53]. Yet, in both studies, lesions were not considered separately by zone. For example, 82.5% of the included lesions in the study by Corsi et al. were localized in the peripheral zone, possibly leading to a bias in the constructed models. Since PI-RADS imaging criteria for peripheral and transition zone lesions are pivotally different, we believe that the zones should also be considered separately, as performed in the presented study [19,55]. Lim et al. attempted to predict csPCa in PI-RADS 3 lesions with a machine learning approach of radiomic features extracted from ADC and T2w in 160 PI-RADS 3 lesions (79 PZ, 81 TZ), including 29 csPCas. Their T2w-based models yielded an AUC of only 0.55, possibly because data from two institutions with different 3T MR scanners were included [49]. Moreover, they contoured target lesions with two-dimensional regions of interest instead of using whole-lesion 3D volumes of interest. Giambelluca et al. built models based on T2w and ADC images of 43 patients with 1–2 PI-RADS 3 lesions [52]. Their models yielded AUCs of, at best, 0.77 for T2w, and they also performed only two-dimensional segmentation. Since two-dimensional segmentation conceivably leads to missing data, all target lesions were segmented with whole-lesion 3D VOIs in the presented study. Hectors et al. constructed a machine learning model based on radiomics from T2w images of PI-RADS category 3 lesions to identify csPCa, yielding an AUC of, at best, 0.76 [51]. The parametric map approach of the presented study outperforms most of the studies mentioned above in diagnostic performance. Also, the “black box” characteristics of complex modeling in previous studies often render the presented data ungraspable [56]. Yet, this study attains higher transparency, as the obtained results can be visually retraced and metrically reproduced in the maps.

Our study has some limitations. Because of the limited traceability of outpatient biopsies, the patient population is small despite the long retrospective screening period. Also, the number of patients with csPCa is rather small. Furthermore, the median age of the patients was 62–68 years. As the peripheral zone presents differently according to age [57], it is rather unlikely that our results are transferrable to a younger patient collective (around <55 years of age). Another limitation is that maps were only computed for T2w images, not DWI or DCE images, which might enhance discriminative performance in combination with the T2w-derived parametric maps.

## 5. Conclusions

The parametric feature maps computed from standard axial T2w MR images of the prostate allow the direct visualization and quantification of radiomic features. The feature maps can be a diagnostic aid in PI-RADS category 3 lesions in prostate MRI, which are equivocal and challenging in clinical care. The map-derived features alone detect the infrequently occurring csPCa in PI-RADS 3 lesions noninvasively (AUCs: 0.70–0.84). Combining the feature maps with PSAD in bivariate models shows outstanding diagnostic performance (AUCs > 0.91), yet the small number of included patients limits the generalizability of these results. A future perspective would be an integrated software pipeline in clinical routine imaging, automatically providing the radiomic feature maps for each prostate MRI. The parametric maps could serve as a quantitative and objective tool for radiologists to reduce the ambiguity of PI-RADS category 3 lesions and contribute to the prevention of unnecessary biopsies.

## Figures and Tables

**Table 1:** Details of the included Patients.

Study Group	Non-csPCa		csPCa	
Biopsy Result	No PCa	ciPCa	csPCa	
Zone of Target Lesion	PZ TZ	PZ TZ	PZ TZ	
Number of Patients	25 8	4 4	8 3	
Median age (IQR) (y)	62 (59–65)	69 (58.3–70.5)	68 (62.5–73.5)	
Median weight (IQR) (kg)	83 (75–90)	73.5 (70.8–86)	90 (74–90)	
Median initial PSA (IQR) (ng/mL)	6 (5.1–7.9)	7.45 (6.1–8.7)	9.7 (6.4–16.3) *	
Median prostate volume (IQR) (mL)	62 (46–75)	47 (35.5–51.8)	46 (38.5–49)	
Median PSAD ((ng/mL)/cm^3^) ***	0.10 (0.1–0.1)	0.16 (0.1–0.2)	0.29 (0.2–0.4) *	
Median days between MRI and biopsy (IQR)	54.5 (35.3–78.8) **	29.5 (24–130.8)	40 (24.5–45.5) **	
Gleason Score (number of patients)	-	3 + 3 (8)	3 + 4 (3)	3 + 4 (1)
			4 + 3 (3)	4 + 3 (1)
			4 + 5 (2)	4 + 5 (1)
Median tumor volume (%) (IQR)		28 (7–47)		
Extraprostatic extension of cancer ****	-	-	2 patients	-

PCa: prostate cancer; ciPCa: clinically insignificant PCa; csPCa: clinically significant prostate cancer; PZ: peripheral zone; TZ: transition zone; PSA: serum prostate-specific antigen; PSAD: serum prostate-specific antigen density (PSAD) level; * one data point is missing. ** One data point is missing since, in one patient, only the biopsy result is available, but not the exact biopsy date. *** Calculated from initial PSA and prostate volume derived from MRI. **** according to the histopathological result.

## Data Availability

The datasets are available from the corresponding author on reasonable request.

## Supplementary Materials

The following supporting information can be downloaded at: https://www.mdpi.com/article/10.3390/curroncol31110503/s1, File S1: Axial T2 TSE sequence technical details, File S2: Software Script, File S3: MWU results (feature maps) for all zones, File S4: MWU results (feature maps) for the peripheral zone only, File S5: MWU results (feature maps) for the transition zone only, File S6: ROC analysis (feature maps) for all zones, File S7: ROC analysis (feature maps) for peripheral zone only, File S8: ROC analysis (feature maps) for transition zone only, File S9: ROC plots (feature maps) for all zones, File S10: ROC plots (feature maps) for the peripheral zone only, File S11: ROC plots (feature maps) for the transition zone only, File S12: ROC analysis and bootstrapping of generalized linear models for all zones, File S13: ROC analysis and bootstrapping of generalized linear models for peripheral zone only, File S14: ROC analysis and bootstrapping of generalized linear models for transition zone only, File S15: ICCs (Interrater agreement).
