# Supplementary material for: Detecting Clinically Significant Prostate Cancer in PI-RADS 3 Lesions Using T2w-Derived Radiomics Feature Maps in 3T Prostate MRI
Source: Curr Oncol. 2024 Nov 1;31(11):6814–28. doi: 10.3390/curroncol31110503 (PMC11592716; doi:10.3390/curroncol31110503)
Supplement: Supplementary file 1 [file curroncol-31-00503-s001.zip › S10_ROCplots_feature_maps_PZ_only.pdf]

firstorder_10Percentile

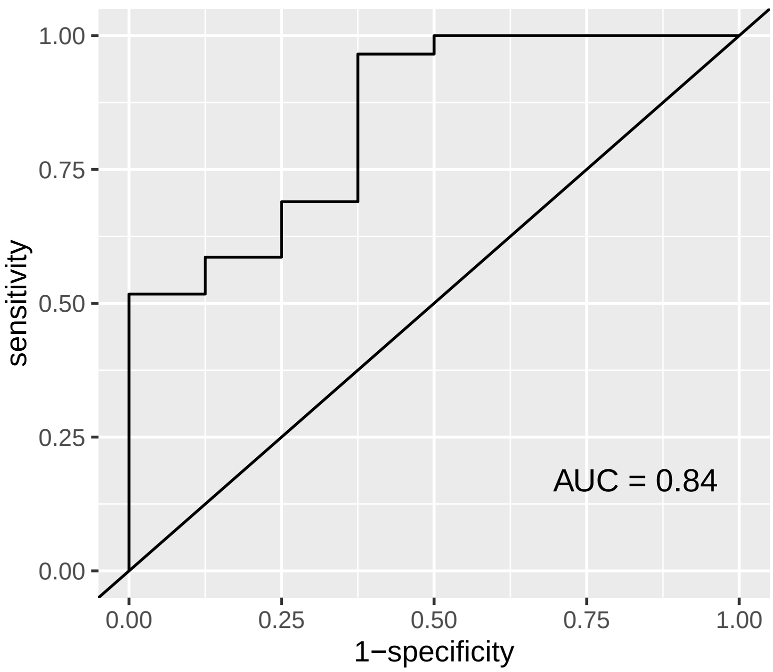

firstorder_Entropy

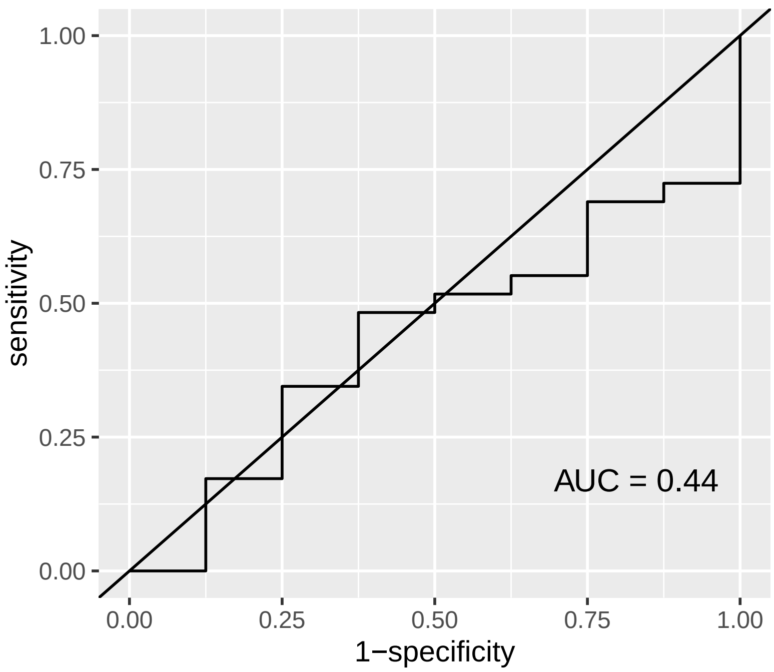

firstorder_90Percentile

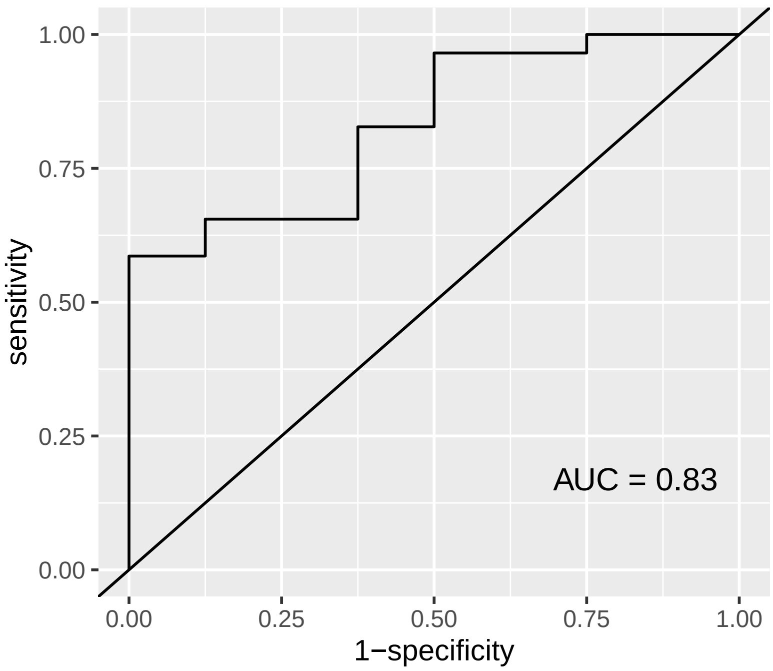

firstorder_InterquartileRange

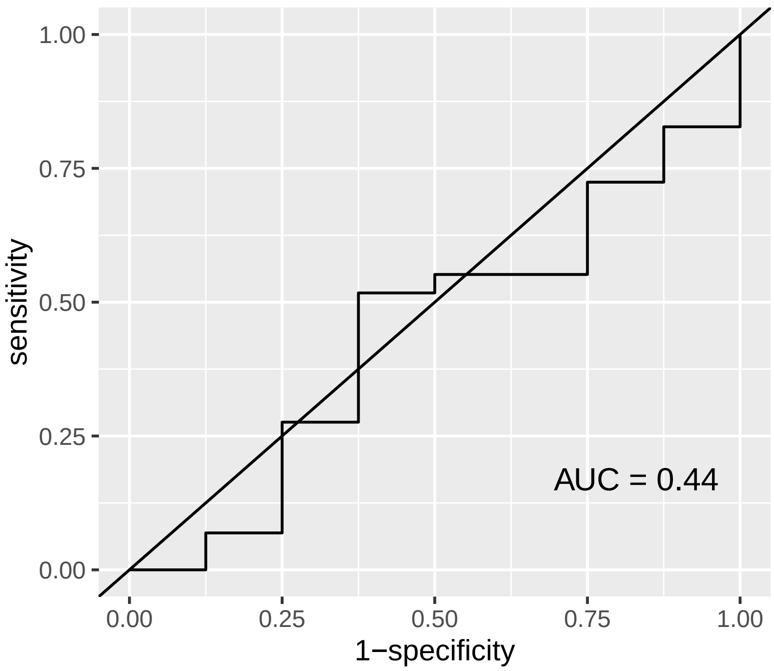

firstorder_Energy

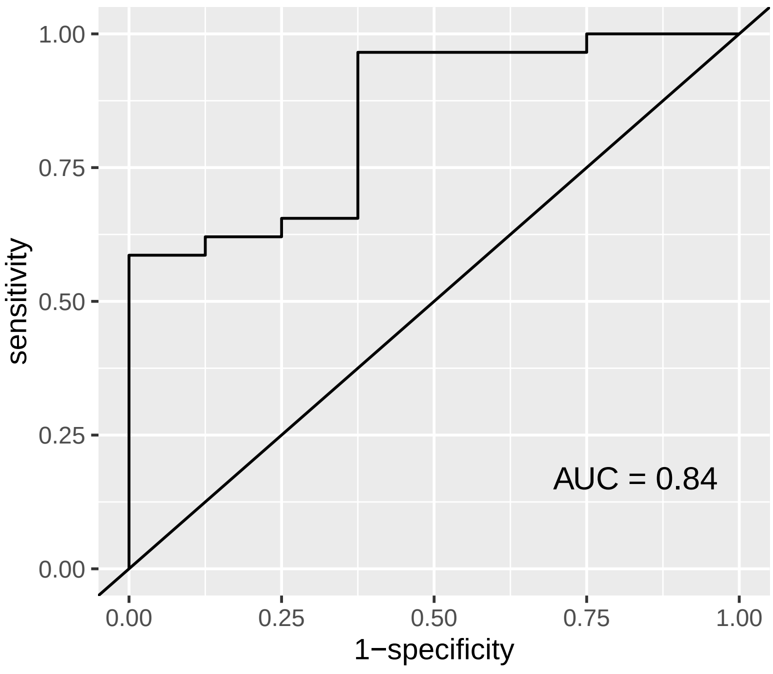

firstorder_Kurtosis

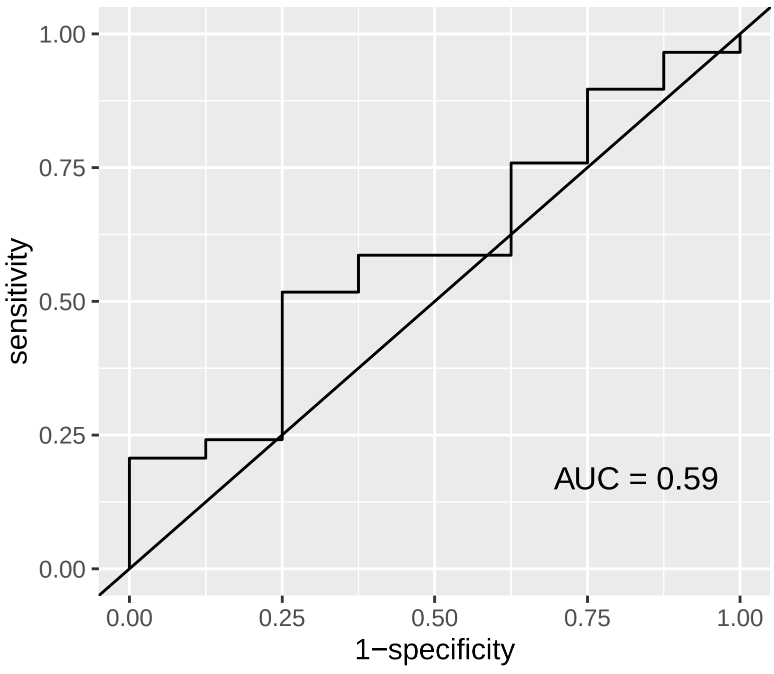

firstorder_Maximum

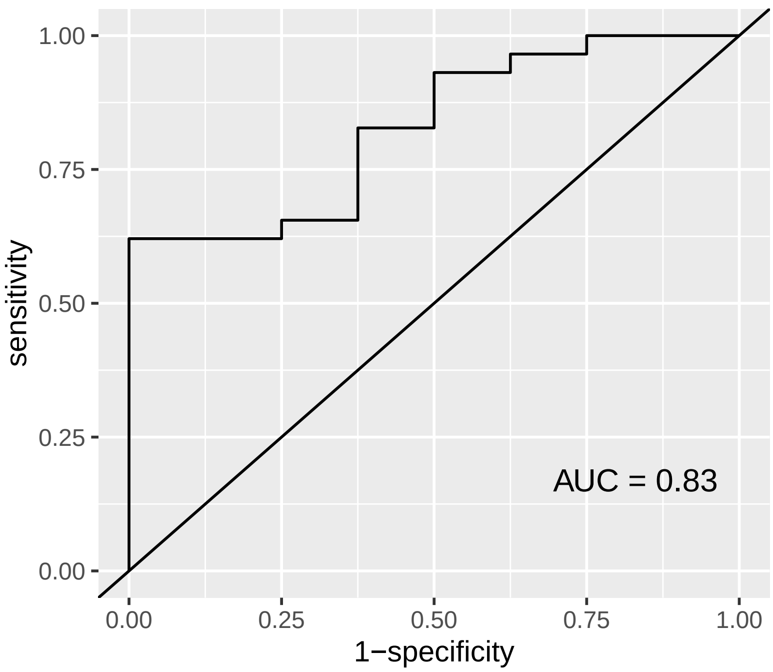

firstorder_Median

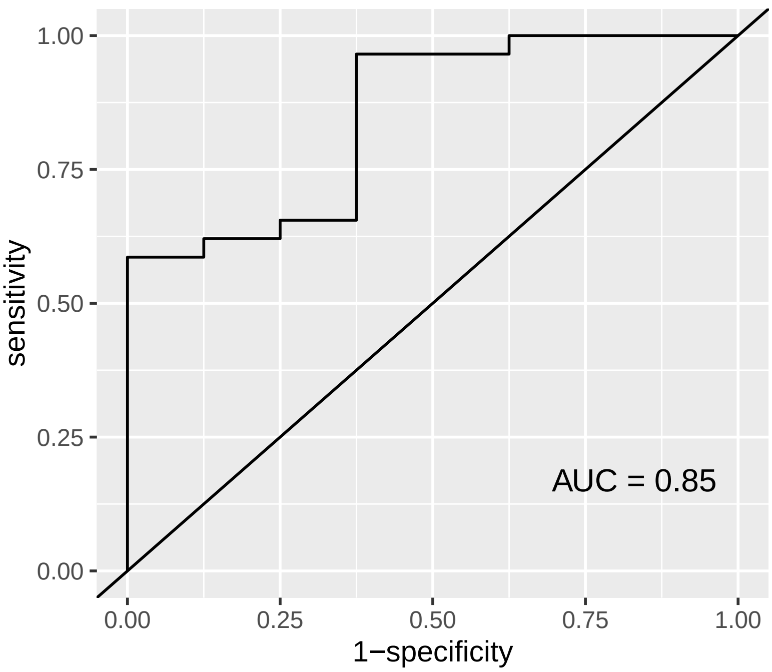

firstorder_Mean

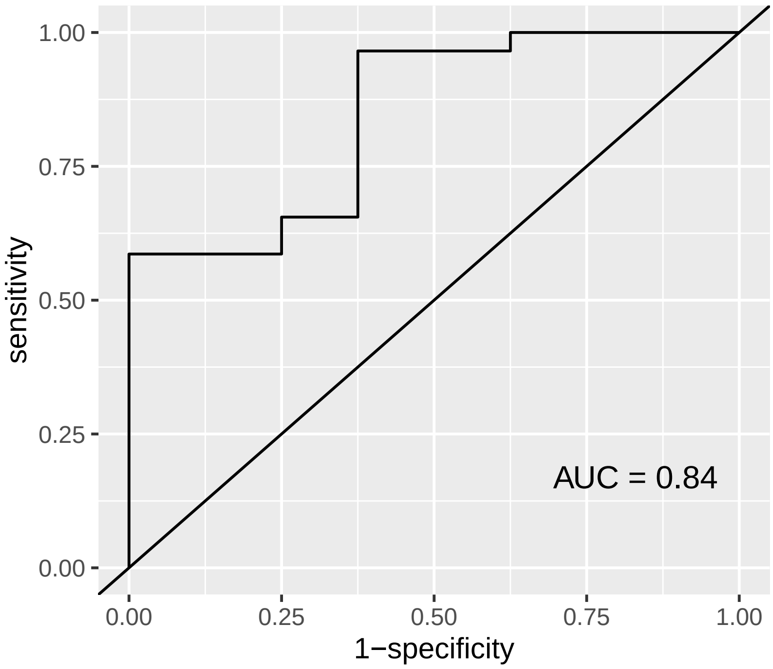

firstorder_Minimum

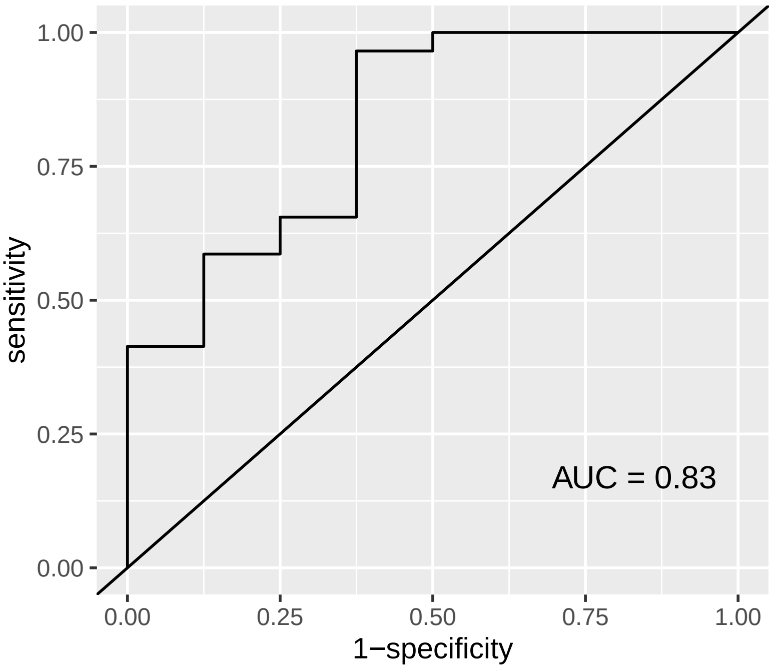

firstorder_MeanAbsoluteDeviation

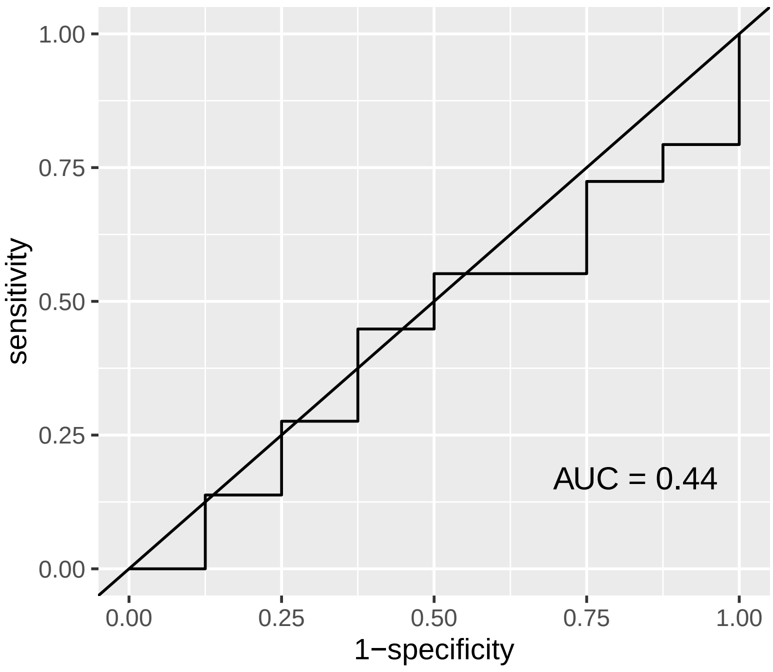

firstorder_Range

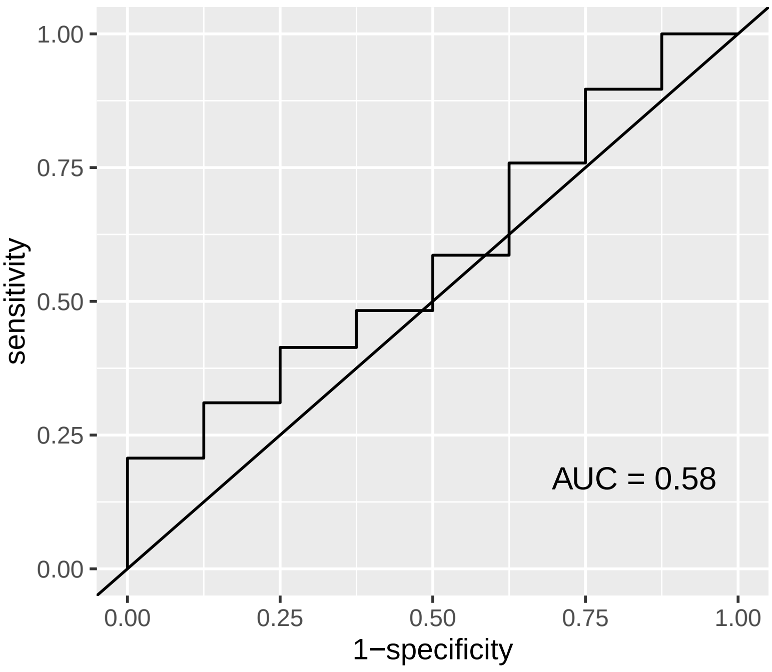

firstorder_RobustMeanAbsoluteDeviation

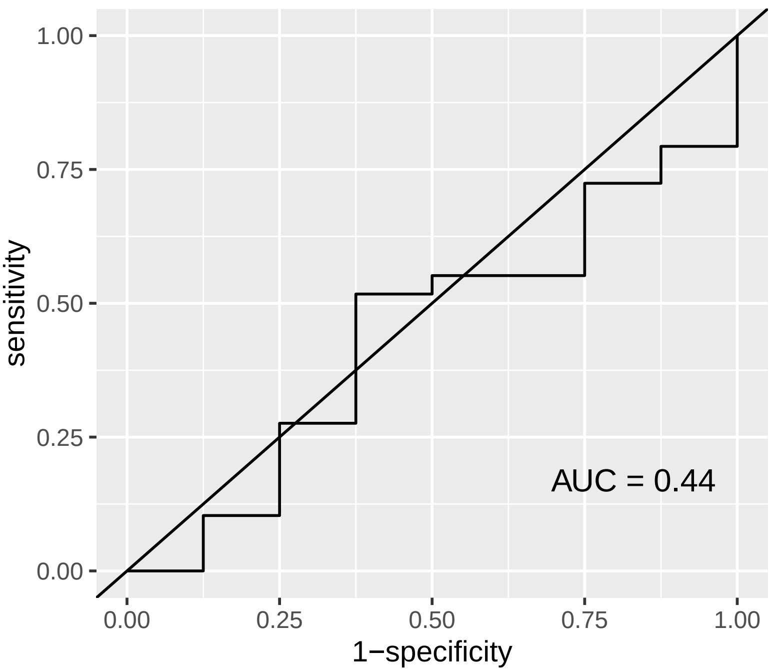

firstorder_TotalEnergy

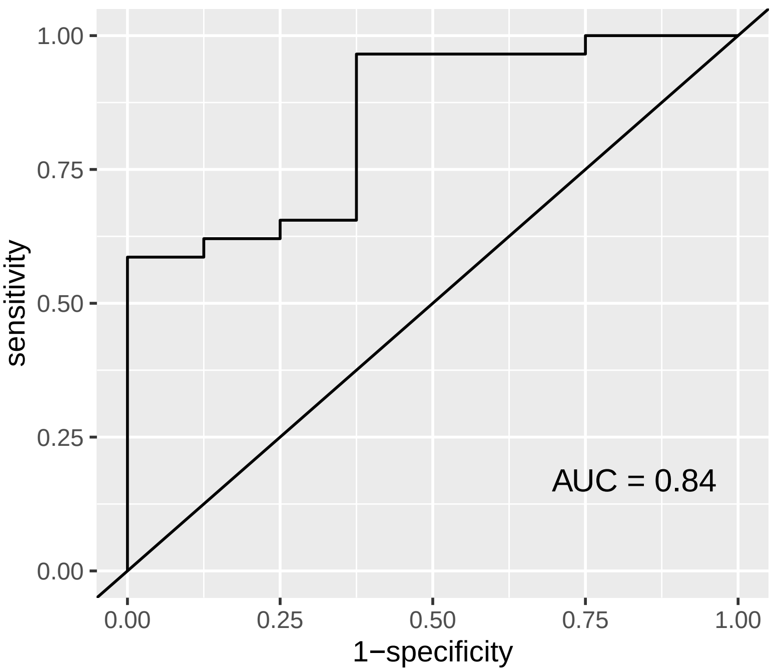

firstorder_RootMeanSquared

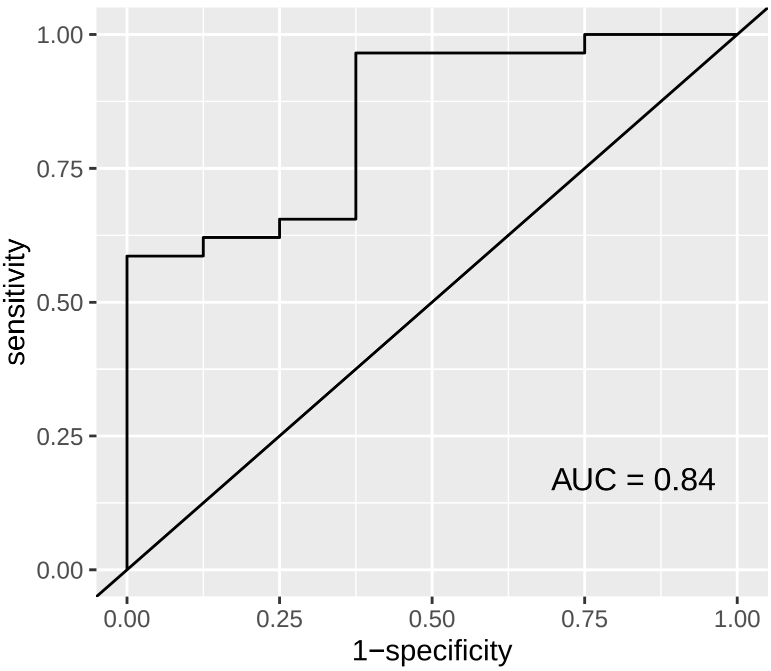

firstorder_Uniformity

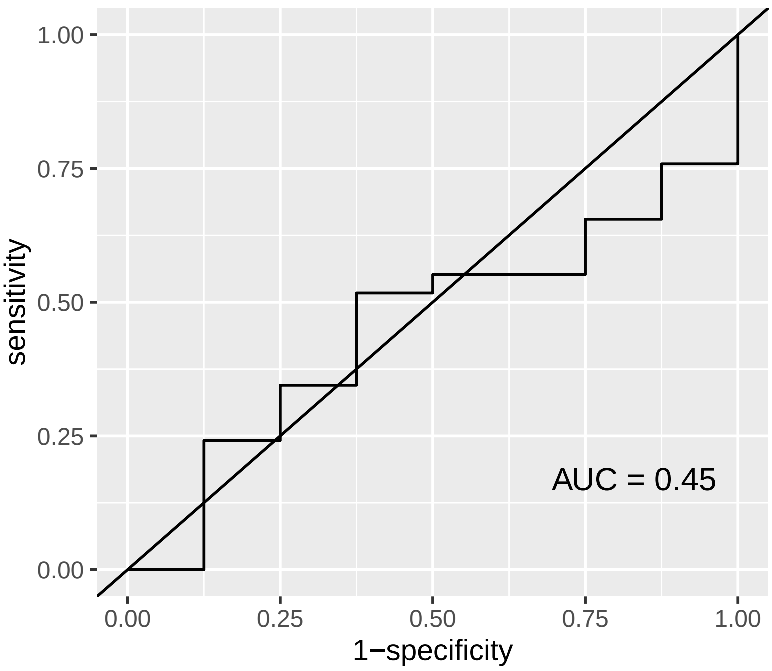

firstorder_Skewness

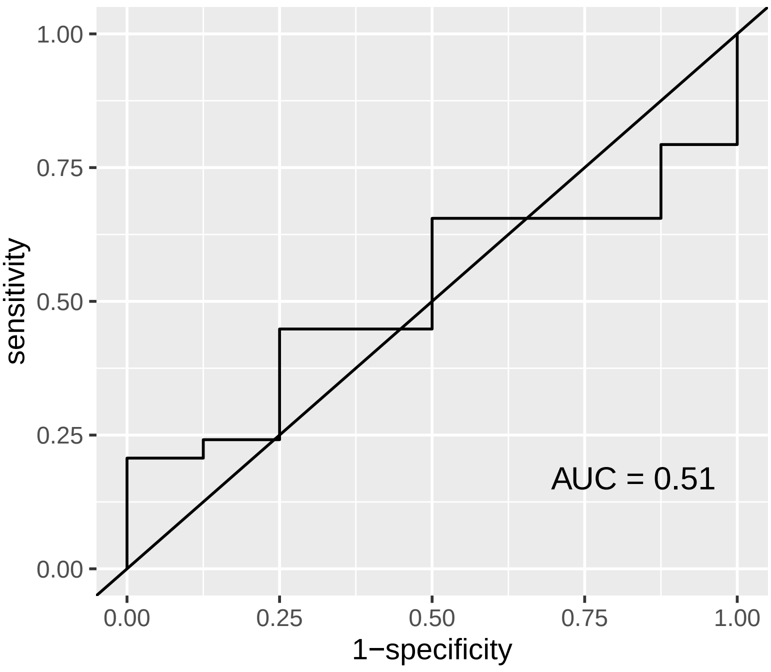

firstorder_Variance

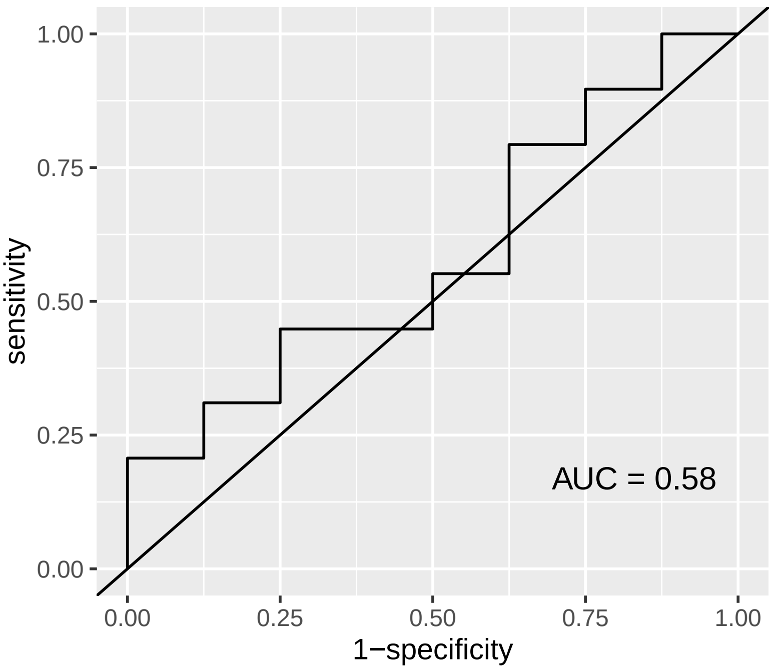

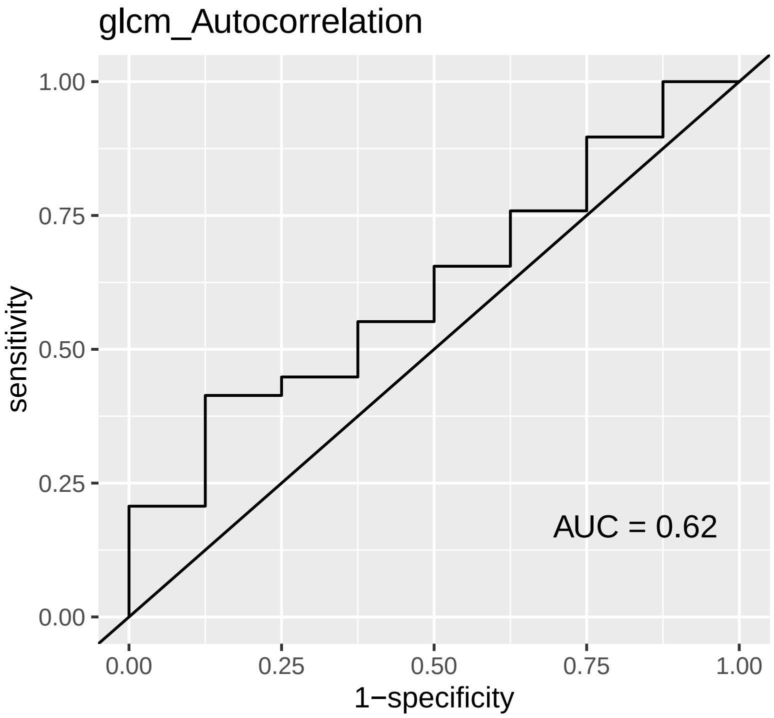
glcm_Autocorrelation
sensitivity
1−specificity
AUC = 0.62

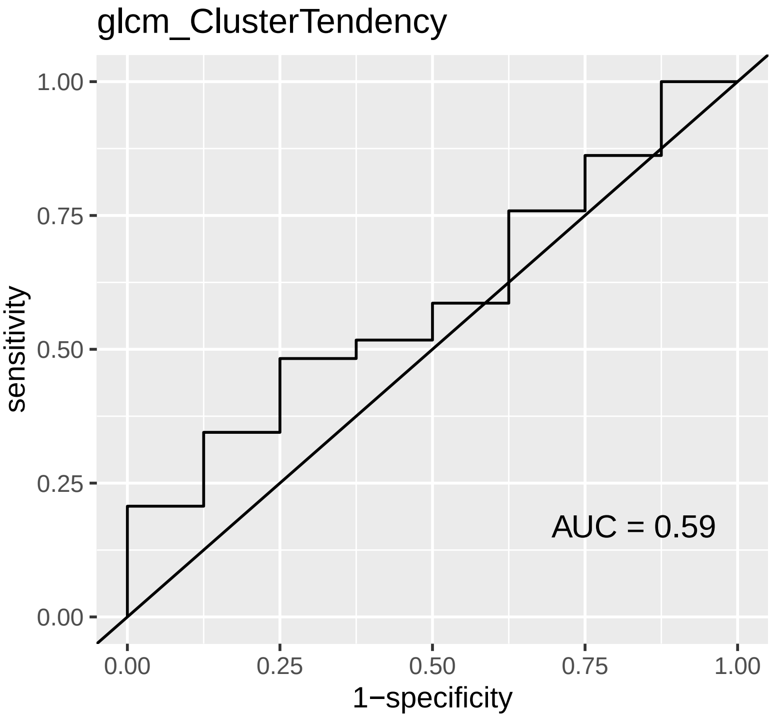
glcm_ClusterTendency
sensitivity
1−specificity
AUC = 0.59

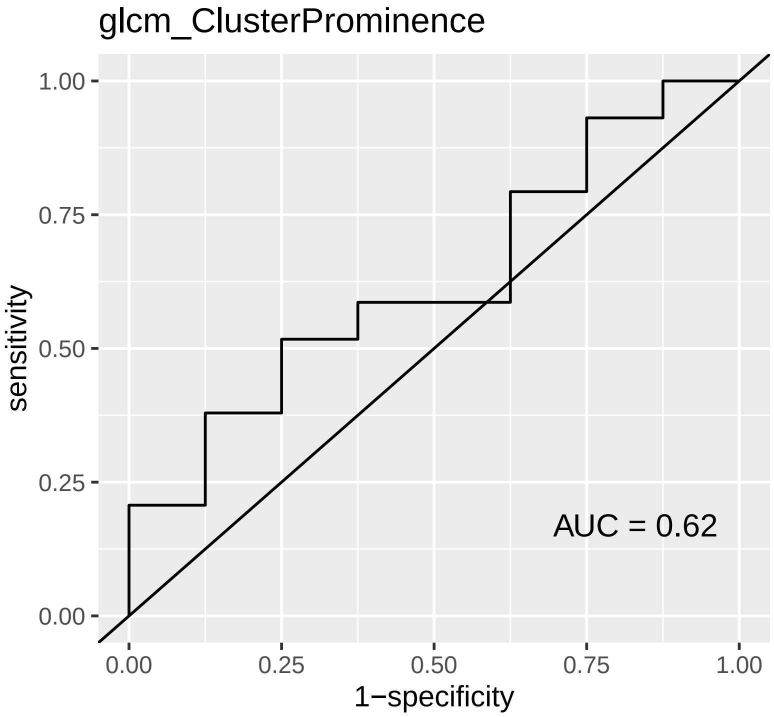
glcm_ClusterProminence
sensitivity
1−specificity
AUC = 0.62

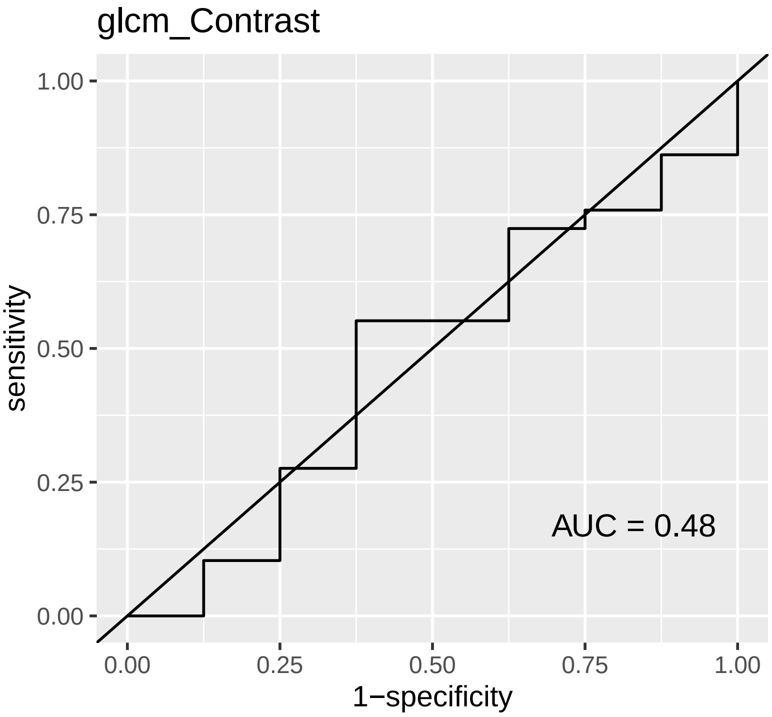
glcm_Contrast
sensitivity
1−specificity
AUC = 0.48

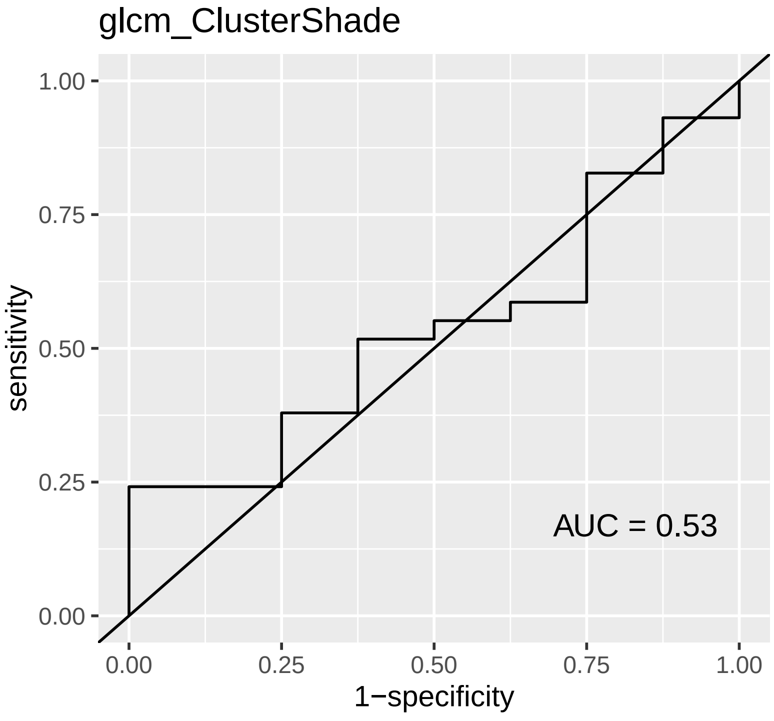
glcm_ClusterShade
sensitivity
1−specificity
AUC = 0.53

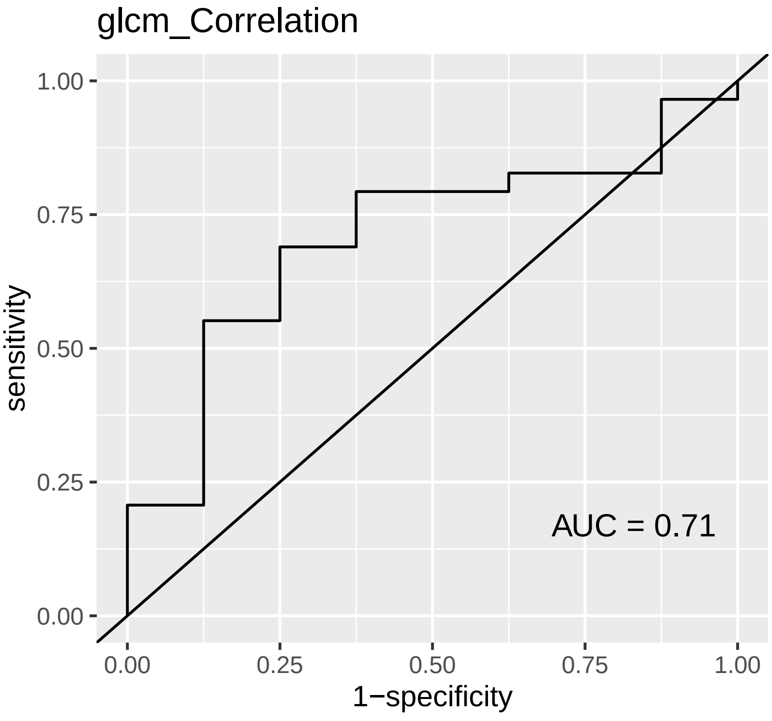
glcm_Correlation
sensitivity
1−specificity
AUC = 0.71

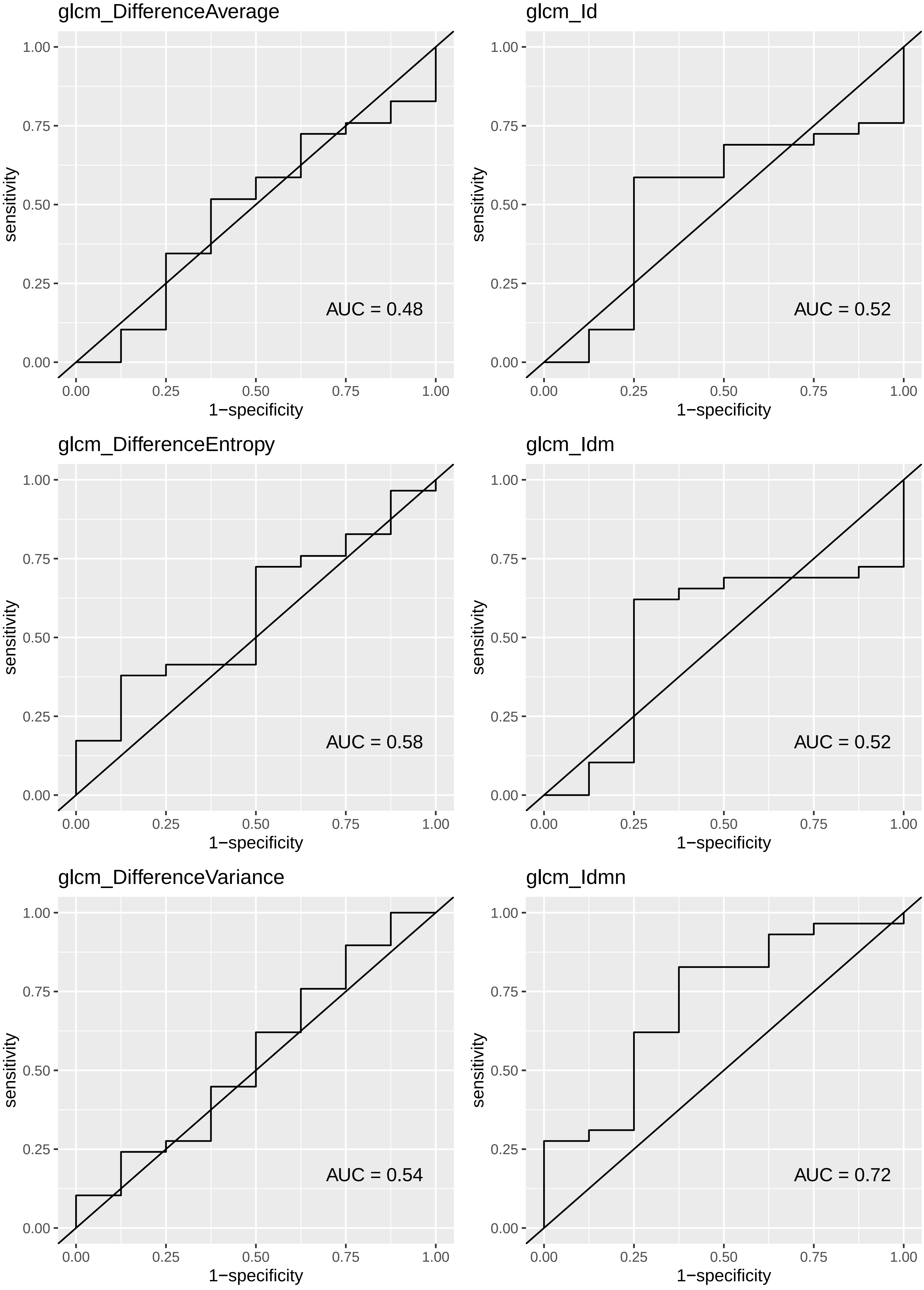
glcm_DifferenceAverage
sensitivity
1−specificity
AUC = 0.48
glcm_Id
AUC = 0.52
glcm_DifferenceEntropy
AUC = 0.58
glcm_Idm
AUC = 0.52
glcm_DifferenceVariance
AUC = 0.54
glcm_Idmn
AUC = 0.72
0.00
0.25
0.50
0.75
1.00

glcm_Idn

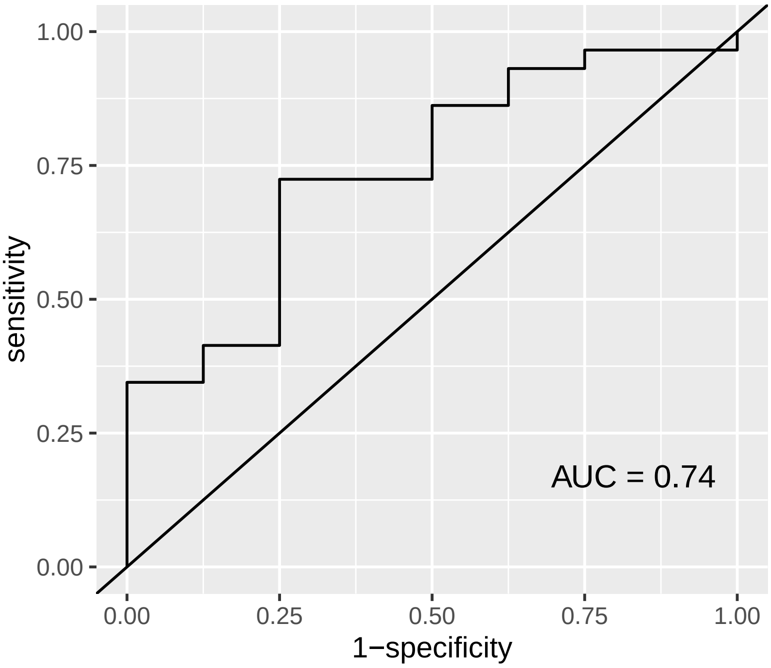

glcm_InverseVariance

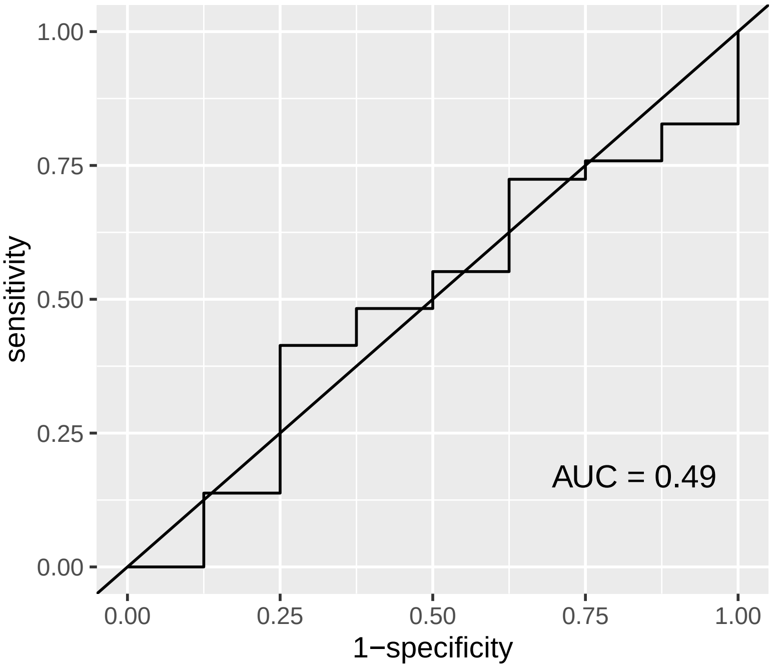

glcm_Imc1

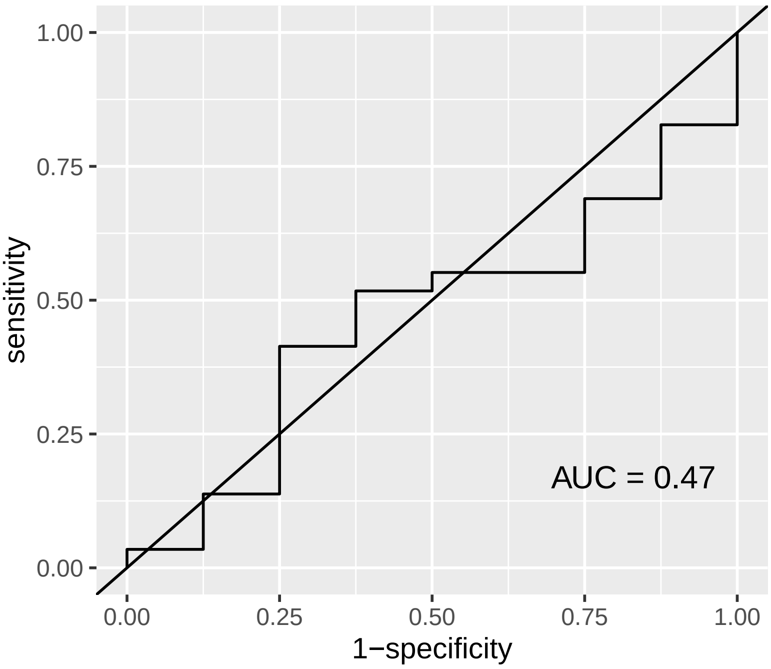

glcm_JointAverage

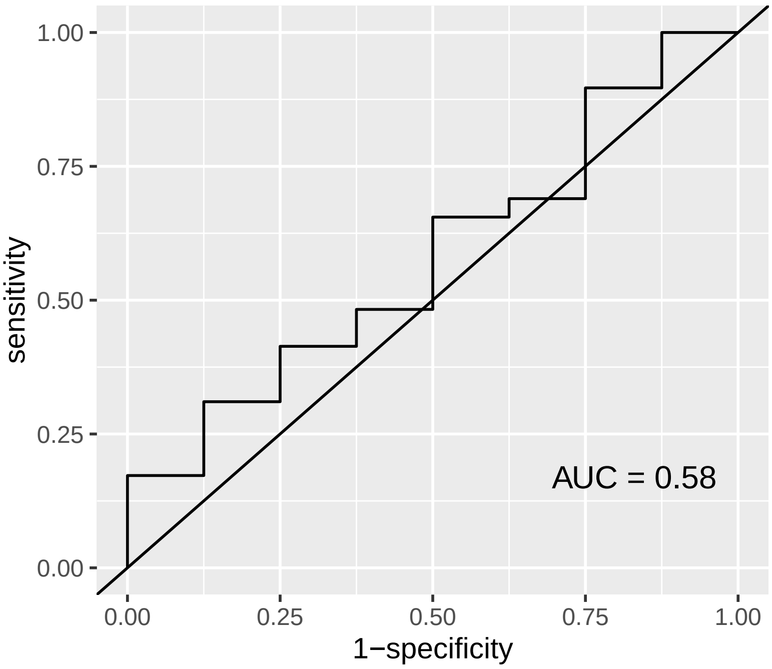

glcm_Imc2

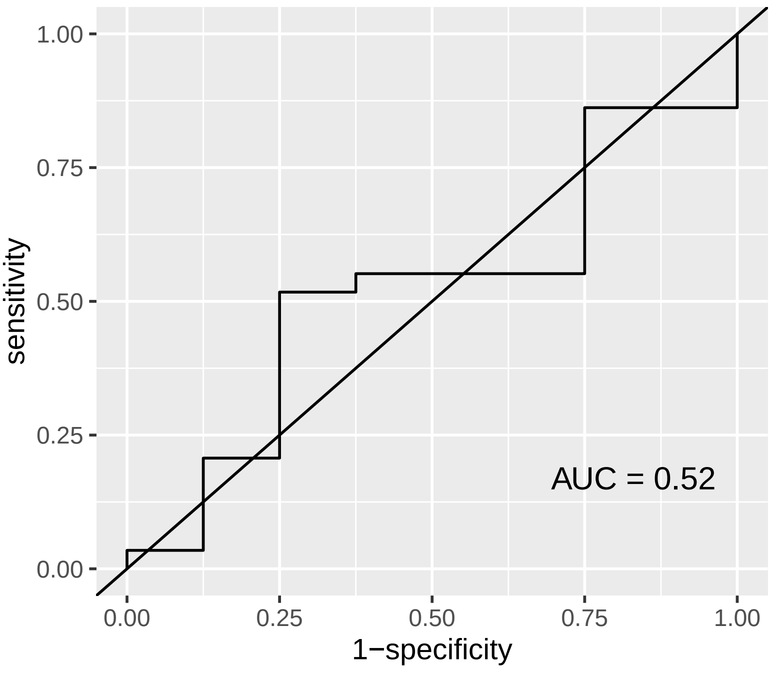

glcm_JointEnergy

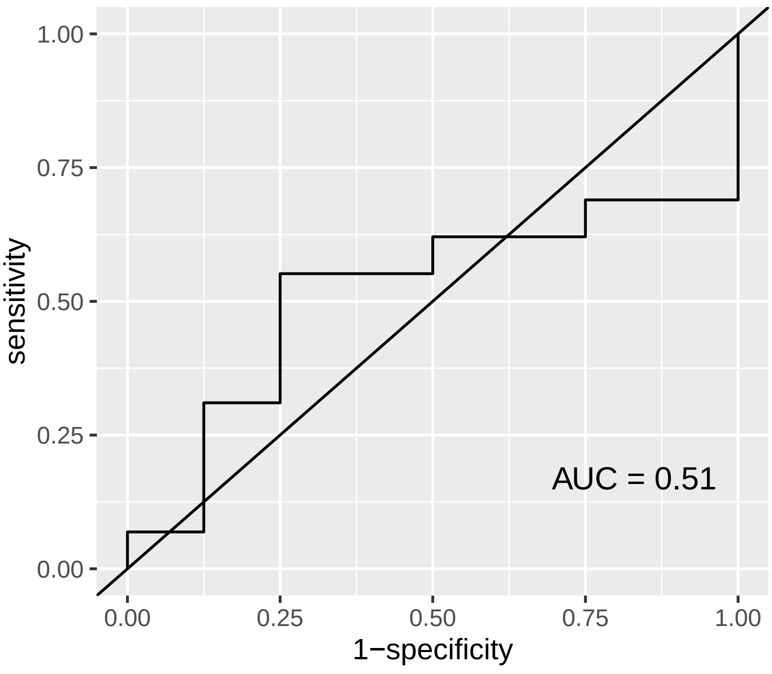

glcm_JointEntropy

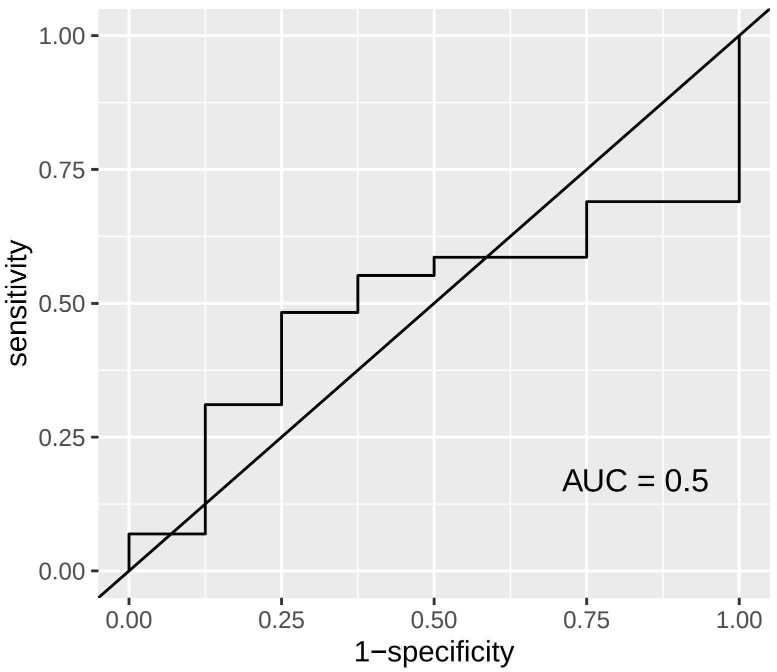

glcm_SumAverage

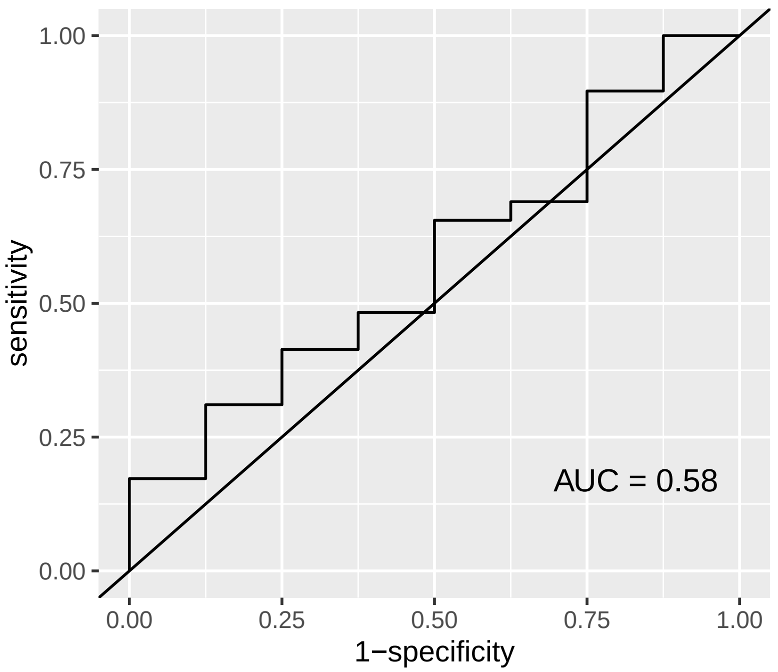

glcm_MaximumProbability

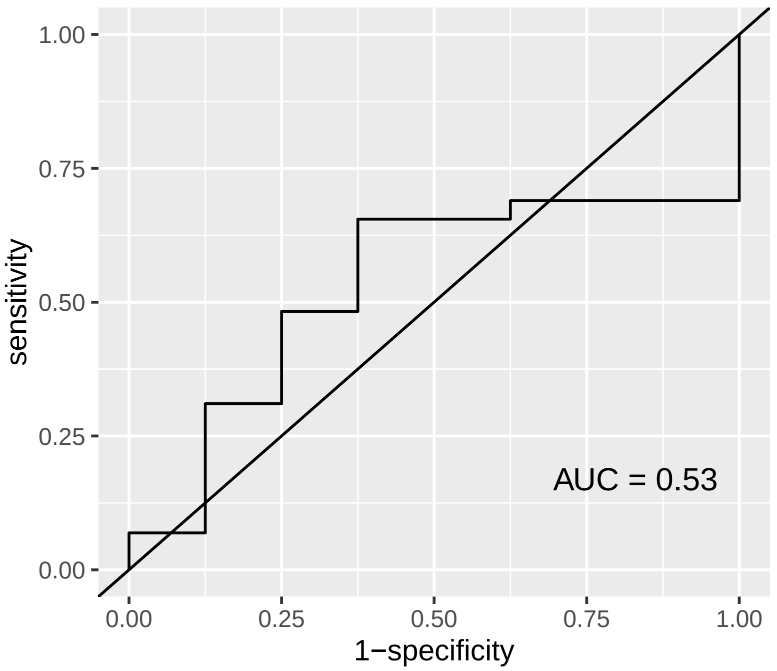

glcm_SumEntropy

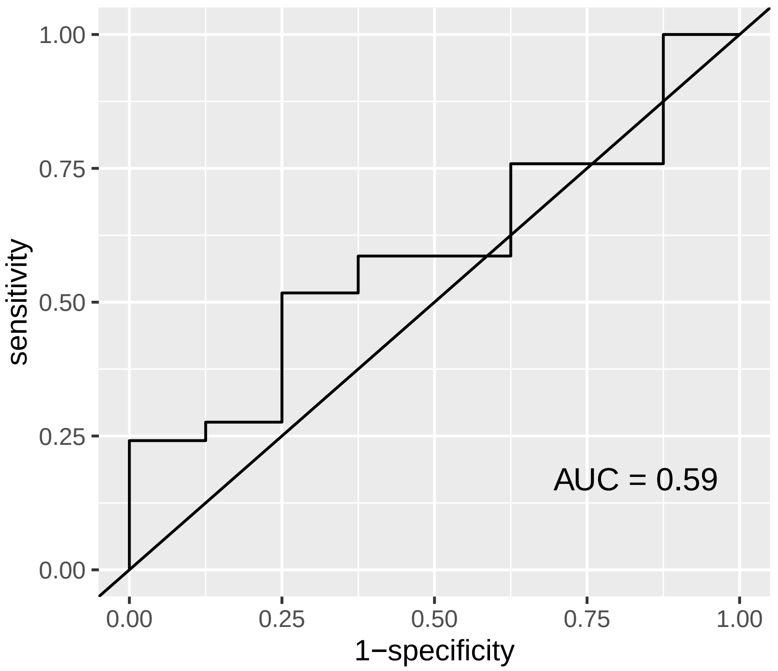

glcm_MCC

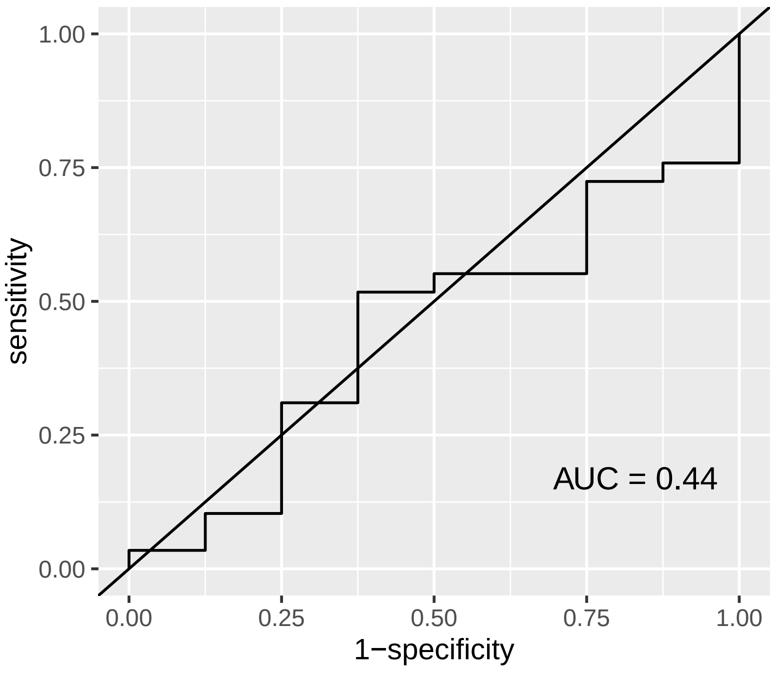

glcm_SumSquares

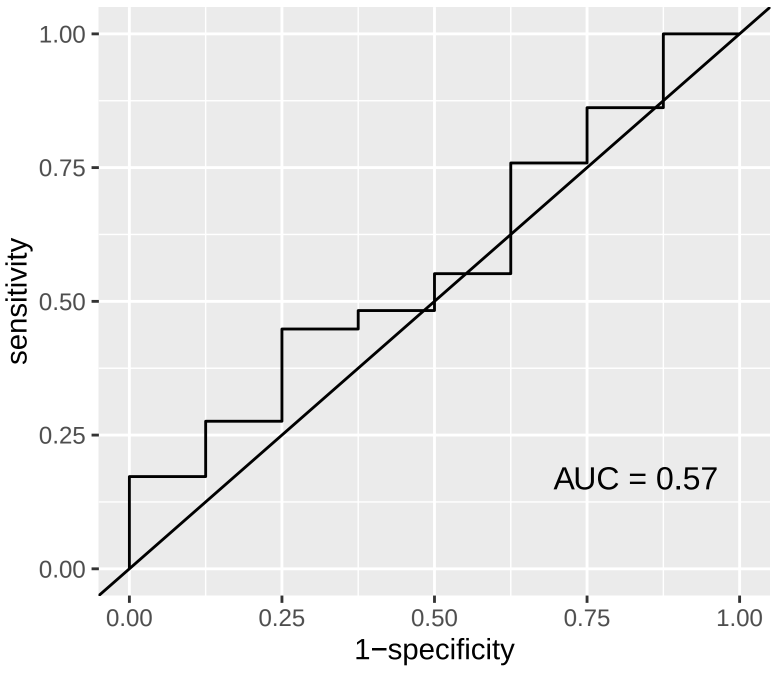

gldm_DependenceEntropy

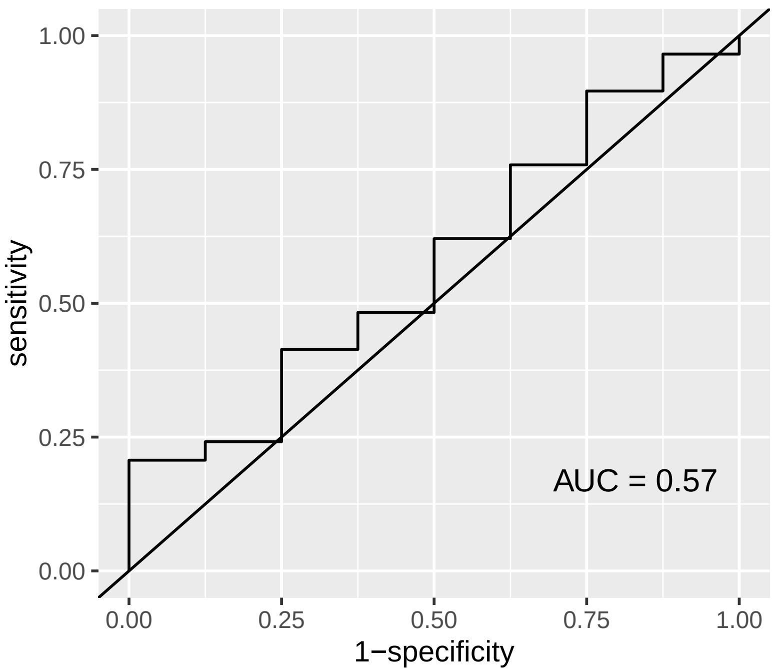

gldm_DependenceVariance

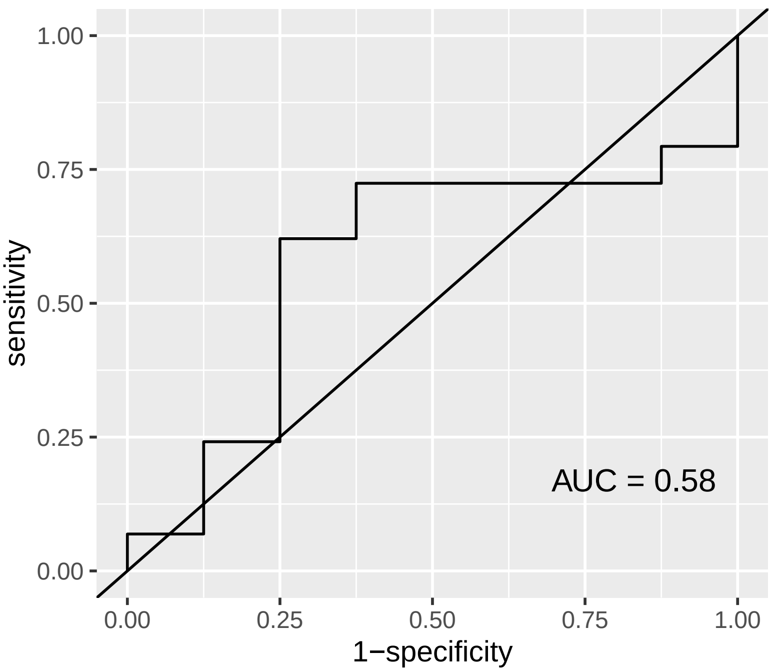

gldm_DependenceNonUniformity

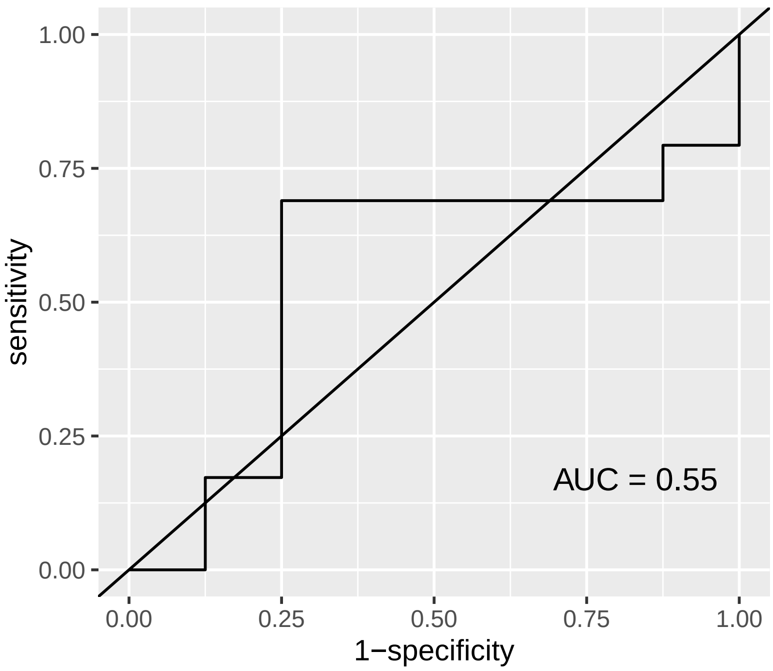

gldm_GrayLevelNonUniformity

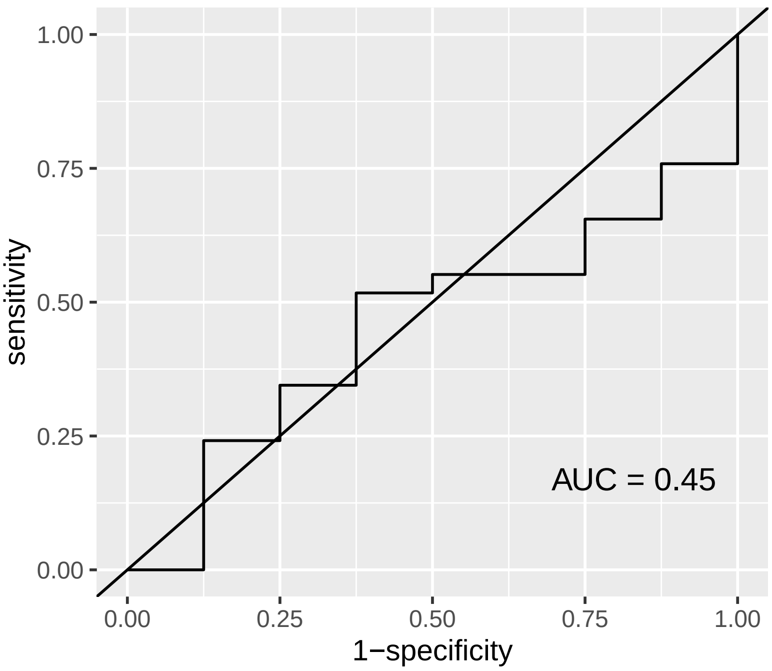

gldm_DependenceNonUniformityNormalized

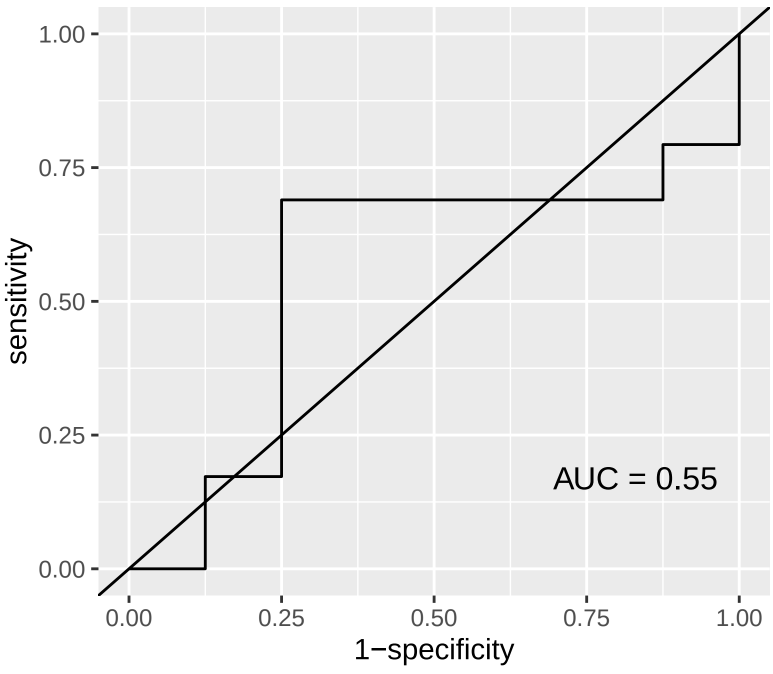

gldm_GrayLevelVariance

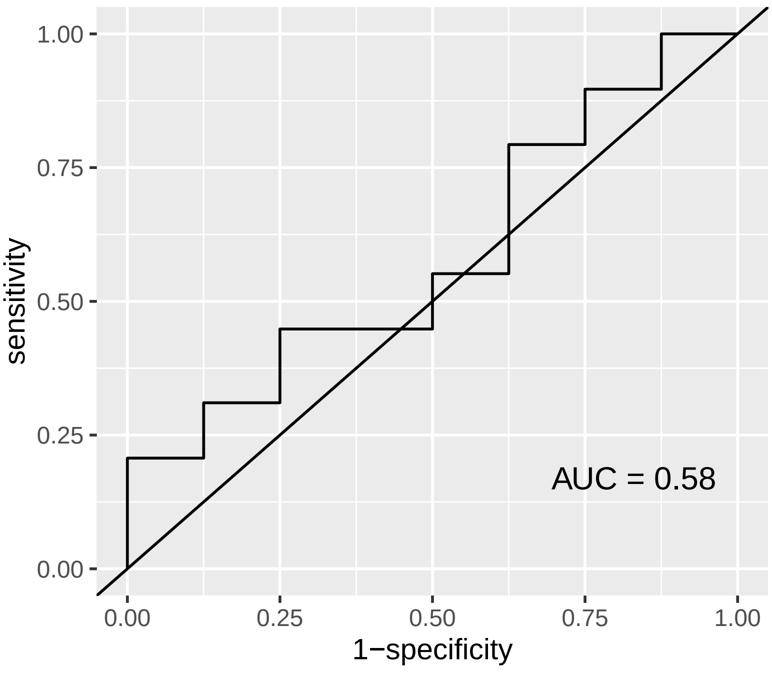

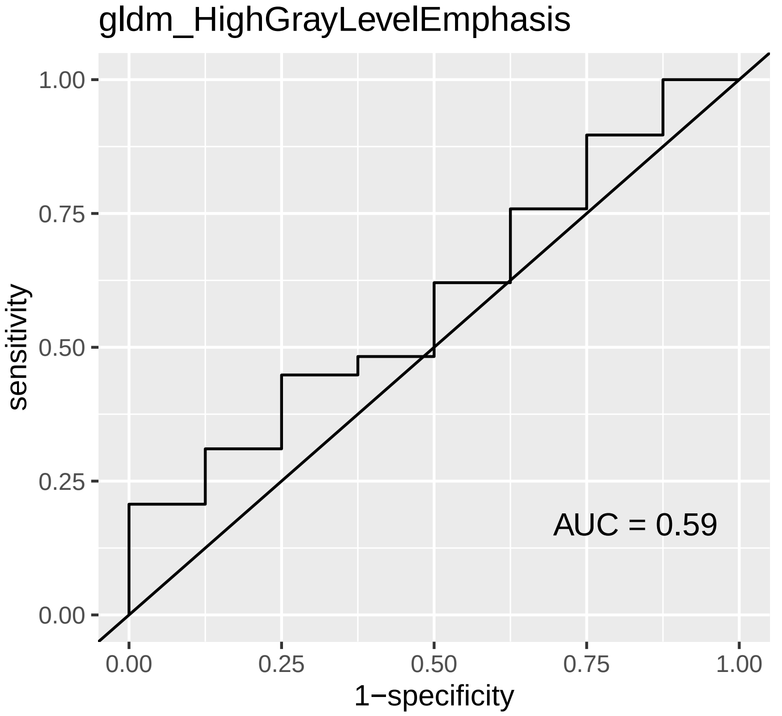
gldm_HighGrayLevelEmphasis
sensitivity
1−specificity
0.00
0.25
0.50
0.75
1.00
AUC = 0.59

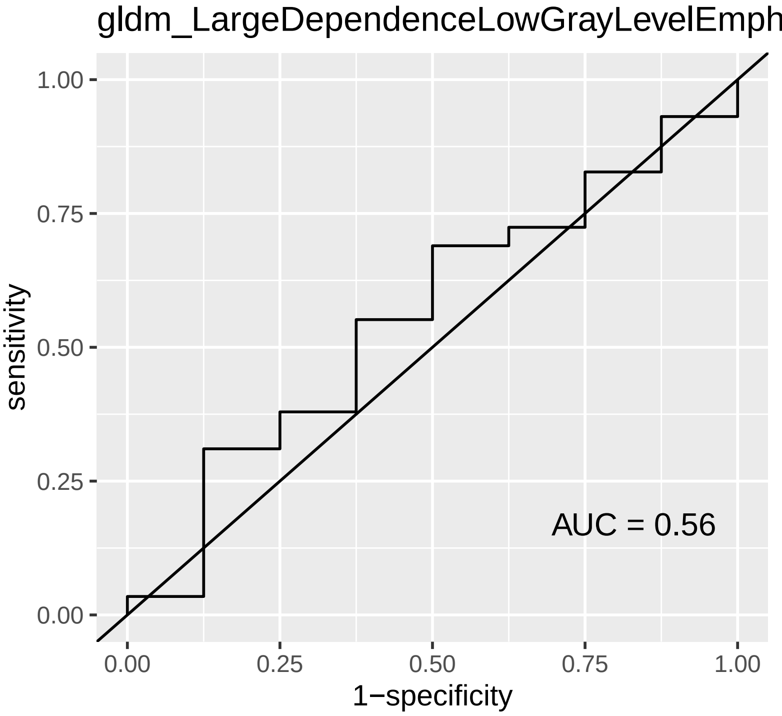
gldm_LargeDependenceLowGrayLevelEmph
sensitivity
1−specificity
0.00
0.25
0.50
0.75
1.00
AUC = 0.56

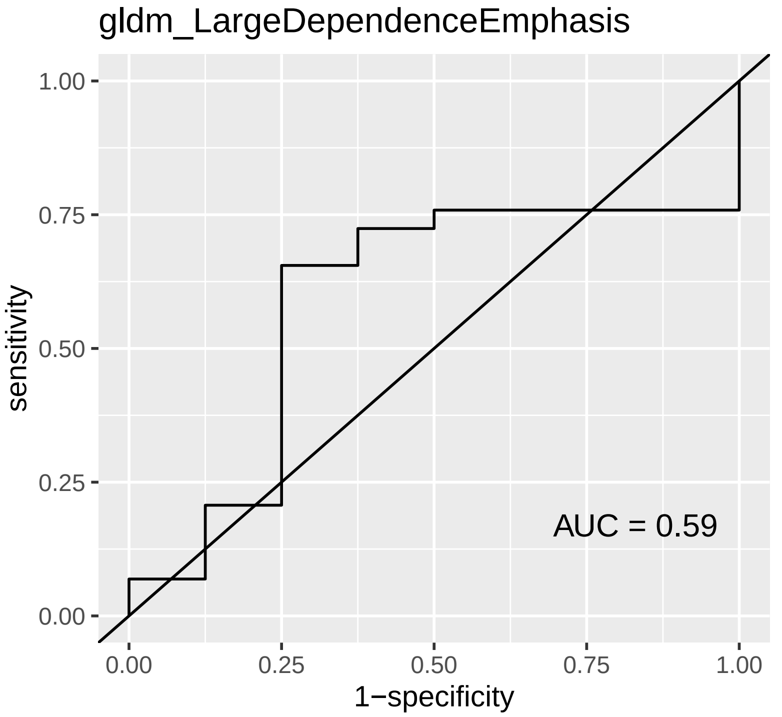
gldm_LargeDependenceEmphasis
sensitivity
1−specificity
0.00
0.25
0.50
0.75
1.00
AUC = 0.59

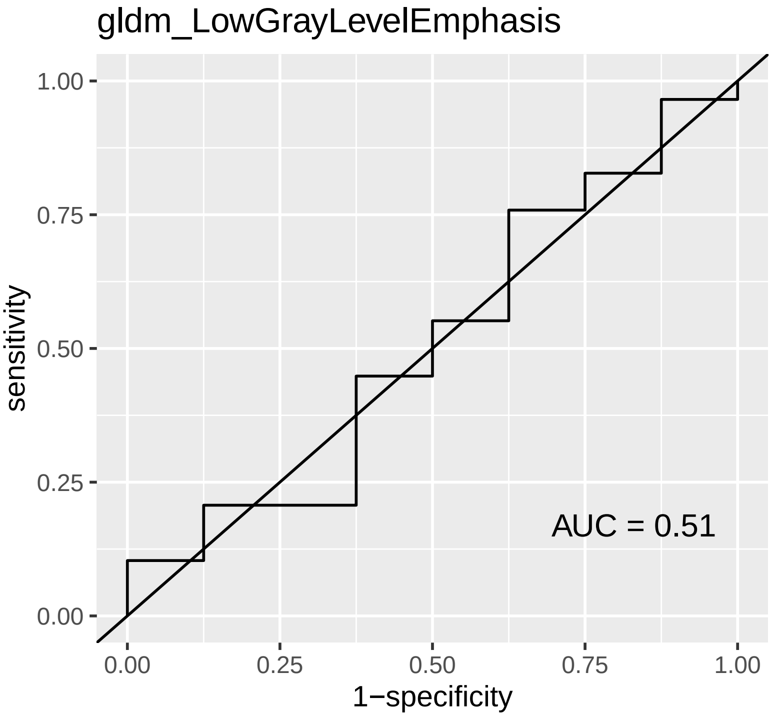
gldm_LowGrayLevelEmphasis
sensitivity
1−specificity
0.00
0.25
0.50
0.75
1.00
AUC = 0.51

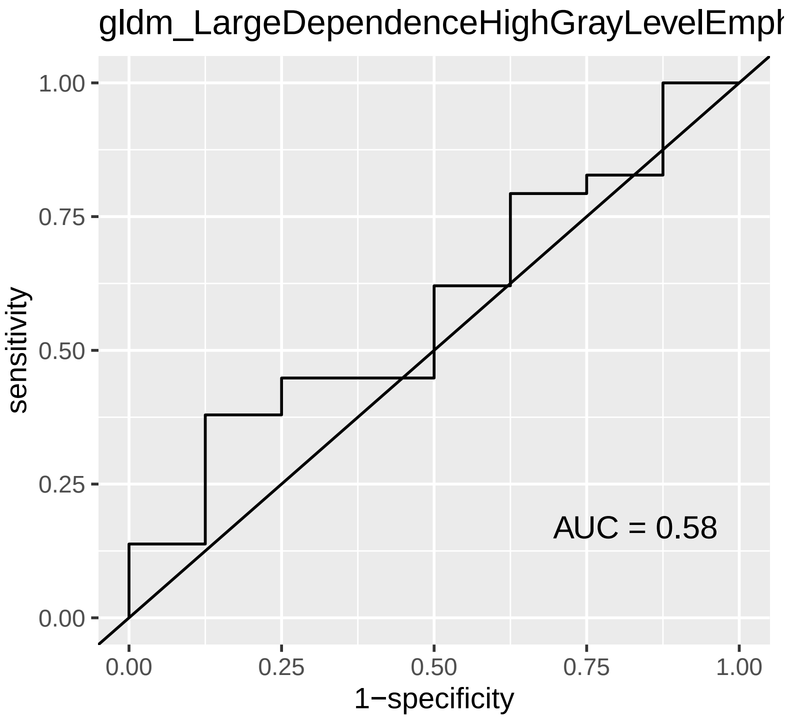
gldm_LargeDependenceHighGrayLevelEmph
sensitivity
1−specificity
0.00
0.25
0.50
0.75
1.00
AUC = 0.58

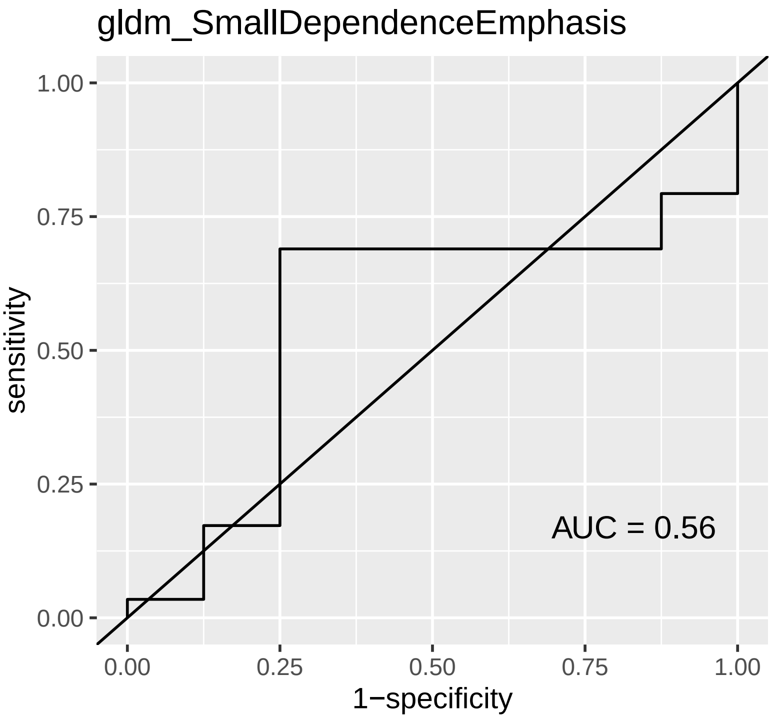
gldm_SmallDependenceEmphasis
sensitivity
1−specificity
0.00
0.25
0.50
0.75
1.00
AUC = 0.56

gldm_SmallDependenceHighGrayLevelEmph

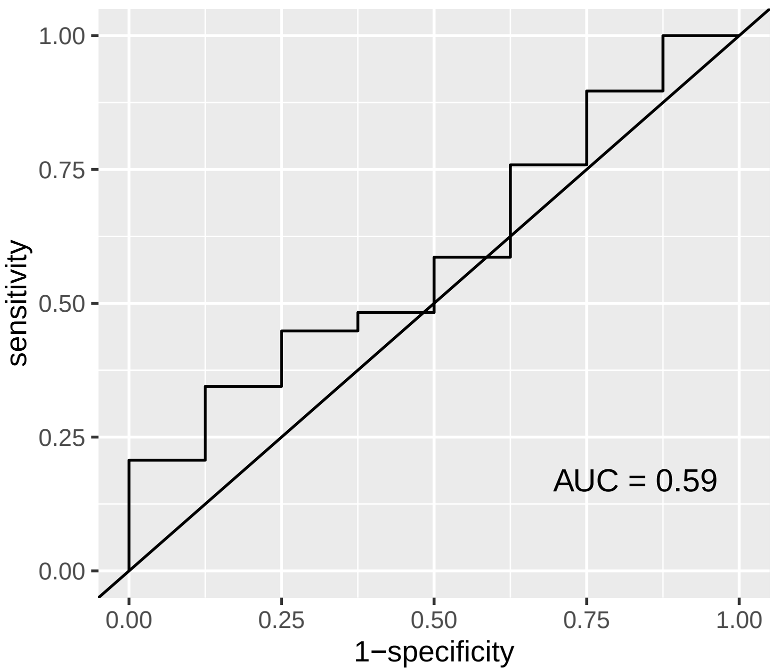

glrlm_GrayLevelNonUniformityNormalized

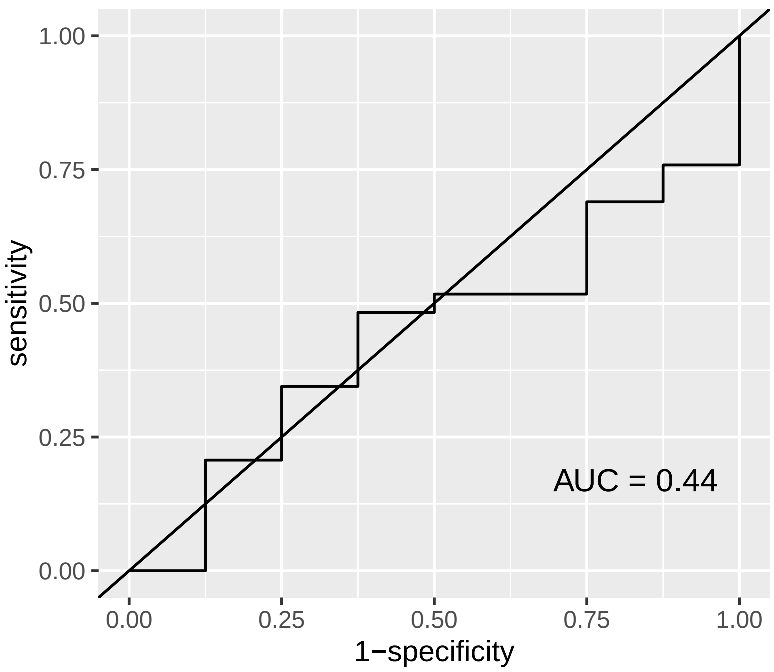

gldm_SmallDependenceLowGrayLevelEmph

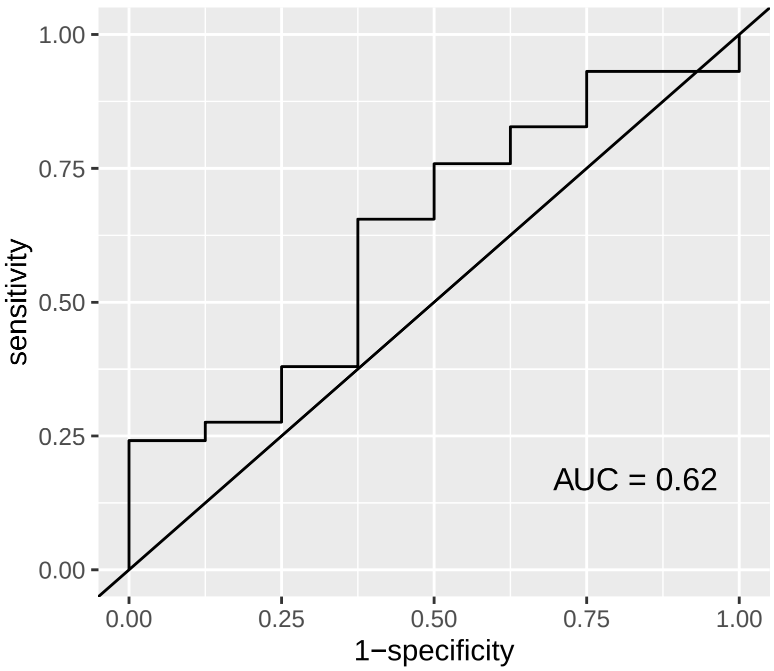

glrlm_GrayLevelVariance

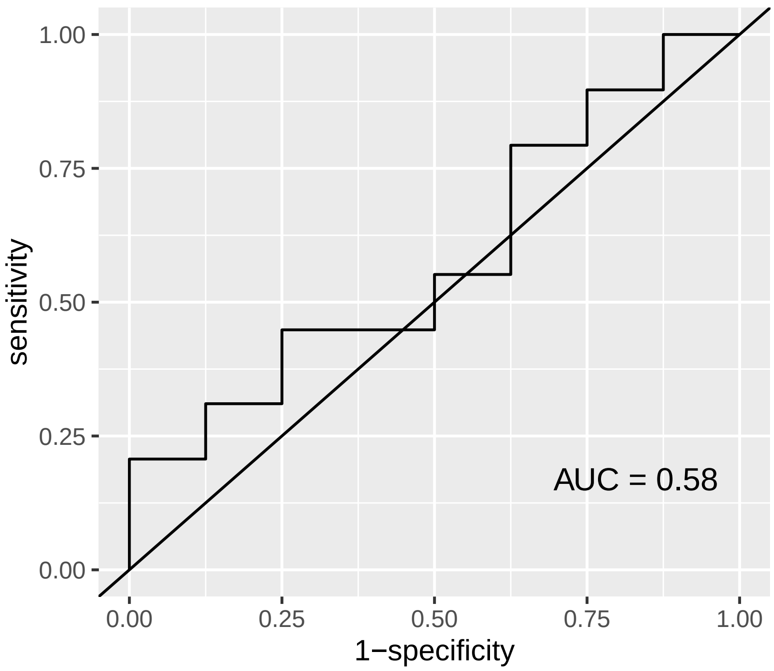

glrlm_GrayLevelNonUniformity

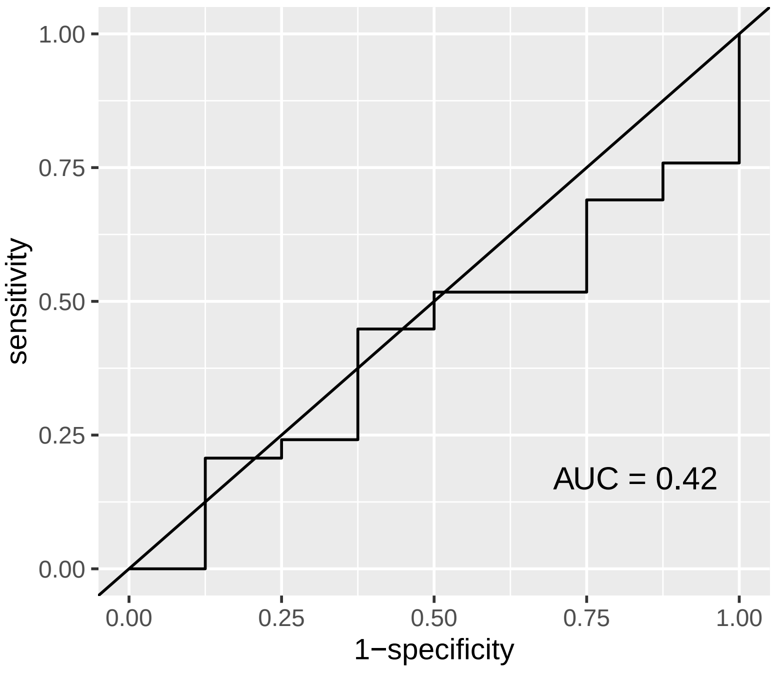

glrlm_HighGrayLevelRunEmphasis

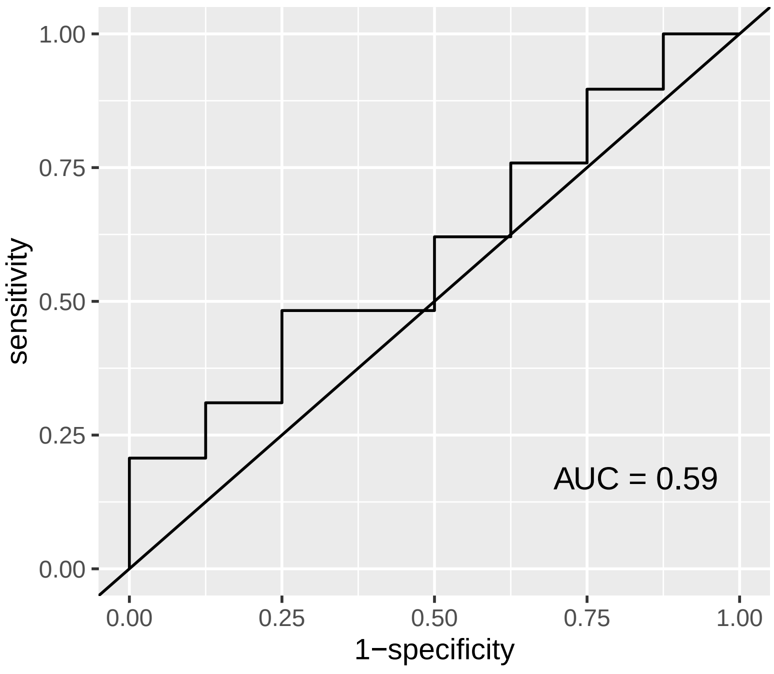

glrlm_LongRunEmphasis

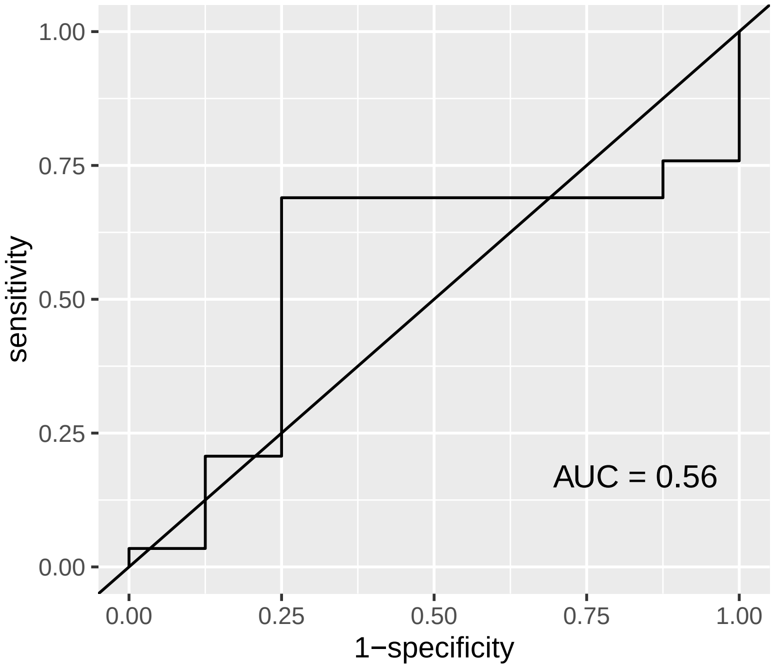

glrlm_LowGrayLevelRunEmphasis

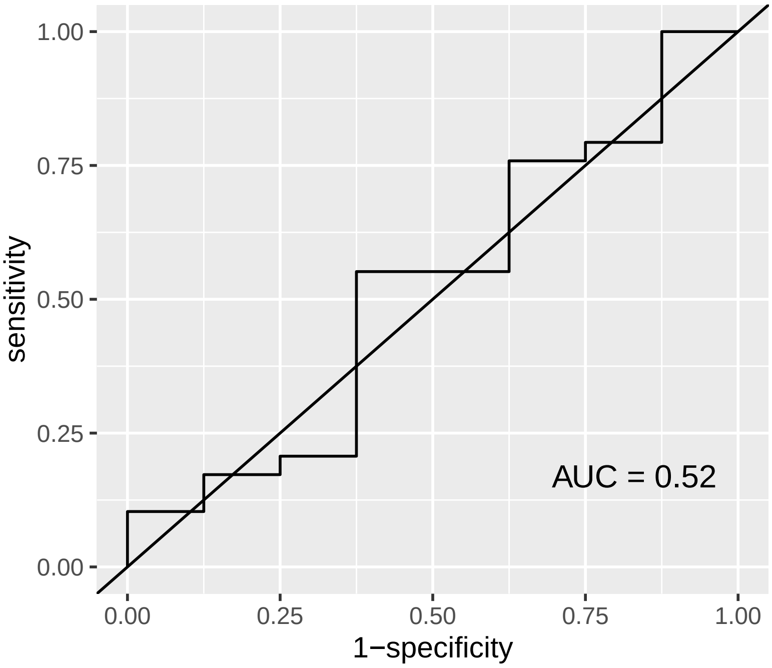

glrlm_LongRunHighGrayLevelEmphasis

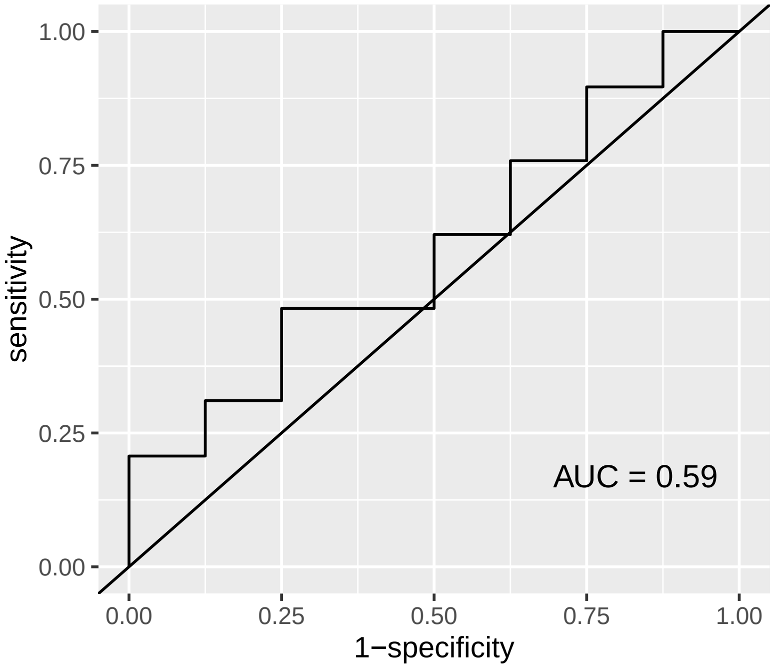

glrlm_RunEntropy

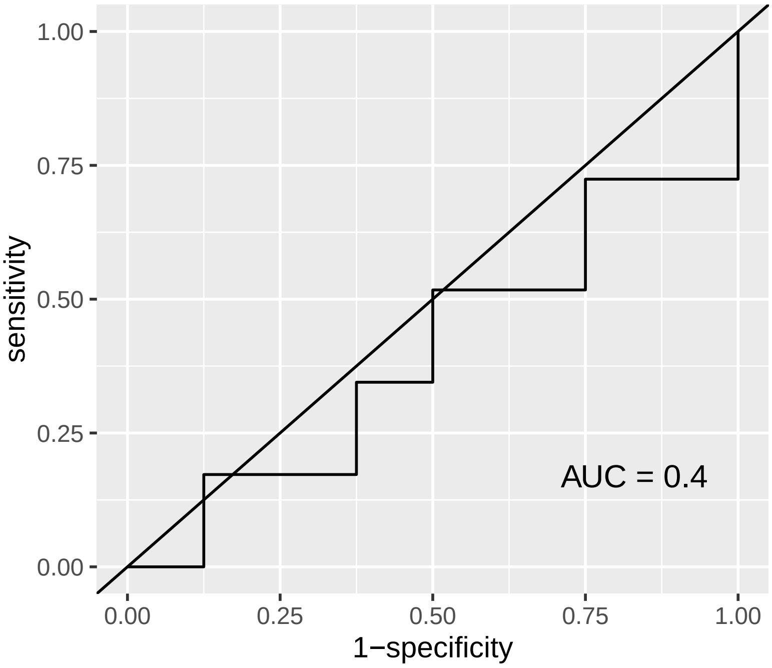

glrlm_LongRunLowGrayLevelEmphasis

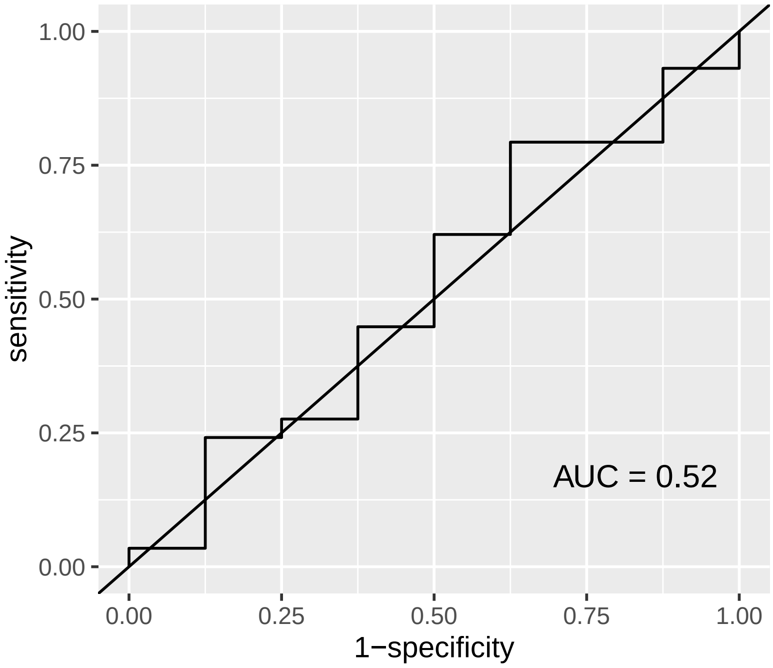

glrlm_RunLengthNonUniformity

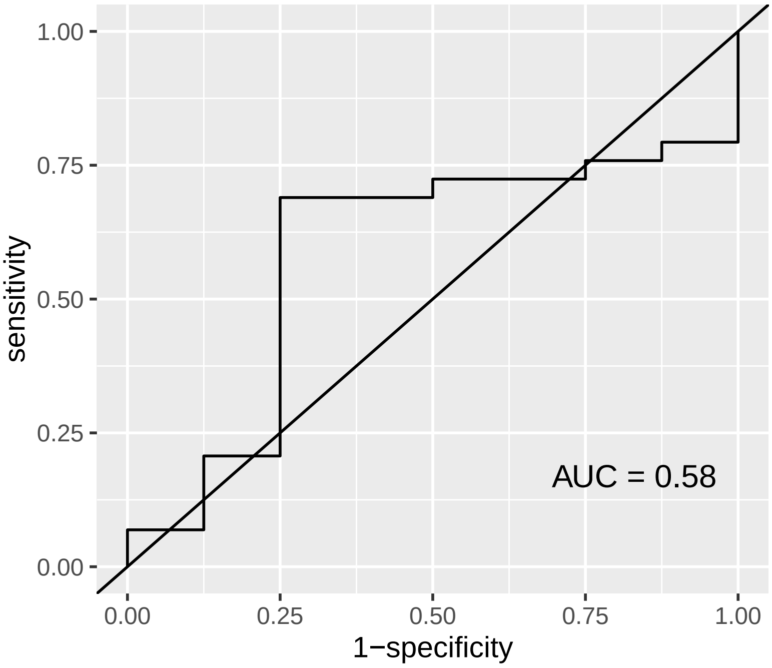

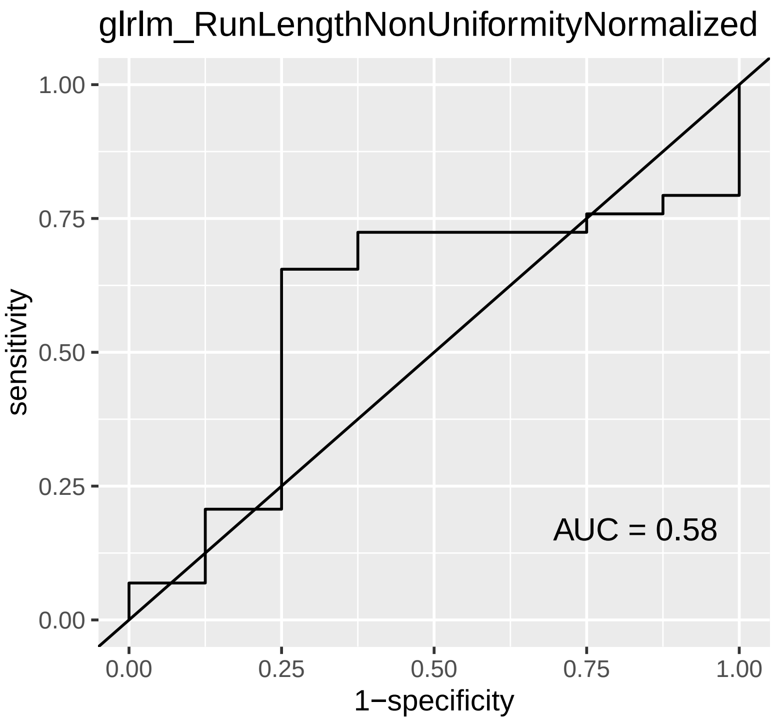
glrlm_RunLengthNonUniformityNormalized
sensitivity
1−specificity
AUC = 0.58
0.00
0.25
0.50
0.75
1.00

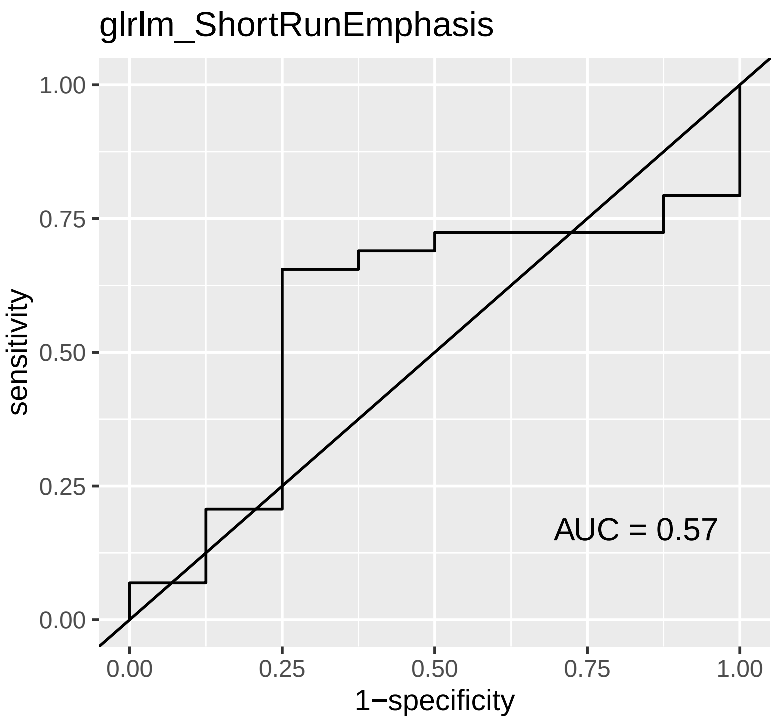
glrlm_ShortRunEmphasis
sensitivity
1−specificity
AUC = 0.57
0.00
0.25
0.50
0.75
1.00

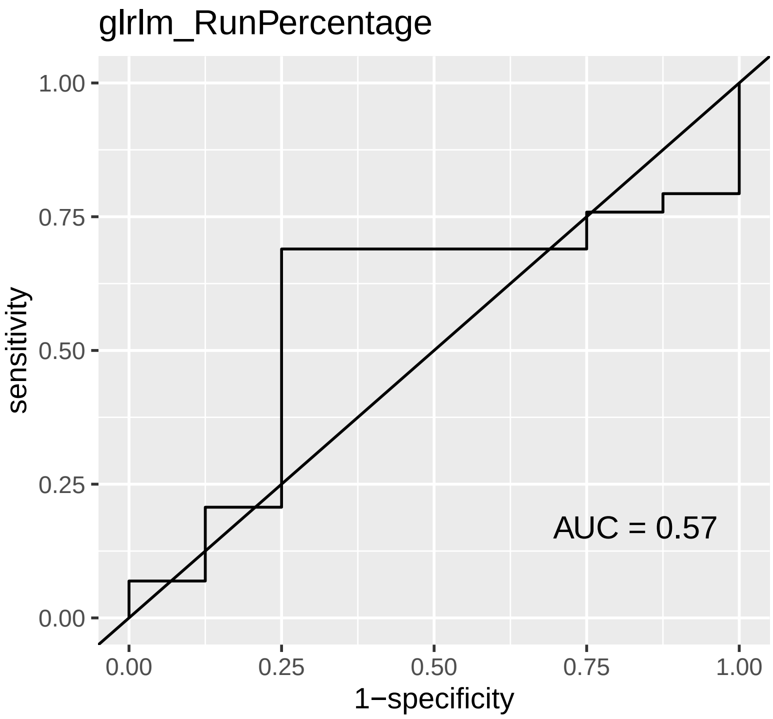
glrlm_RunPercentage
sensitivity
1−specificity
AUC = 0.57
0.00
0.25
0.50
0.75
1.00

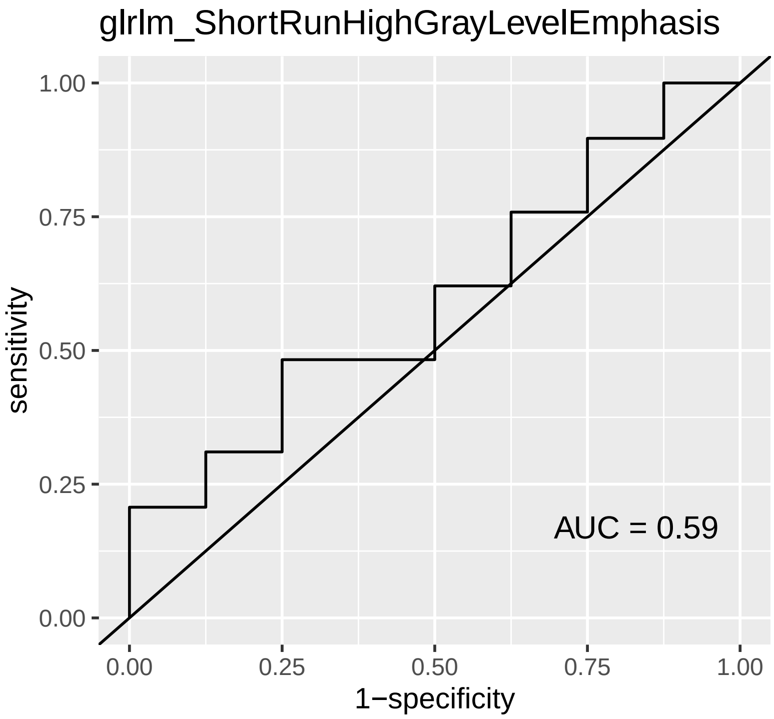
glrlm_ShortRunHighGrayLevelEmphasis
sensitivity
1−specificity
AUC = 0.59
0.00
0.25
0.50
0.75
1.00

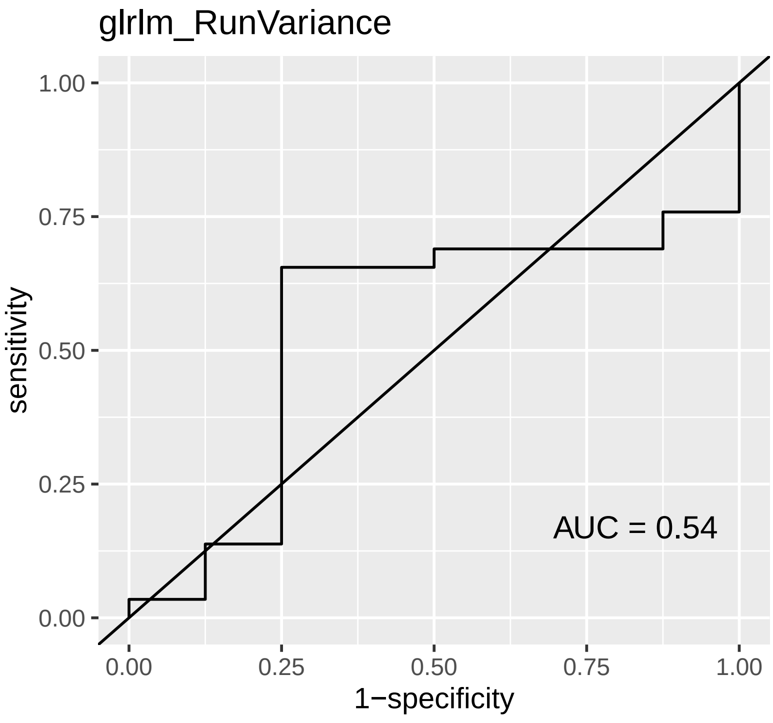
glrlm_RunVariance
sensitivity
1−specificity
AUC = 0.54
0.00
0.25
0.50
0.75
1.00

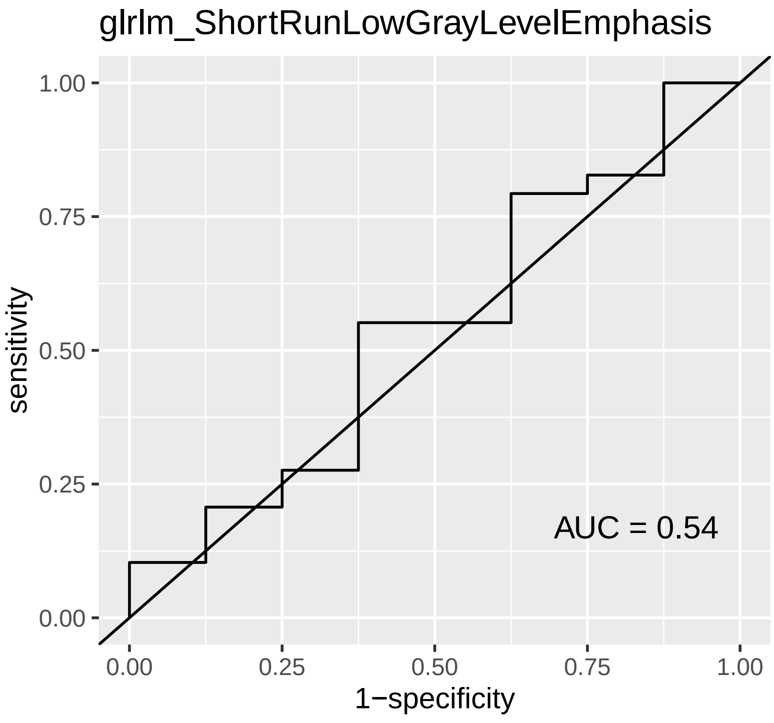
glrlm_ShortRunLowGrayLevelEmphasis
sensitivity
1−specificity
AUC = 0.54
0.00
0.25
0.50
0.75
1.00

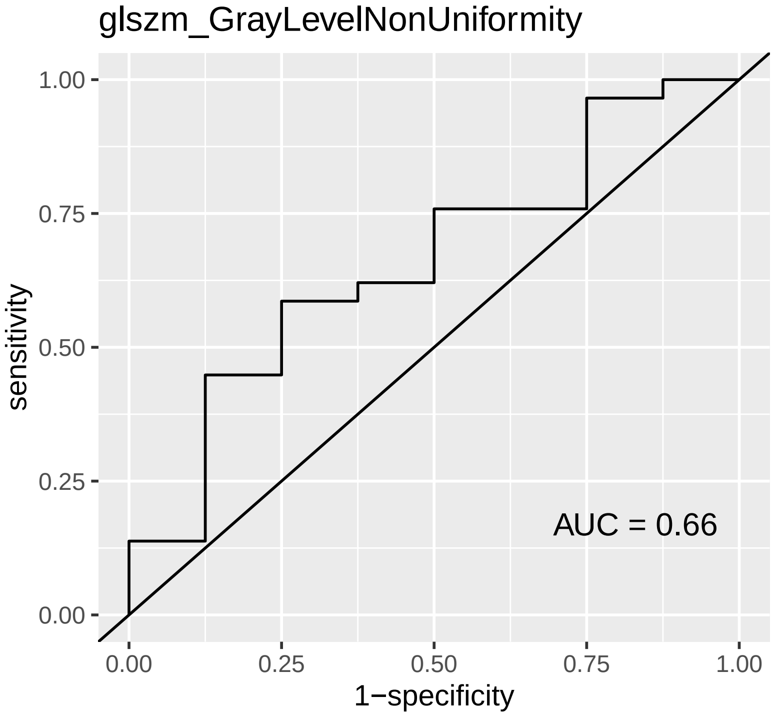
glszm_GrayLevelNonUniformity
sensitivity
1−specificity
AUC = 0.66

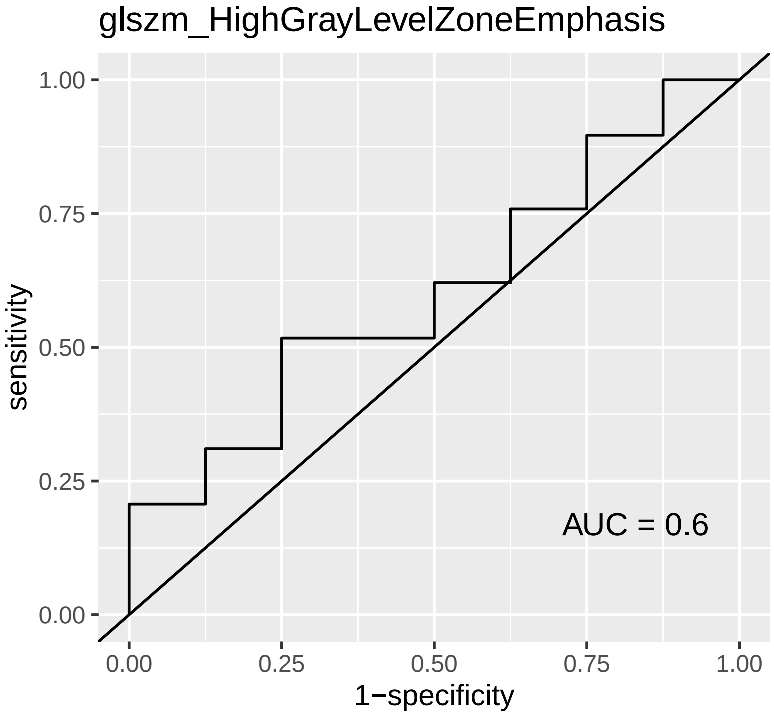
glszm_HighGrayLevelZoneEmphasis
sensitivity
1−specificity
AUC = 0.6

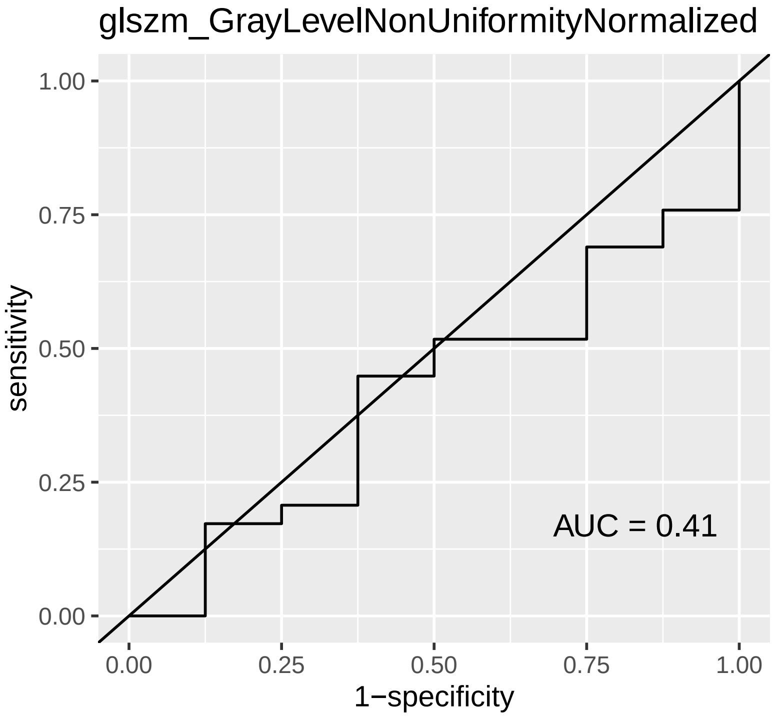
glszm_GrayLevelNonUniformityNormalized
sensitivity
1−specificity
AUC = 0.41

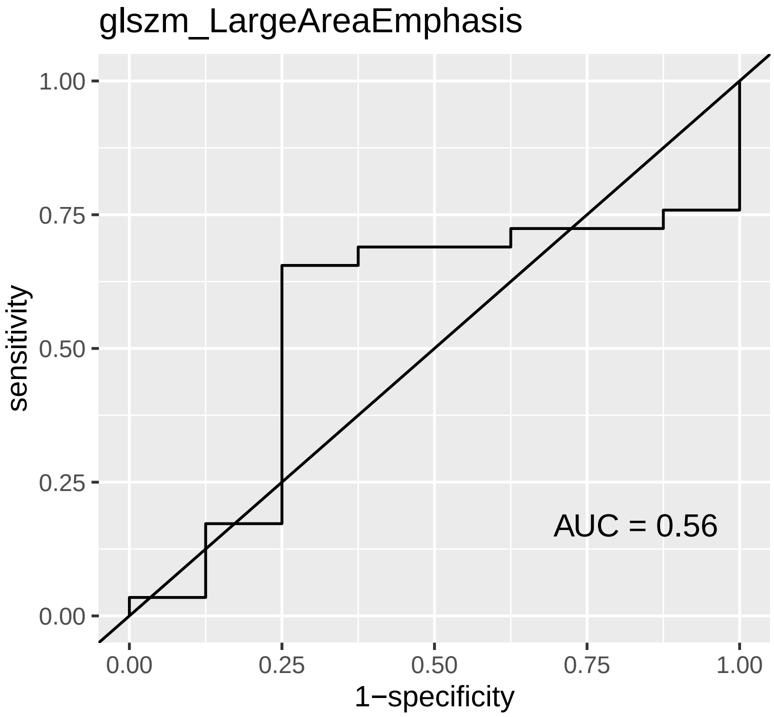
glszm_LargeAreaEmphasis
sensitivity
1−specificity
AUC = 0.56

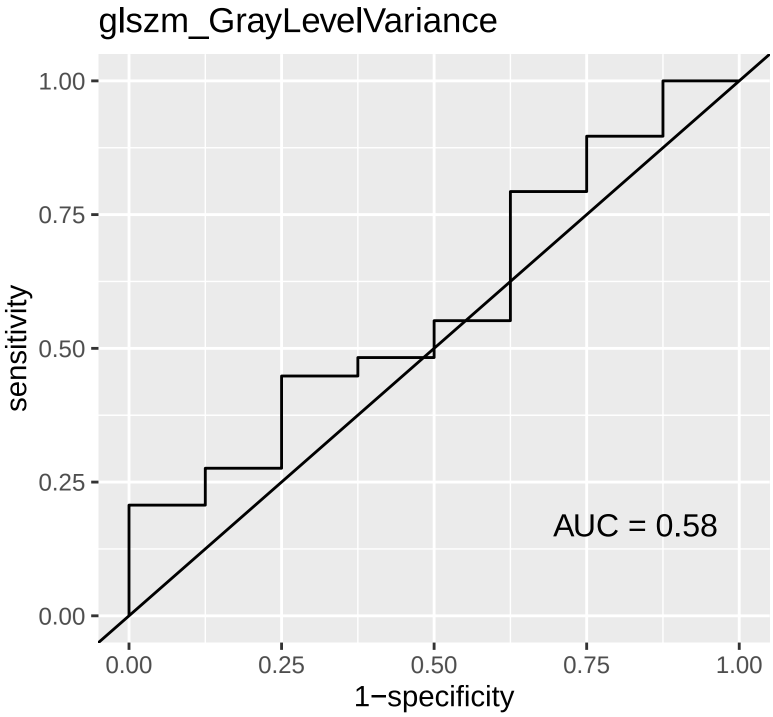
glszm_GrayLevelVariance
sensitivity
1−specificity
AUC = 0.58

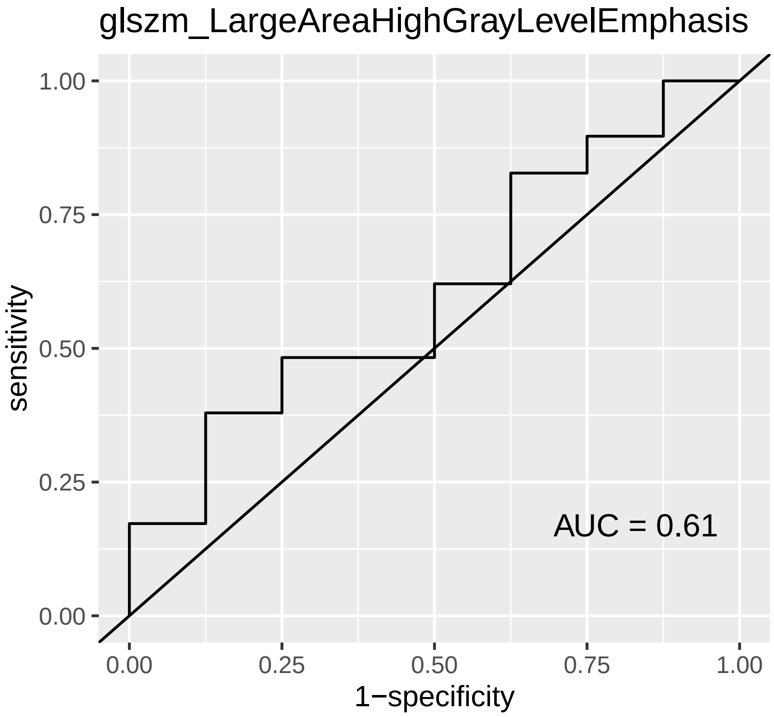
glszm_LargeAreaHighGrayLevelEmphasis
sensitivity
1−specificity
AUC = 0.61

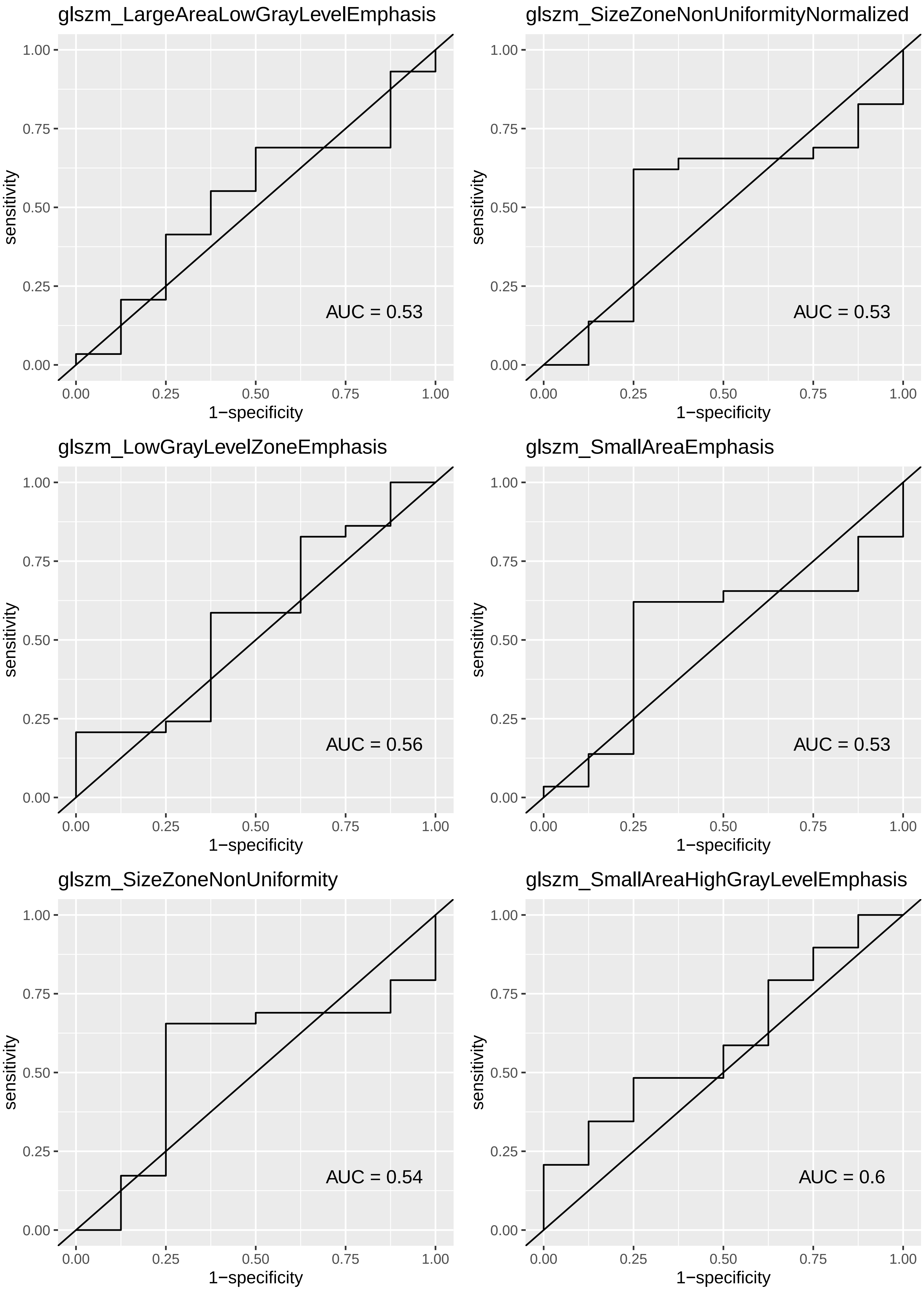
glszm_LargeAreaLowGrayLevelEmphasis
AUC = 0.53
glszm_SizeZoneNonUniformityNormalized
AUC = 0.53
glszm_LowGrayLevelZoneEmphasis
AUC = 0.56
glszm_SmallAreaEmphasis
AUC = 0.53
glszm_SizeZoneNonUniformity
AUC = 0.54
glszm_SmallAreaHighGrayLevelEmphasis
AUC = 0.6
sensitivity
1−specificity
0.00
0.25
0.50
0.75
1.00

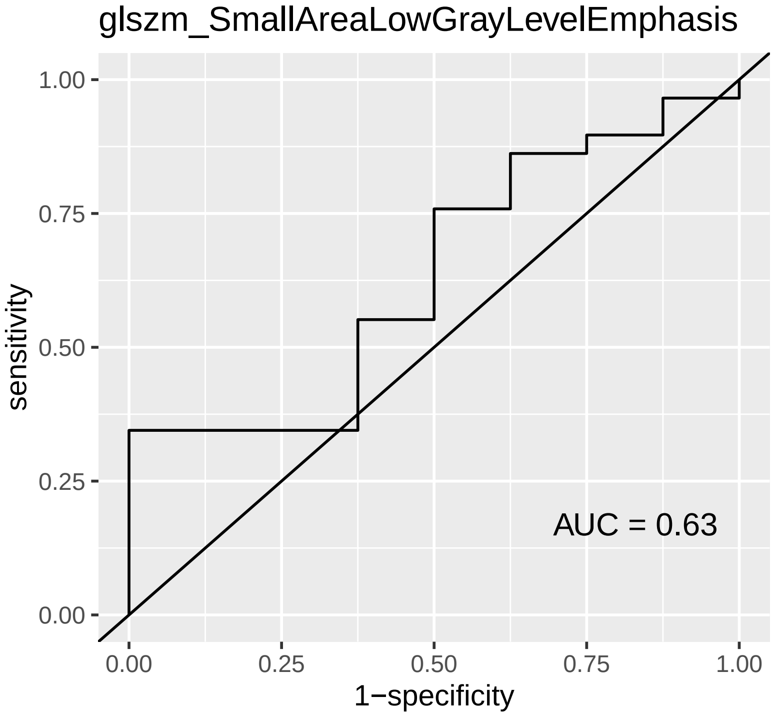
glszm_SmallAreaLowGrayLevelEmphasis
sensitivity
1−specificity
AUC = 0.63

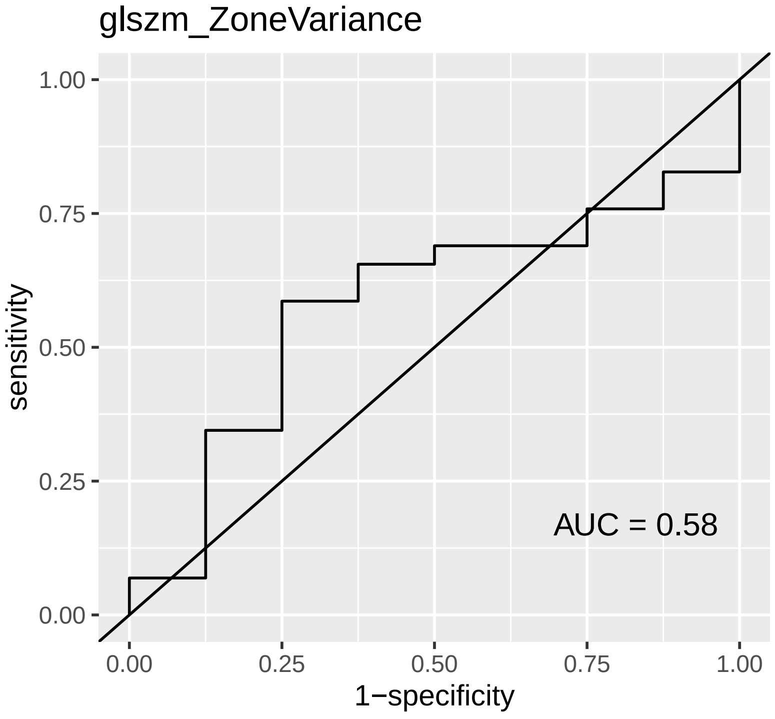
glszm_ZoneVariance
sensitivity
1−specificity
AUC = 0.58

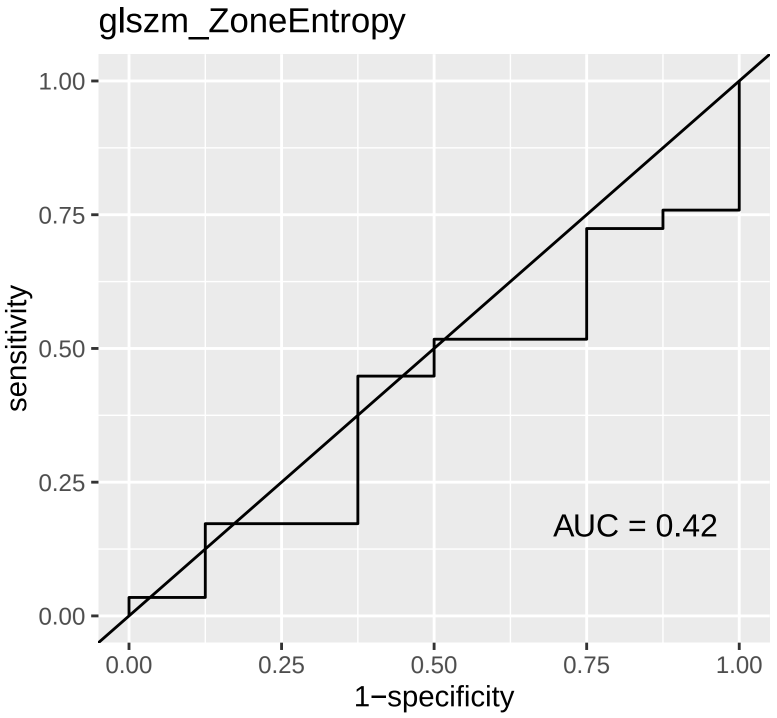
glszm_ZoneEntropy
sensitivity
1−specificity
AUC = 0.42

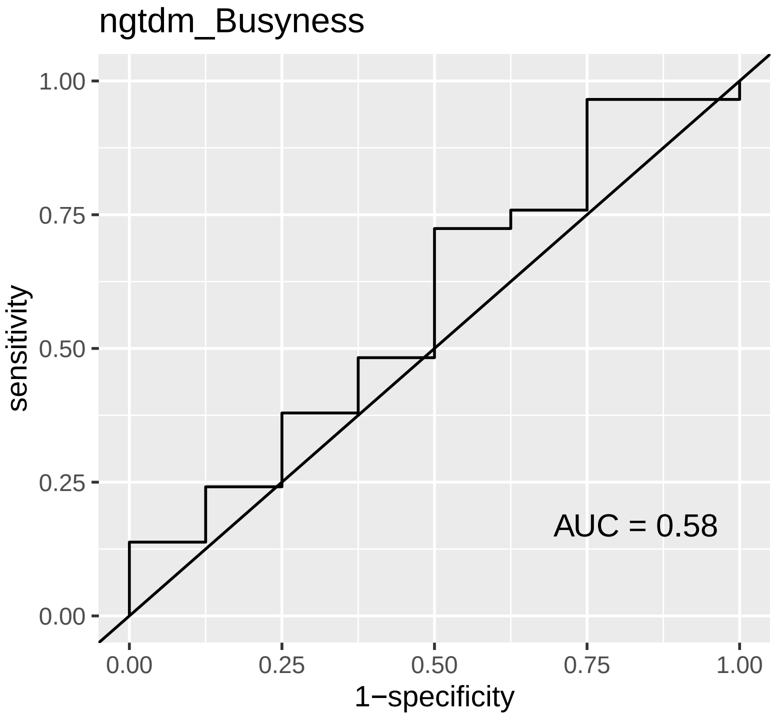
ngtdm_Busyness
sensitivity
1−specificity
AUC = 0.58

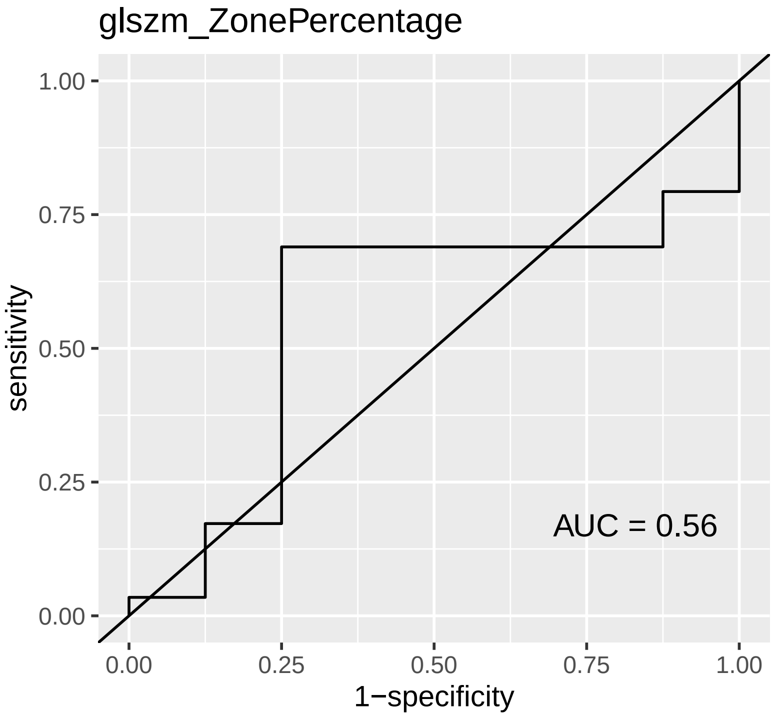
glszm_ZonePercentage
sensitivity
1−specificity
AUC = 0.56

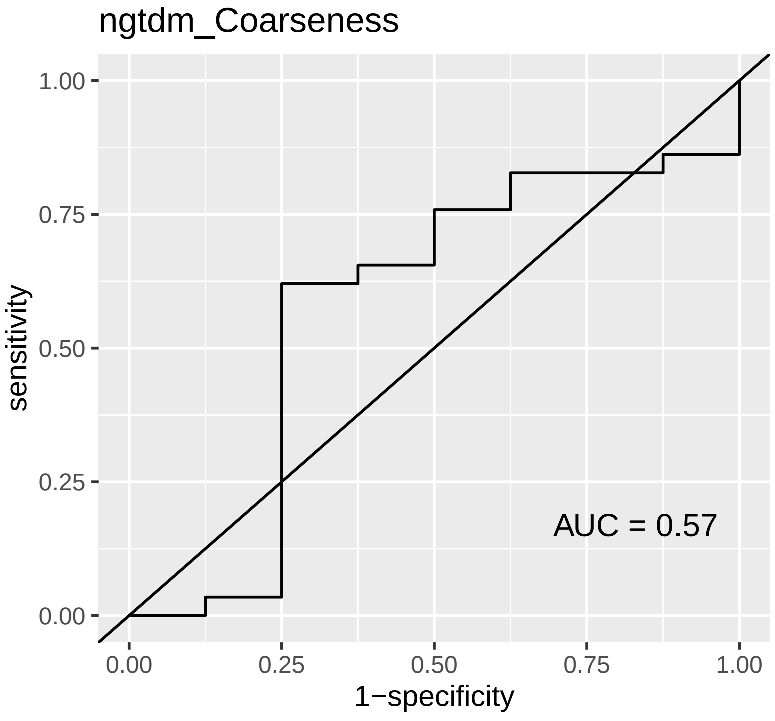
ngtdm_Coarseness
sensitivity
1−specificity
AUC = 0.57

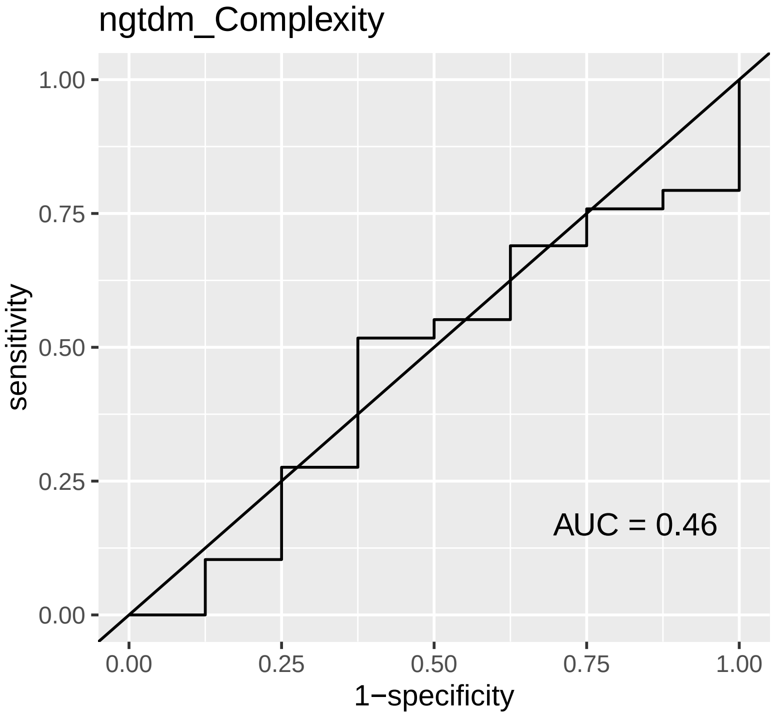
ngtdm_Complexity
sensitivity
1−specificity
AUC = 0.46
0.00
0.25
0.50
0.75
1.00

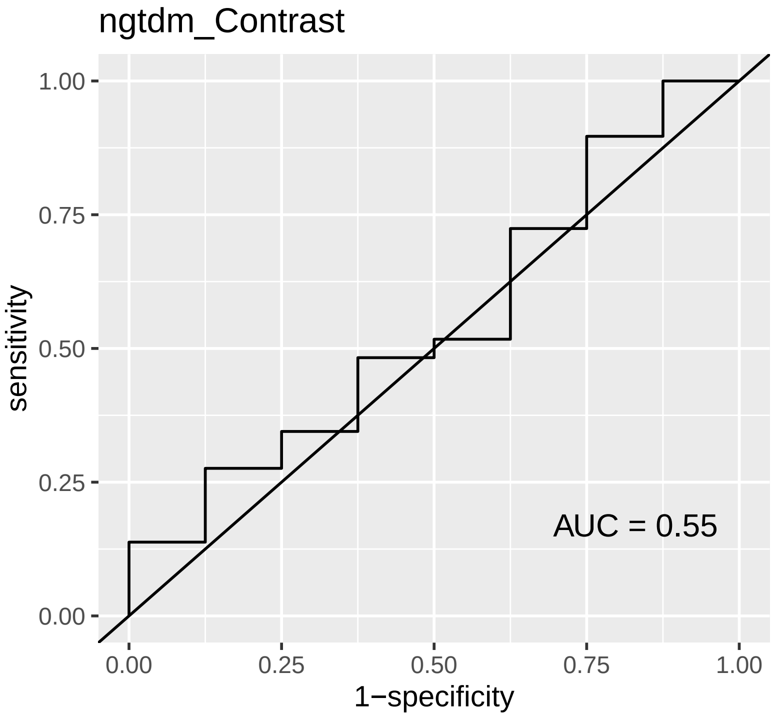
ngtdm_Contrast
sensitivity
1−specificity
AUC = 0.55
0.00
0.25
0.50
0.75
1.00

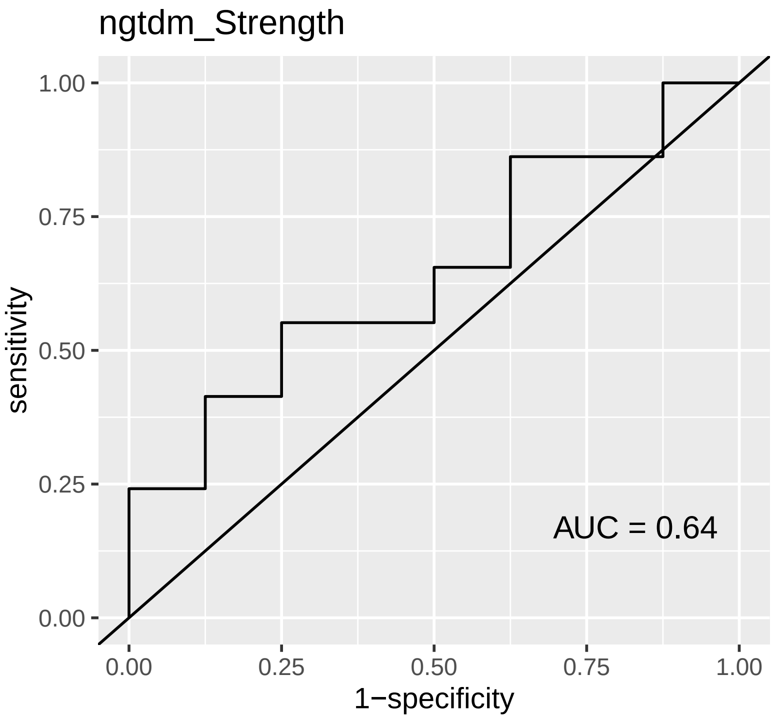
ngtdm_Strength
sensitivity
1−specificity
AUC = 0.64
0.00
0.25
0.50
0.75
1.00
