# Supplementary material for: Detecting Clinically Significant Prostate Cancer in PI-RADS 3 Lesions Using T2w-Derived Radiomics Feature Maps in 3T Prostate MRI
Source: Curr Oncol. 2024 Nov 1;31(11):6814–28. doi: 10.3390/curroncol31110503 (PMC11592716; doi:10.3390/curroncol31110503)

firstorder_10Percentile

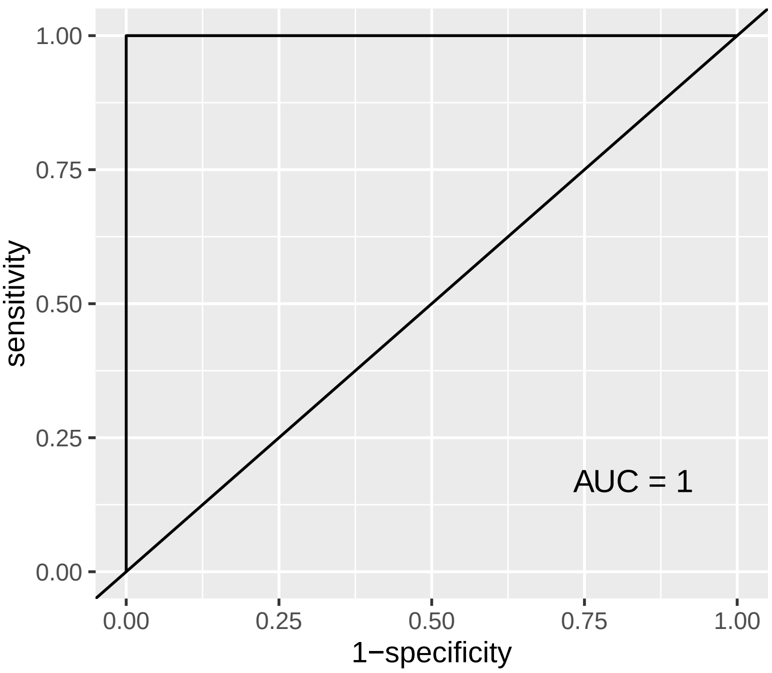

firstorder_Entropy

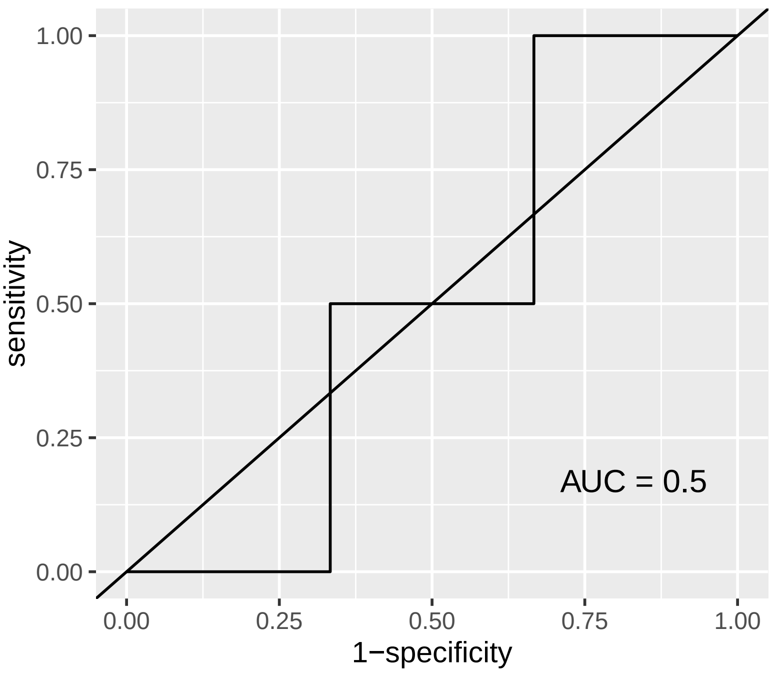

firstorder_90Percentile

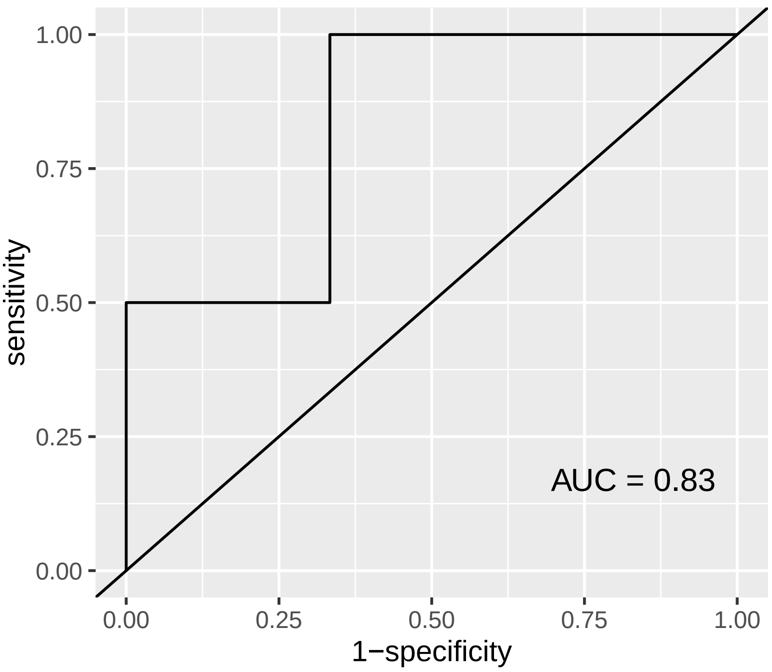

firstorder_InterquartileRange

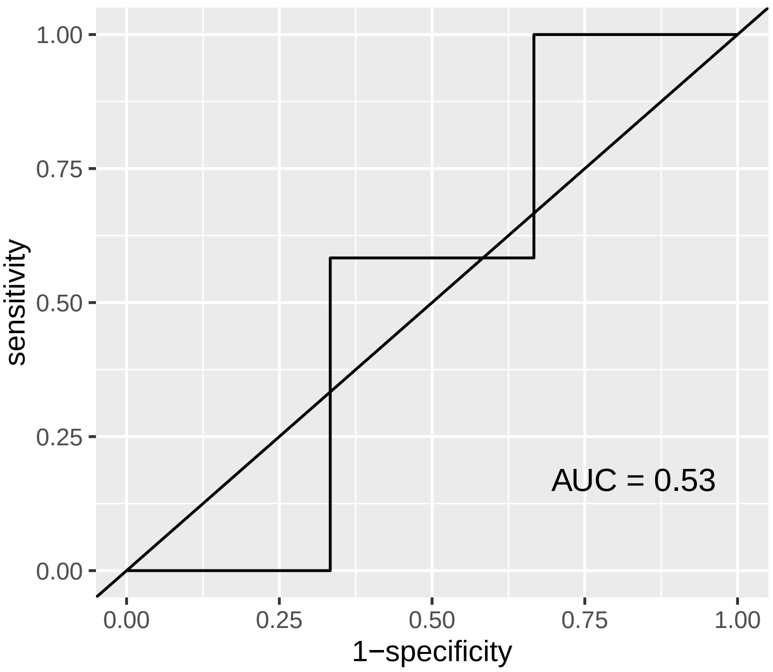

firstorder_Energy

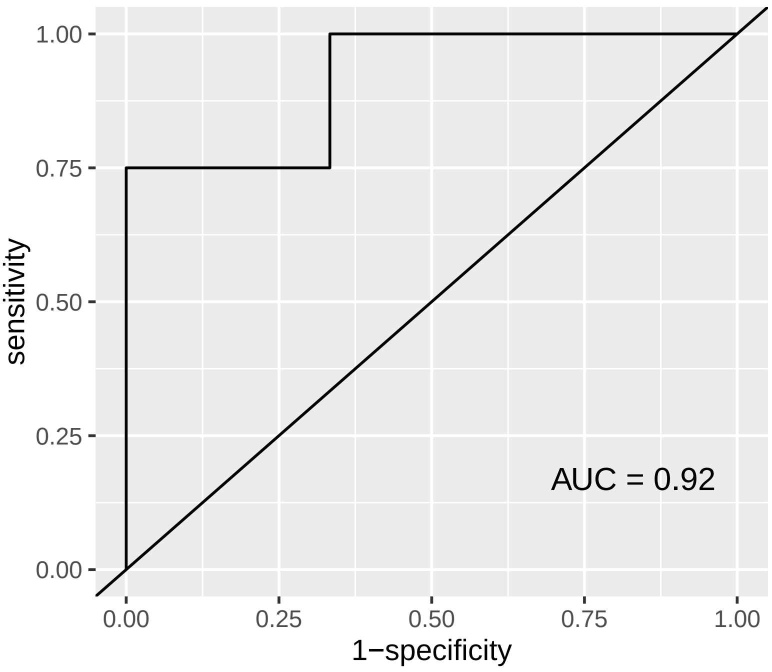

firstorder_Kurtosis

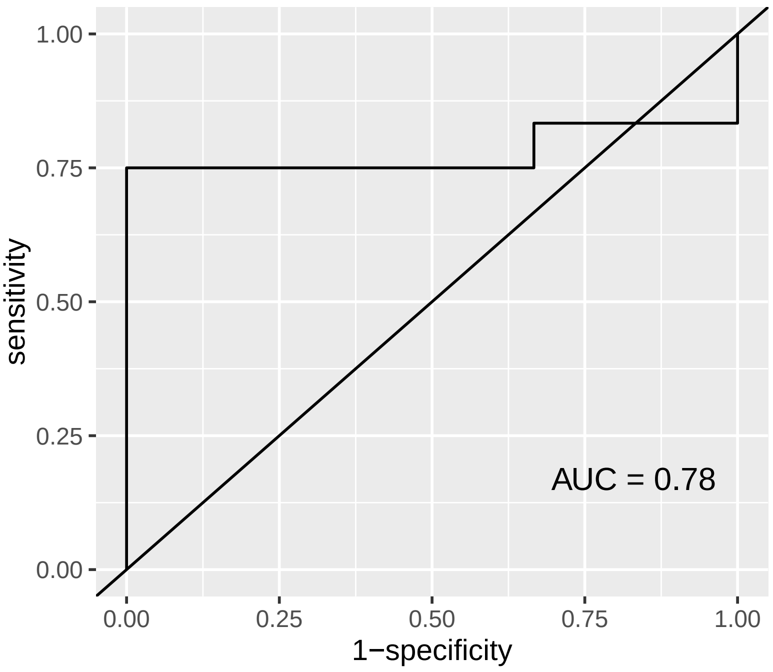

firstorder_Maximum

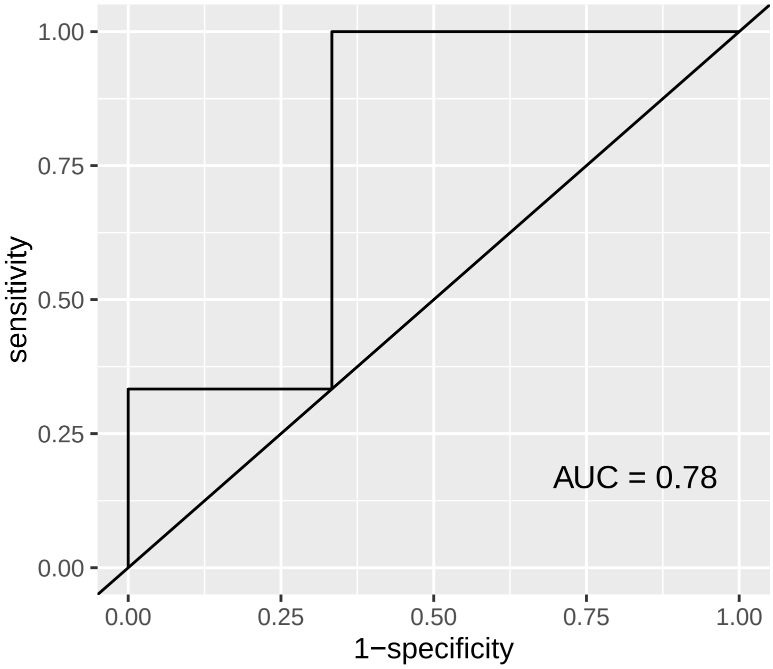

firstorder_Median

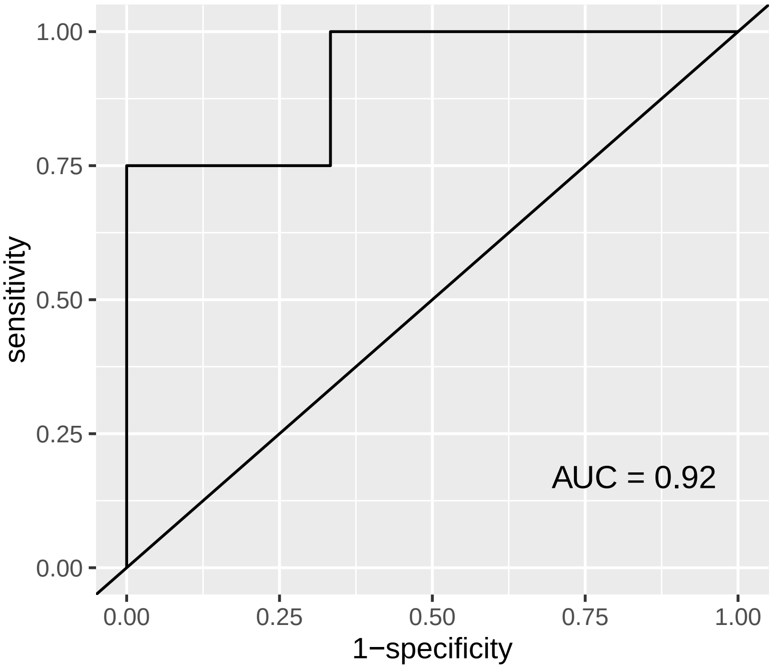

firstorder_Mean

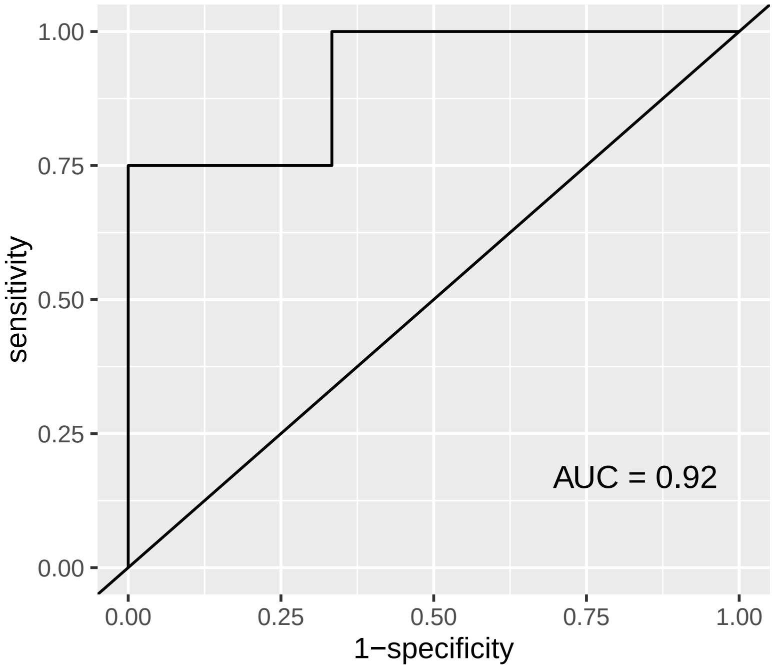

firstorder_Minimum

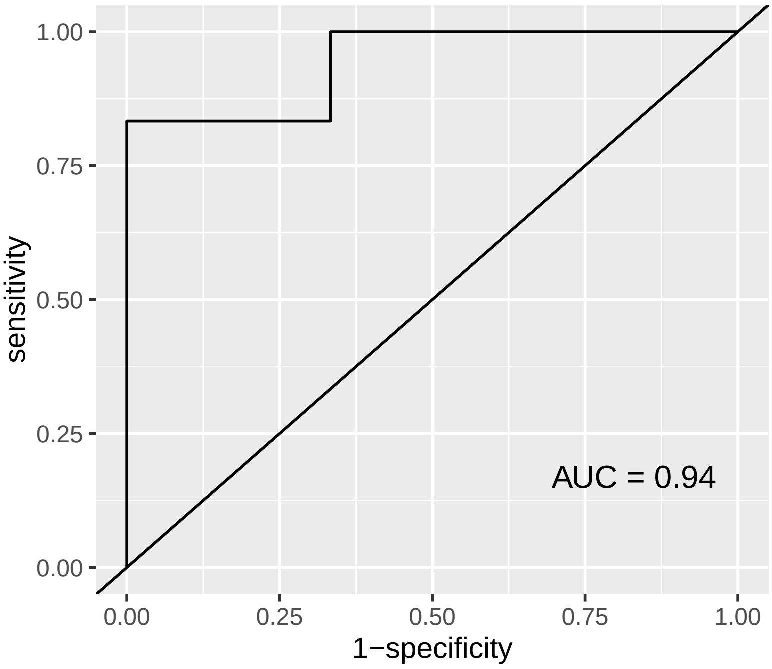

firstorder_MeanAbsoluteDeviation

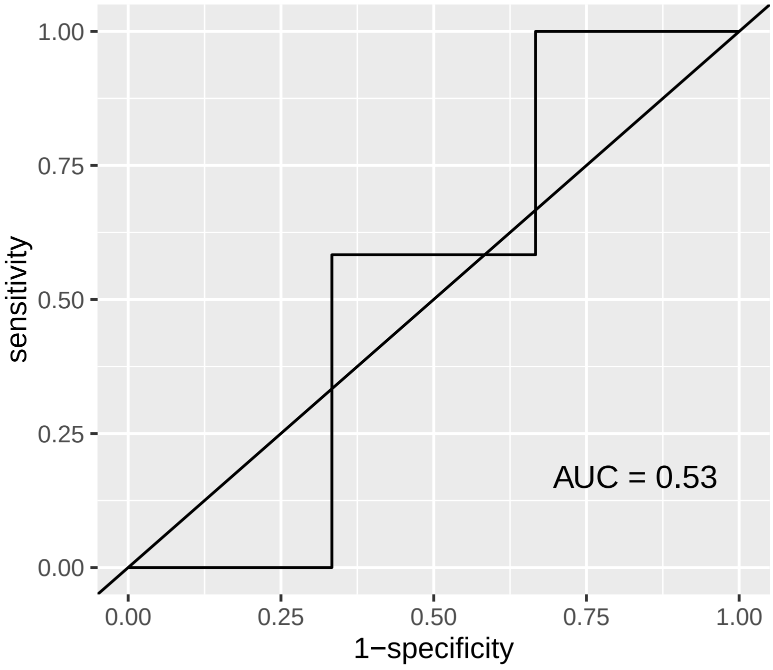

firstorder_Range

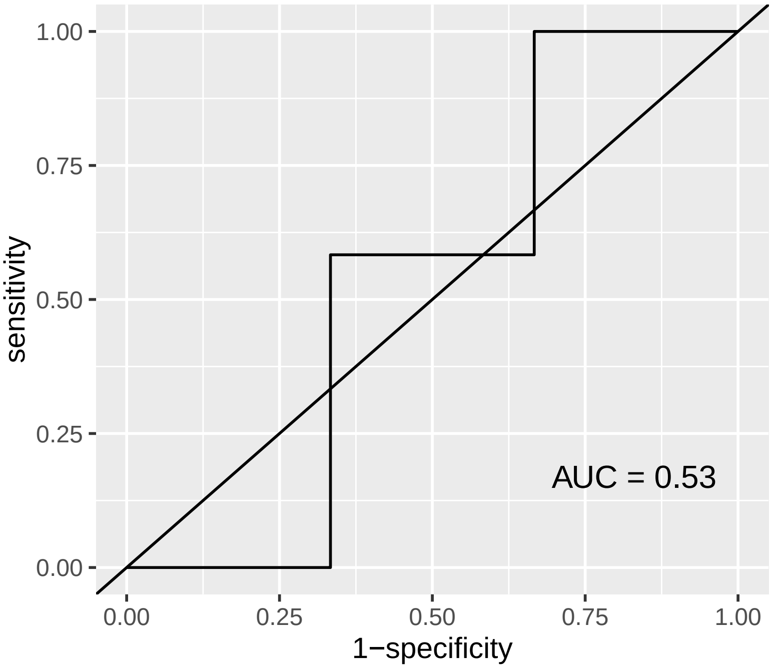

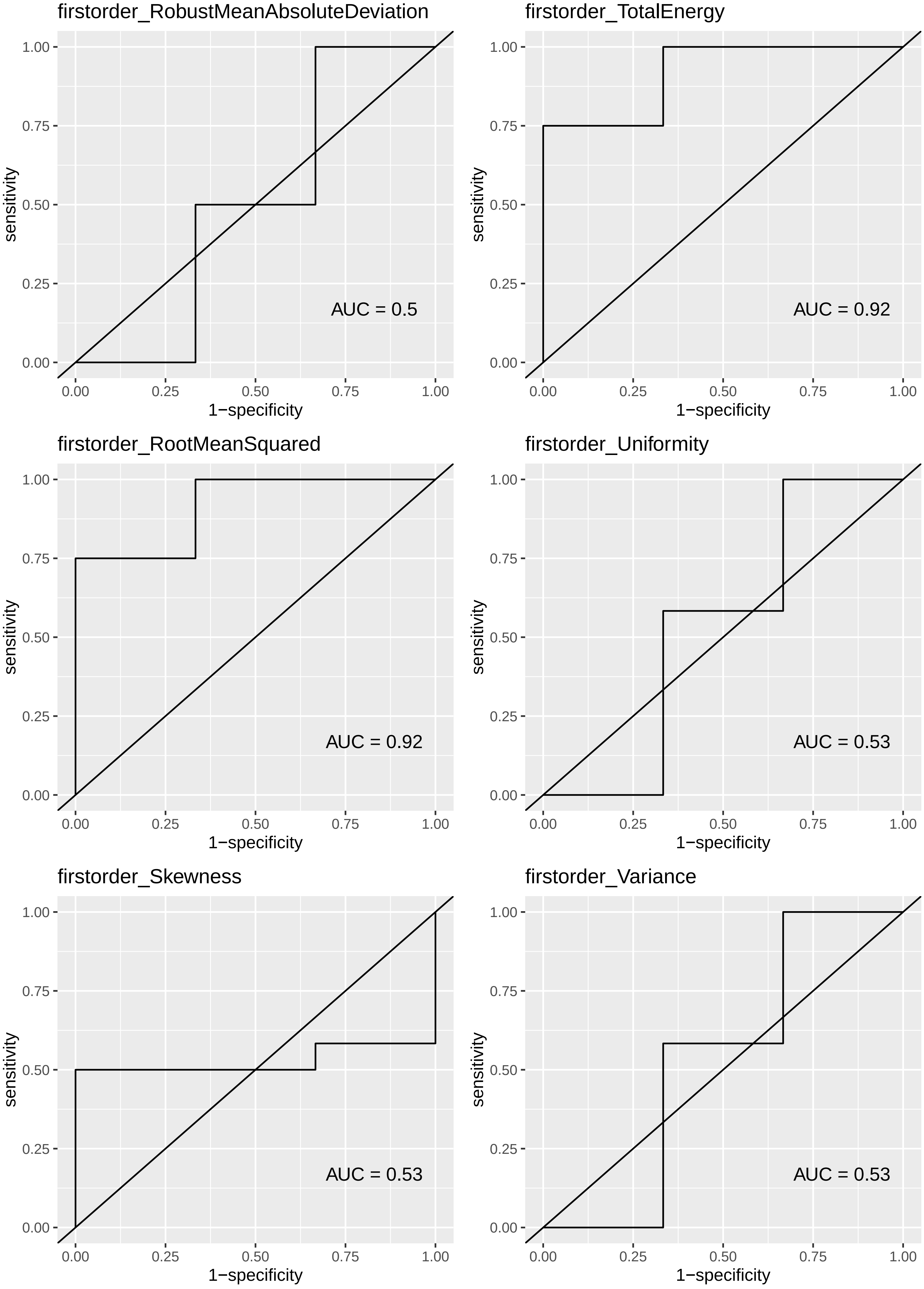
firstorder_RobustMeanAbsoluteDeviation
sensitivity
1−specificity
AUC = 0.5
firstorder_TotalEnergy
sensitivity
1−specificity
AUC = 0.92
firstorder_RootMeanSquared
sensitivity
1−specificity
AUC = 0.92
firstorder_Uniformity
sensitivity
1−specificity
AUC = 0.53
firstorder_Skewness
sensitivity
1−specificity
AUC = 0.53
firstorder_Variance
sensitivity
1−specificity
AUC = 0.53
0.00
0.25
0.50
0.75
1.00

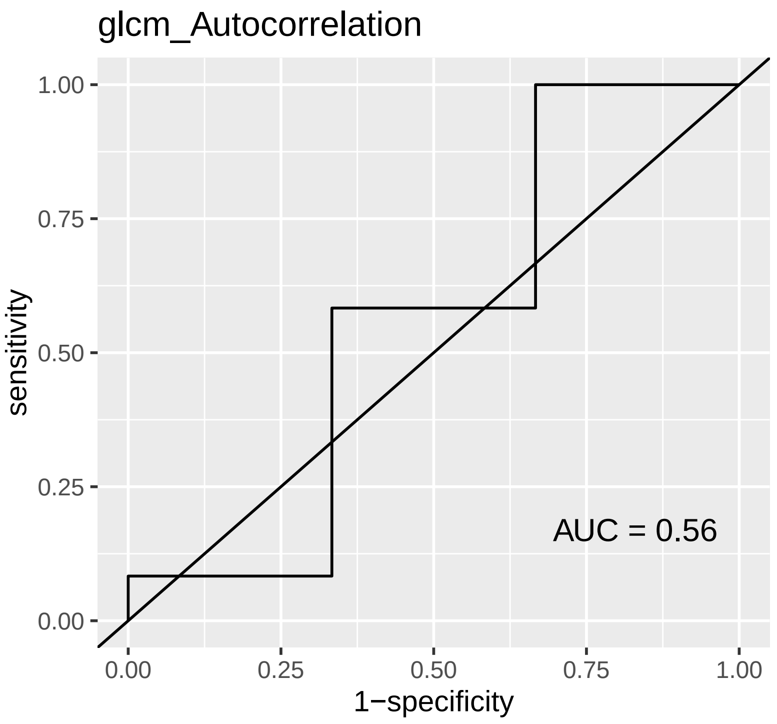
glcm_Autocorrelation
sensitivity
1−specificity
0.00
0.25
0.50
0.75
1.00
AUC = 0.56

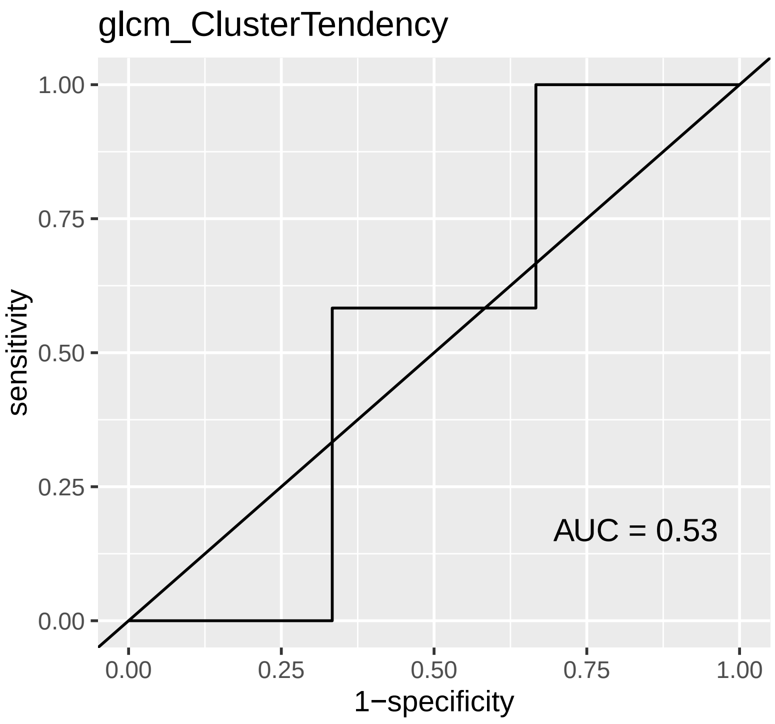
glcm_ClusterTendency
sensitivity
1−specificity
0.00
0.25
0.50
0.75
1.00
AUC = 0.53

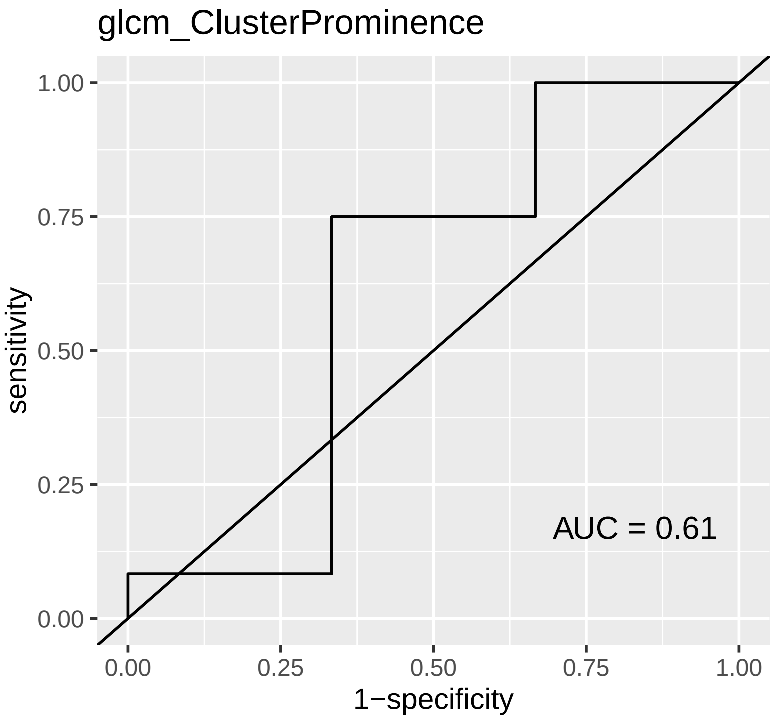
glcm_ClusterProminence
sensitivity
1−specificity
0.00
0.25
0.50
0.75
1.00
AUC = 0.61

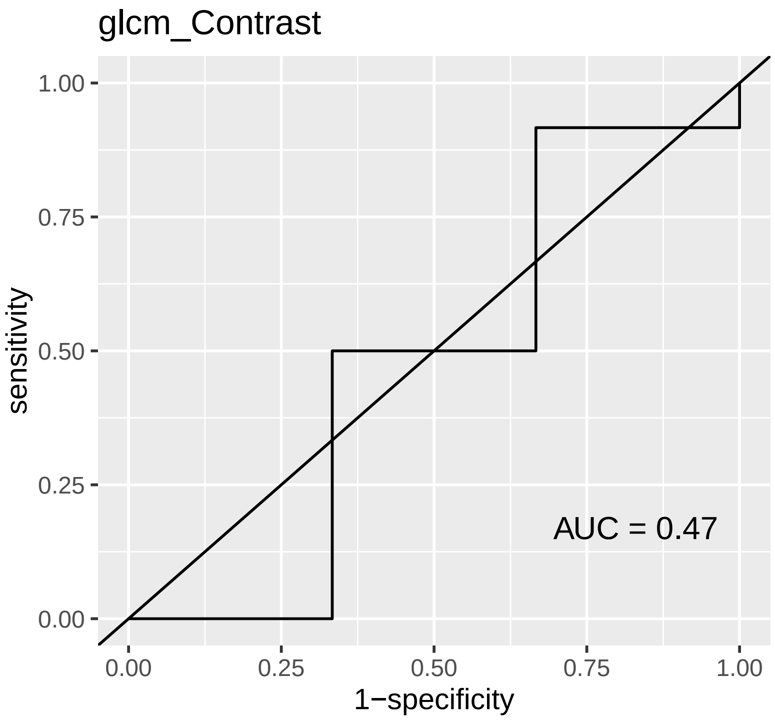
glcm_Contrast
sensitivity
1−specificity
0.00
0.25
0.50
0.75
1.00
AUC = 0.47

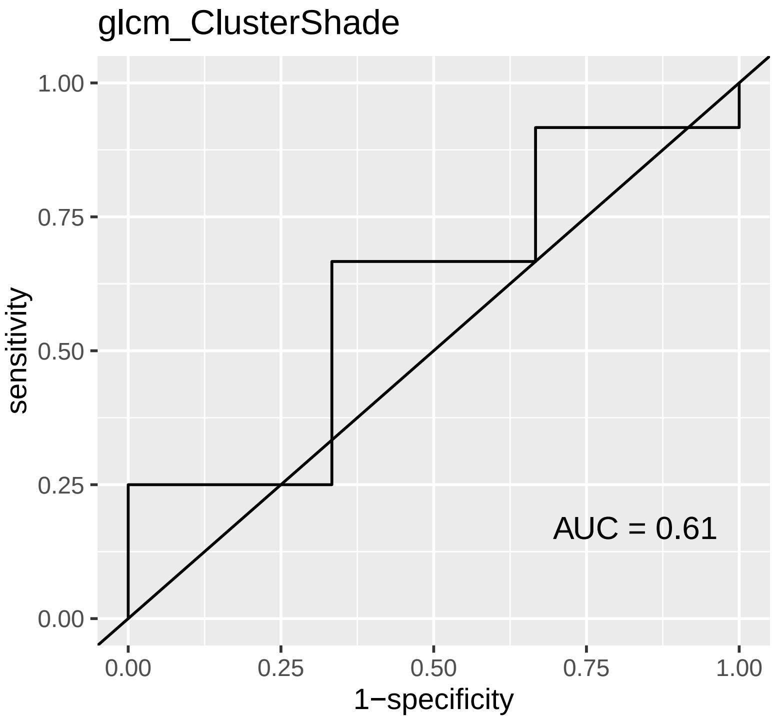
glcm_ClusterShade
sensitivity
1−specificity
0.00
0.25
0.50
0.75
1.00
AUC = 0.61

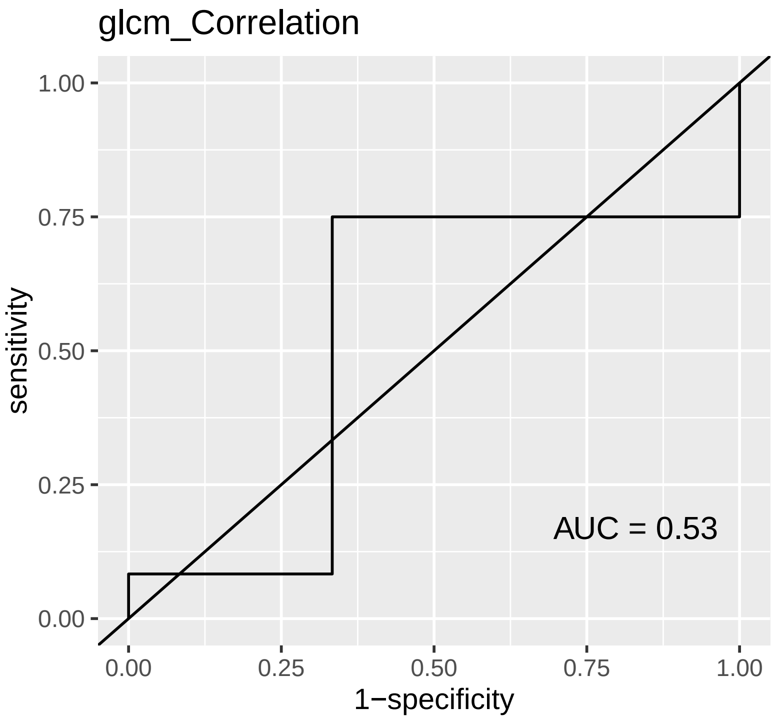
glcm_Correlation
sensitivity
1−specificity
0.00
0.25
0.50
0.75
1.00
AUC = 0.53

glcm_DifferenceAverage

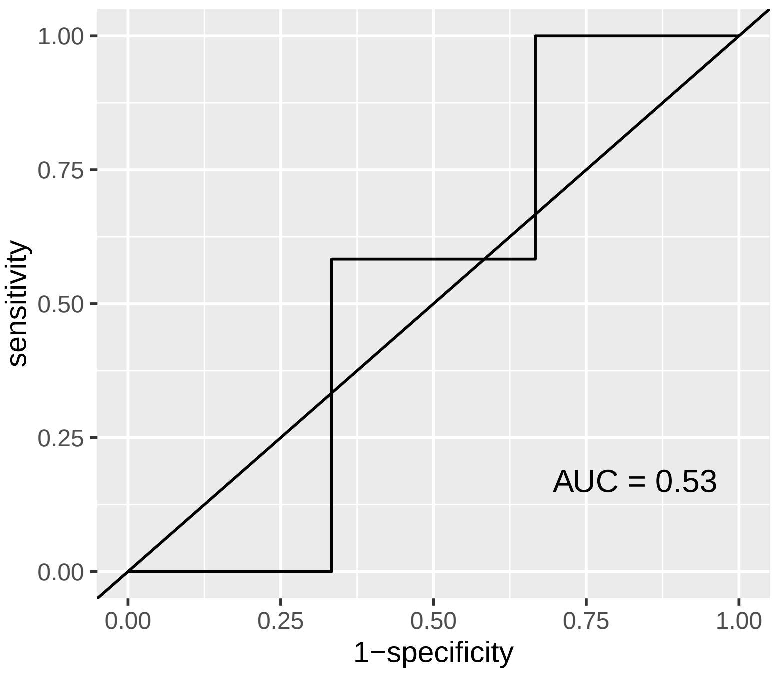

glcm_DifferenceEntropy

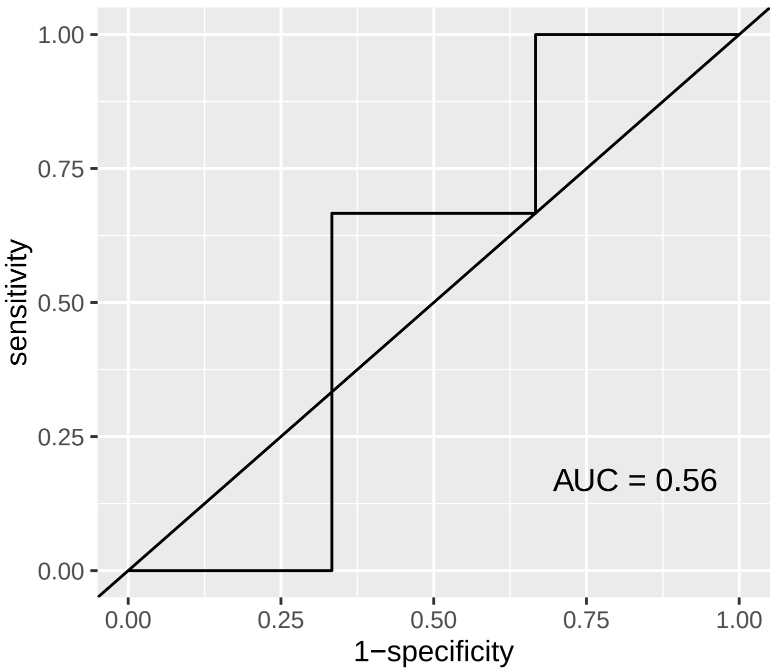

glcm_DifferenceVariance

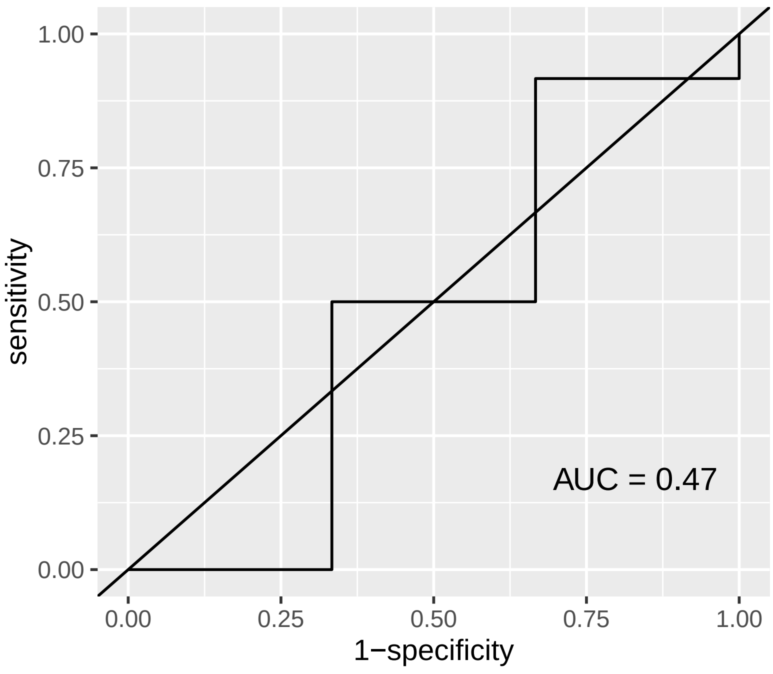

glcm_Id

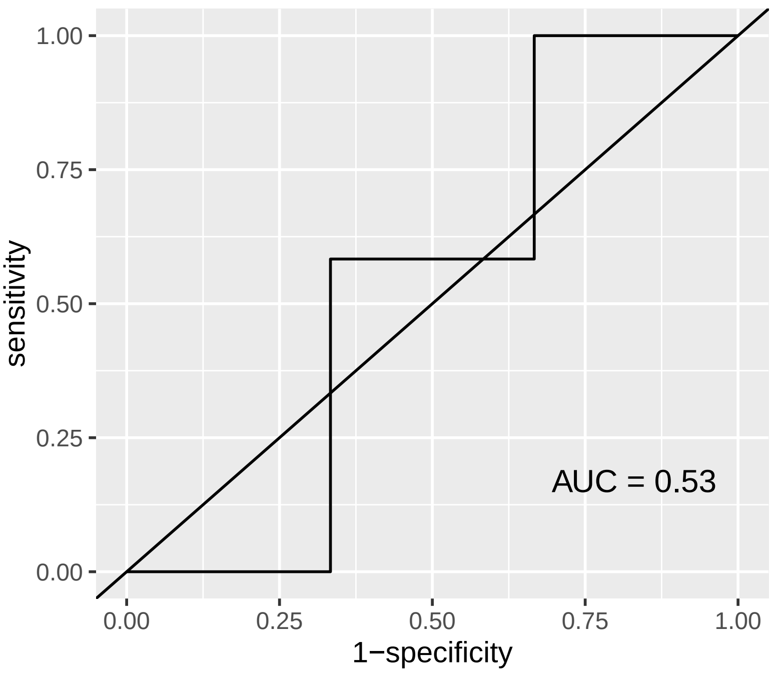

glcm_Idm

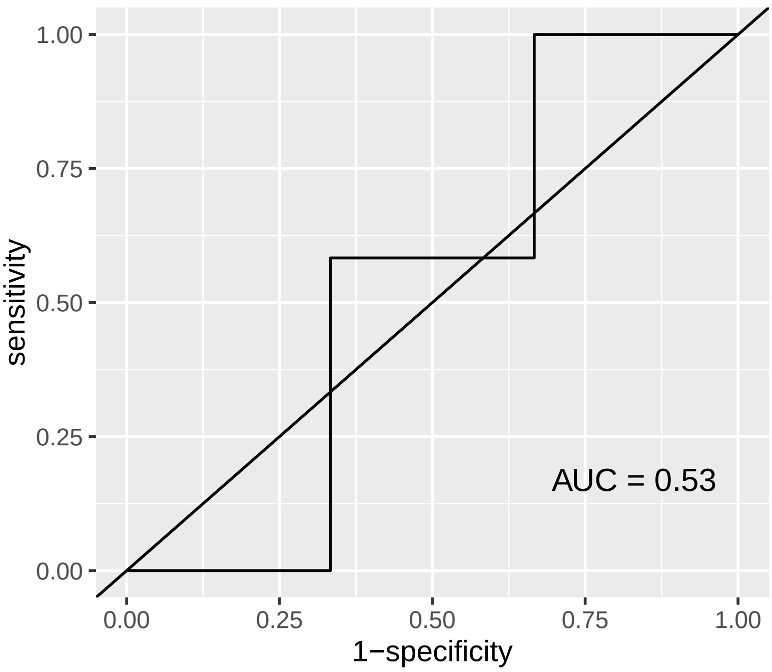

glcm_Idmn

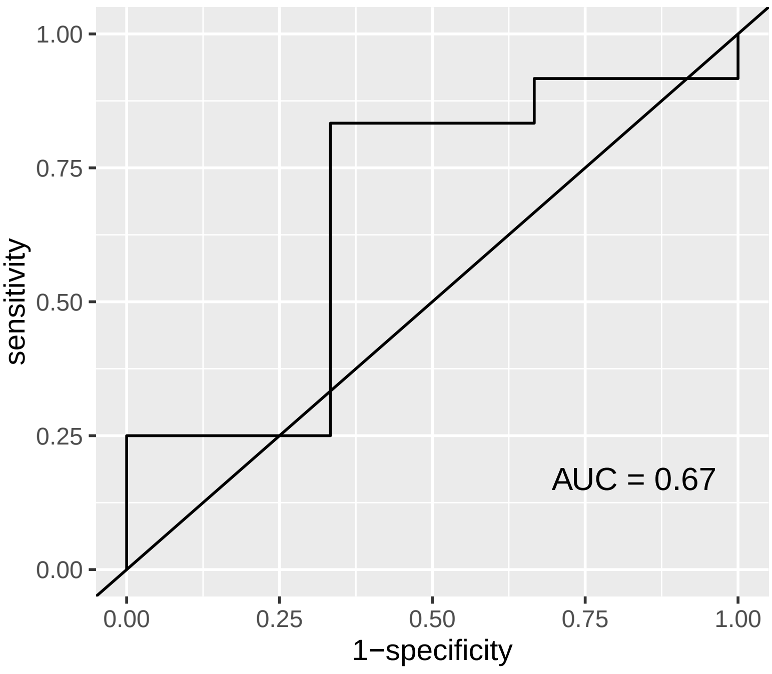

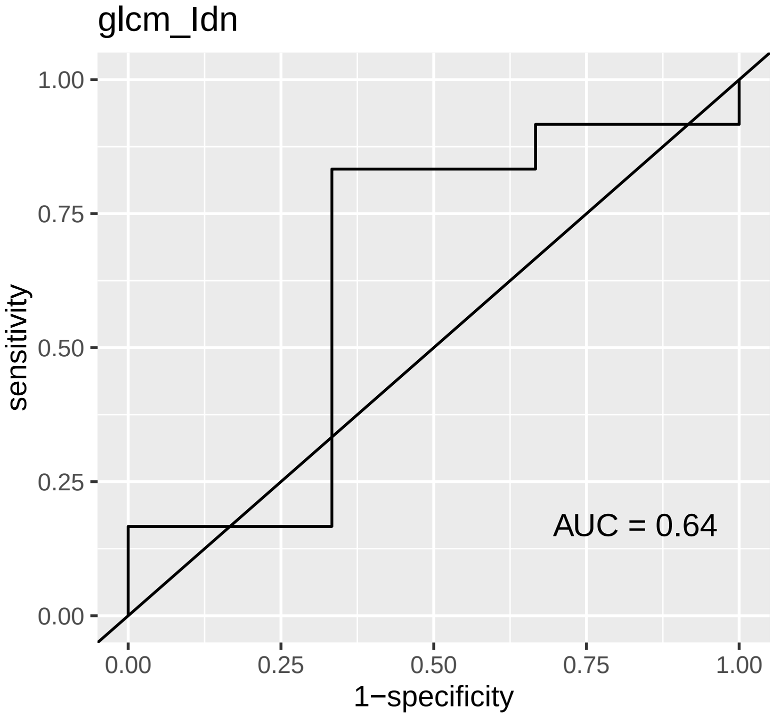
glcm_Idn
AUC = 0.64
sensitivity
0.00
0.25
0.50
0.75
1.00
1−specificity
0.00
0.25
0.50
0.75
1.00

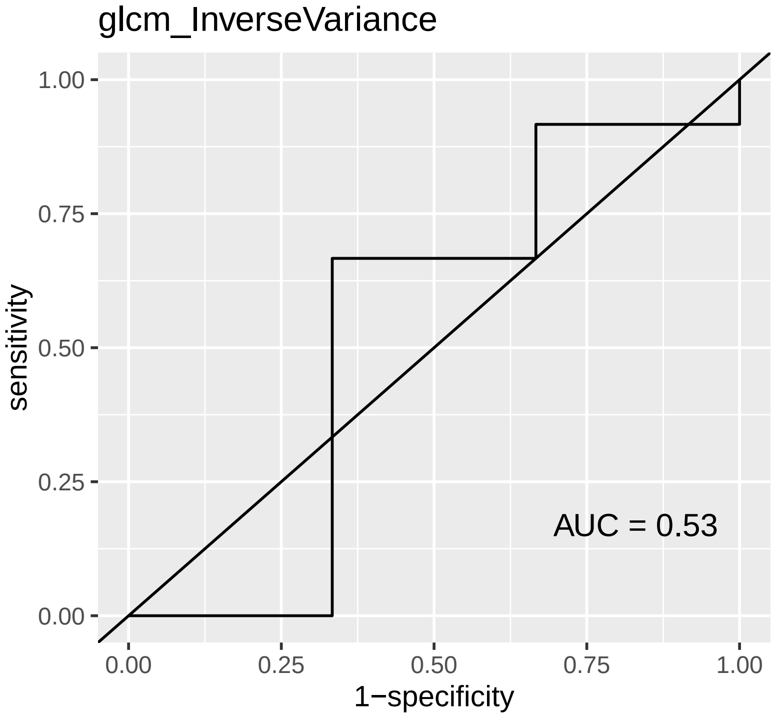
glcm_InverseVariance
AUC = 0.53
sensitivity
0.00
0.25
0.50
0.75
1.00
1−specificity
0.00
0.25
0.50
0.75
1.00

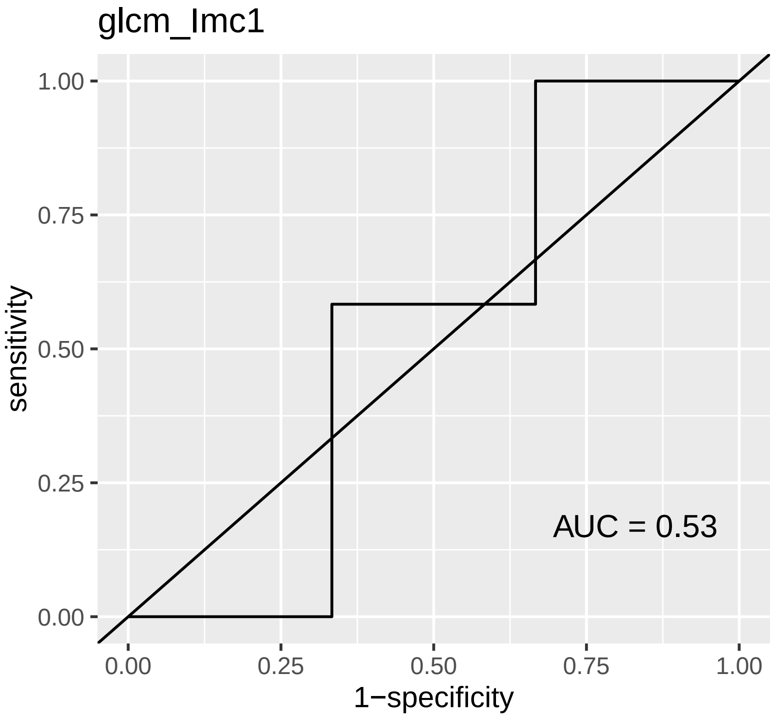
glcm_Imc1
AUC = 0.53
sensitivity
0.00
0.25
0.50
0.75
1.00
1−specificity
0.00
0.25
0.50
0.75
1.00

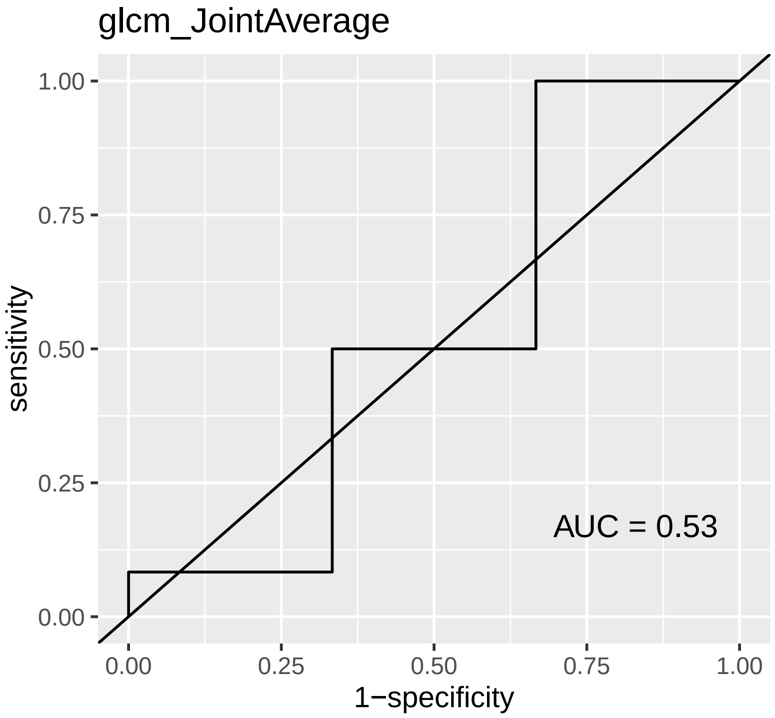
glcm_JointAverage
AUC = 0.53
sensitivity
0.00
0.25
0.50
0.75
1.00
1−specificity
0.00
0.25
0.50
0.75
1.00

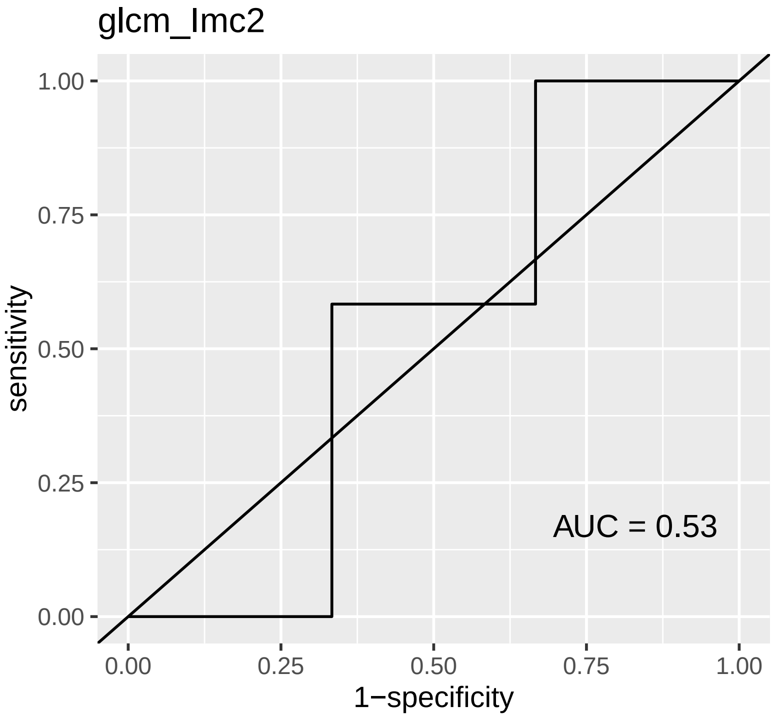
glcm_Imc2
AUC = 0.53
sensitivity
0.00
0.25
0.50
0.75
1.00
1−specificity
0.00
0.25
0.50
0.75
1.00

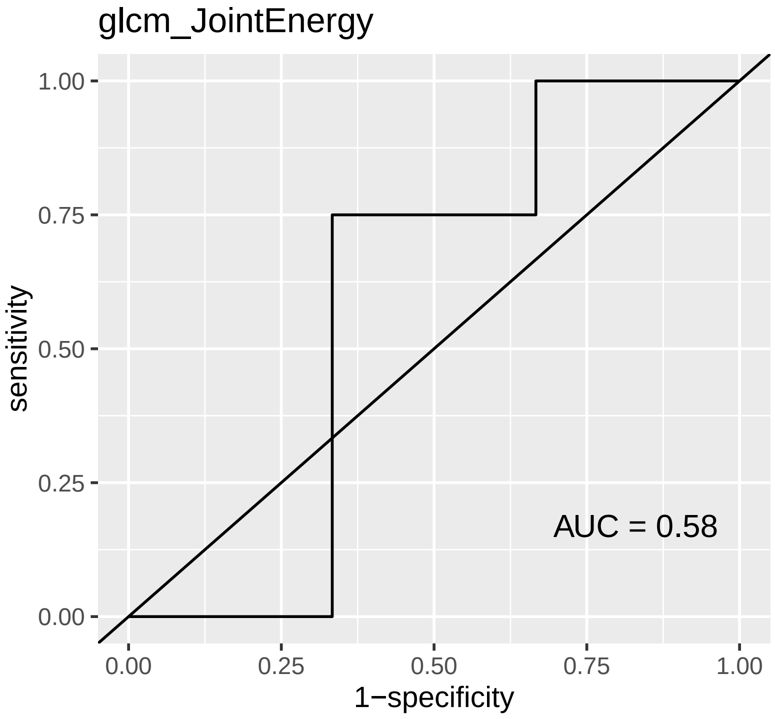
glcm_JointEnergy
AUC = 0.58
sensitivity
0.00
0.25
0.50
0.75
1.00
1−specificity
0.00
0.25
0.50
0.75
1.00

glcm_JointEntropy

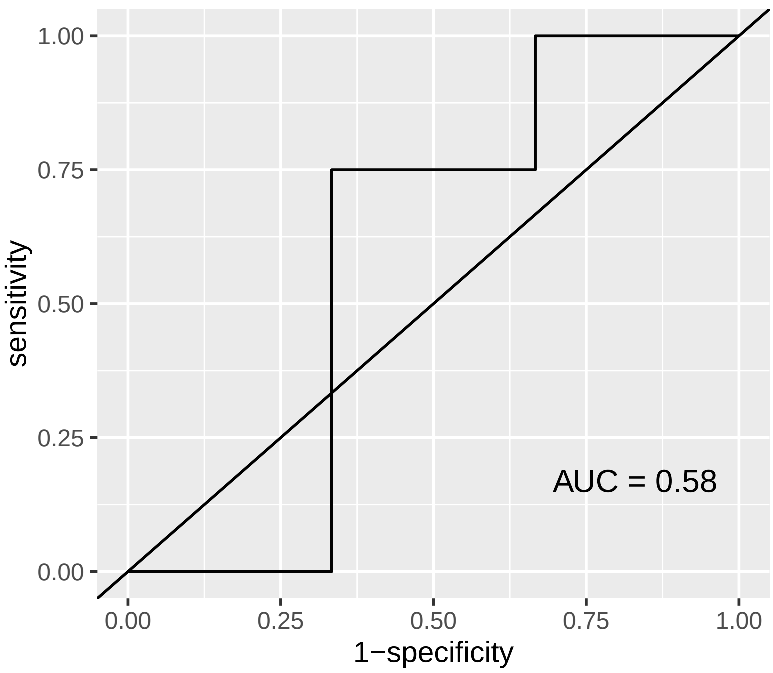

glcm_SumAverage

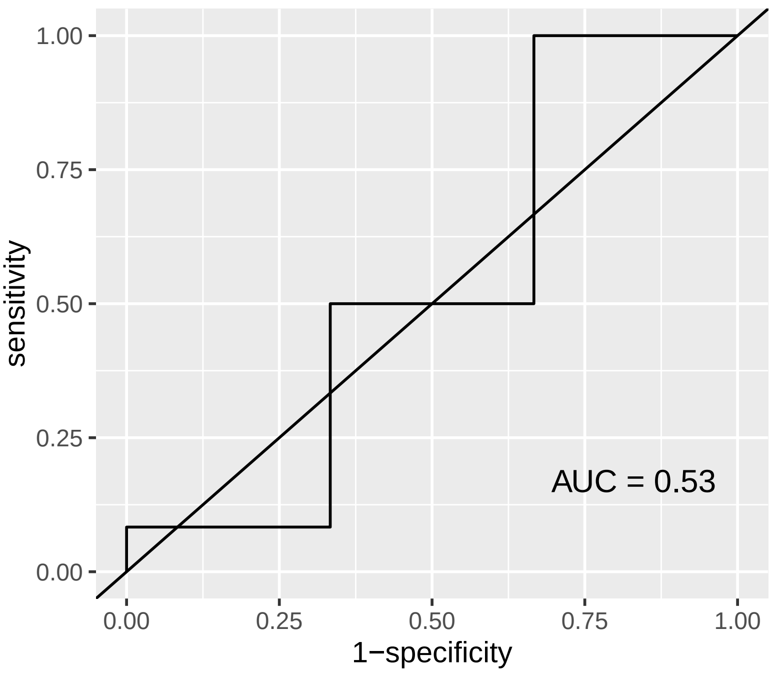

glcm_MaximumProbability

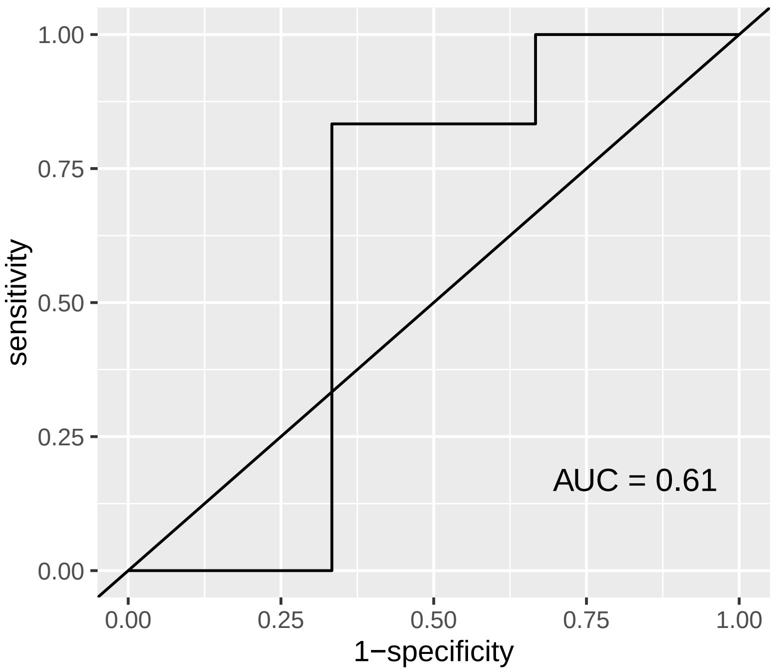

glcm_SumEntropy

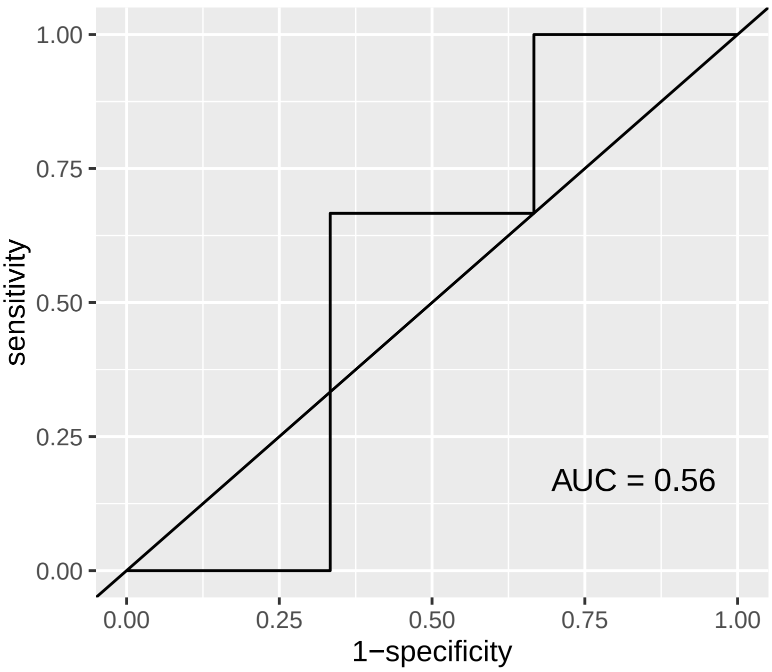

glcm_MCC

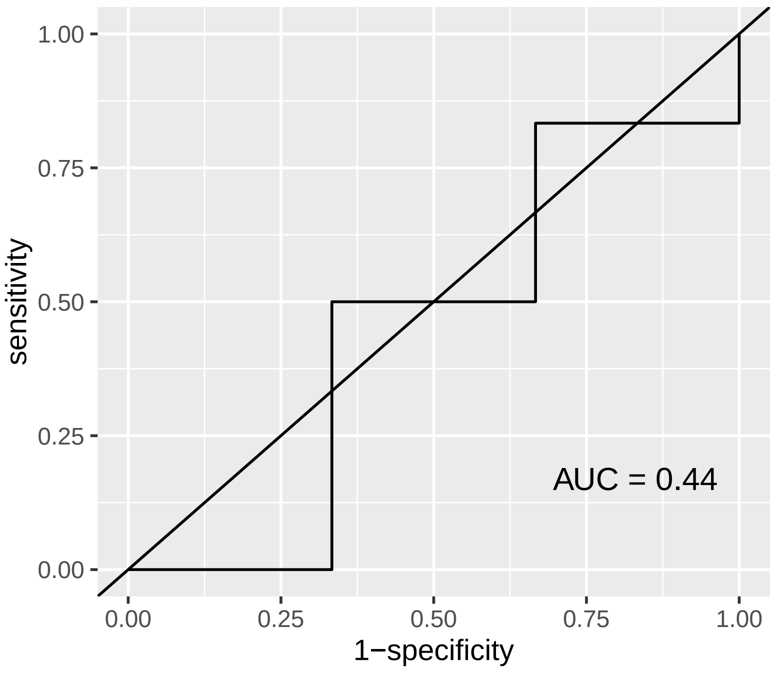

glcm_SumSquares

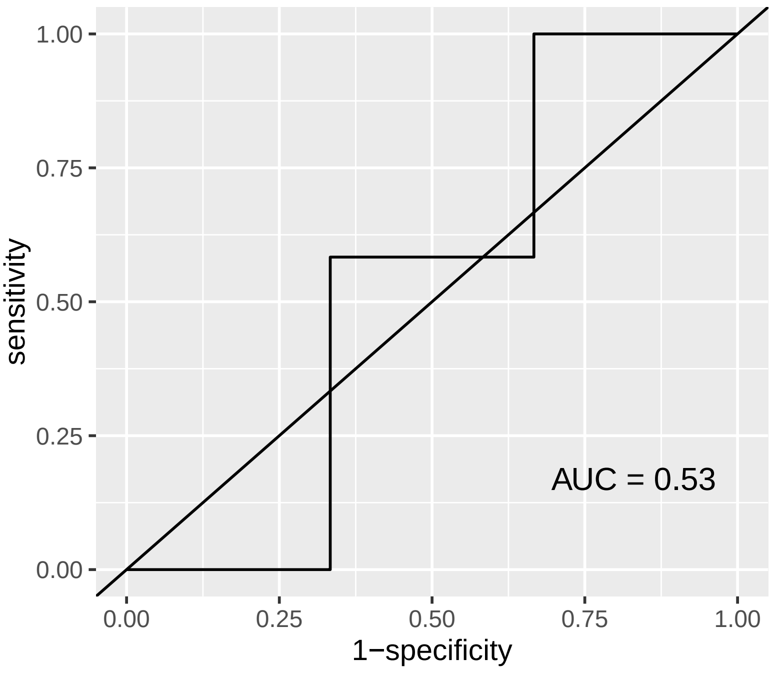

gldm_DependenceEntropy

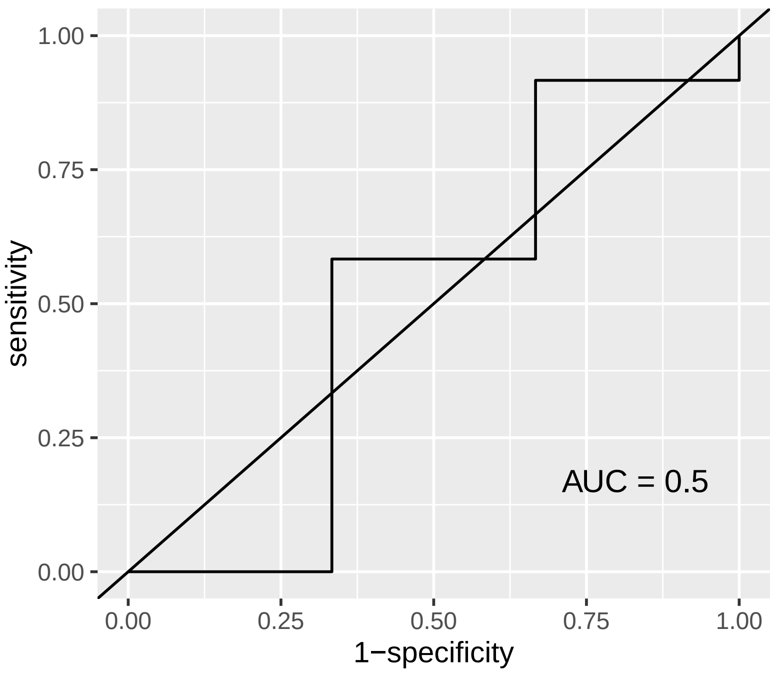

gldm_DependenceVariance

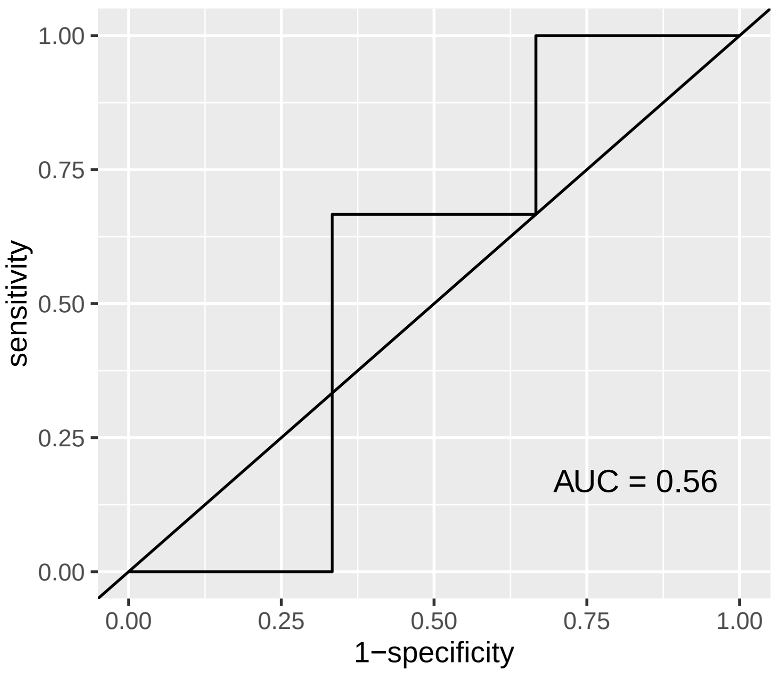

gldm_DependenceNonUniformity

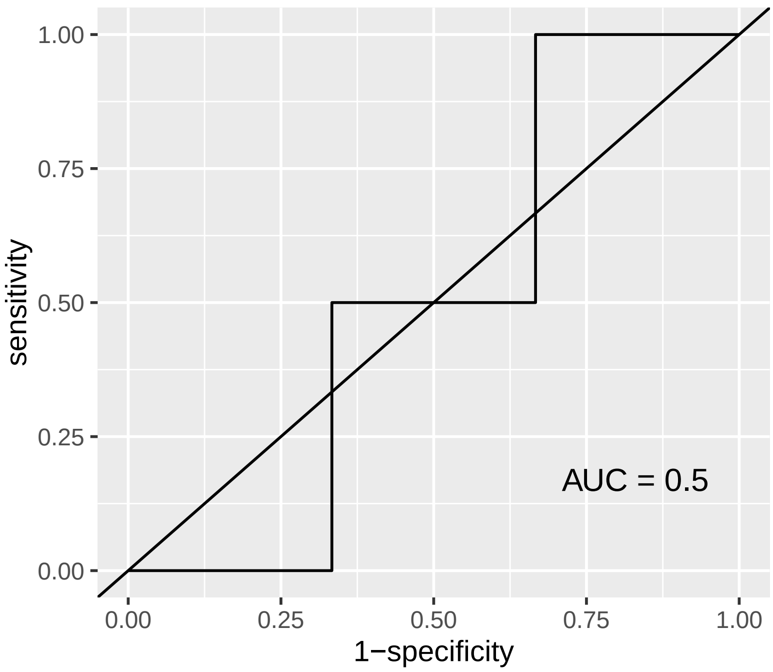

gldm_GrayLevelNonUniformity

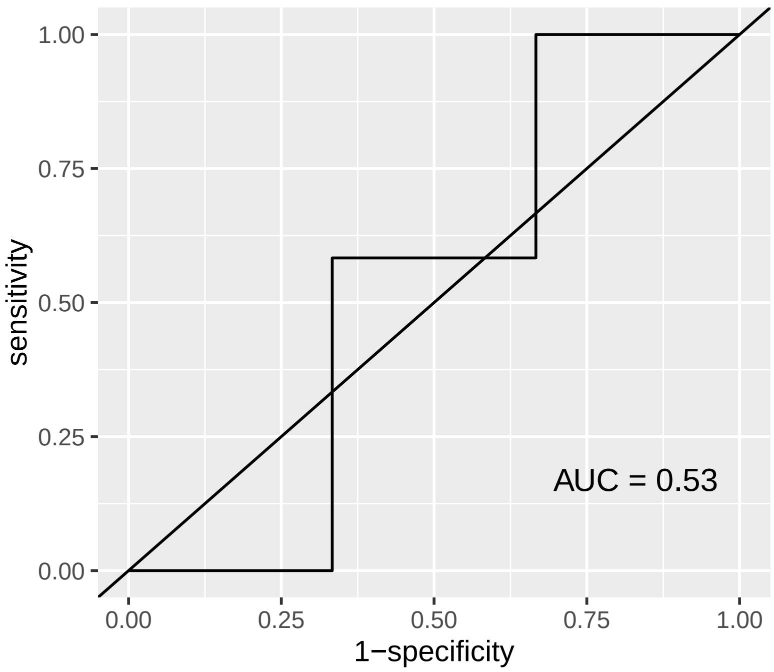

gldm_DependenceNonUniformityNormalized

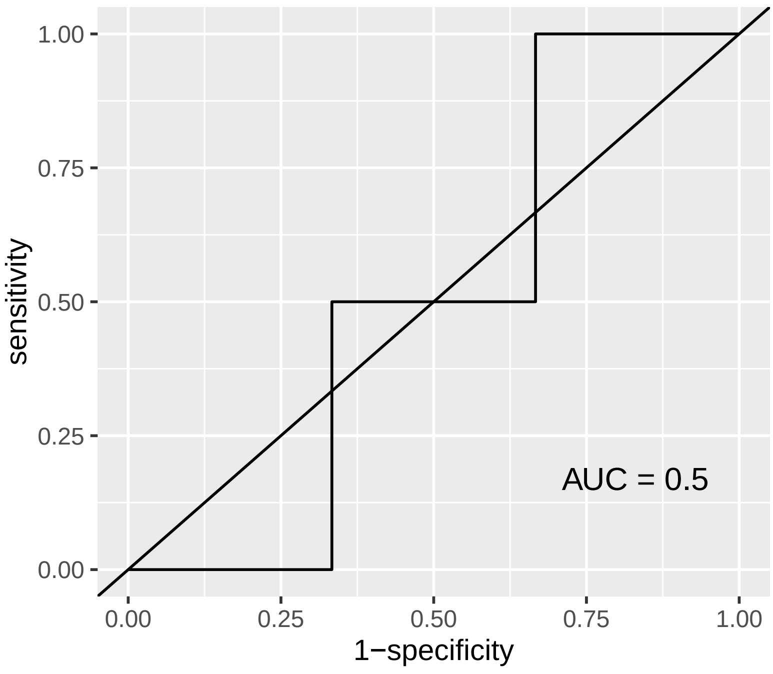

gldm_GrayLevelVariance

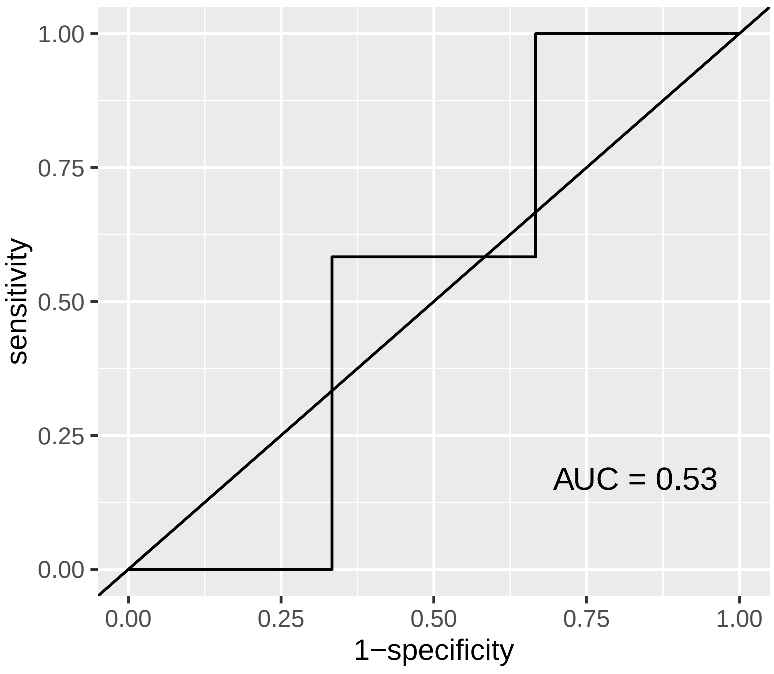

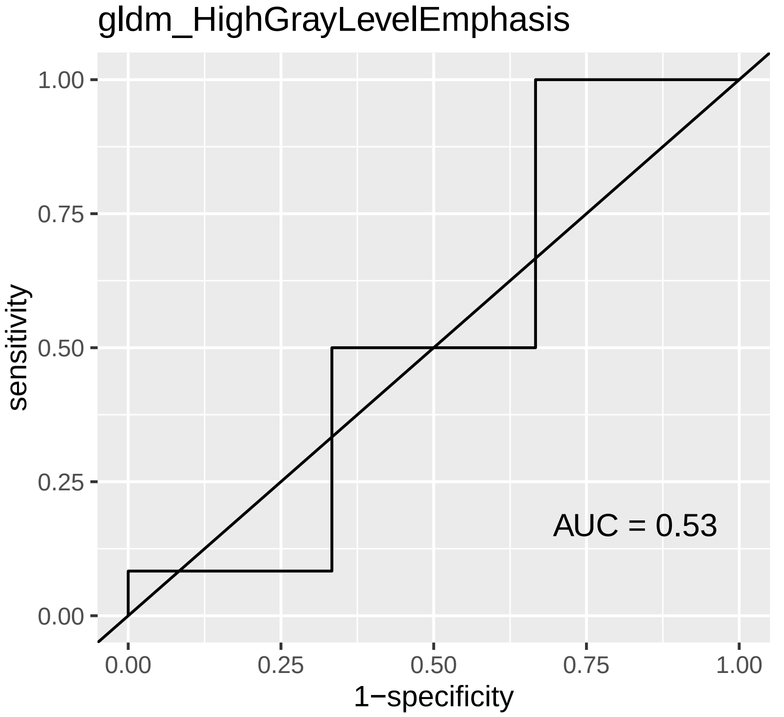
gldm_HighGrayLevelEmphasis
sensitivity
1−specificity
0.00
0.25
0.50
0.75
1.00
AUC = 0.53

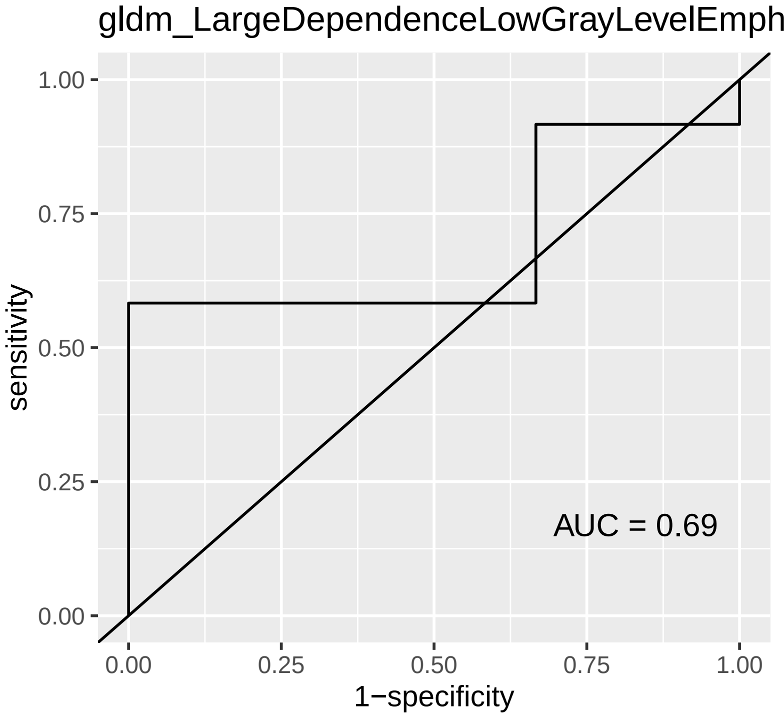
gldm_LargeDependenceLowGrayLevelEmph
sensitivity
1−specificity
0.00
0.25
0.50
0.75
1.00
AUC = 0.69

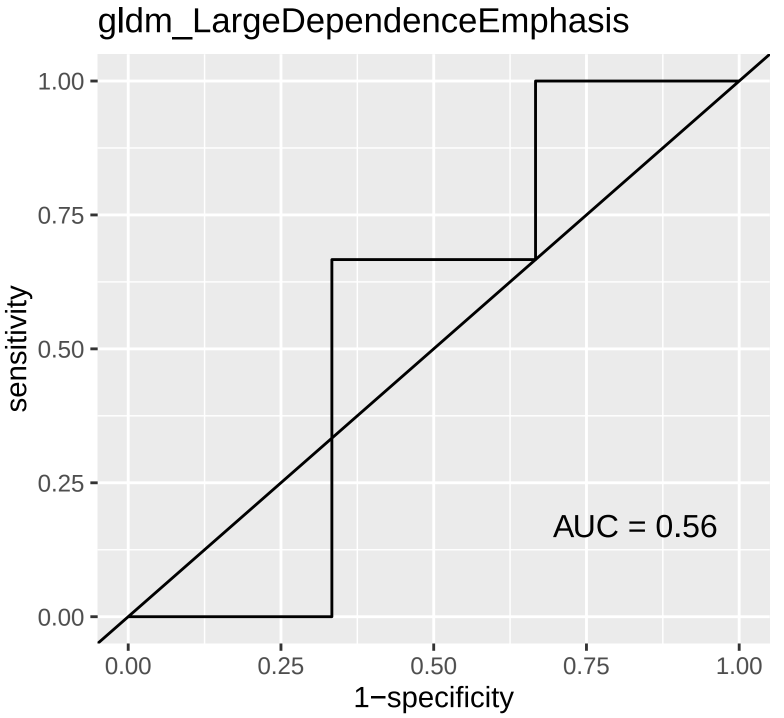
gldm_LargeDependenceEmphasis
sensitivity
1−specificity
0.00
0.25
0.50
0.75
1.00
AUC = 0.56

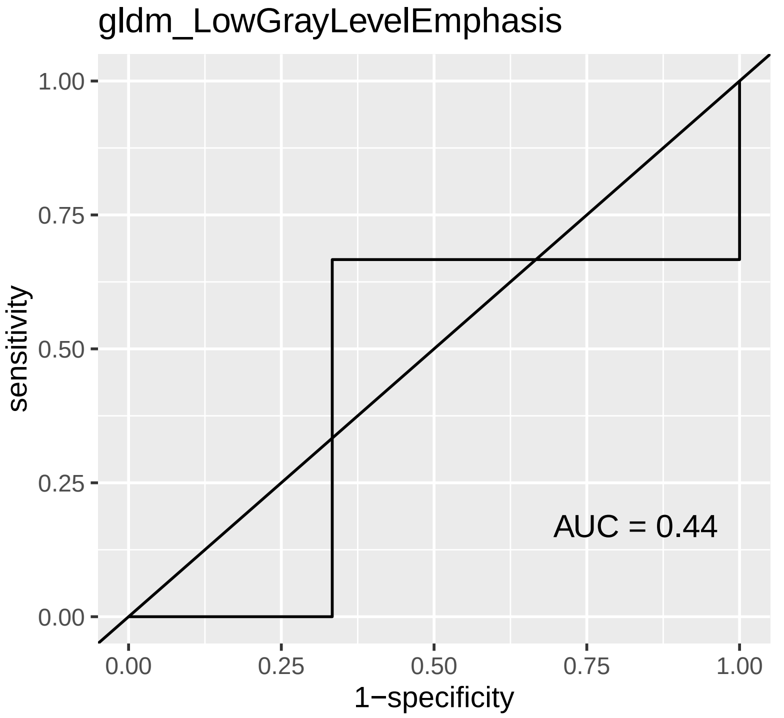
gldm_LowGrayLevelEmphasis
sensitivity
1−specificity
0.00
0.25
0.50
0.75
1.00
AUC = 0.44

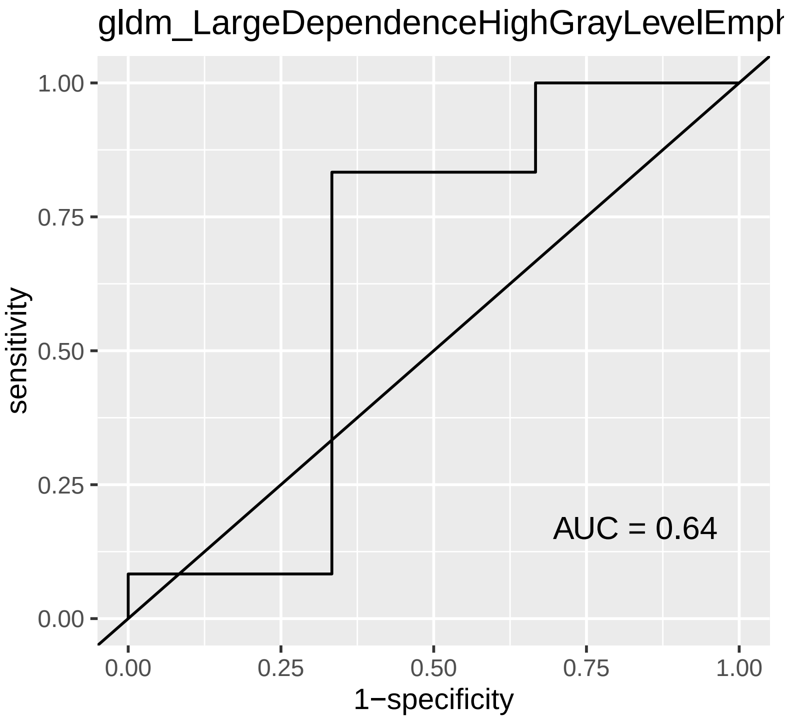
gldm_LargeDependenceHighGrayLevelEmph
sensitivity
1−specificity
0.00
0.25
0.50
0.75
1.00
AUC = 0.64

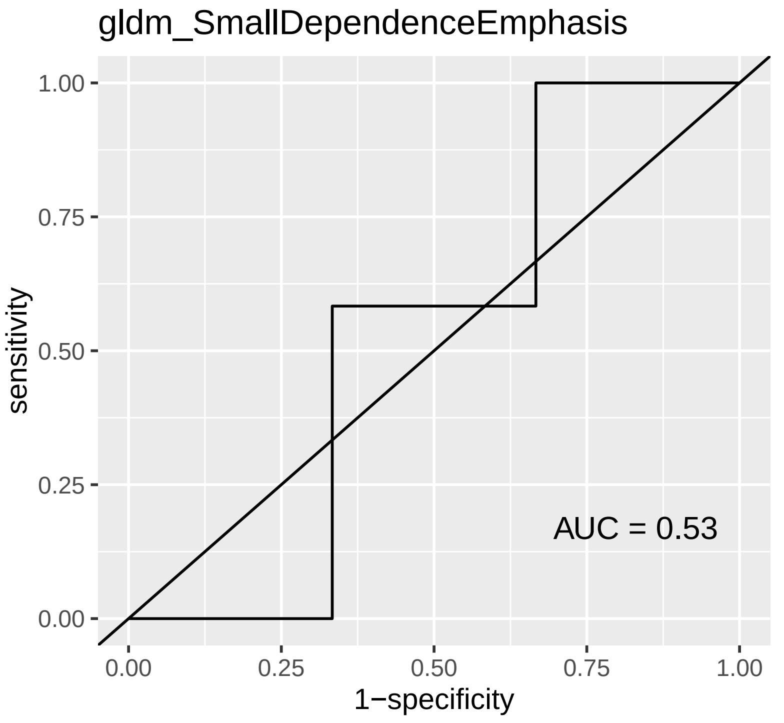
gldm_SmallDependenceEmphasis
sensitivity
1−specificity
0.00
0.25
0.50
0.75
1.00
AUC = 0.53

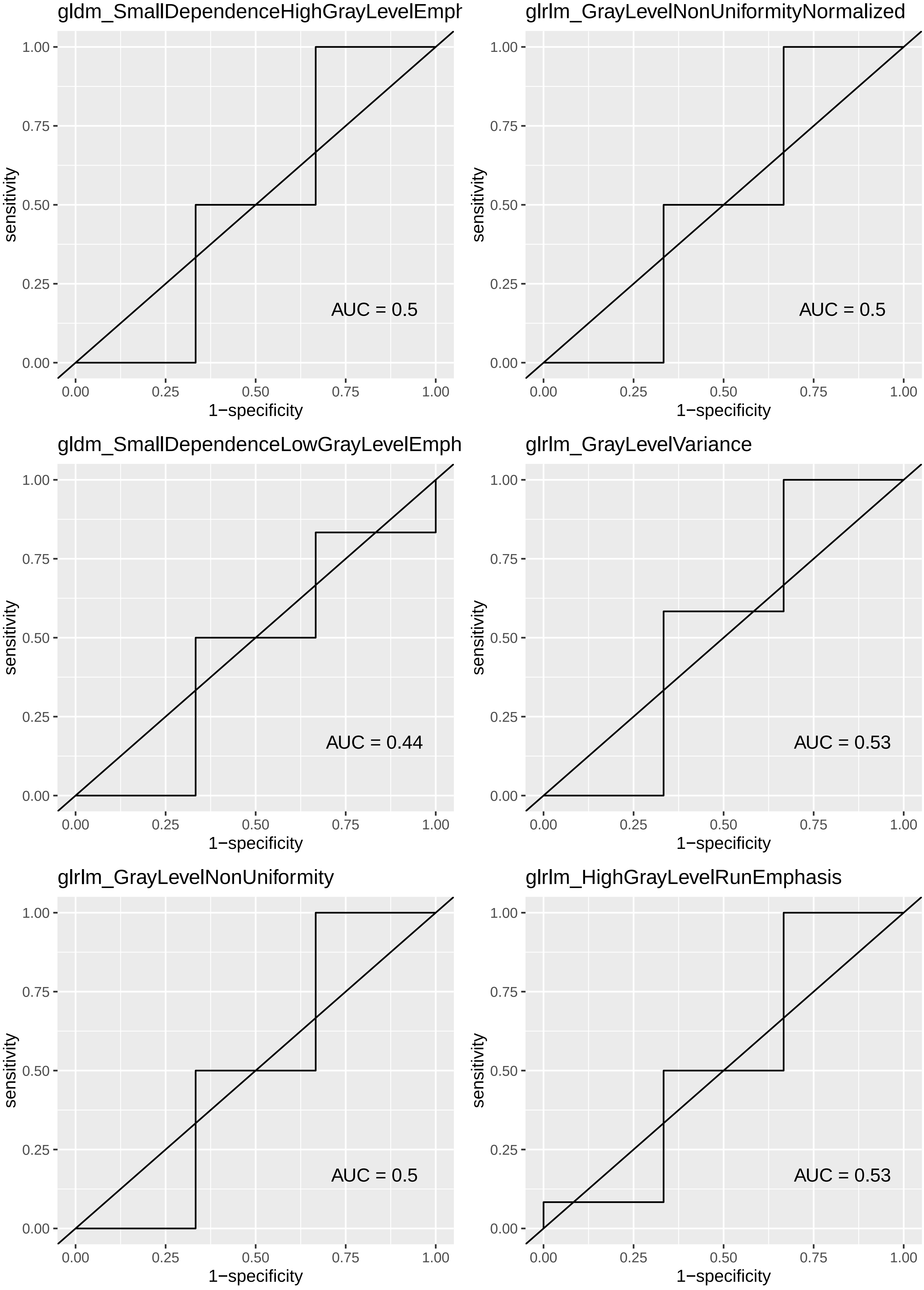
gldm_SmallDependenceHighGrayLevelEmph
glrlm_GrayLevelNonUniformityNormalized
gldm_SmallDependenceLowGrayLevelEmph
glrlm_GrayLevelVariance
glrlm_GrayLevelNonUniformity
glrlm_HighGrayLevelRunEmphasis
sensitivity
1−specificity
0.00
0.25
0.50
0.75
1.00
AUC = 0.5
AUC = 0.5
AUC = 0.44
AUC = 0.53
AUC = 0.5
AUC = 0.53

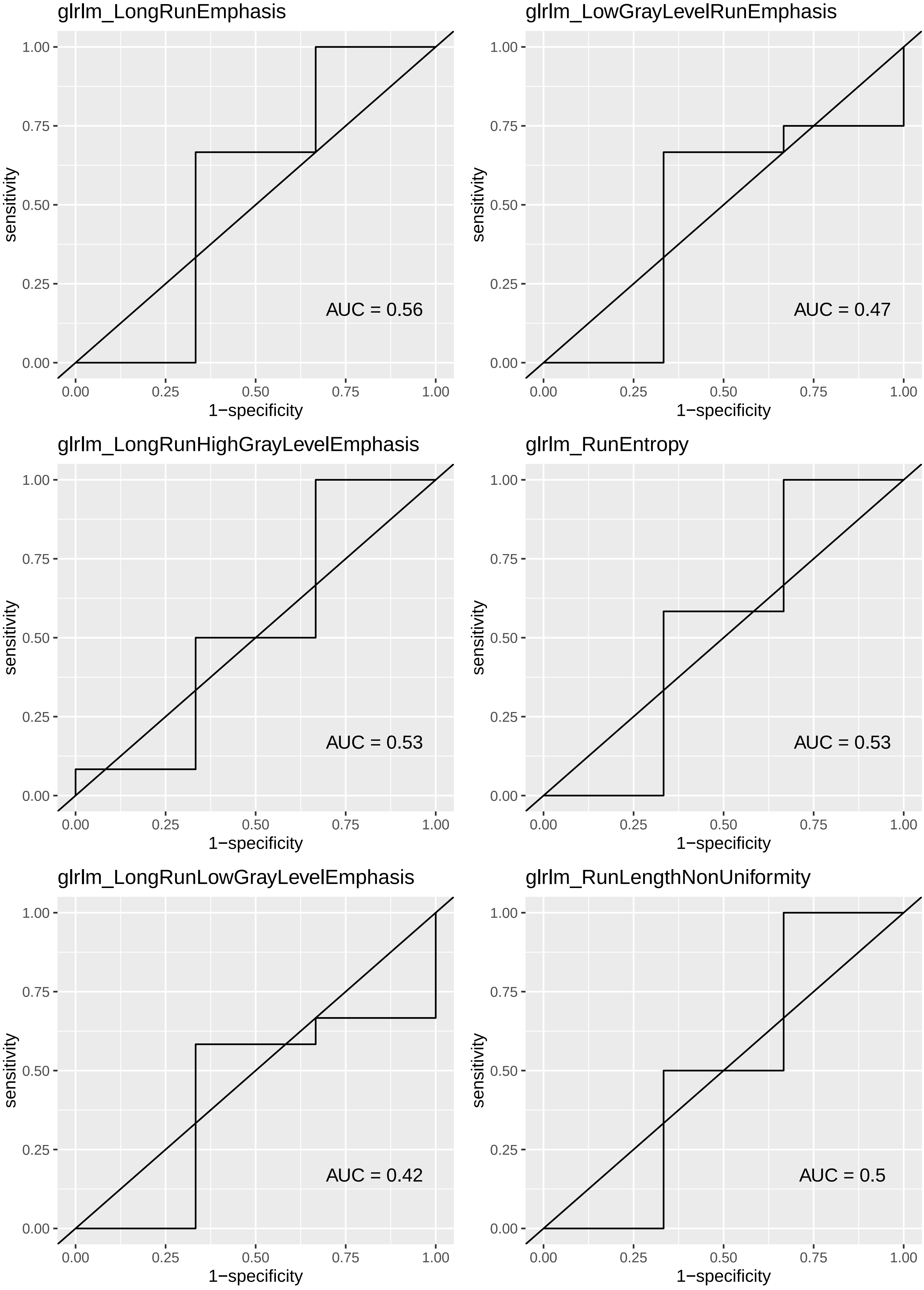
glrlm_LongRunEmphasis
AUC = 0.56
glrlm_LowGrayLevelRunEmphasis
AUC = 0.47
glrlm_LongRunHighGrayLevelEmphasis
AUC = 0.53
glrlm_RunEntropy
AUC = 0.53
glrlm_LongRunLowGrayLevelEmphasis
AUC = 0.42
glrlm_RunLengthNonUniformity
AUC = 0.5
sensitivity
1−specificity
0.00
0.25
0.50
0.75
1.00

glrlm_RunLengthNonUniformityNormalized

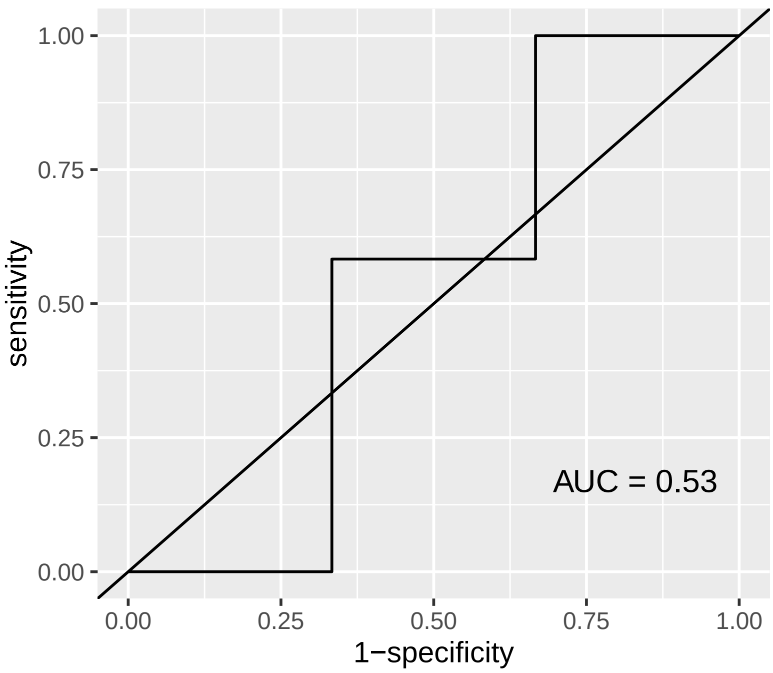

glrlm_ShortRunEmphasis

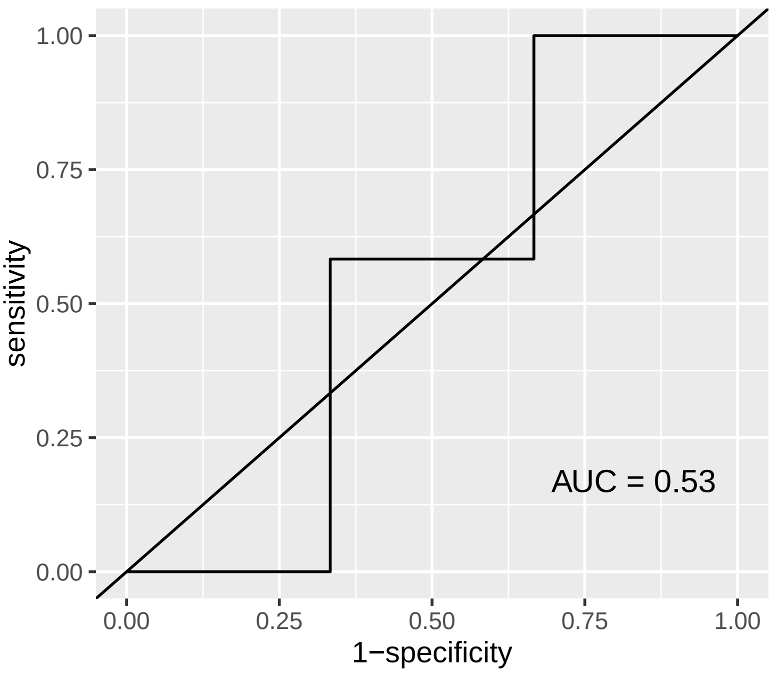

glrlm_RunPercentage

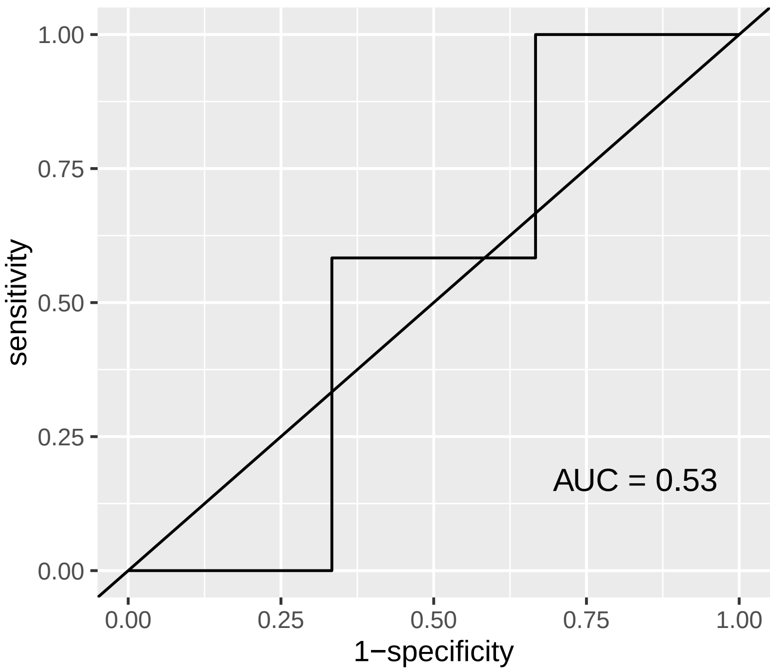

glrlm_ShortRunHighGrayLevelEmphasis

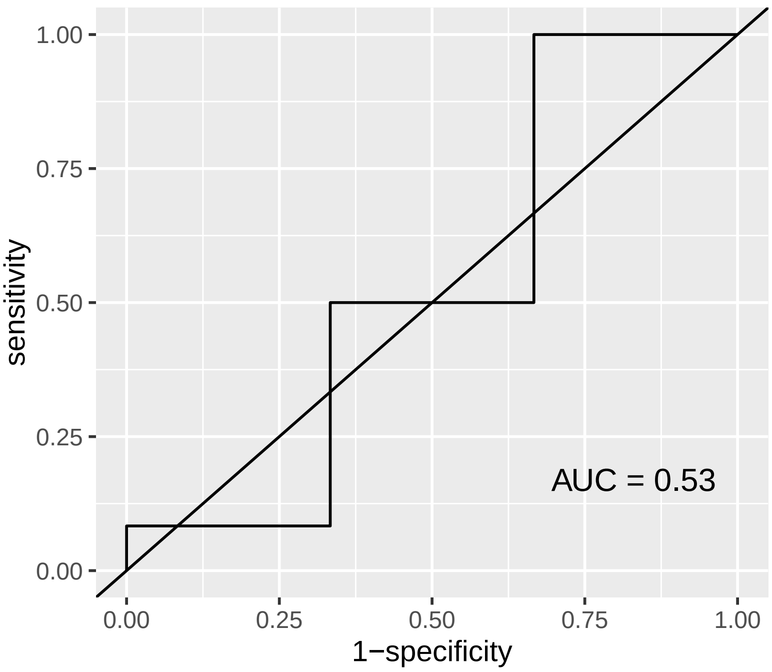

glrlm_RunVariance

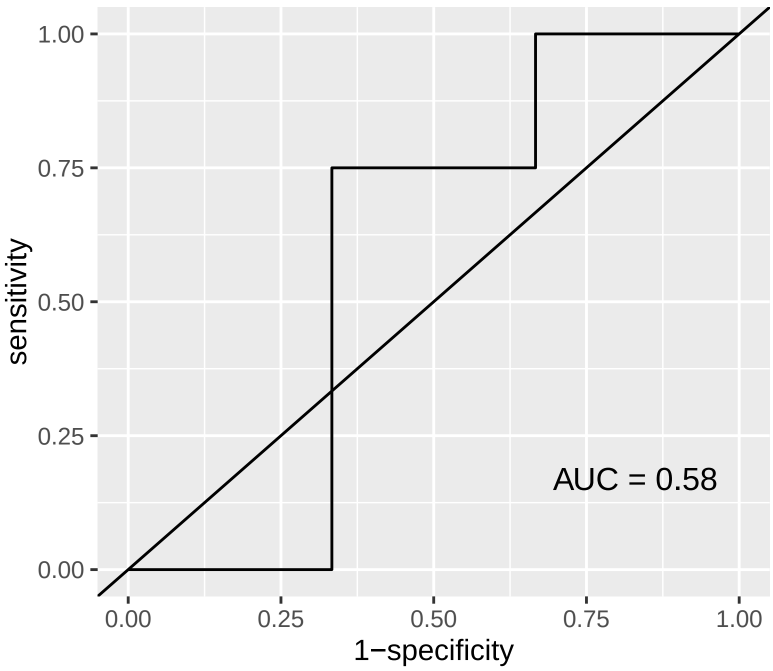

glrlm_ShortRunLowGrayLevelEmphasis

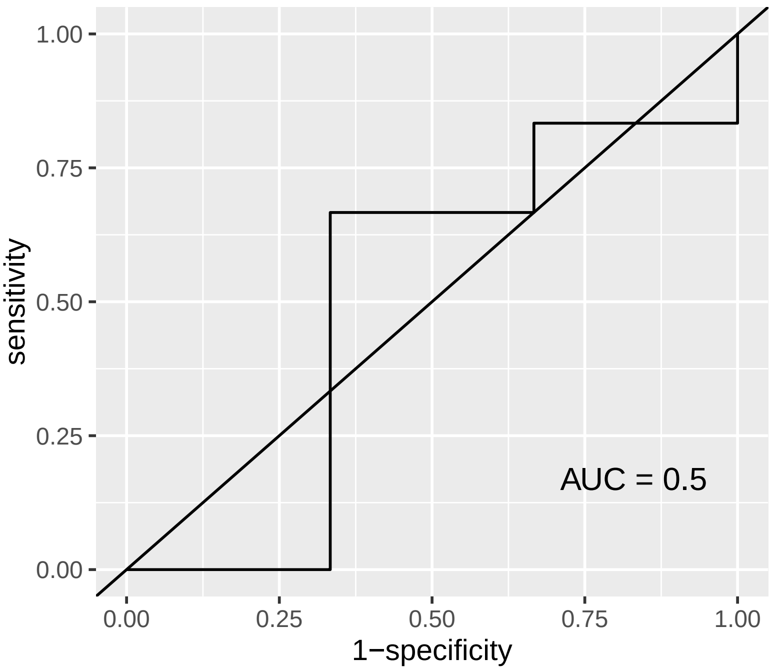

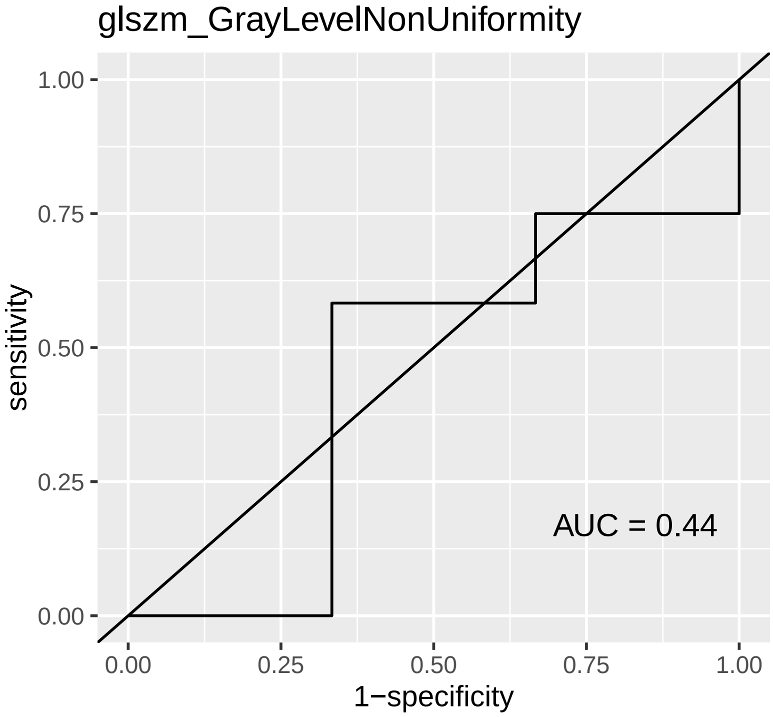
glszm_GrayLevelNonUniformity
sensitivity
1−specificity
AUC = 0.44
0.00
0.25
0.50
0.75
1.00

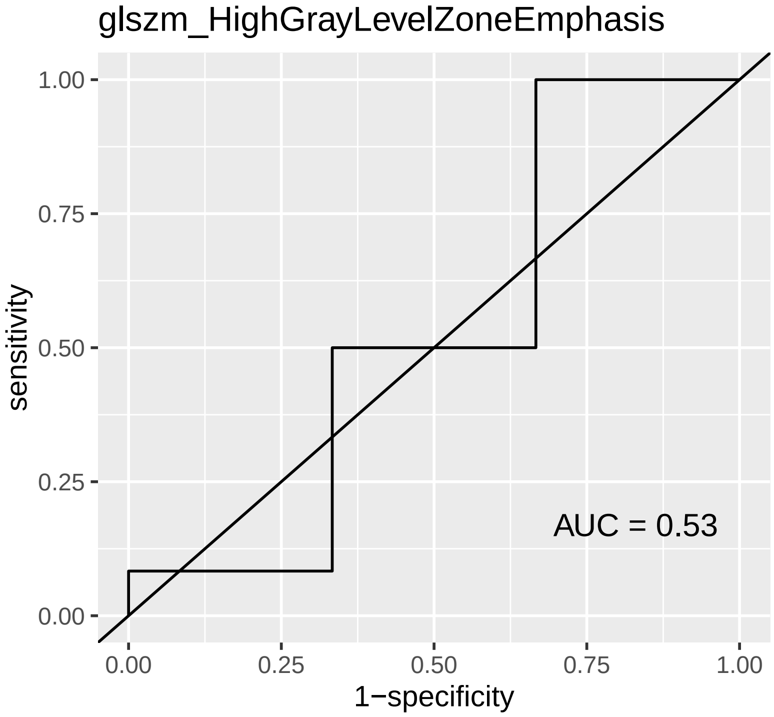
glszm_HighGrayLevelZoneEmphasis
sensitivity
1−specificity
AUC = 0.53
0.00
0.25
0.50
0.75
1.00

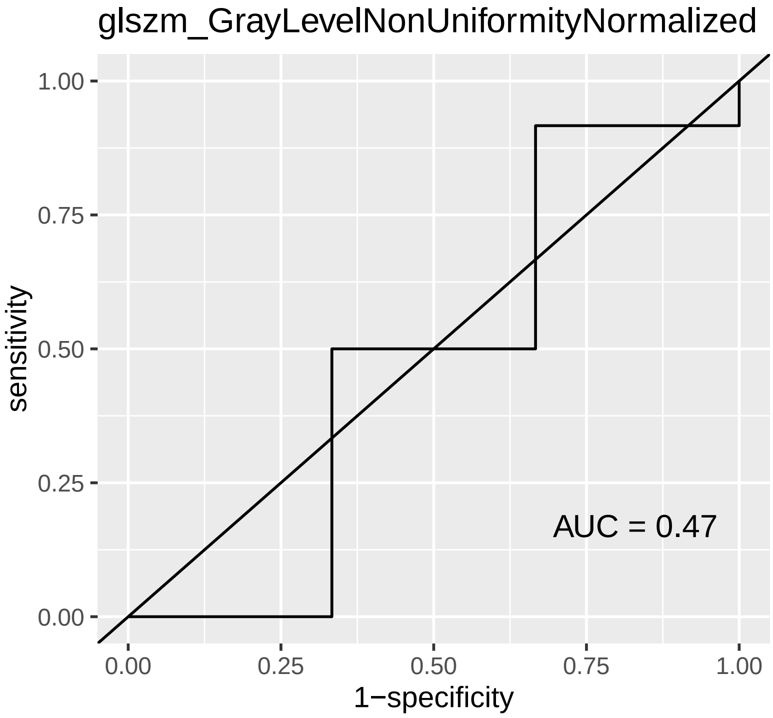
glszm_GrayLevelNonUniformityNormalized
sensitivity
1−specificity
AUC = 0.47
0.00
0.25
0.50
0.75
1.00

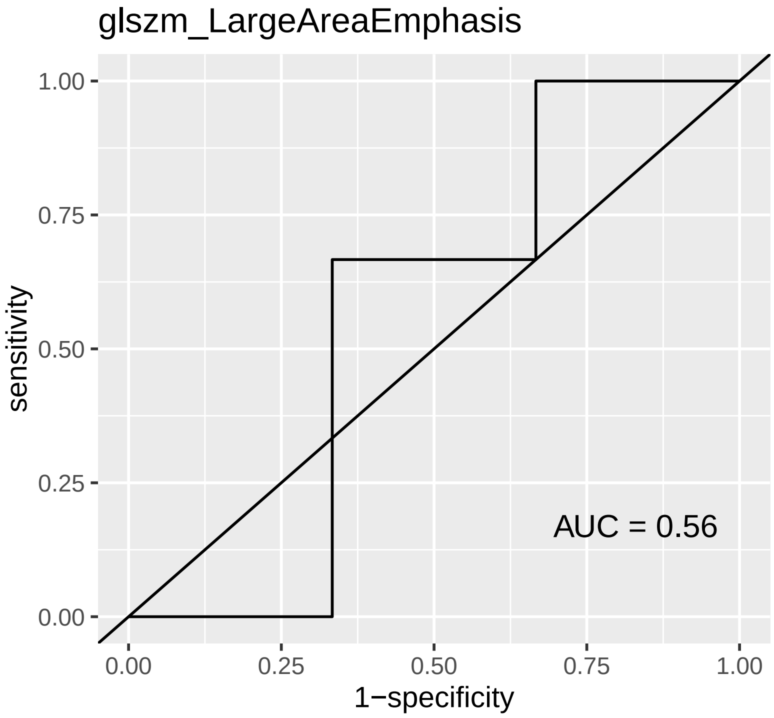
glszm_LargeAreaEmphasis
sensitivity
1−specificity
AUC = 0.56
0.00
0.25
0.50
0.75
1.00

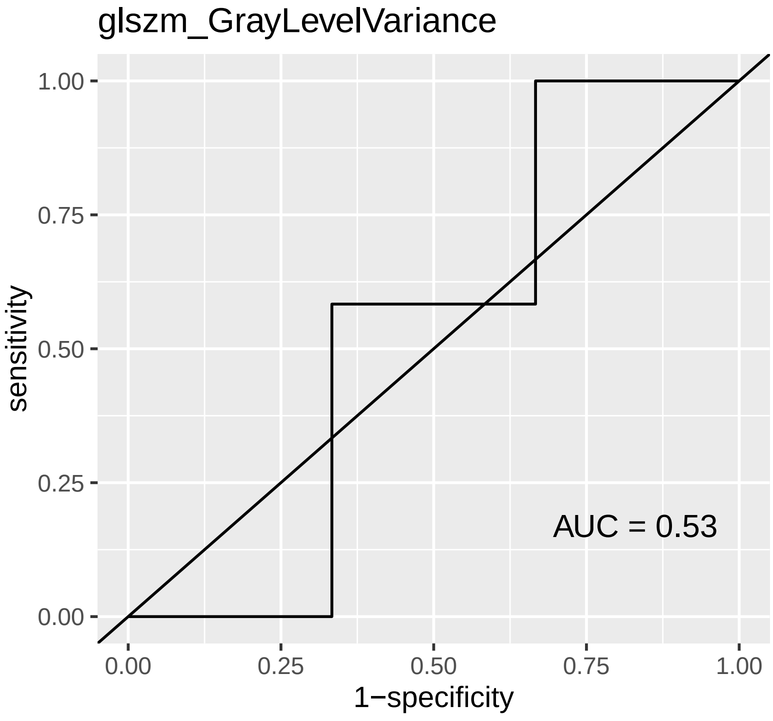
glszm_GrayLevelVariance
sensitivity
1−specificity
AUC = 0.53
0.00
0.25
0.50
0.75
1.00

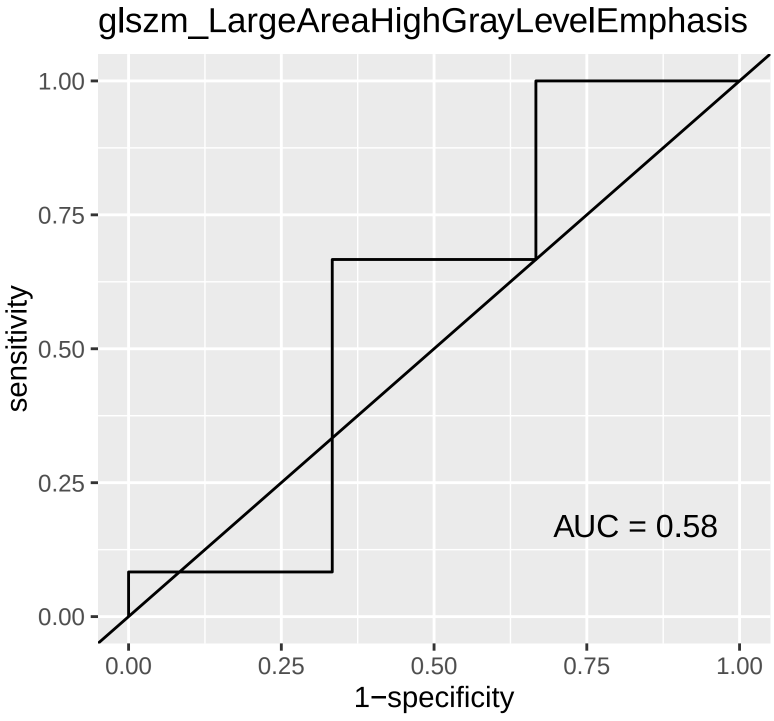
glszm_LargeAreaHighGrayLevelEmphasis
sensitivity
1−specificity
AUC = 0.58
0.00
0.25
0.50
0.75
1.00

glszm_LargeAreaLowGrayLevelEmphasis

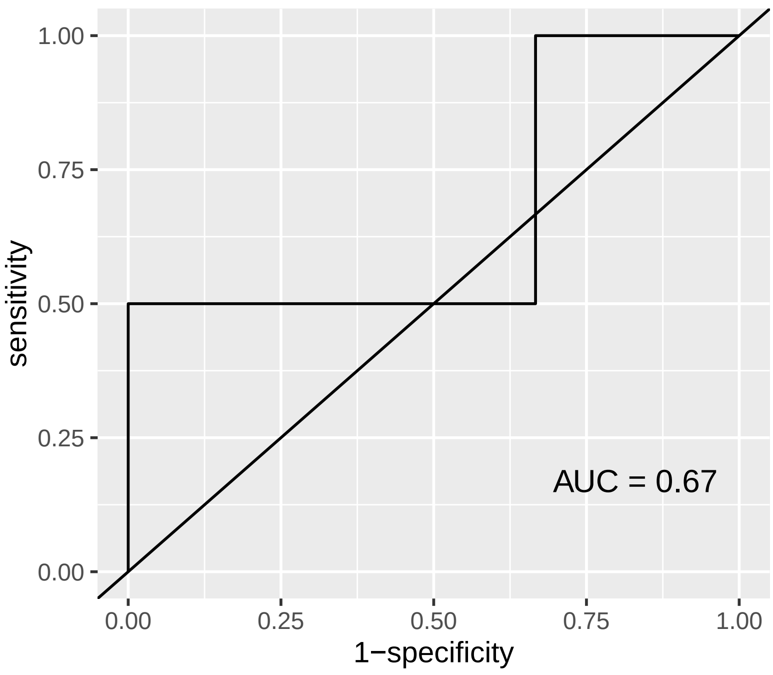

glszm_SizeZoneNonUniformityNormalized

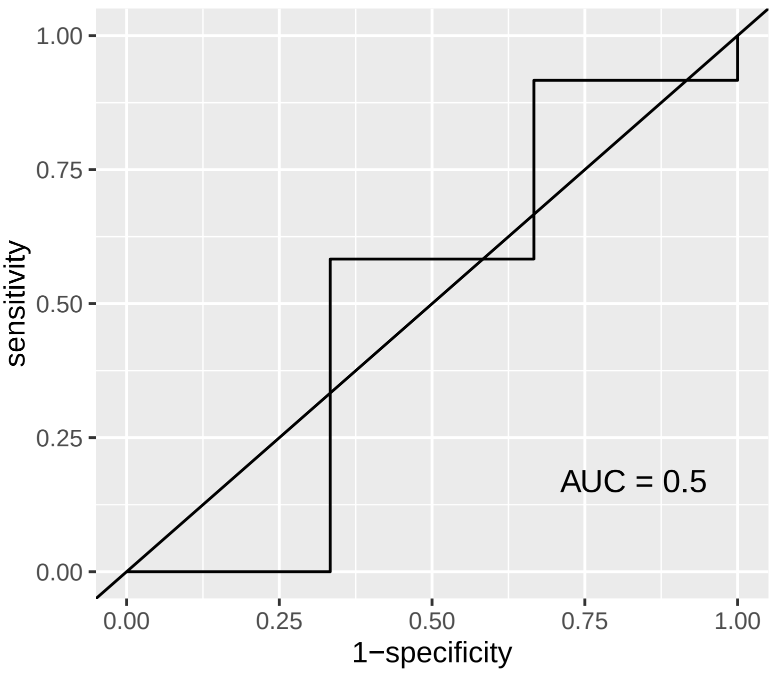

glszm_LowGrayLevelZoneEmphasis

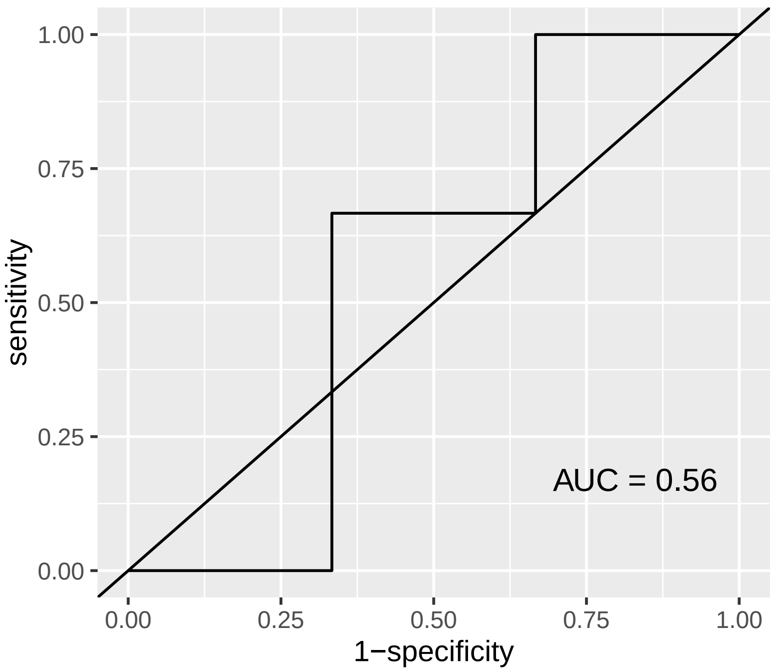

glszm_SmallAreaEmphasis

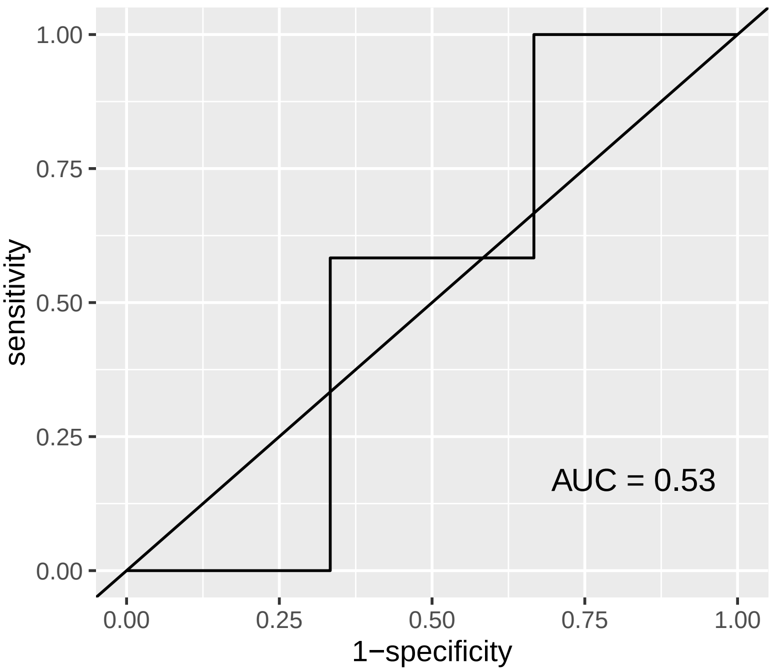

glszm_SizeZoneNonUniformity

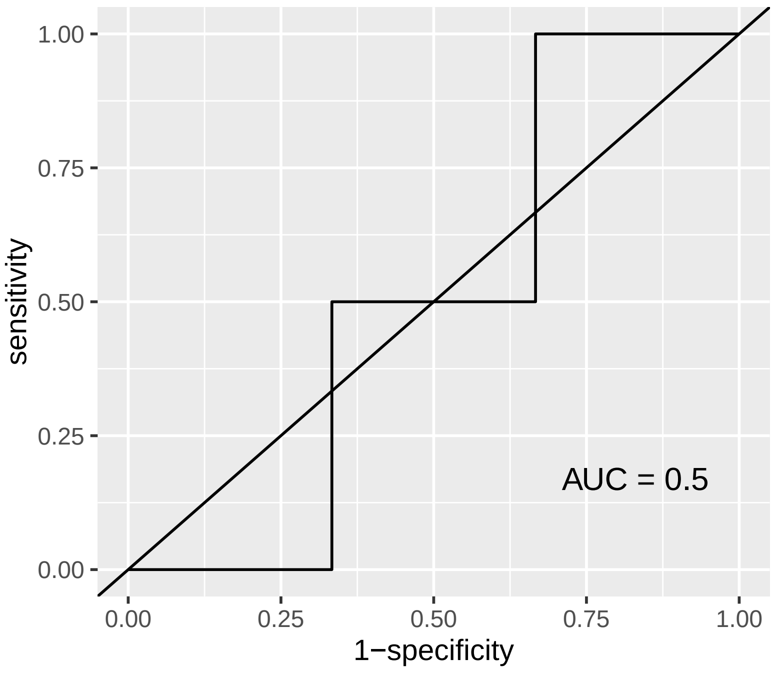

glszm_SmallAreaHighGrayLevelEmphasis

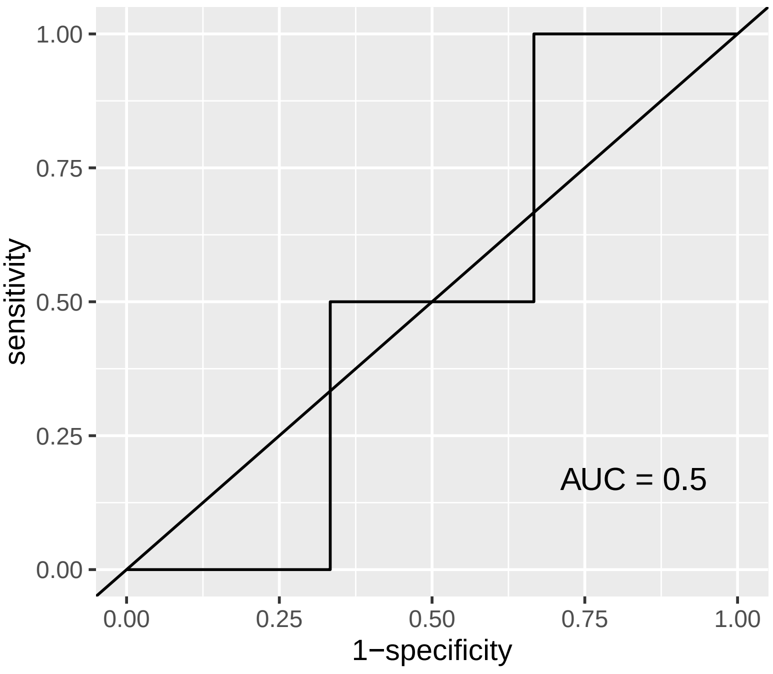

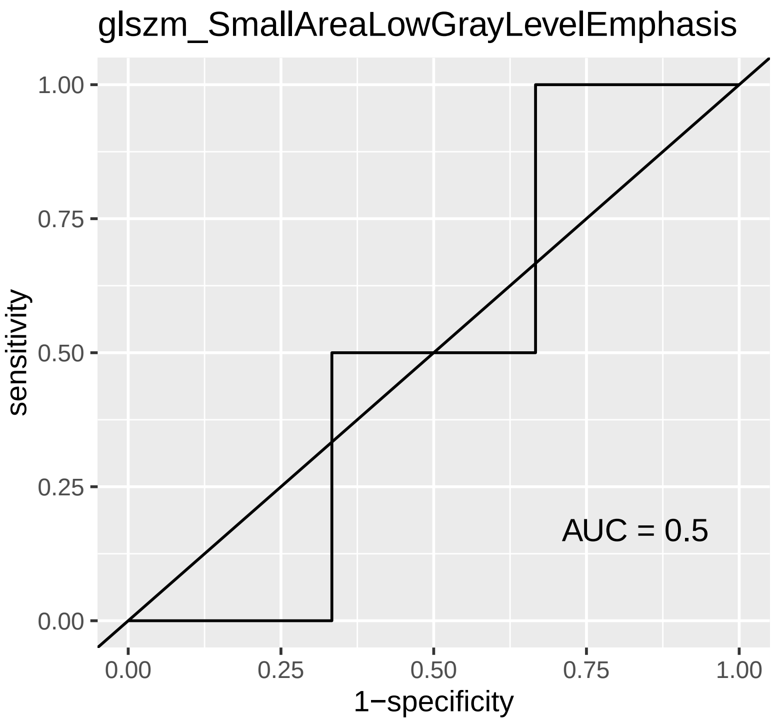
glszm_SmallAreaLowGrayLevelEmphasis
sensitivity
1−specificity
AUC = 0.5
0.00
0.25
0.50
0.75
1.00

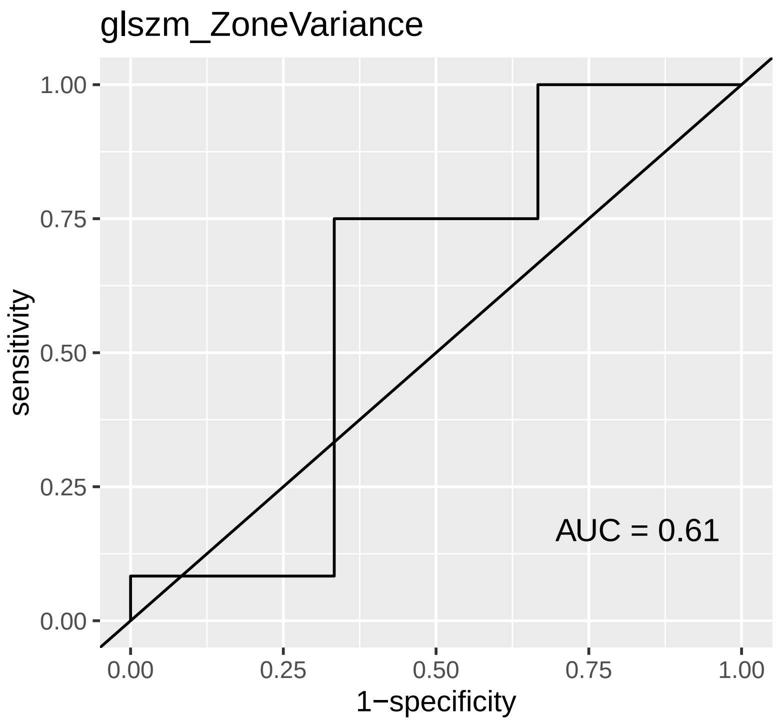
glszm_ZoneVariance
sensitivity
1−specificity
AUC = 0.61
0.00
0.25
0.50
0.75
1.00

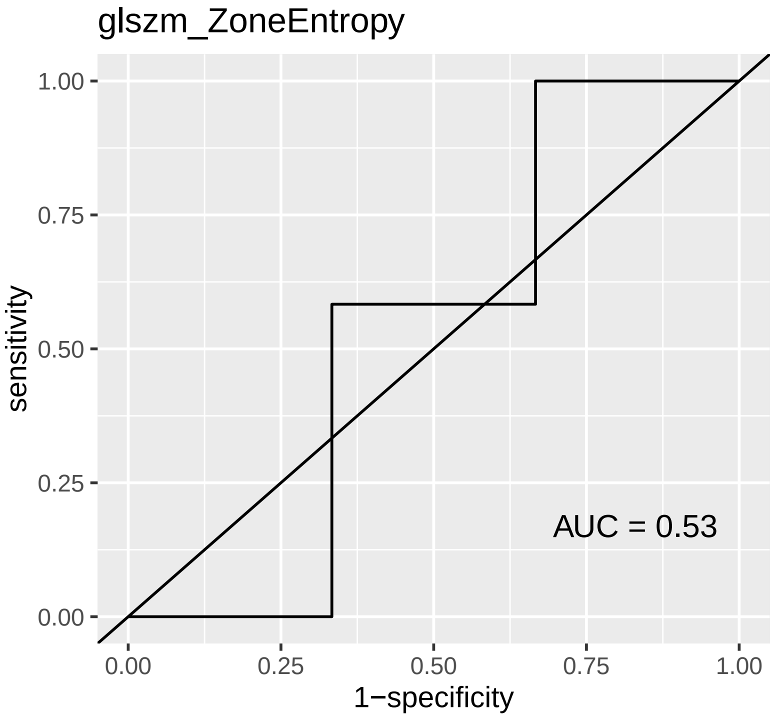
glszm_ZoneEntropy
sensitivity
1−specificity
AUC = 0.53
0.00
0.25
0.50
0.75
1.00

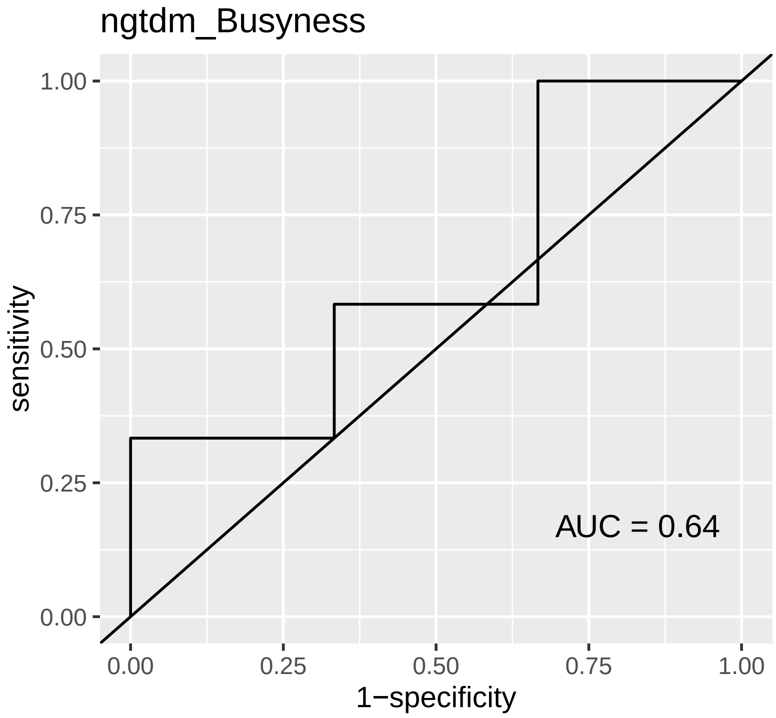
ngtdm_Busyness
sensitivity
1−specificity
AUC = 0.64
0.00
0.25
0.50
0.75
1.00

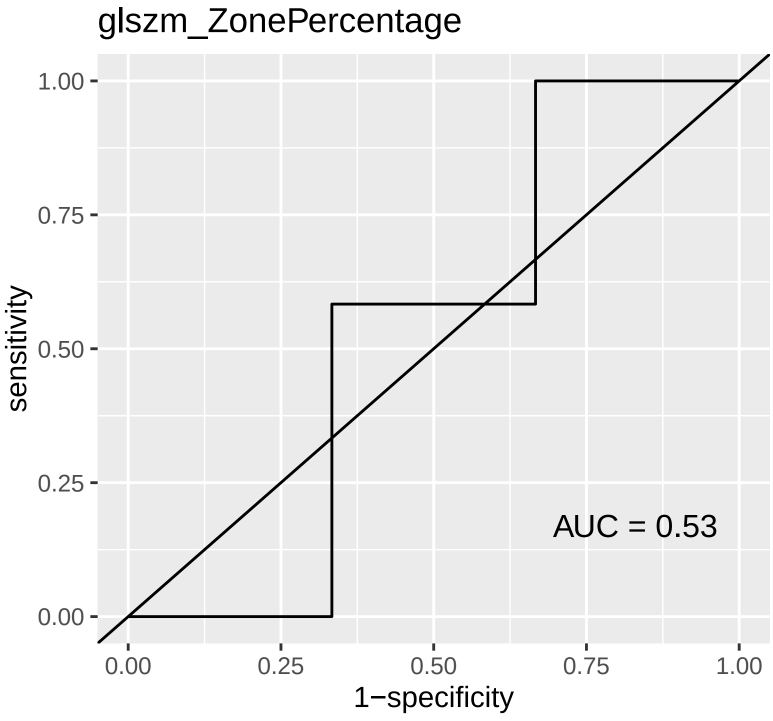
glszm_ZonePercentage
sensitivity
1−specificity
AUC = 0.53
0.00
0.25
0.50
0.75
1.00

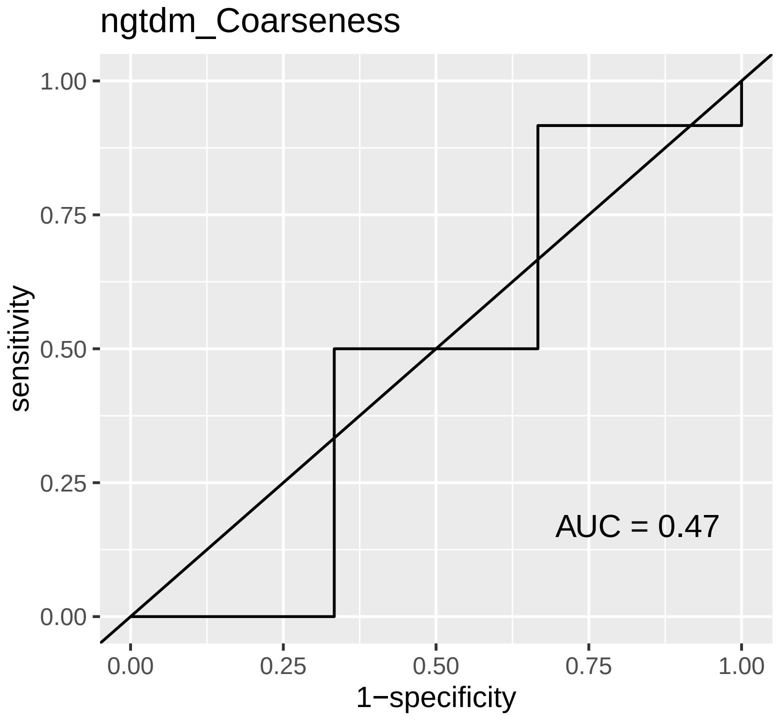
ngtdm_Coarseness
sensitivity
1−specificity
AUC = 0.47
0.00
0.25
0.50
0.75
1.00

ngtdm_Complexity

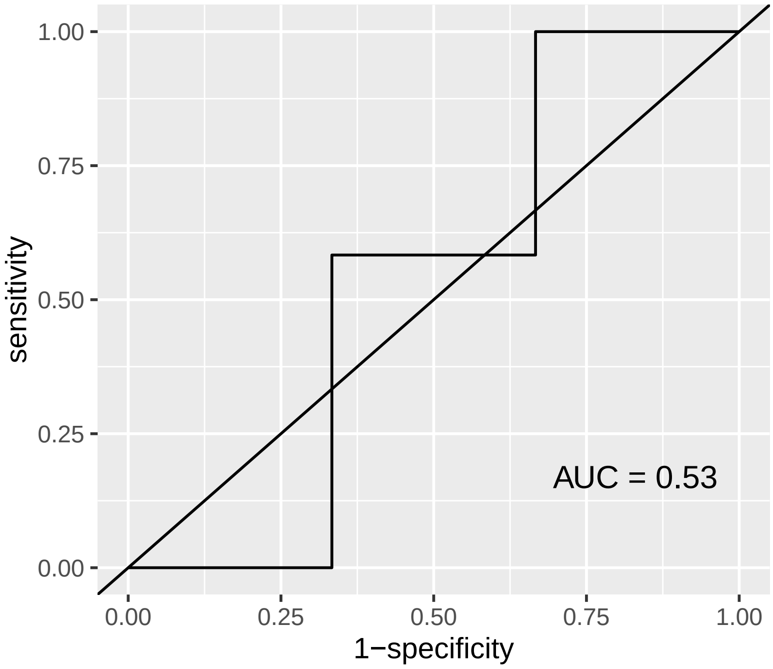

ngtdm_Contrast

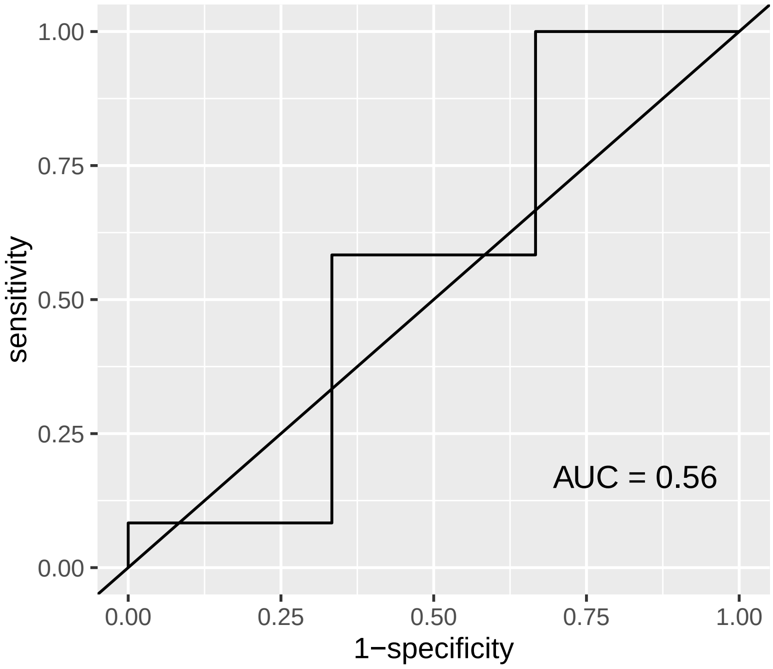

ngtdm_Strength

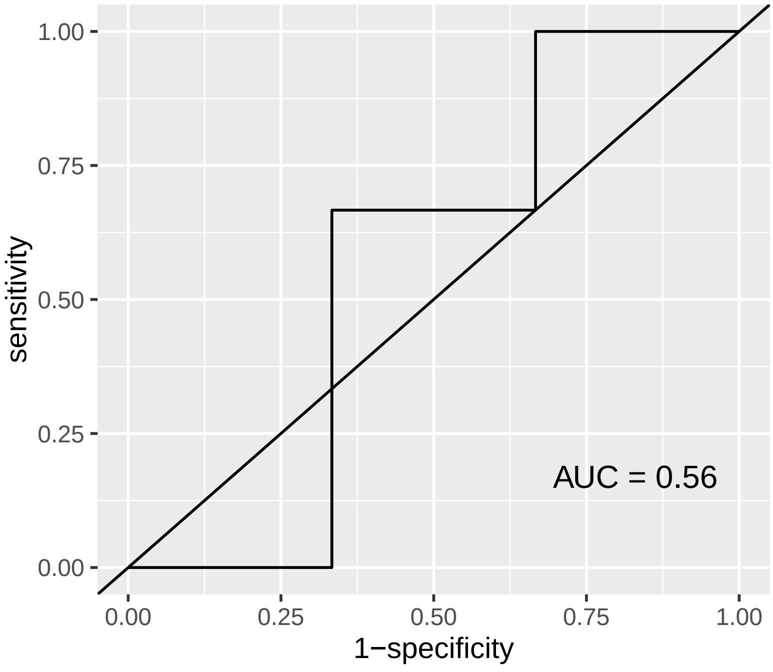

Supplement: Supplementary file 1 [file curroncol-31-00503-s001.zip › S11_ROCplots_feature_maps_TZ_only.pdf]
