# Supplementary material for: Detecting Clinically Significant Prostate Cancer in PI-RADS 3 Lesions Using T2w-Derived Radiomics Feature Maps in 3T Prostate MRI
Source: Curr Oncol. 2024 Nov 1;31(11):6814–28. doi: 10.3390/curroncol31110503 (PMC11592716; doi:10.3390/curroncol31110503)
Supplement: Supplementary file 1 [file curroncol-31-00503-s001.zip › S1_axial_T2_TSE_ sequence_technical_details.pdf]

File S1: Details of the axial T2-weighted turbo spin echo sequence.

	Scanner 1
Magnetic field strength	3 Tesla
Patient position	supine, arms positioned next to the body, 18-channel body phased-array coil positioned on pelvis
Angulation	oblique axial plane to bladder neck
Field of view* (mm)	180 x 180
Acquisition matrix	384 x 326
Phase encoding direction	rows (left to right)
Target TR/TE (ms)	4000/116
Preset flip angle (degrees)	160
Number of phase encoding steps	651
Slice thickness (mm)	3
Spacing between slices	0.3
Number of averages	2
Buscopan i.m. (with/without)	32/11

TE: echo time; TR: repetition time; *with prostate in the center of the field of view
