# Supplementary material for: Detecting Clinically Significant Prostate Cancer in PI-RADS 3 Lesions Using T2w-Derived Radiomics Feature Maps in 3T Prostate MRI
Source: Curr Oncol. 2024 Nov 1;31(11):6814–28. doi: 10.3390/curroncol31110503 (PMC11592716; doi:10.3390/curroncol31110503)

firstorder_10Percentile

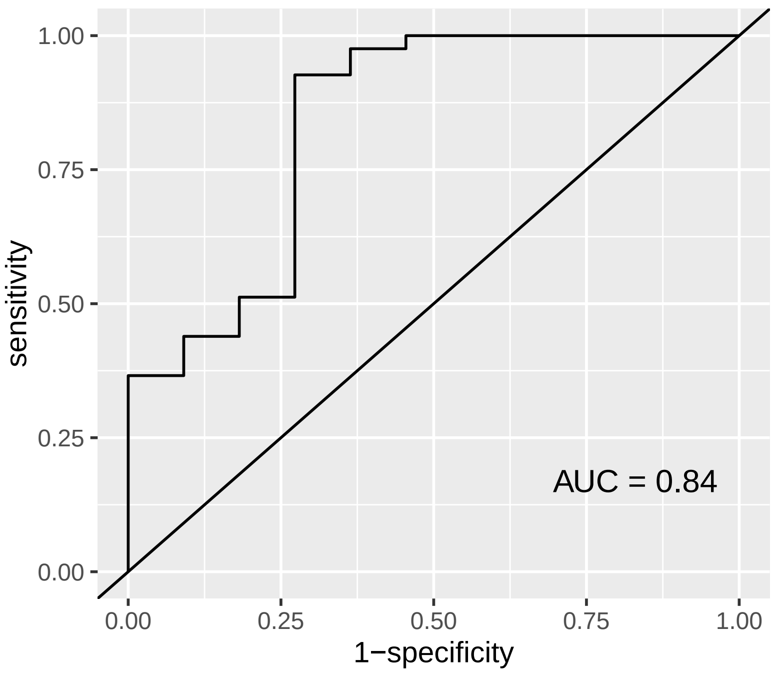

firstorder_Entropy

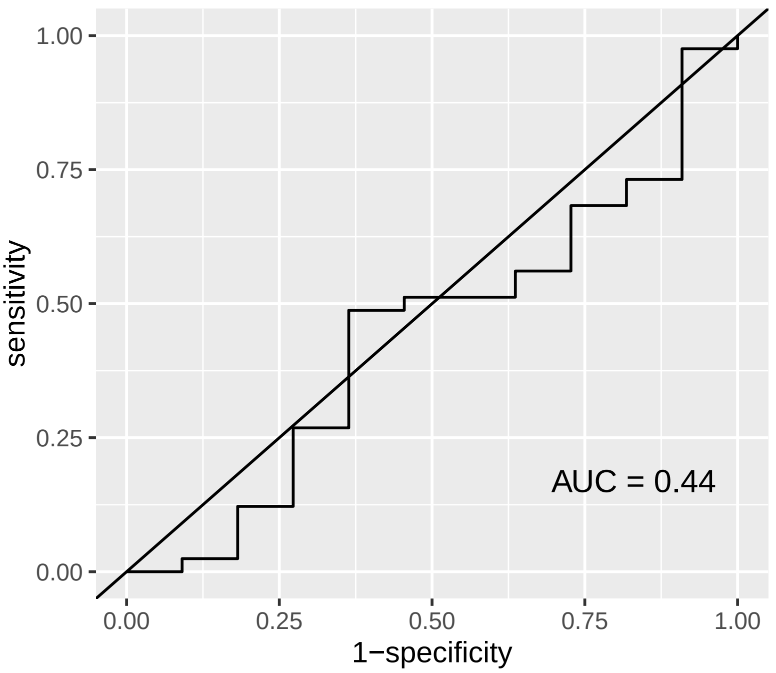

firstorder_90Percentile

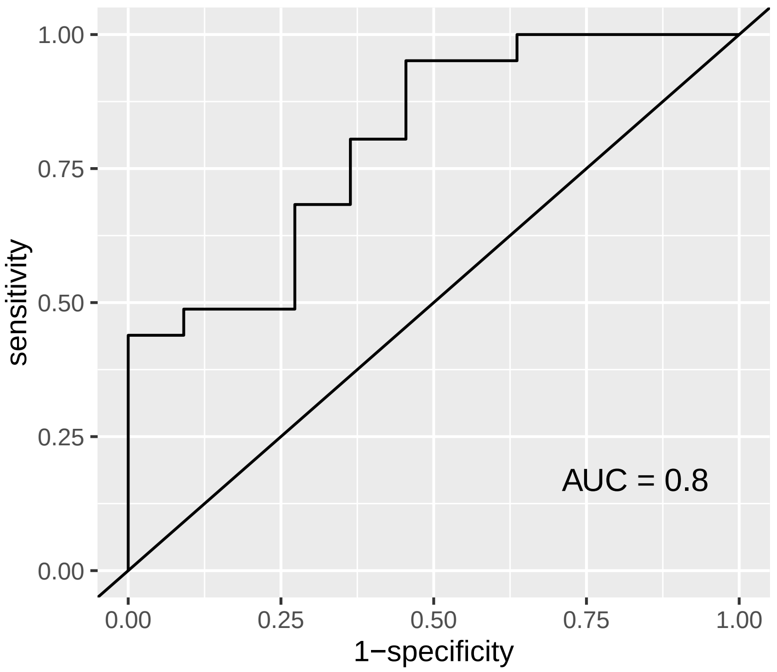

firstorder_InterquartileRange

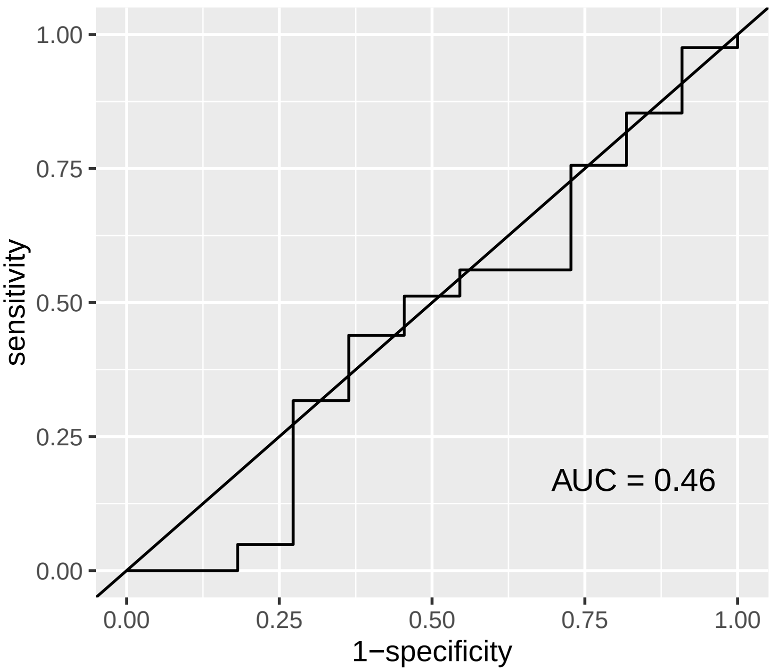

firstorder_Energy

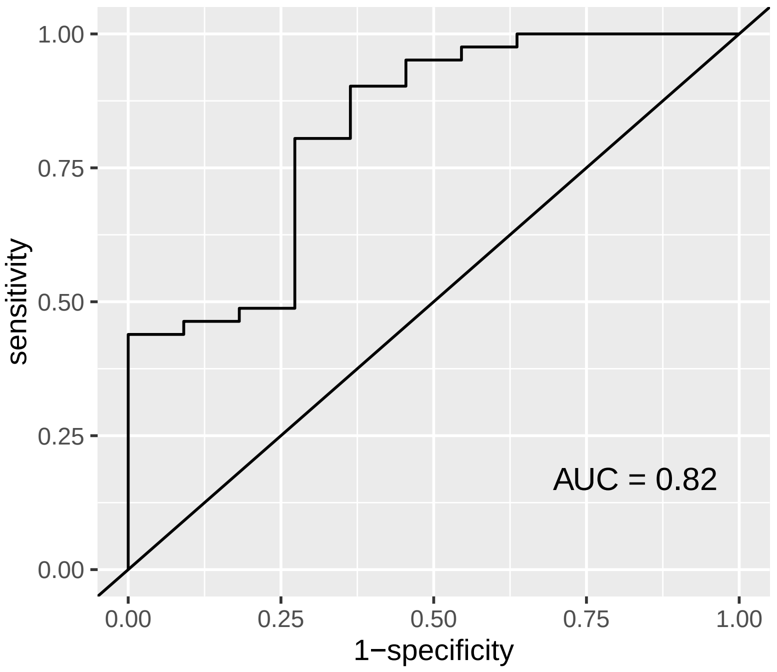

firstorder_Kurtosis

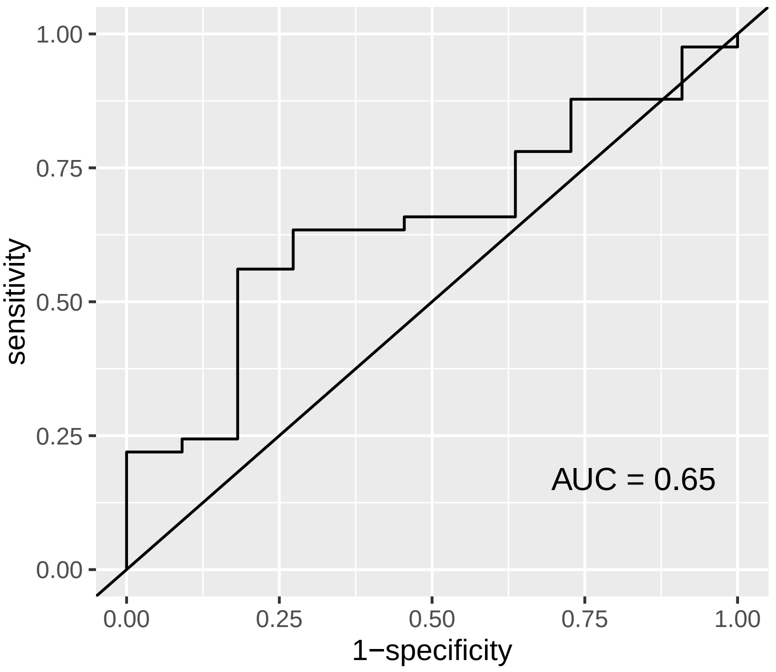

firstorder_Maximum

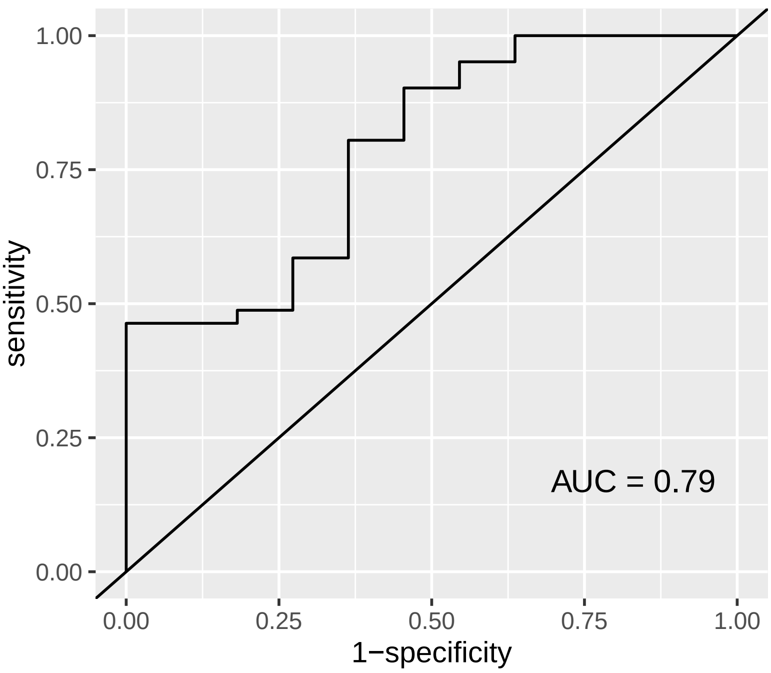

firstorder_Median

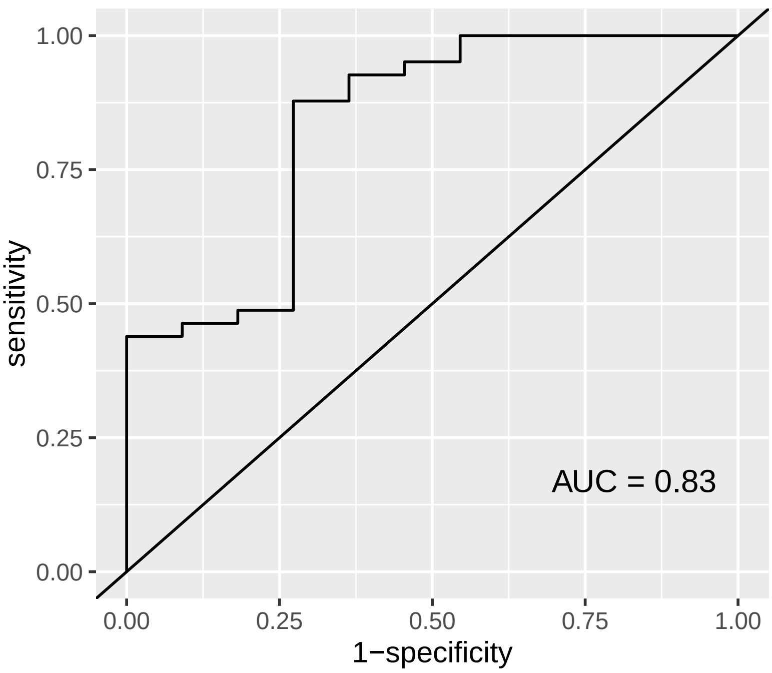

firstorder_Mean

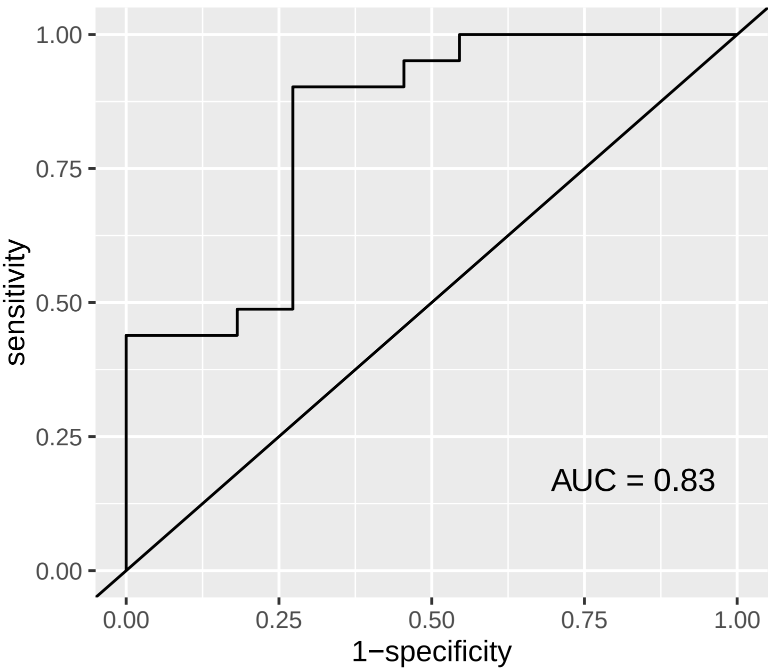

firstorder_Minimum

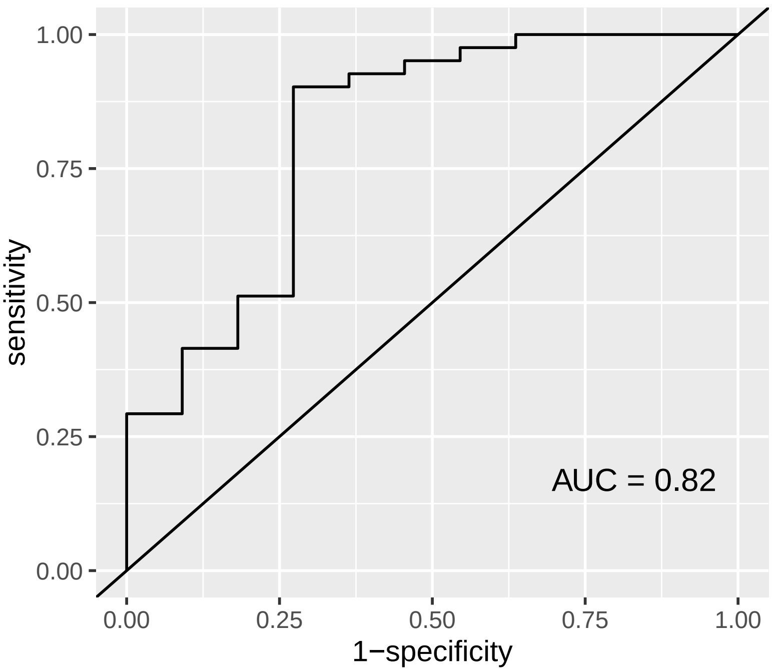

firstorder_MeanAbsoluteDeviation

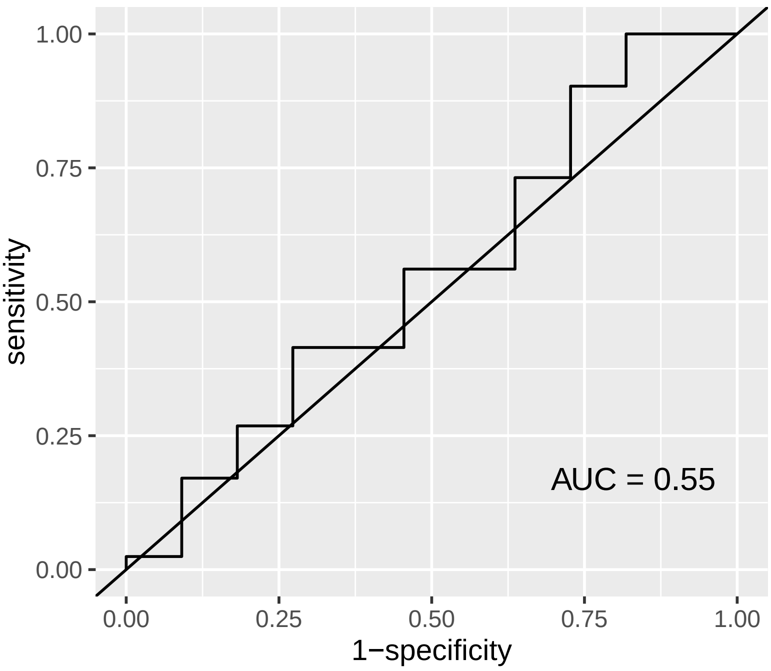

firstorder_Range

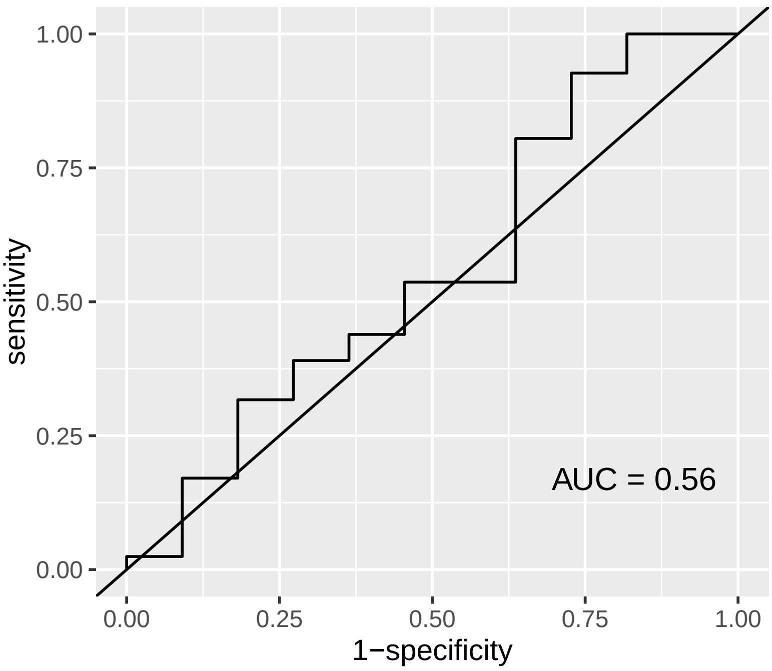

firstorder_RobustMeanAbsoluteDeviation

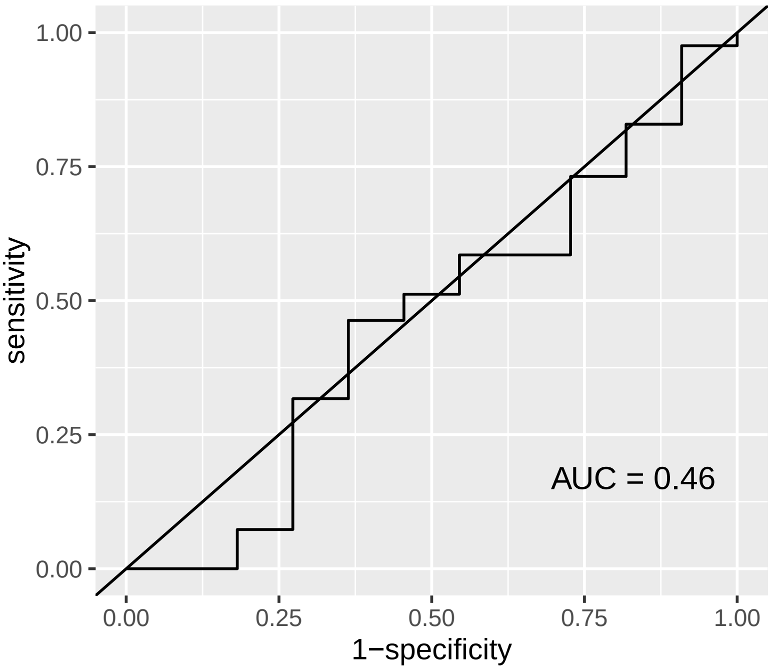

firstorder_TotalEnergy

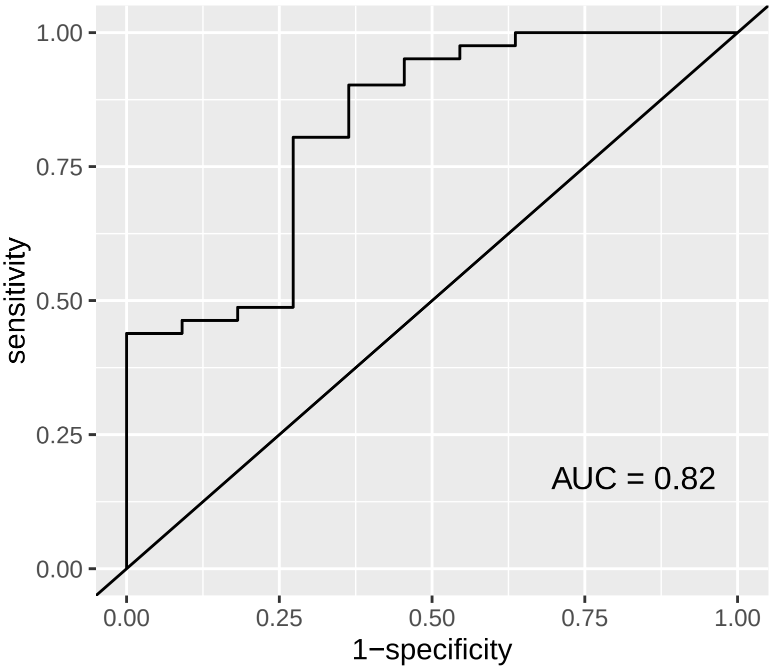

firstorder_RootMeanSquared

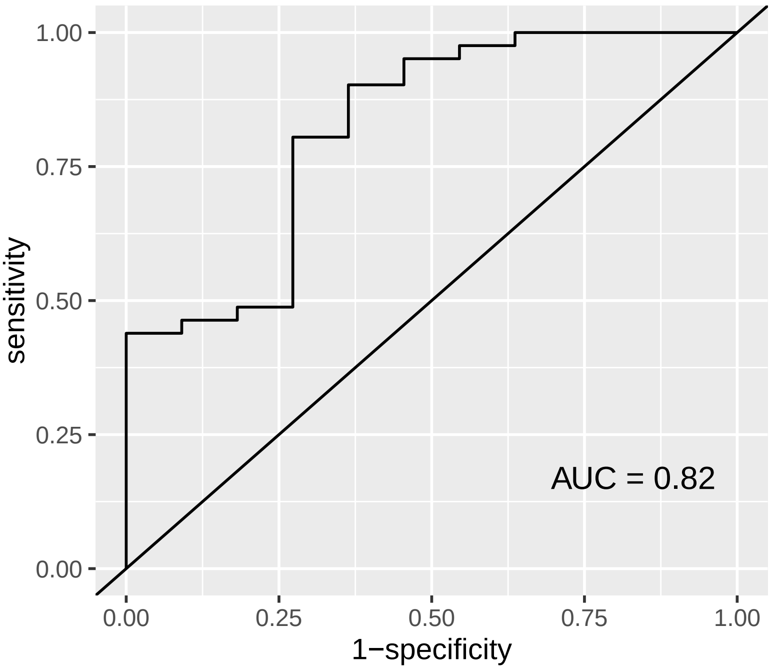

firstorder_Uniformity

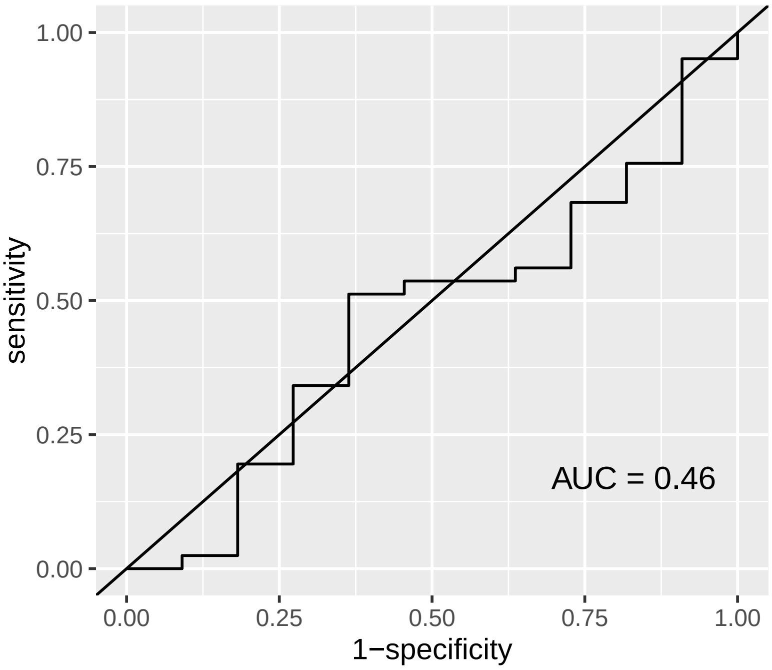

firstorder_Skewness

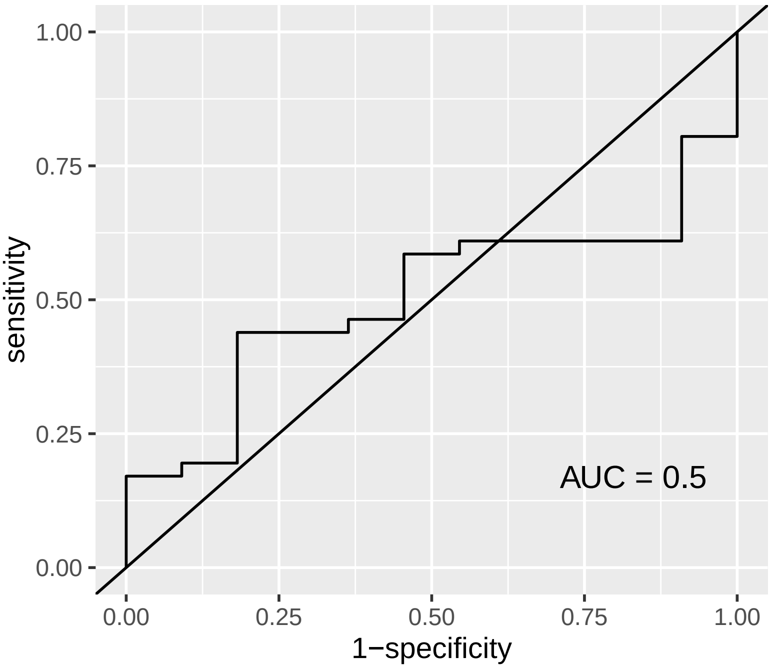

firstorder_Variance

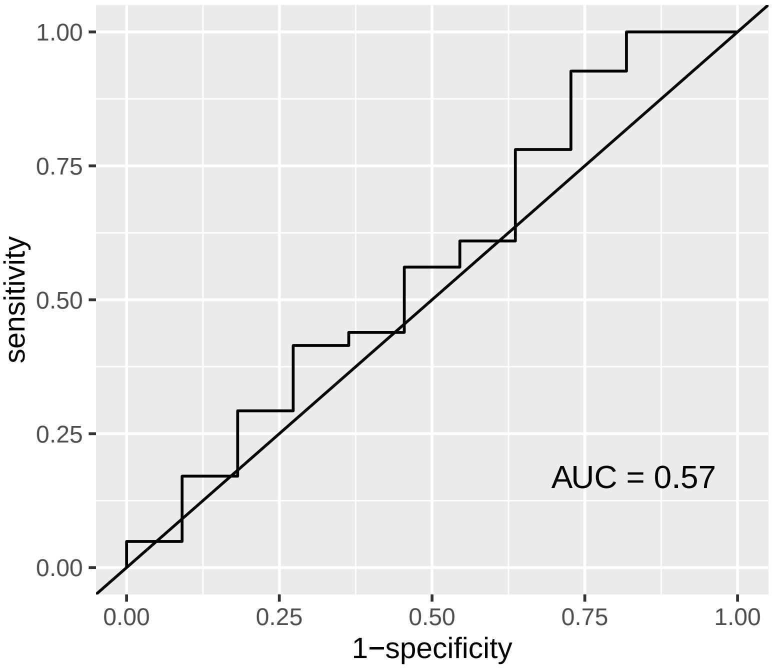

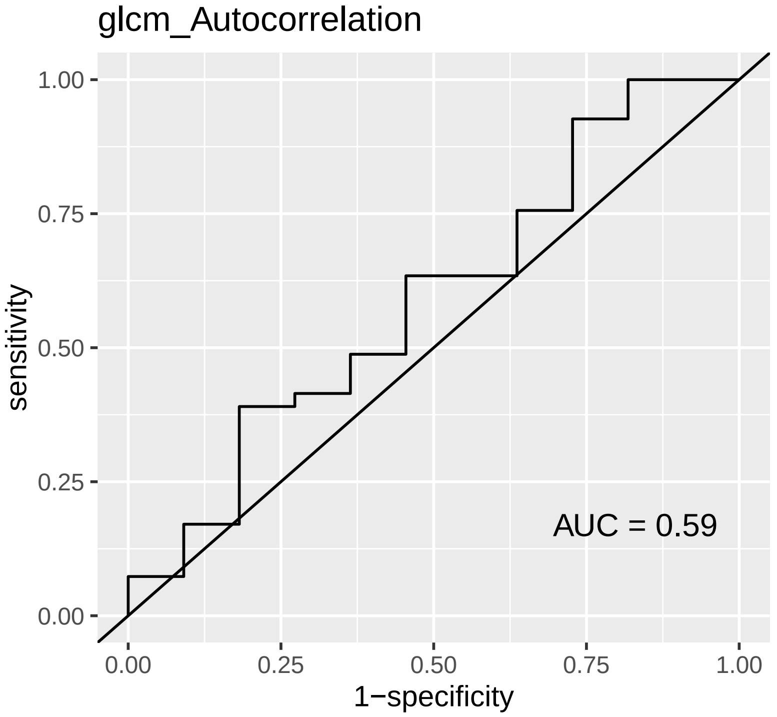
glcm_Autocorrelation
sensitivity
1−specificity
0.00
0.25
0.50
0.75
1.00
AUC = 0.59

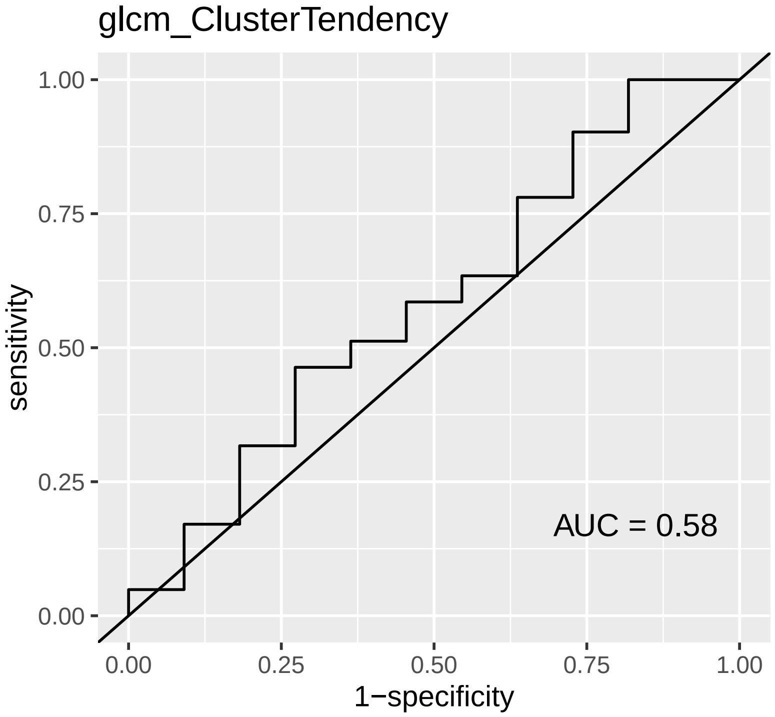
glcm_ClusterTendency
sensitivity
1−specificity
0.00
0.25
0.50
0.75
1.00
AUC = 0.58

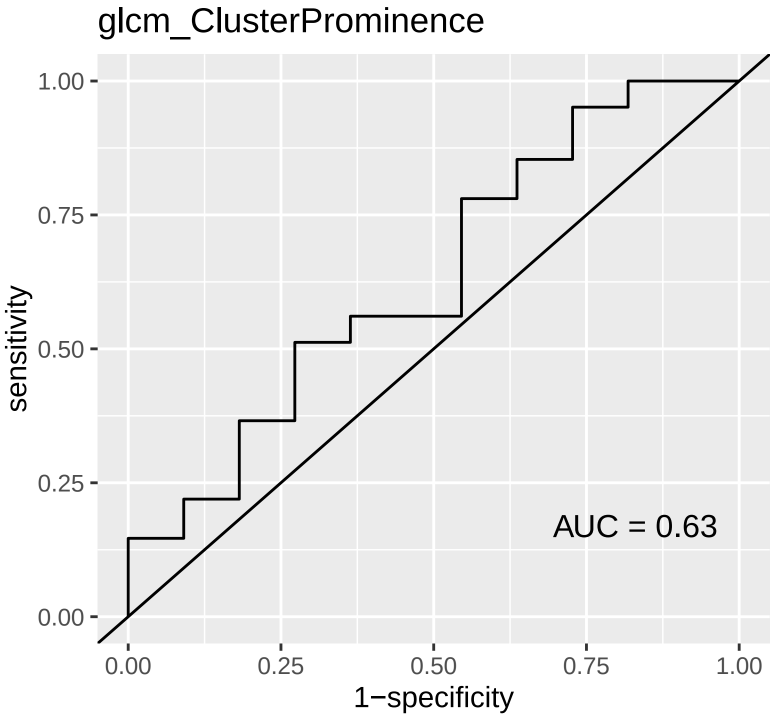
glcm_ClusterProminence
sensitivity
1−specificity
0.00
0.25
0.50
0.75
1.00
AUC = 0.63

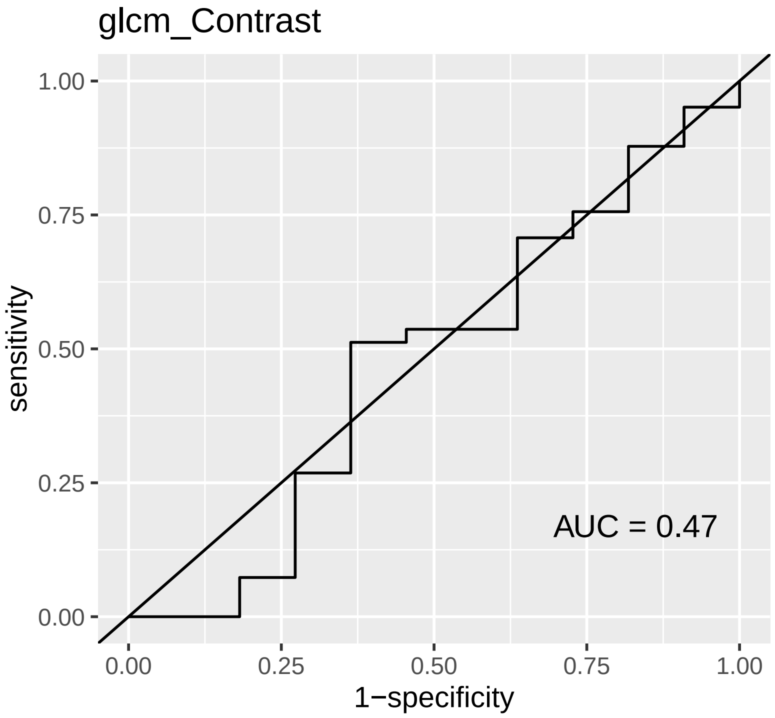
glcm_Contrast
sensitivity
1−specificity
0.00
0.25
0.50
0.75
1.00
AUC = 0.47

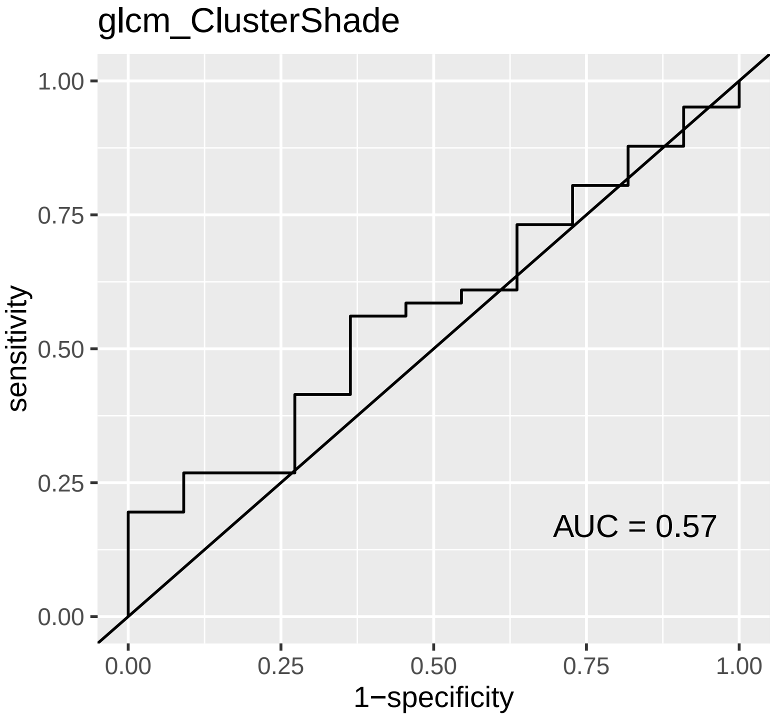
glcm_ClusterShade
sensitivity
1−specificity
0.00
0.25
0.50
0.75
1.00
AUC = 0.57

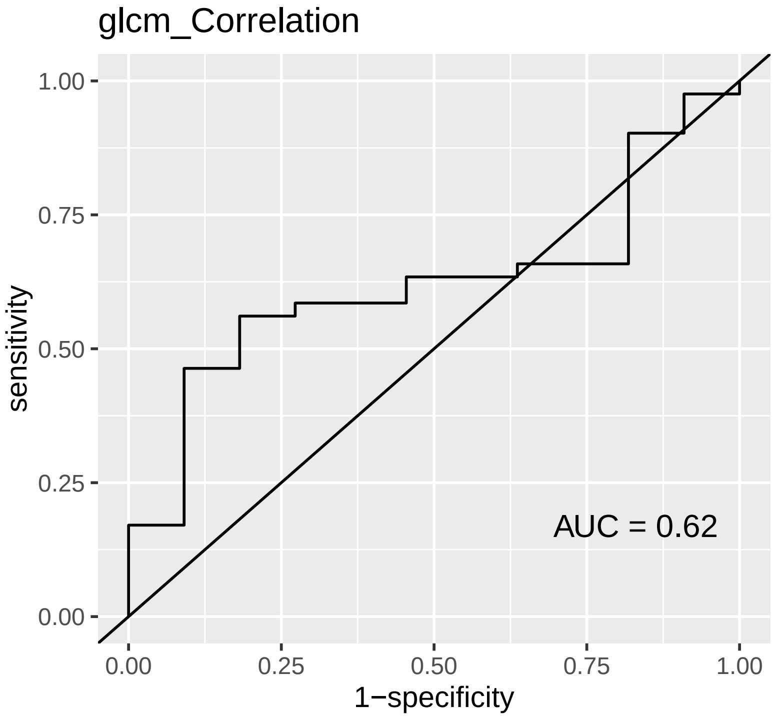
glcm_Correlation
sensitivity
1−specificity
0.00
0.25
0.50
0.75
1.00
AUC = 0.62

glcm_DifferenceAverage

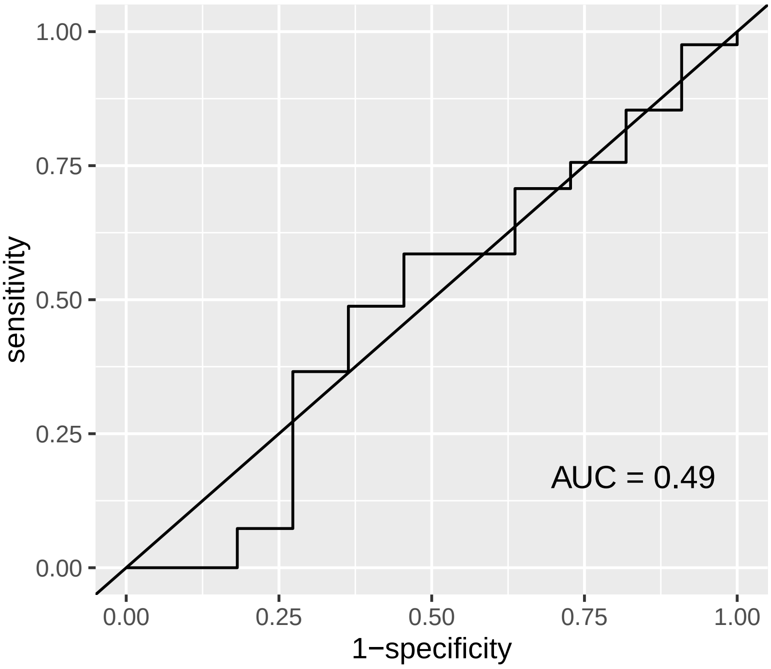

glcm_Id

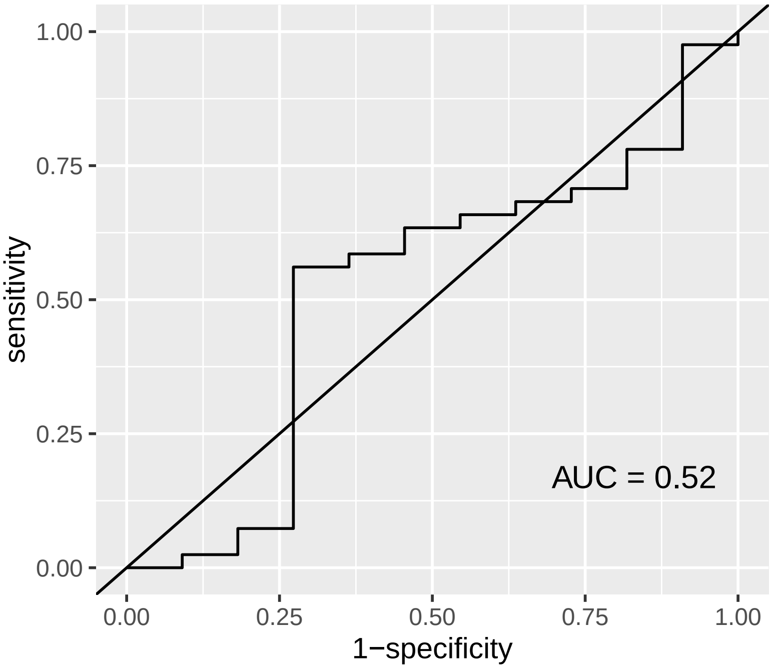

glcm_DifferenceEntropy

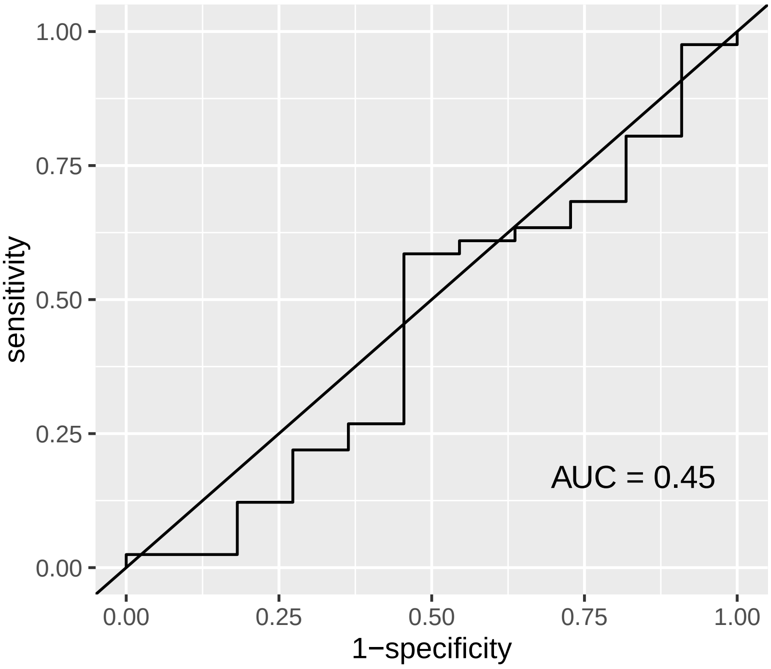

glcm_Idm

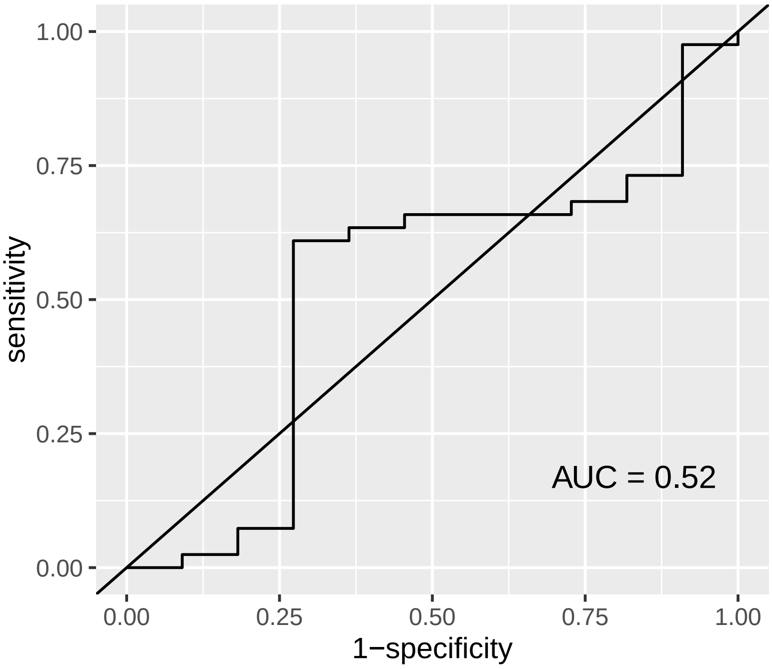

glcm_DifferenceVariance

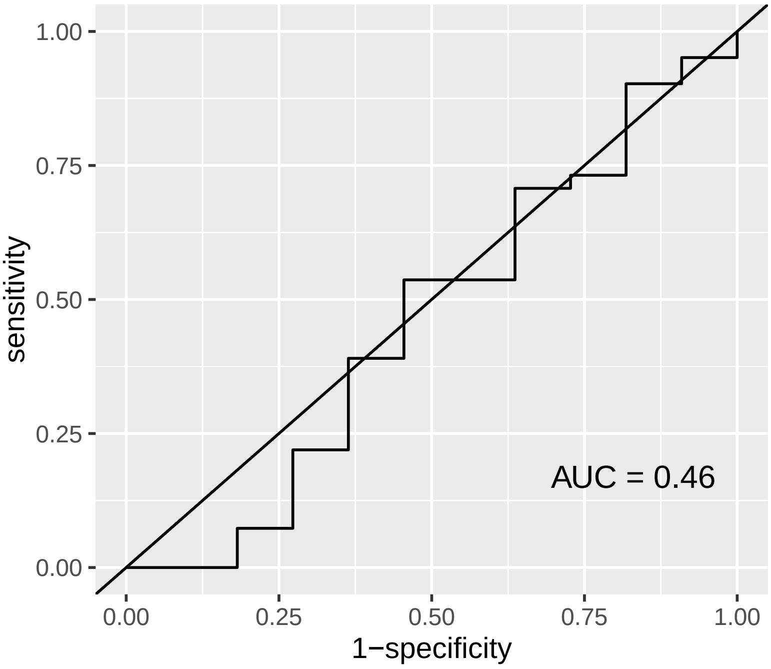

glcm_Idmn

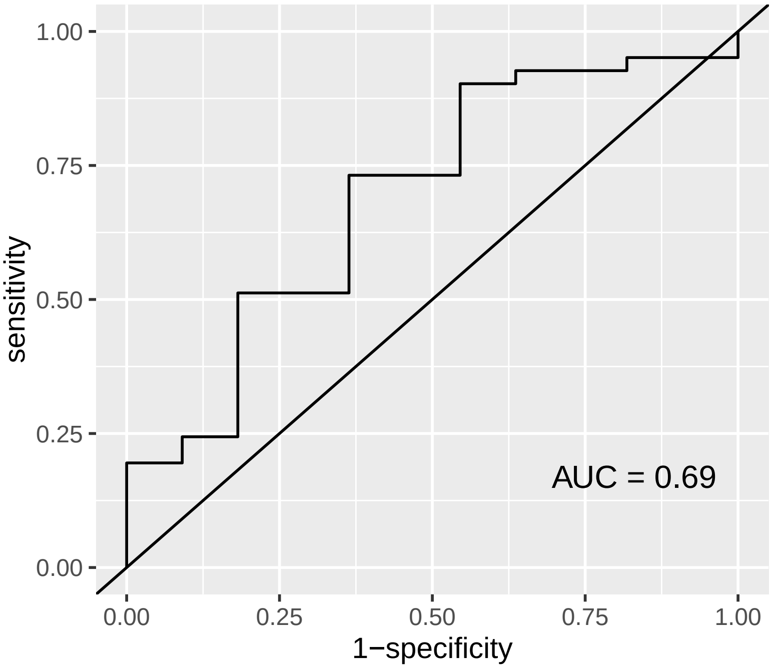

glcm_Idn

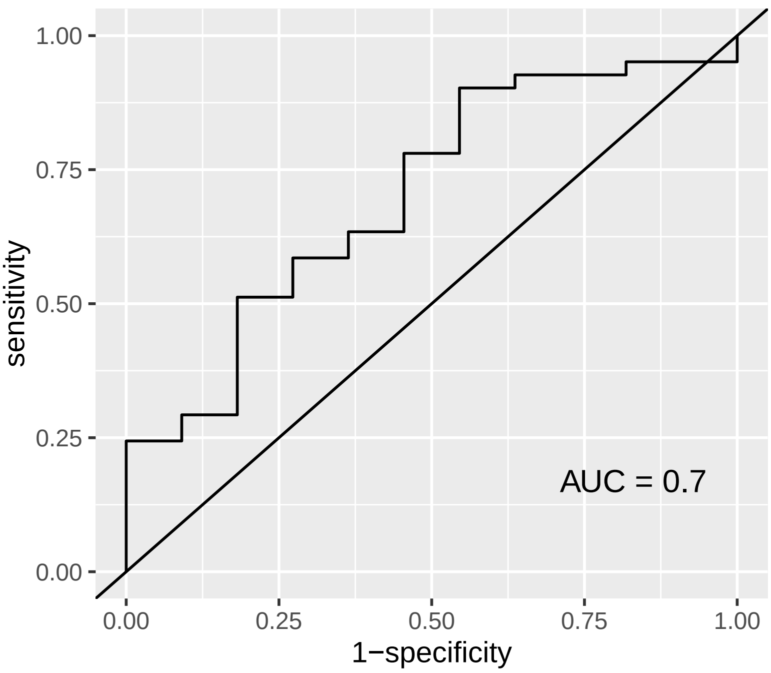

glcm_InverseVariance

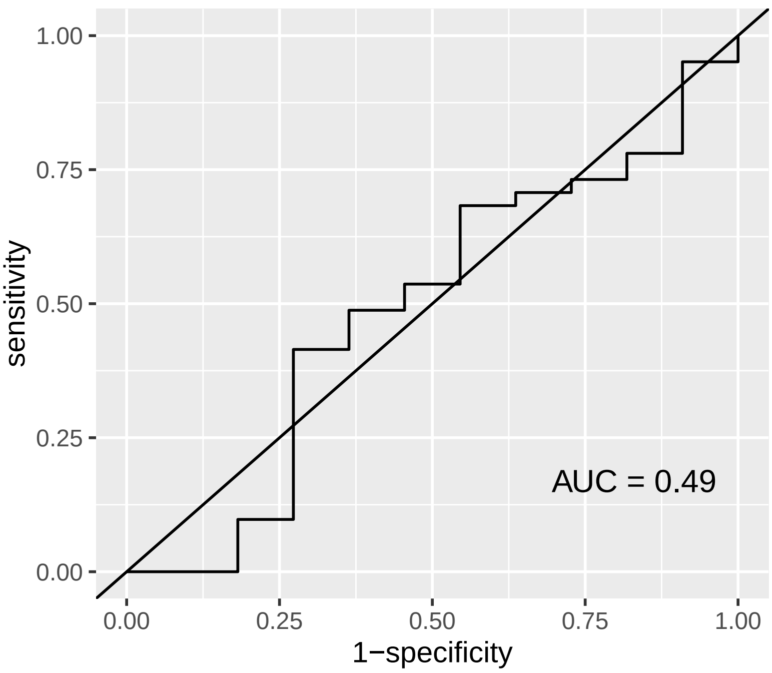

glcm_Imc1

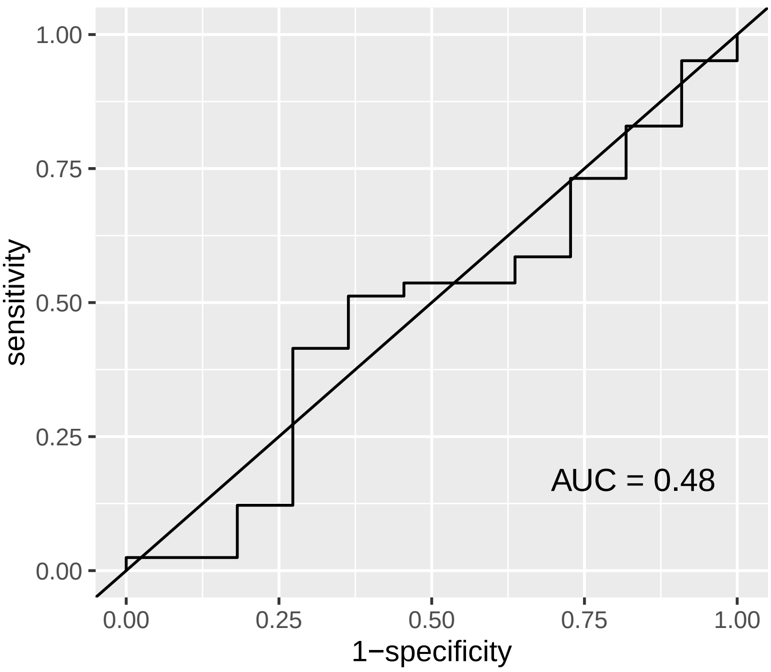

glcm_JointAverage

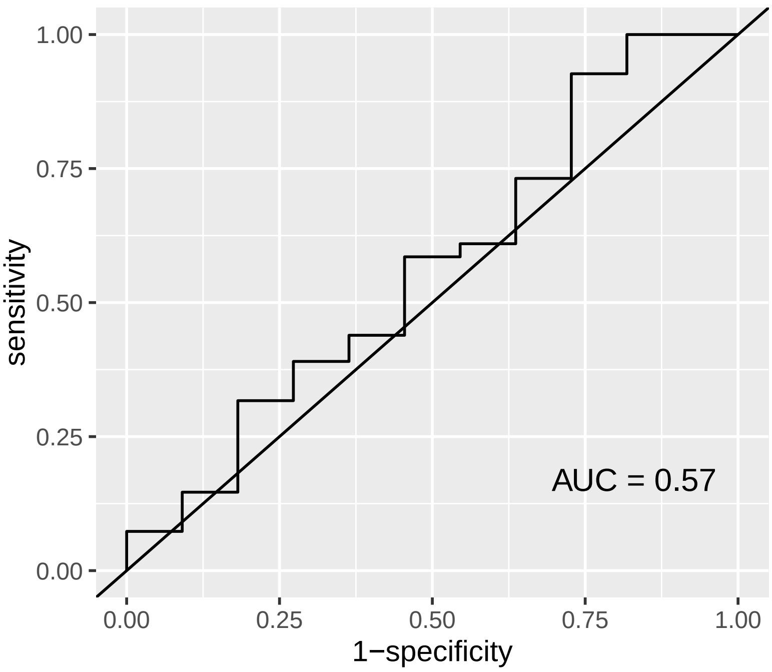

glcm_Imc2

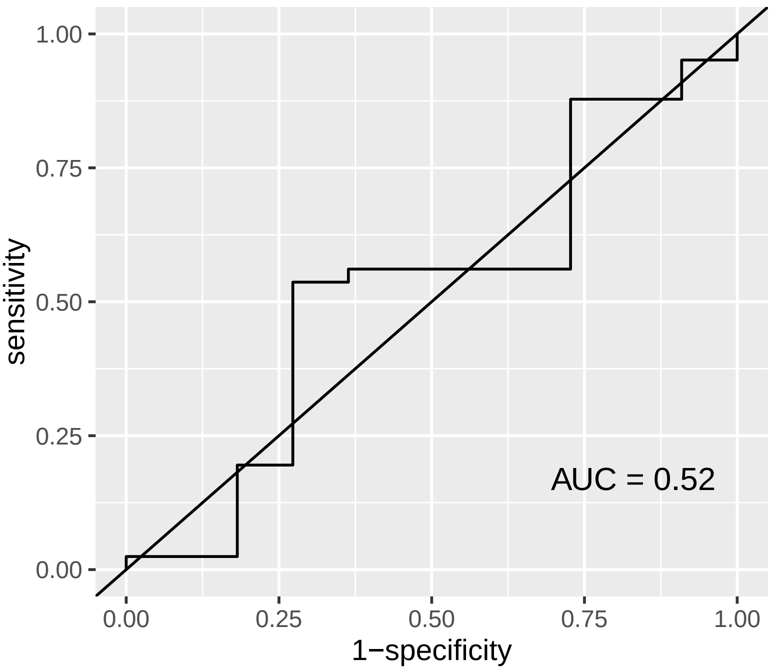

glcm_JointEnergy

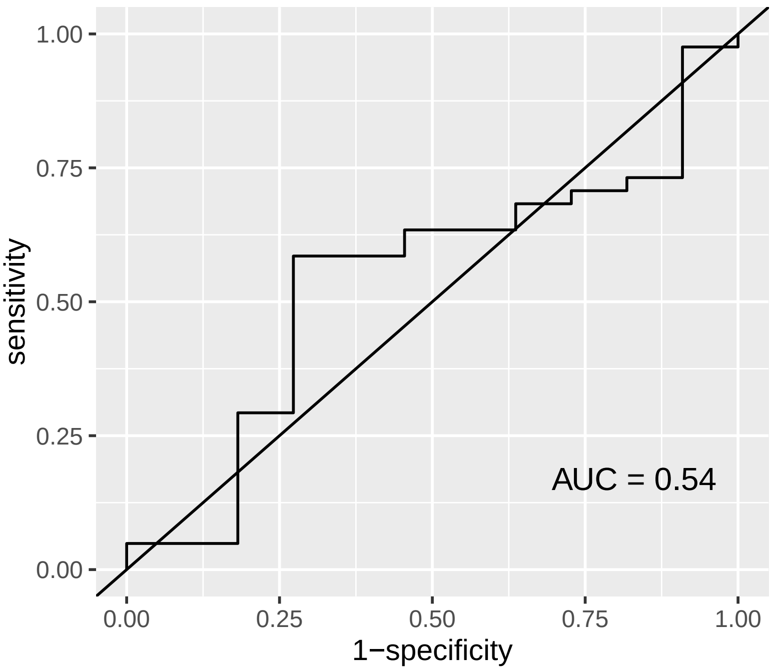

glcm_JointEntropy

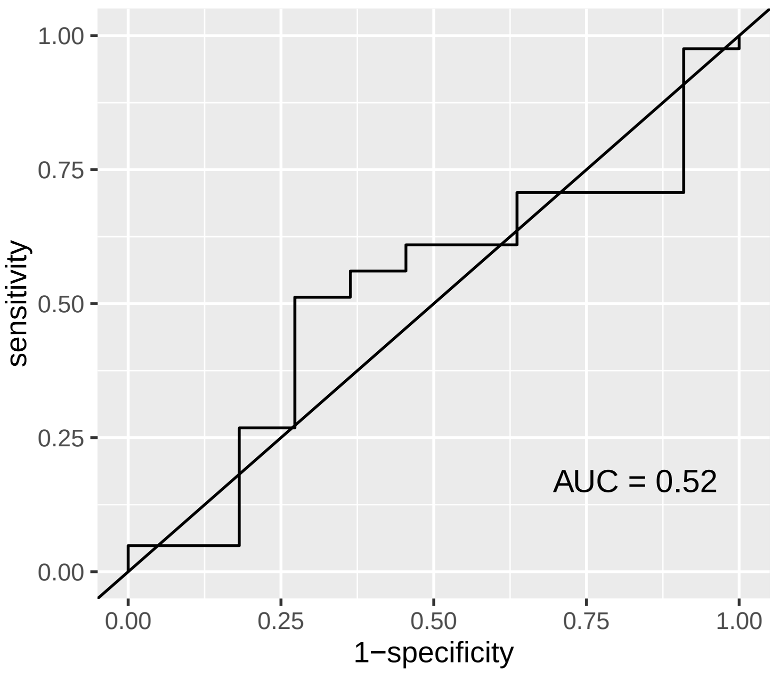

glcm_SumAverage

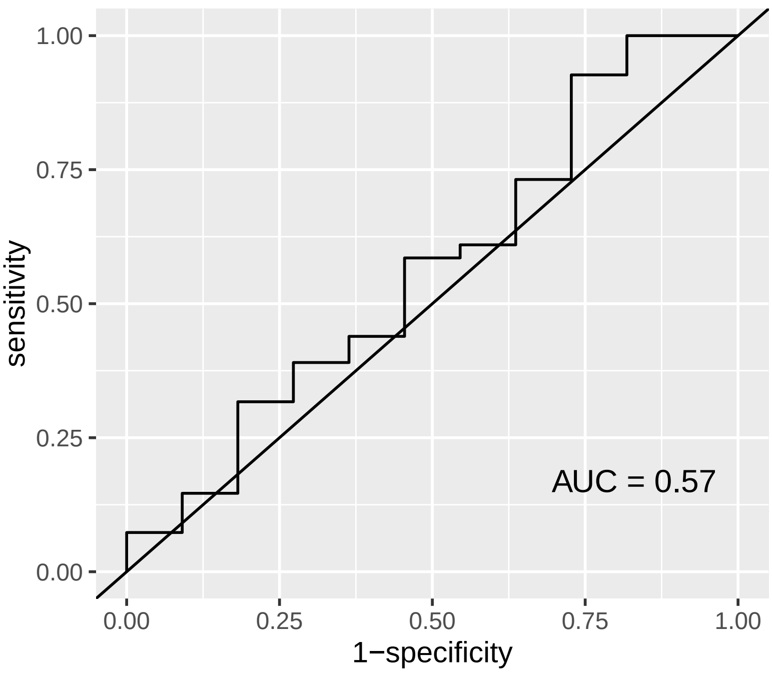

glcm_MaximumProbability

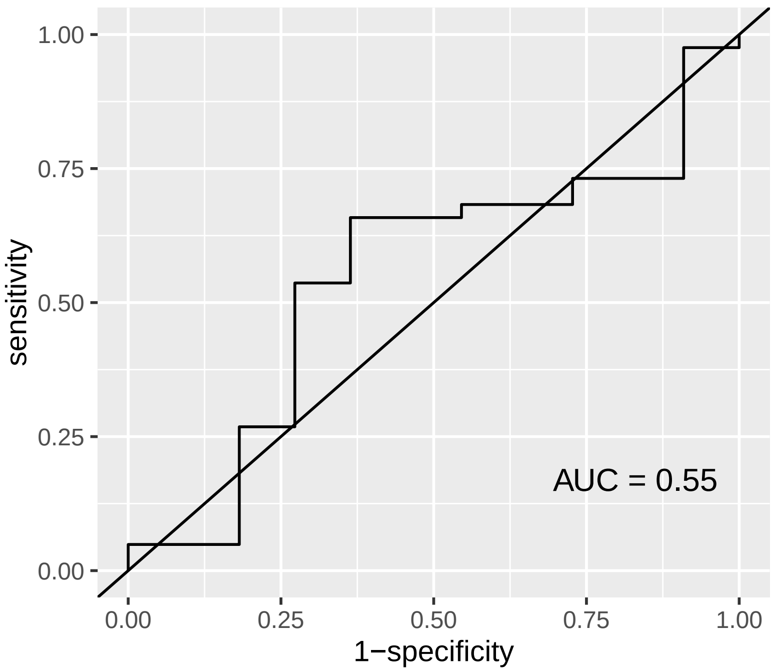

glcm_SumEntropy

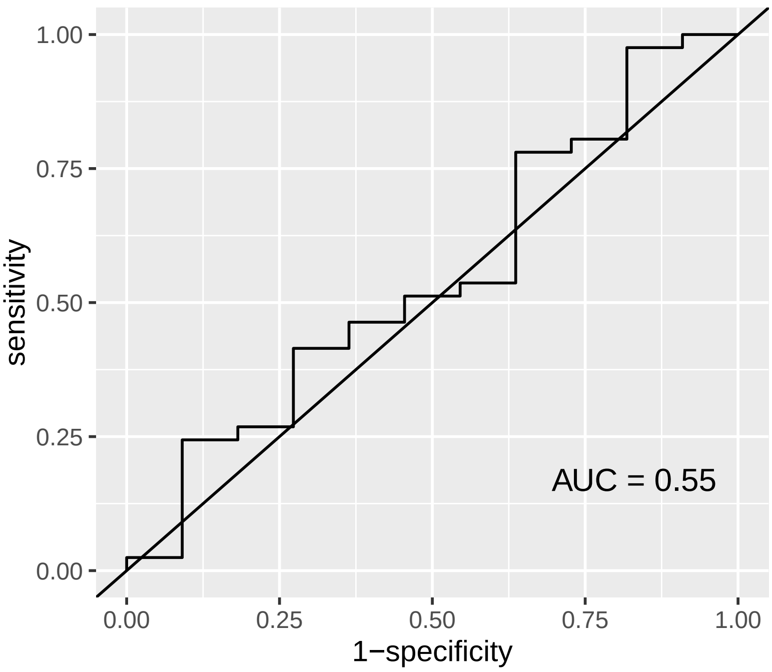

glcm_MCC

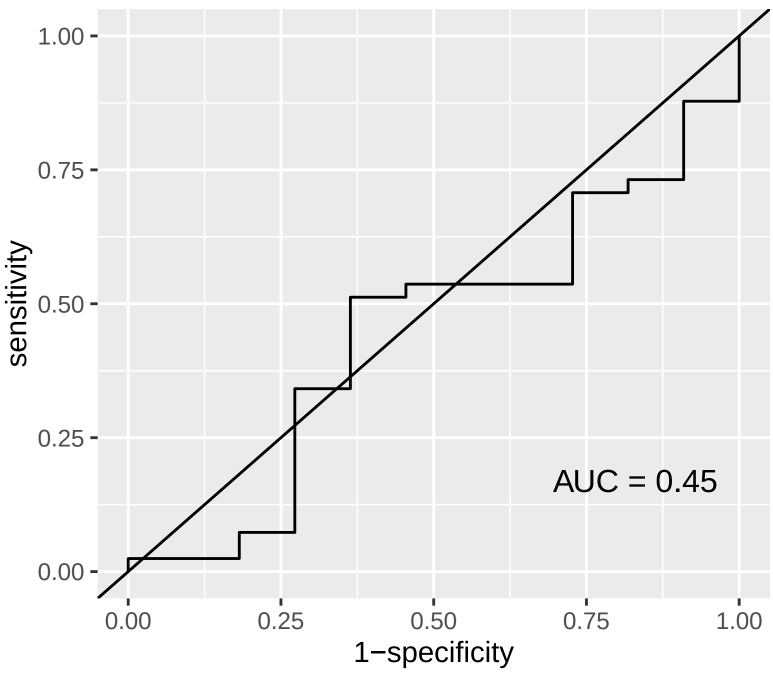

glcm_SumSquares

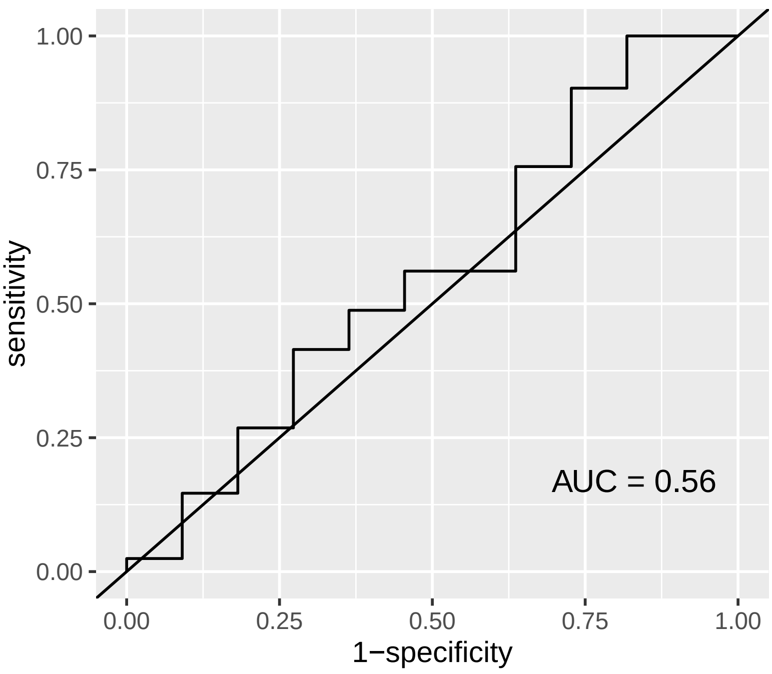

gldm_DependenceEntropy

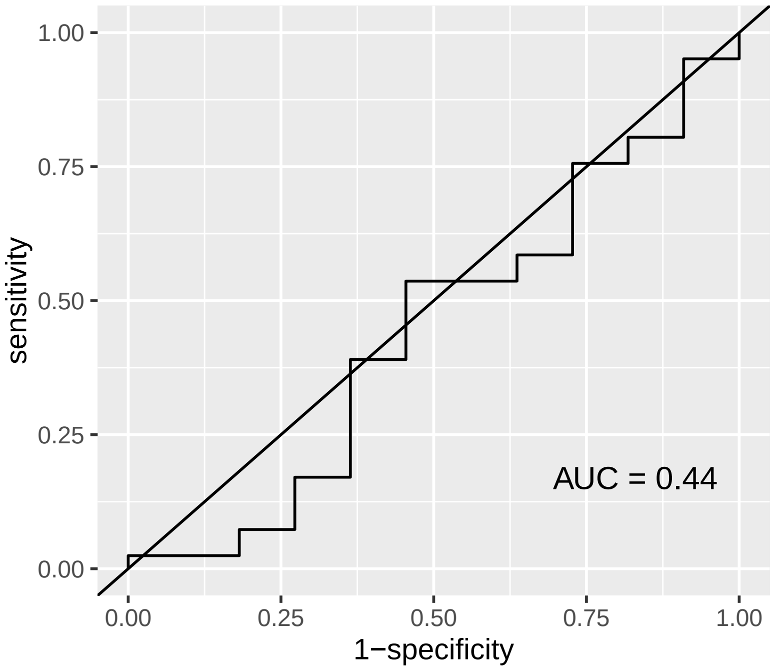

gldm_DependenceVariance

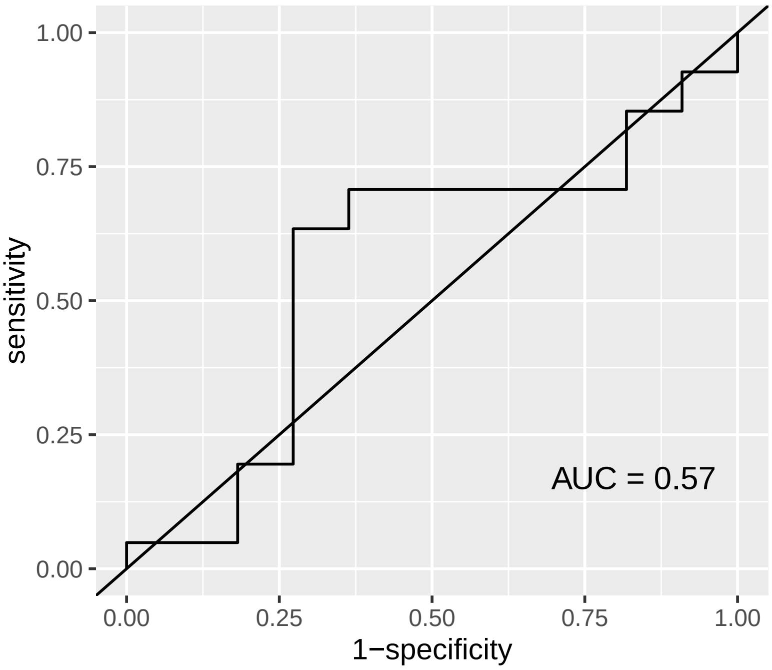

gldm_DependenceNonUniformity

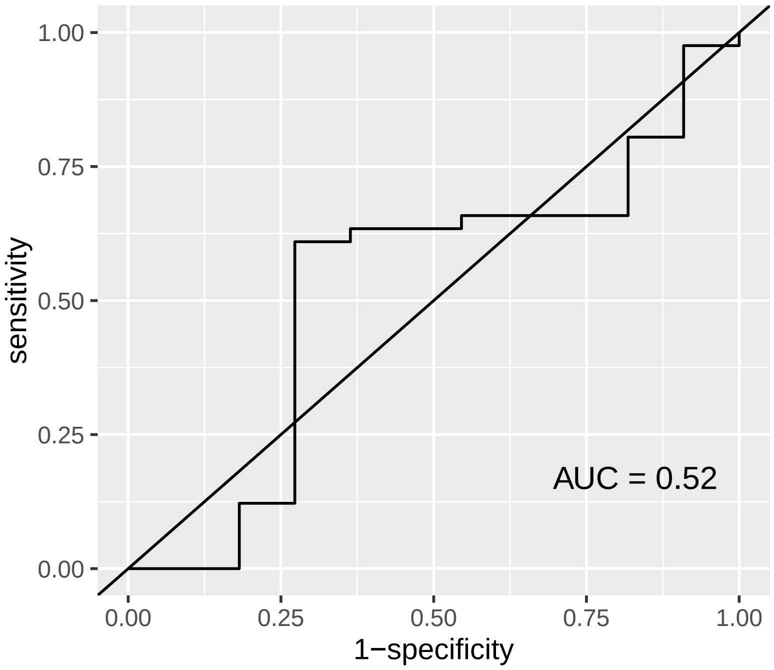

gldm_GrayLevelNonUniformity

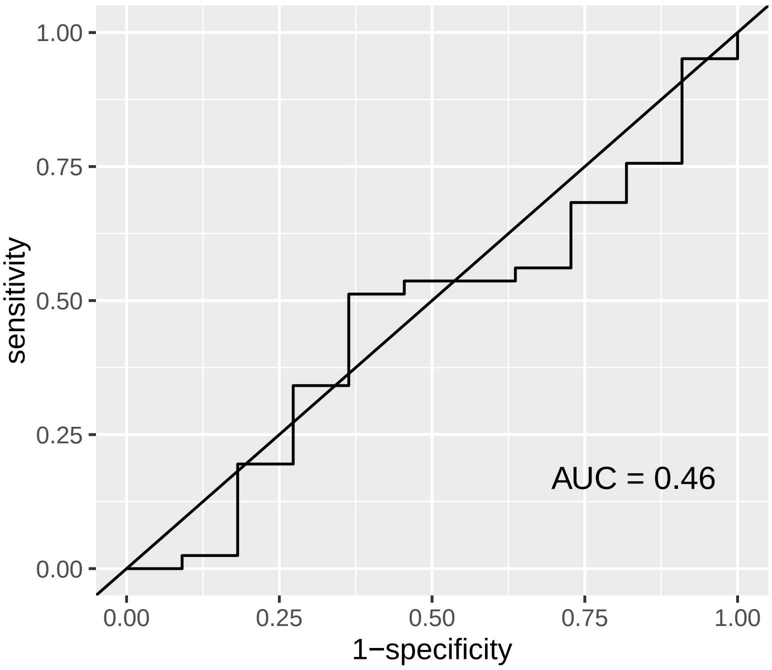

gldm_DependenceNonUniformityNormalized

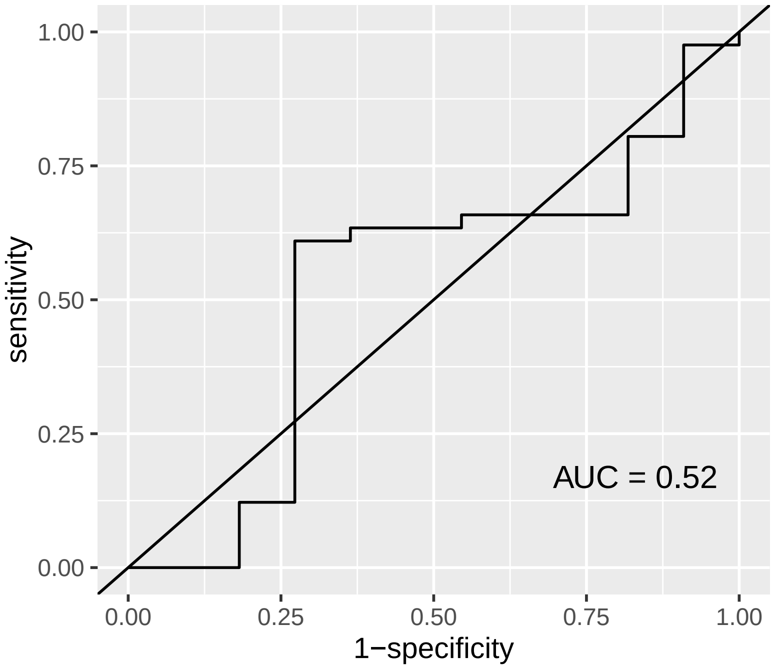

gldm_GrayLevelVariance

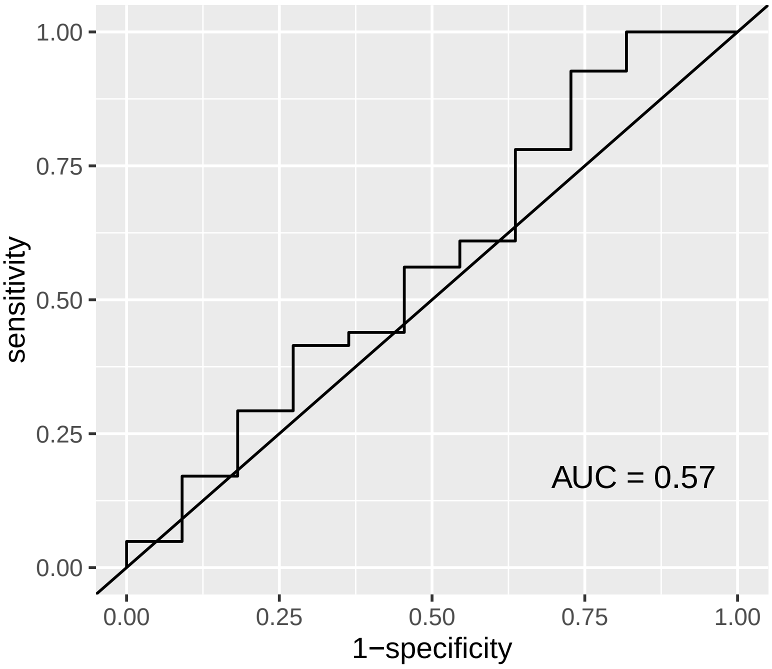

gldm_HighGrayLevelEmphasis

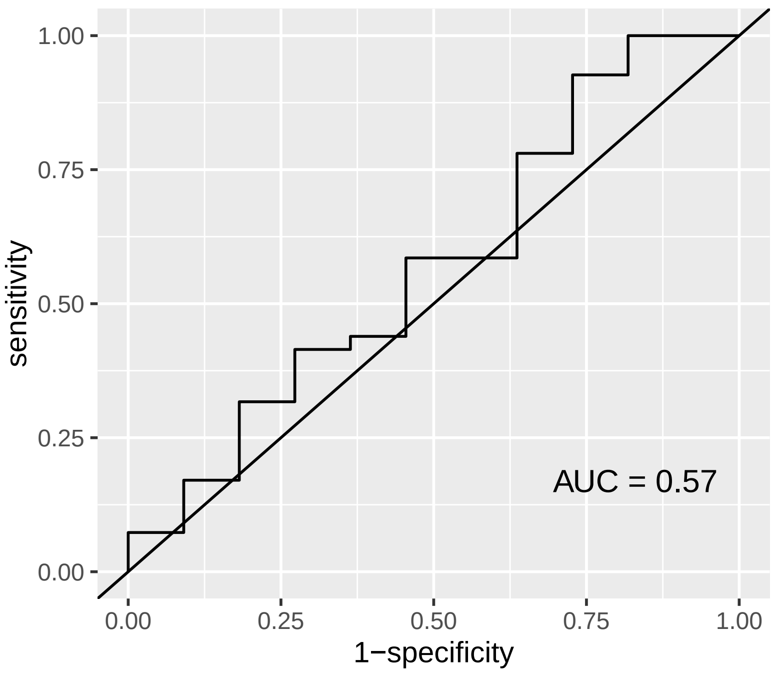

gldm_LargeDependenceLowGrayLevelEmph

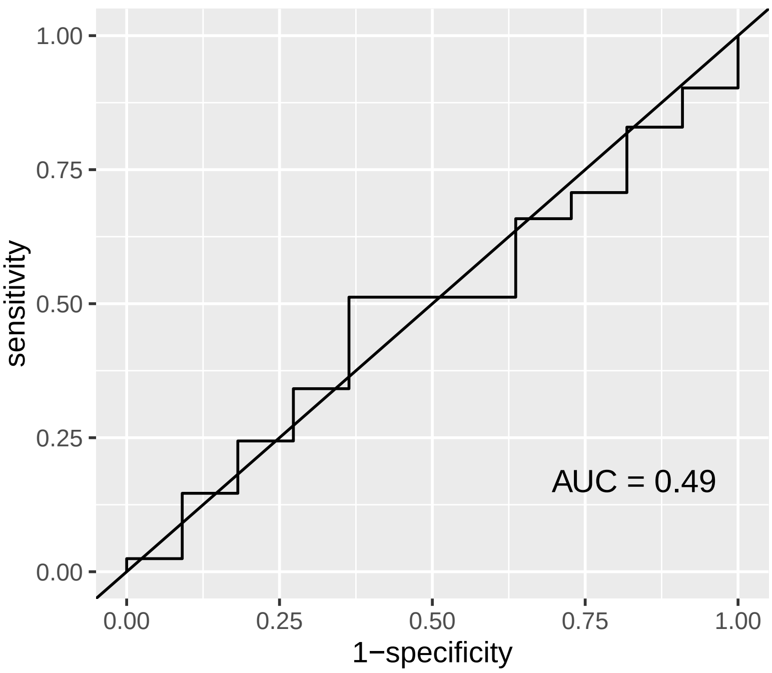

gldm_LargeDependenceEmphasis

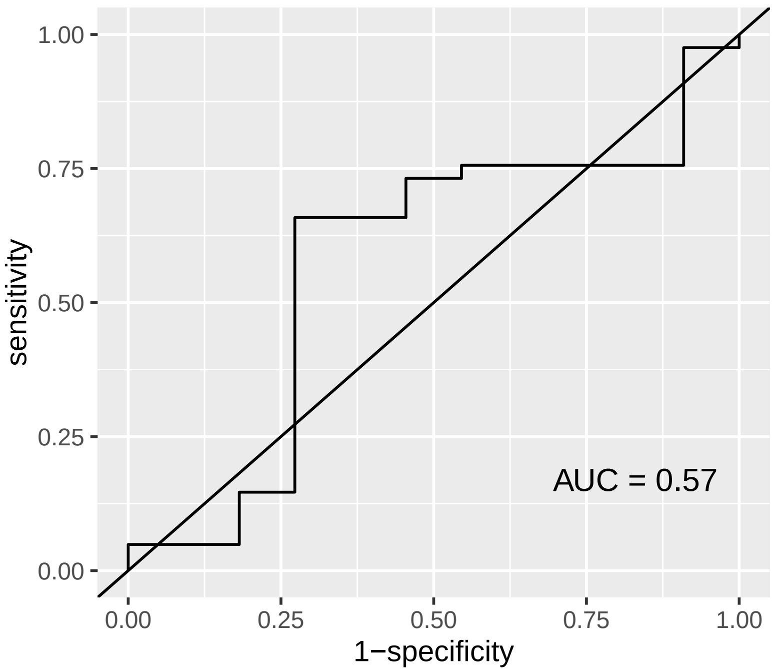

gldm_LowGrayLevelEmphasis

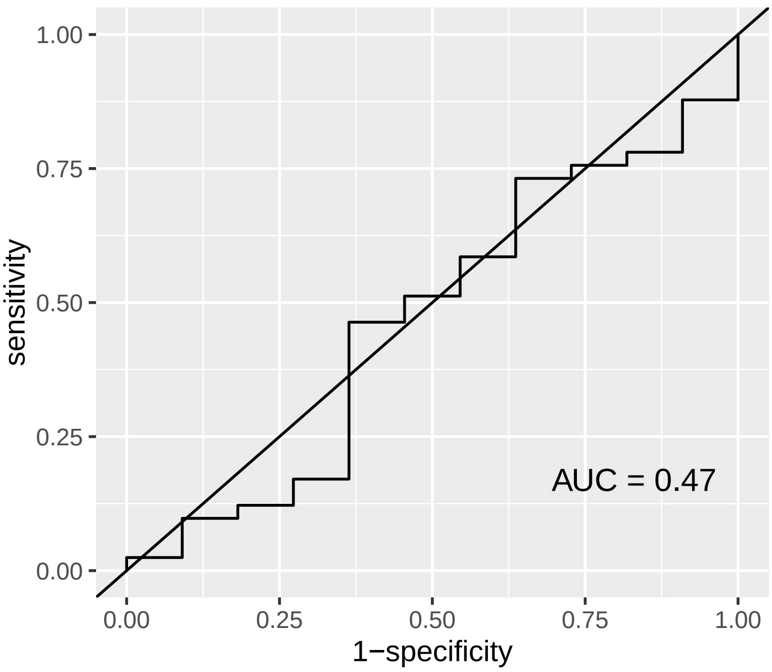

gldm_LargeDependenceHighGrayLevelEmph

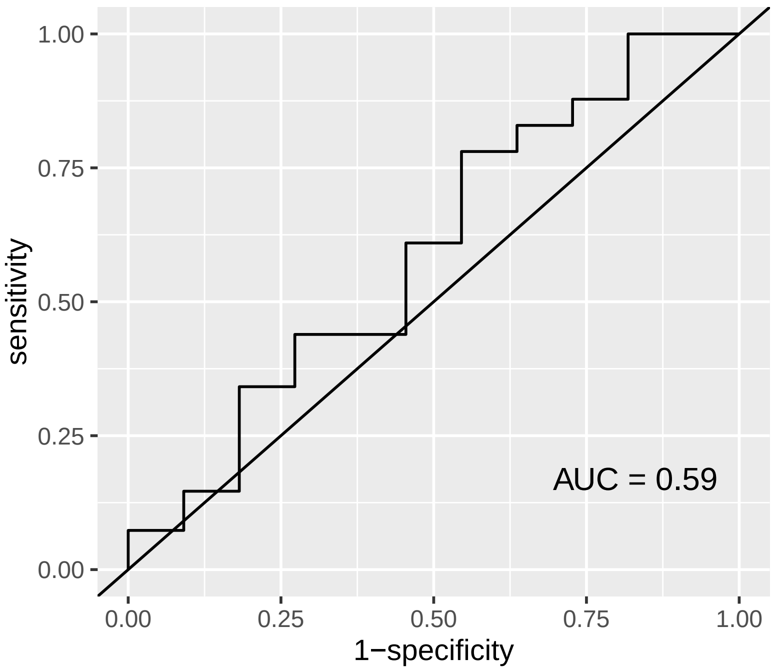

gldm_SmallDependenceEmphasis

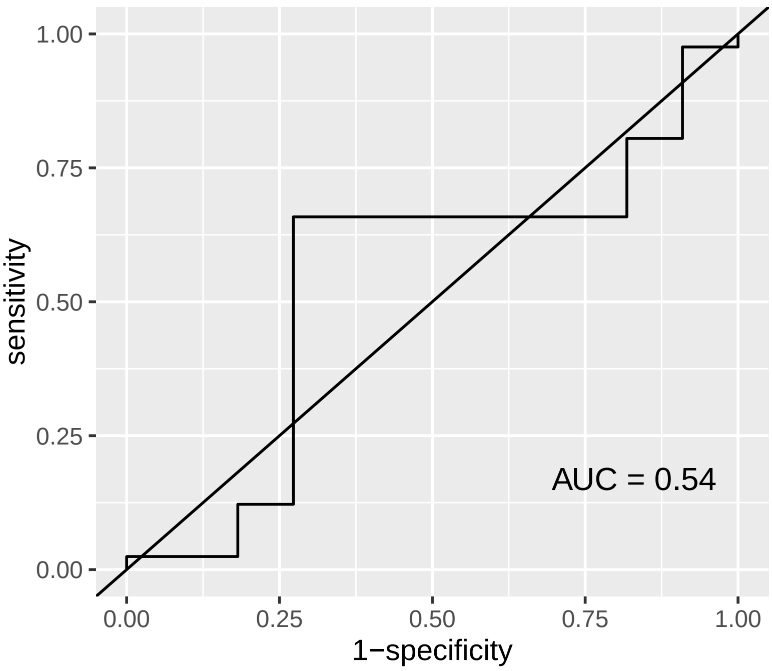

gldm_SmallDependenceHighGrayLevelEmph

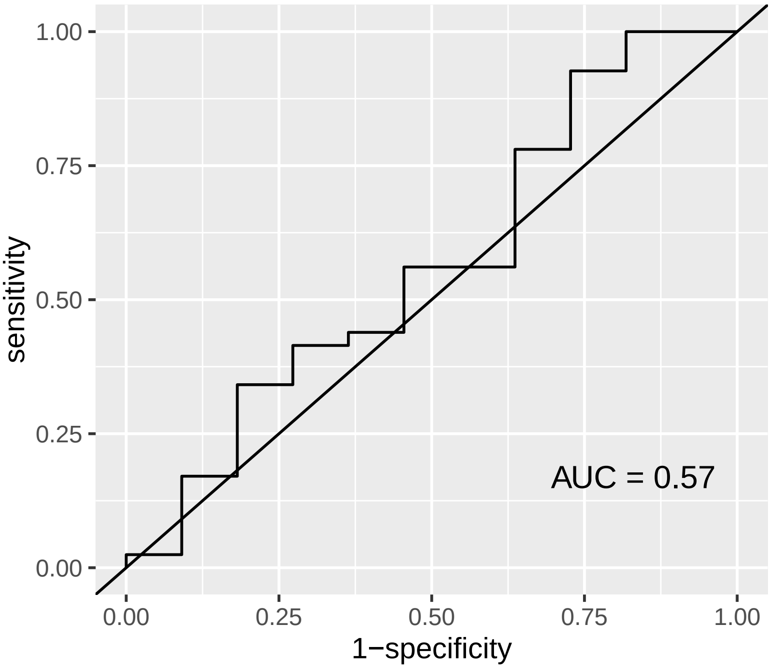

glrlm_GrayLevelNonUniformityNormalized

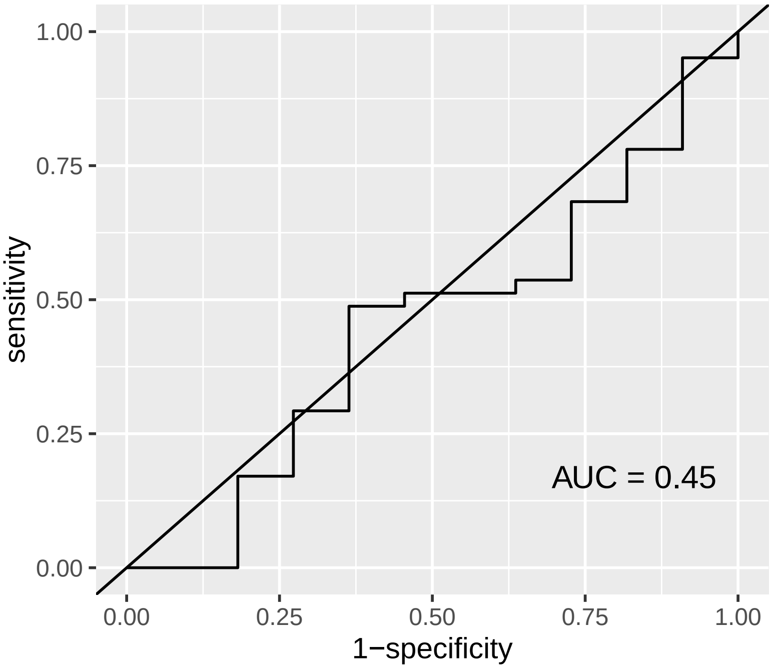

gldm_SmallDependenceLowGrayLevelEmph

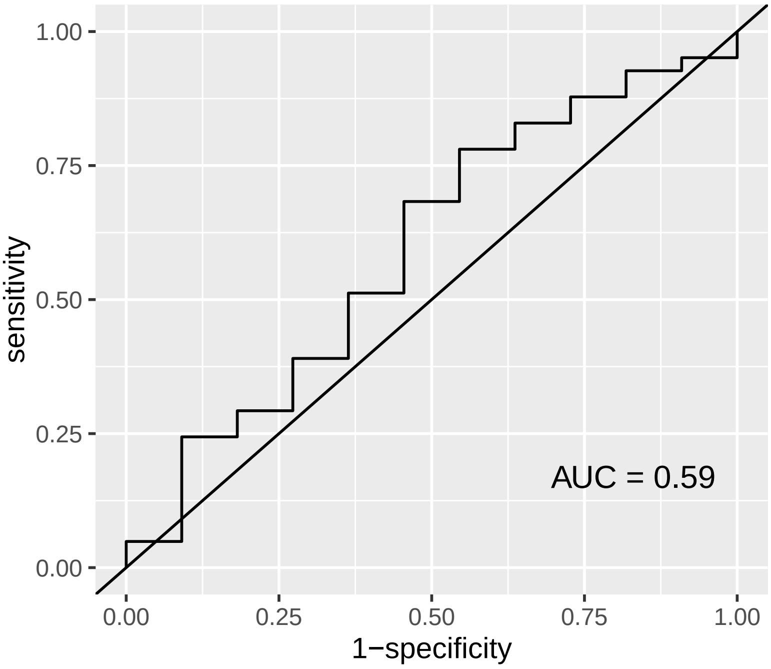

glrlm_GrayLevelVariance

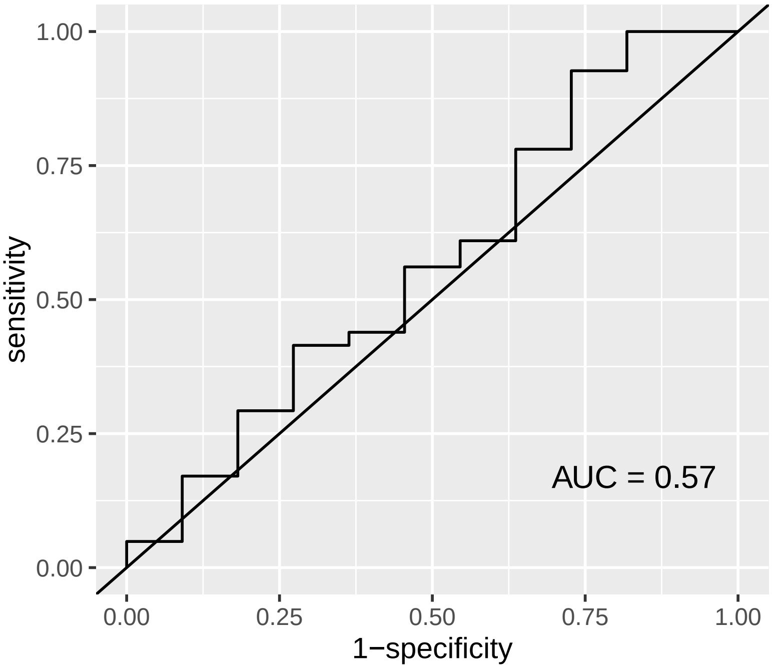

glrlm_GrayLevelNonUniformity

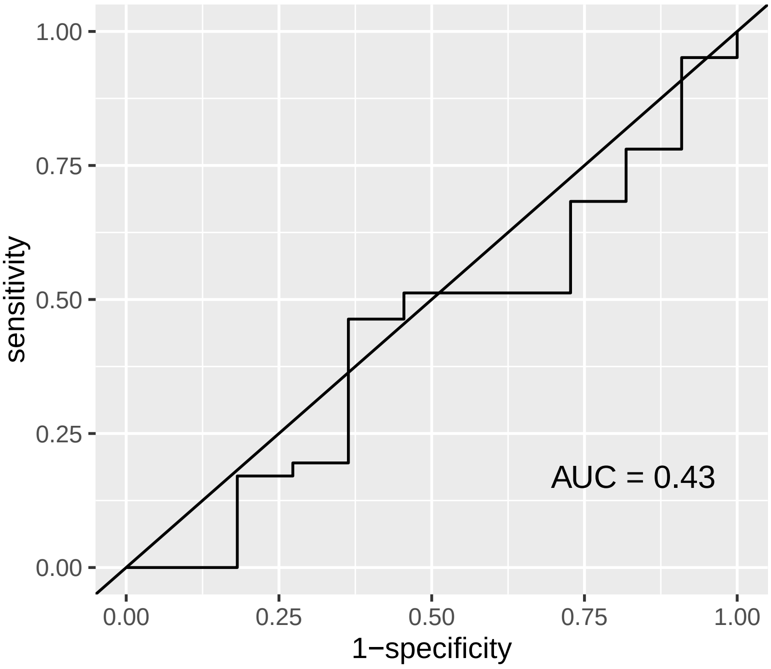

glrlm_HighGrayLevelRunEmphasis

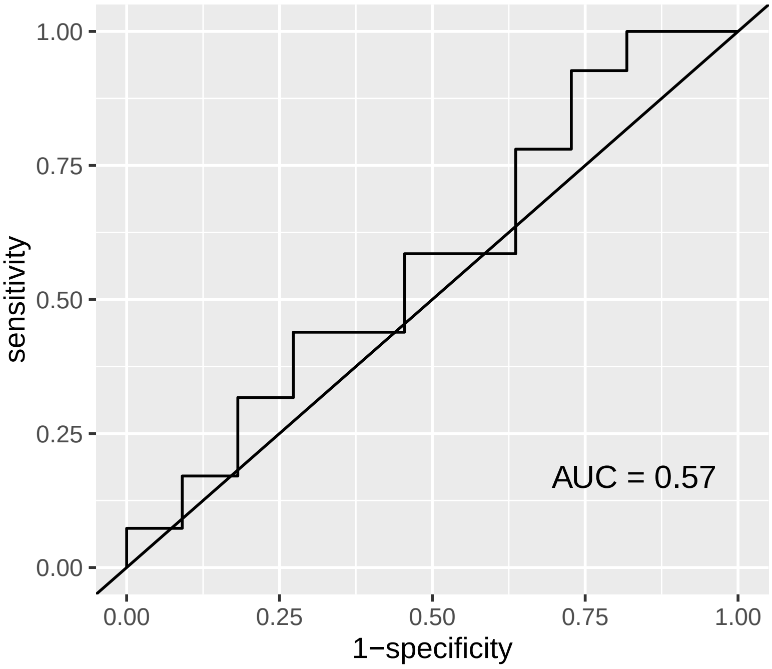

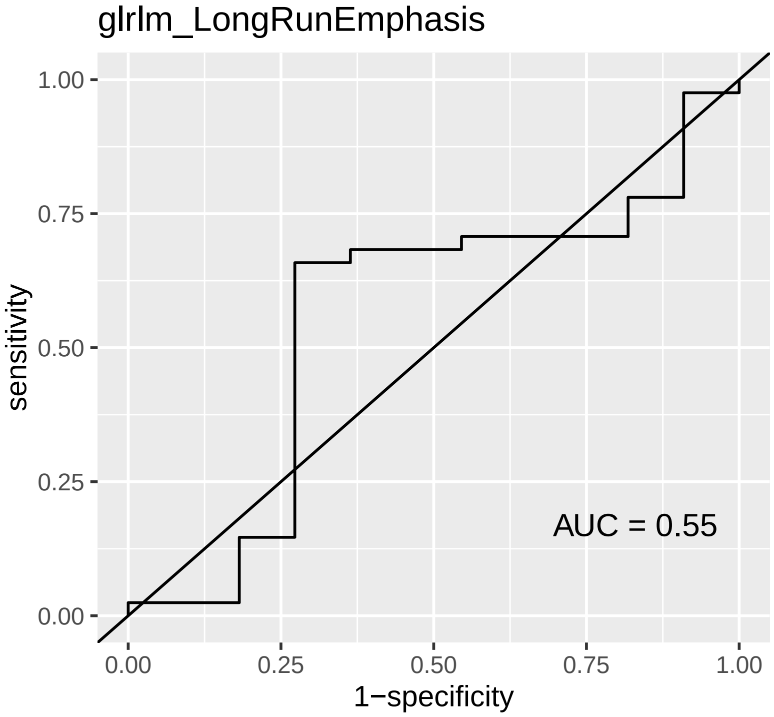
glrlm_LongRunEmphasis
AUC = 0.55
sensitivity
1−specificity
0.00
0.25
0.50
0.75
1.00

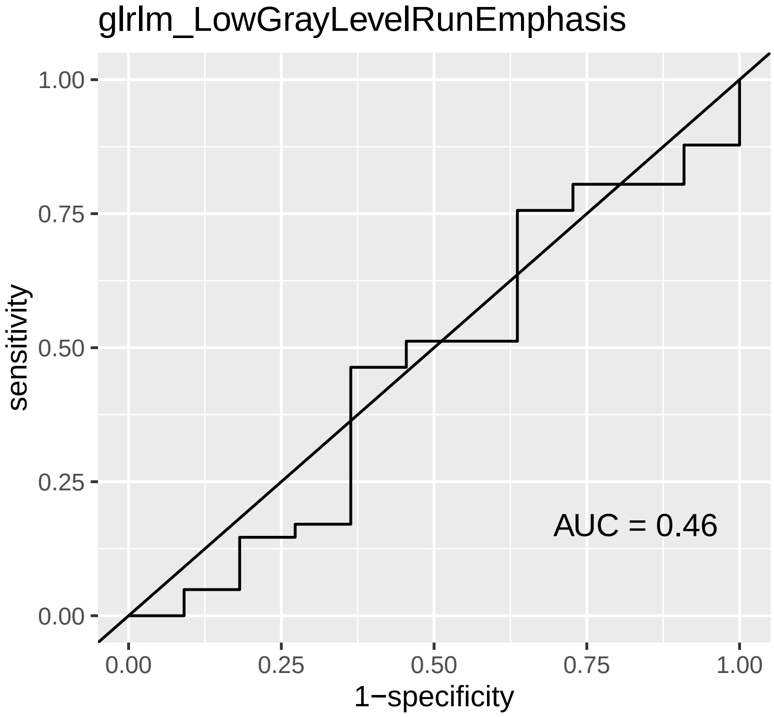
glrlm_LowGrayLevelRunEmphasis
AUC = 0.46
sensitivity
1−specificity
0.00
0.25
0.50
0.75
1.00

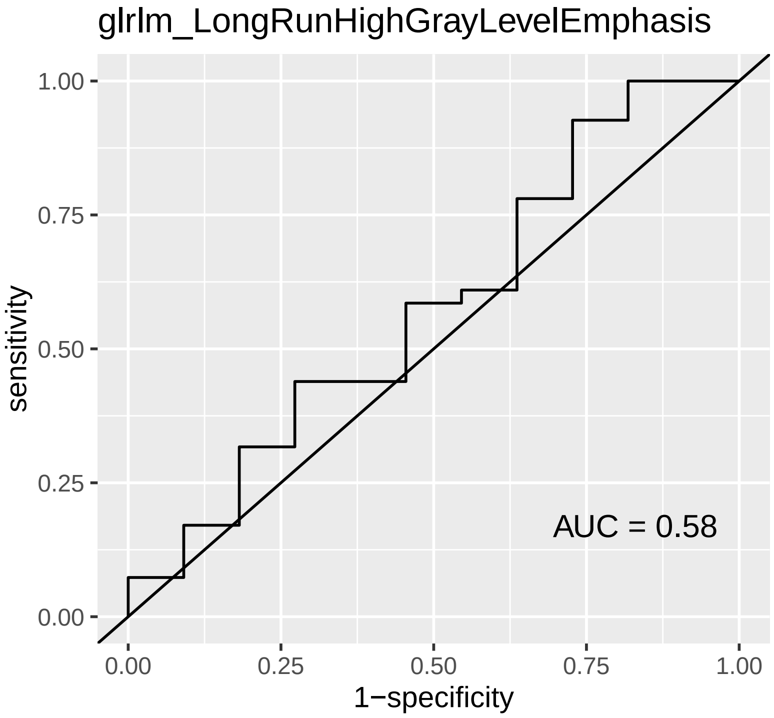
glrlm_LongRunHighGrayLevelEmphasis
AUC = 0.58
sensitivity
1−specificity
0.00
0.25
0.50
0.75
1.00

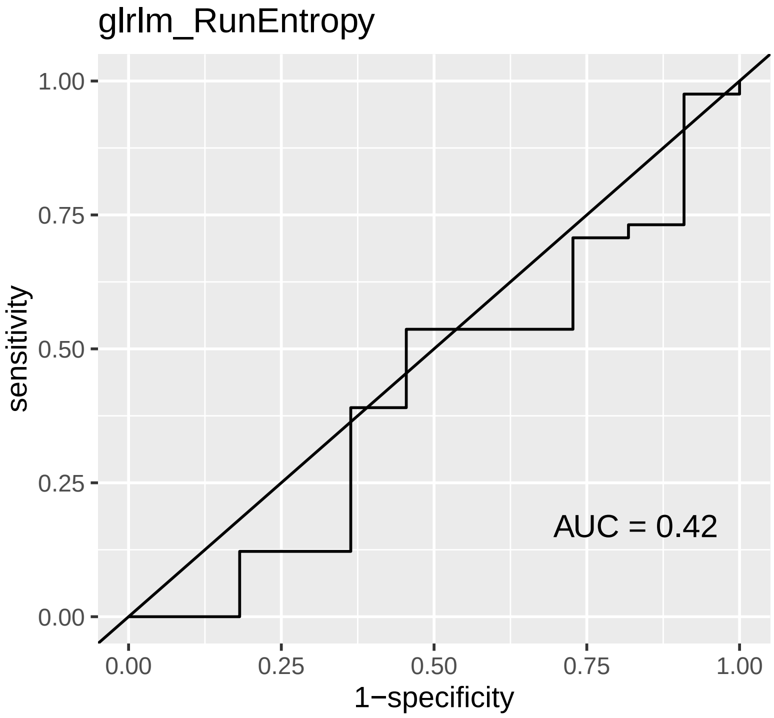
glrlm_RunEntropy
AUC = 0.42
sensitivity
1−specificity
0.00
0.25
0.50
0.75
1.00

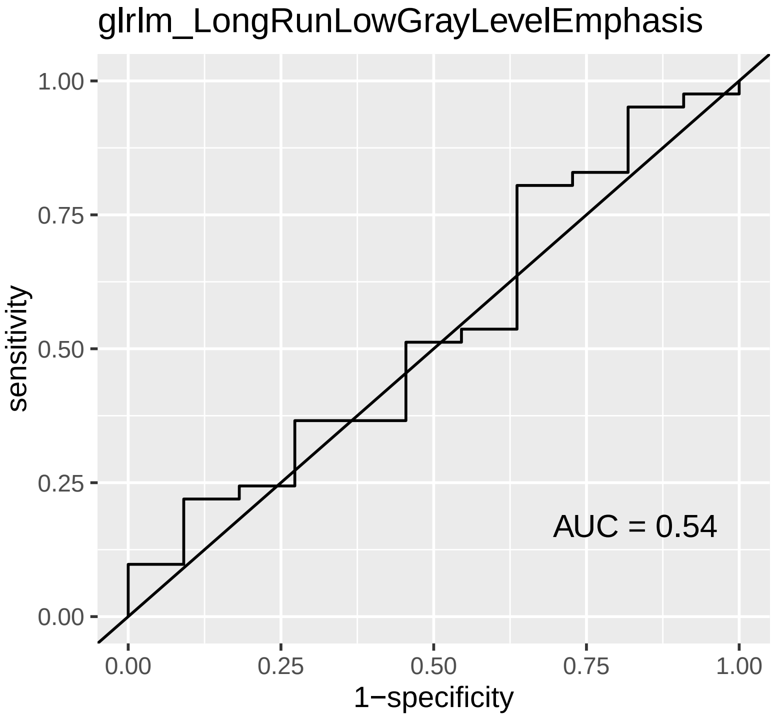
glrlm_LongRunLowGrayLevelEmphasis
AUC = 0.54
sensitivity
1−specificity
0.00
0.25
0.50
0.75
1.00

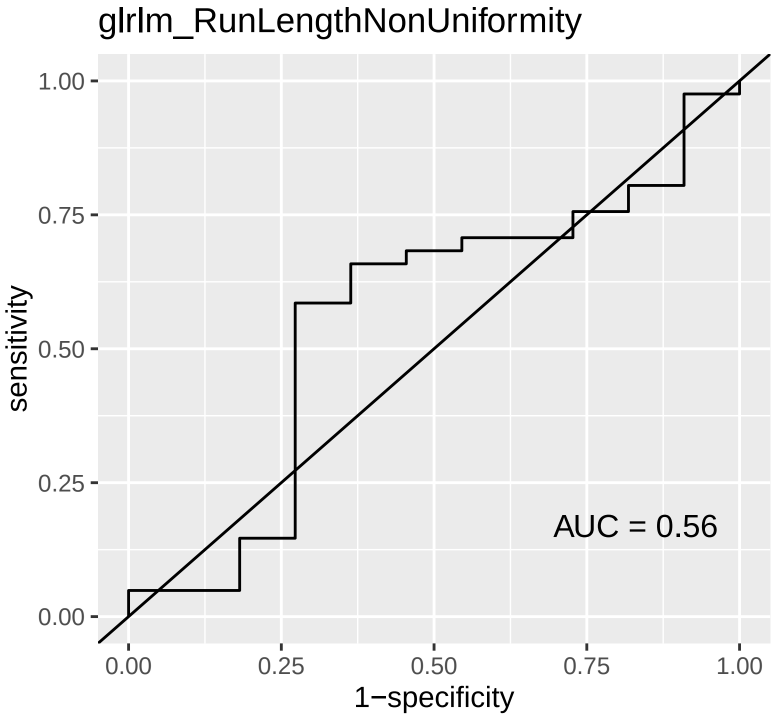
glrlm_RunLengthNonUniformity
AUC = 0.56
sensitivity
1−specificity
0.00
0.25
0.50
0.75
1.00

glrlm_RunLengthNonUniformityNormalized

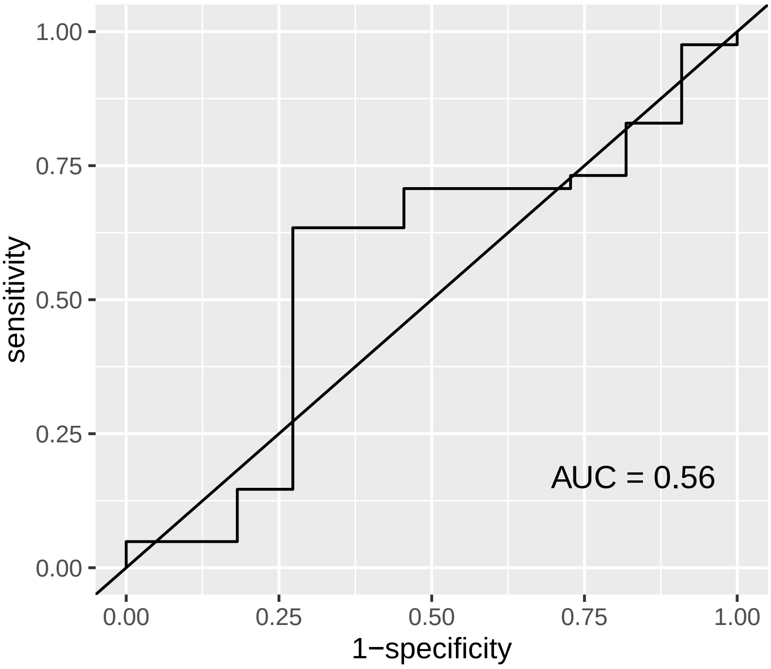

glrlm_ShortRunEmphasis

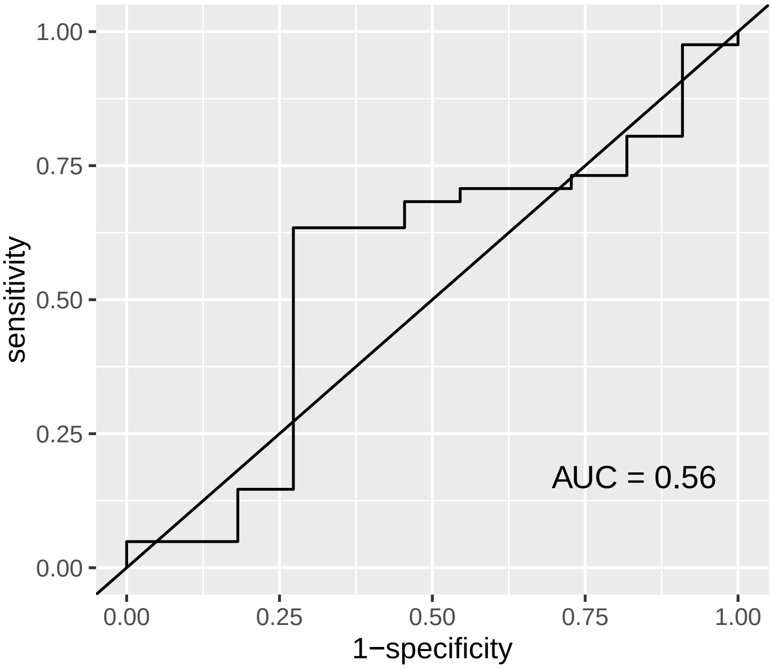

glrlm_RunPercentage

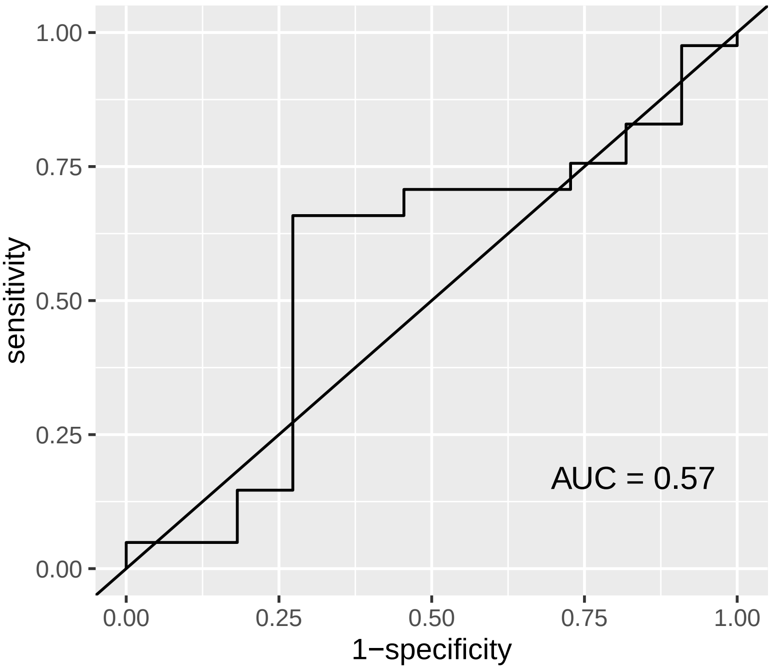

glrlm_ShortRunHighGrayLevelEmphasis

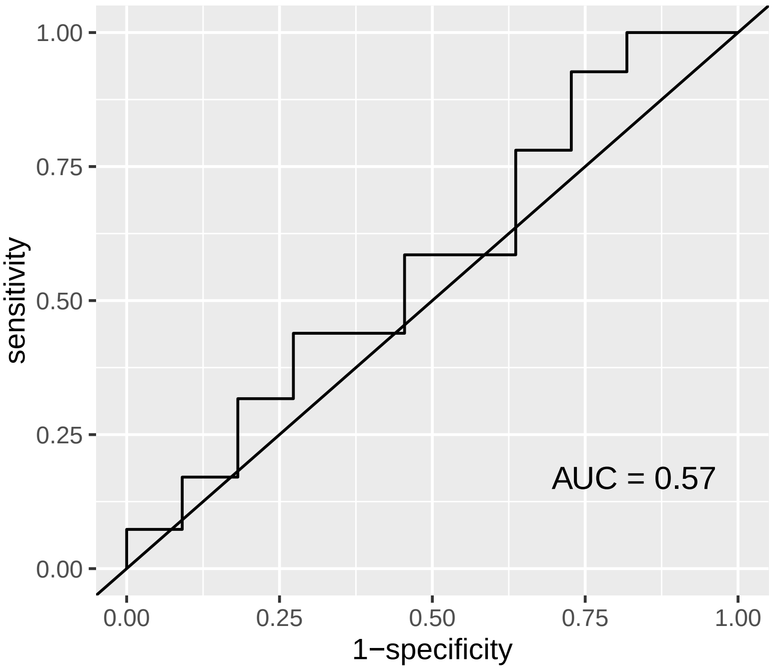

glrlm_RunVariance

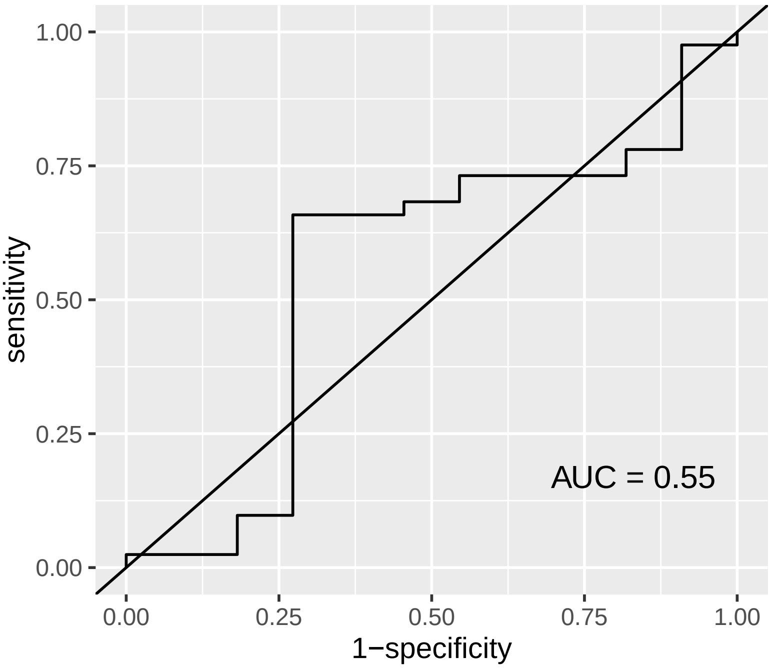

glrlm_ShortRunLowGrayLevelEmphasis

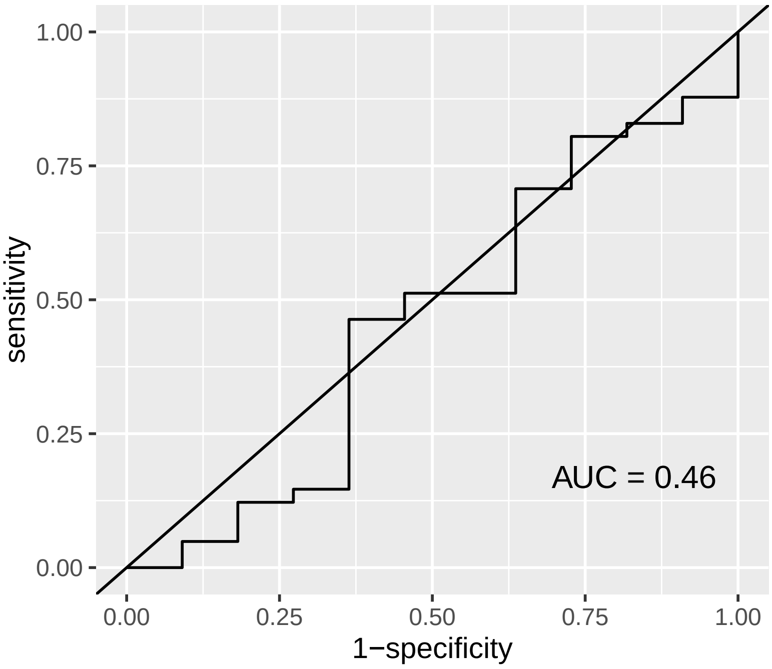

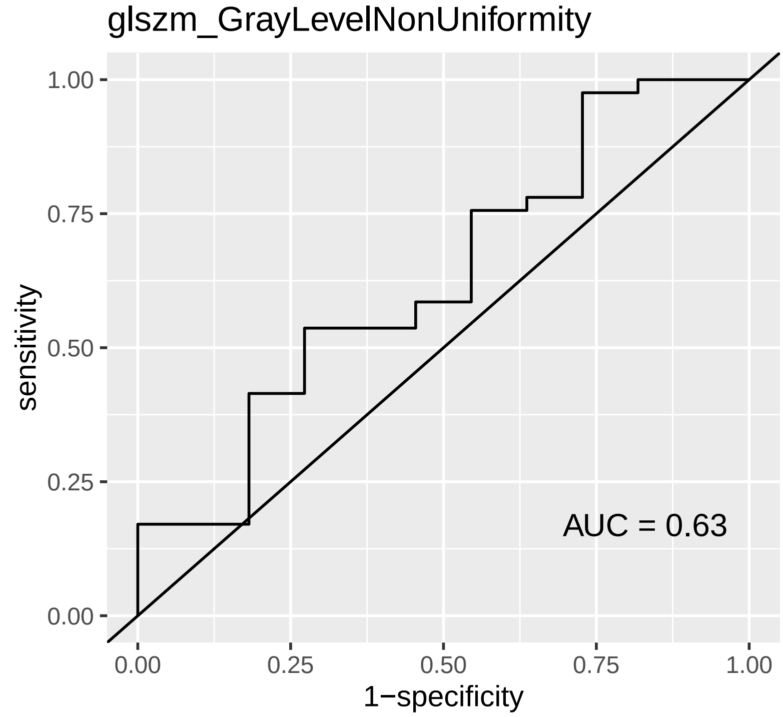
glszm_GrayLevelNonUniformity
sensitivity
1−specificity
0.00
0.25
0.50
0.75
1.00
AUC = 0.63

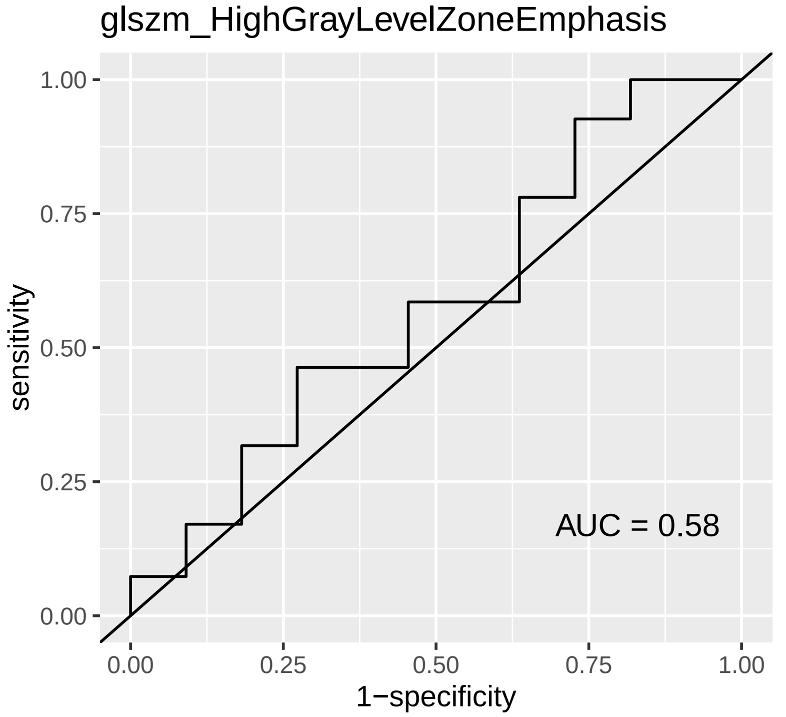
glszm_HighGrayLevelZoneEmphasis
sensitivity
1−specificity
0.00
0.25
0.50
0.75
1.00
AUC = 0.58

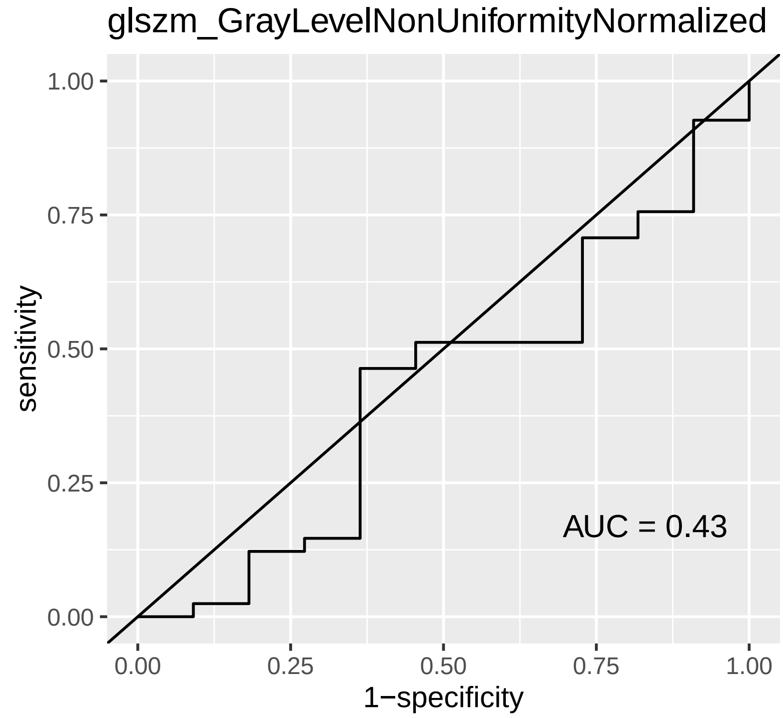
glszm_GrayLevelNonUniformityNormalized
sensitivity
1−specificity
0.00
0.25
0.50
0.75
1.00
AUC = 0.43

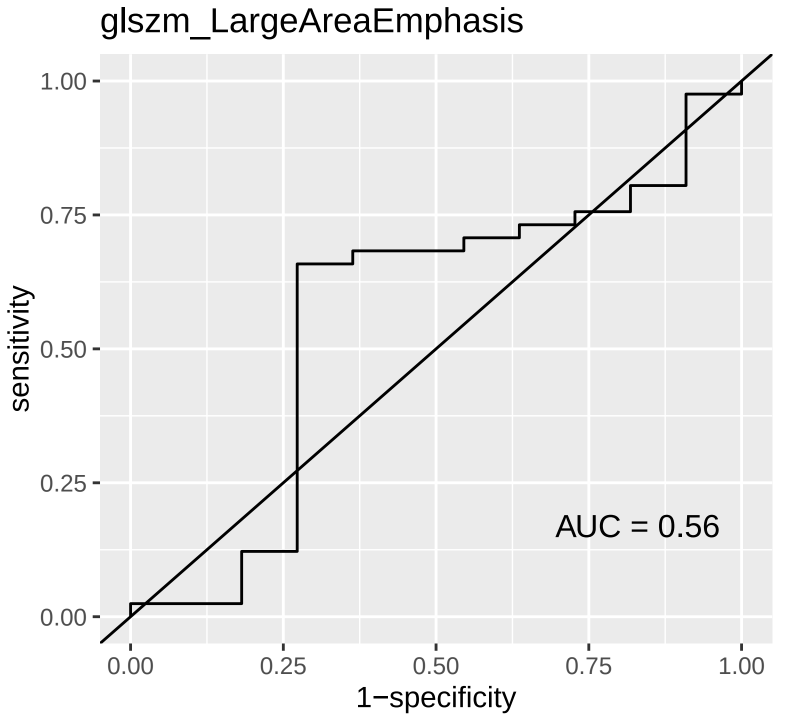
glszm_LargeAreaEmphasis
sensitivity
1−specificity
0.00
0.25
0.50
0.75
1.00
AUC = 0.56

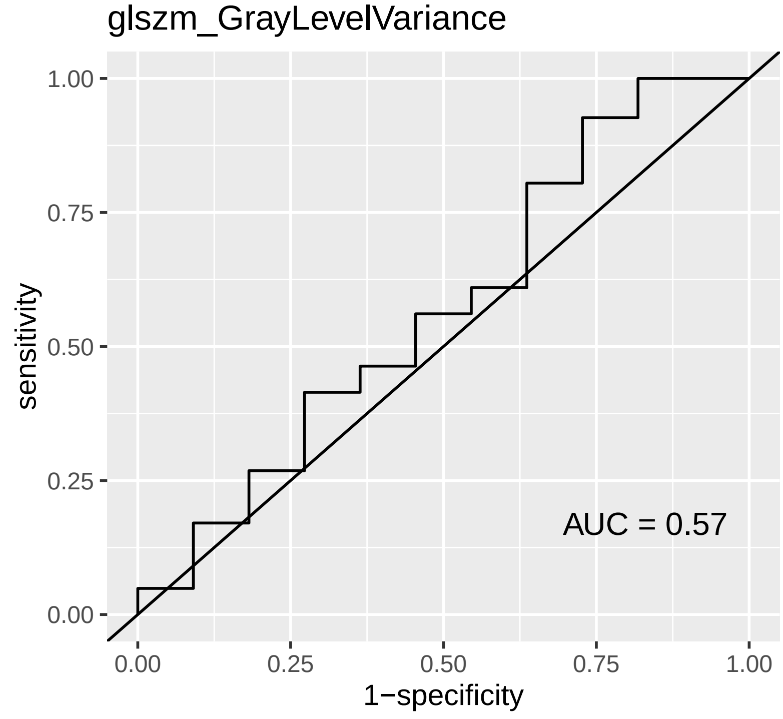
glszm_GrayLevelVariance
sensitivity
1−specificity
0.00
0.25
0.50
0.75
1.00
AUC = 0.57

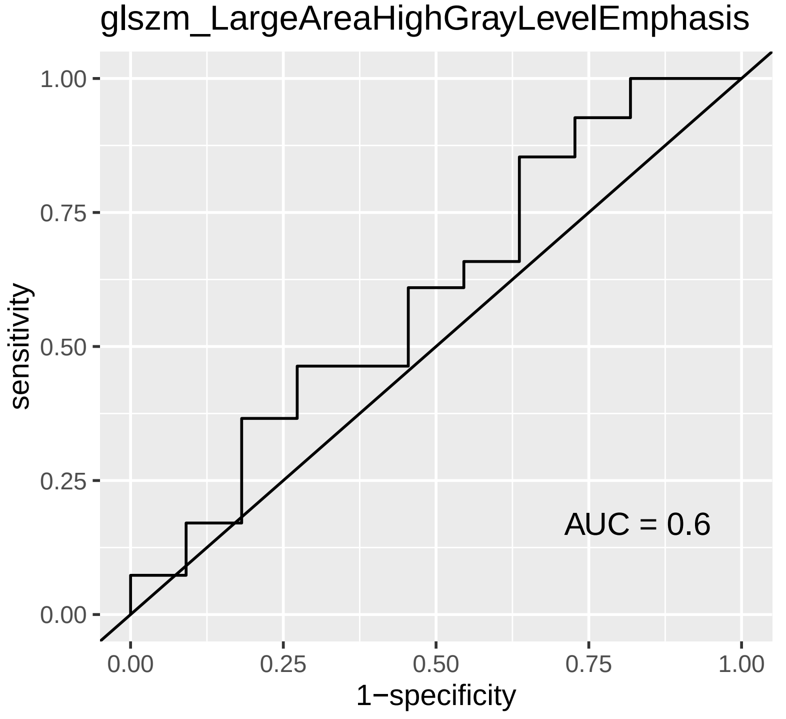
glszm_LargeAreaHighGrayLevelEmphasis
sensitivity
1−specificity
0.00
0.25
0.50
0.75
1.00
AUC = 0.6

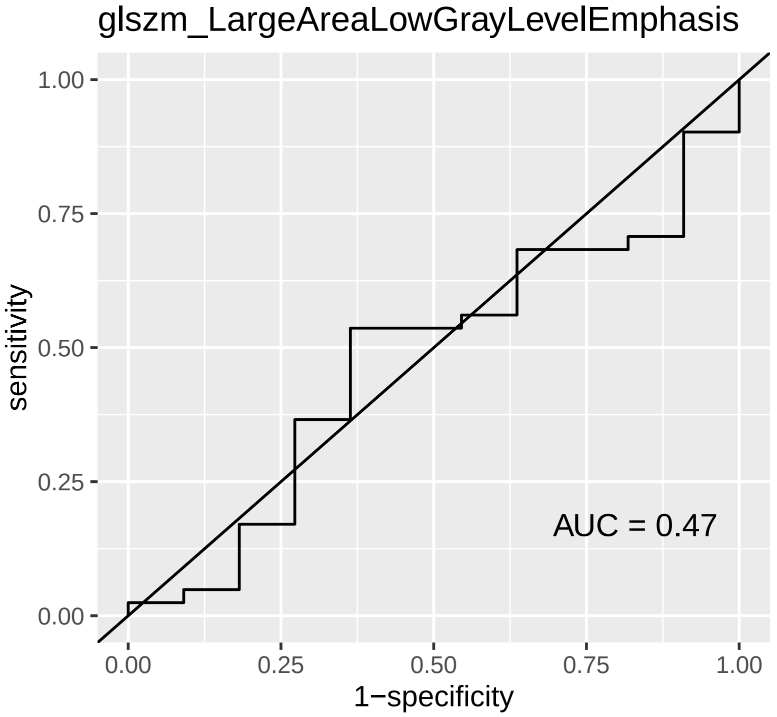
glszm_LargeAreaLowGrayLevelEmphasis
sensitivity
1−specificity
0.00
0.25
0.50
0.75
1.00
AUC = 0.47

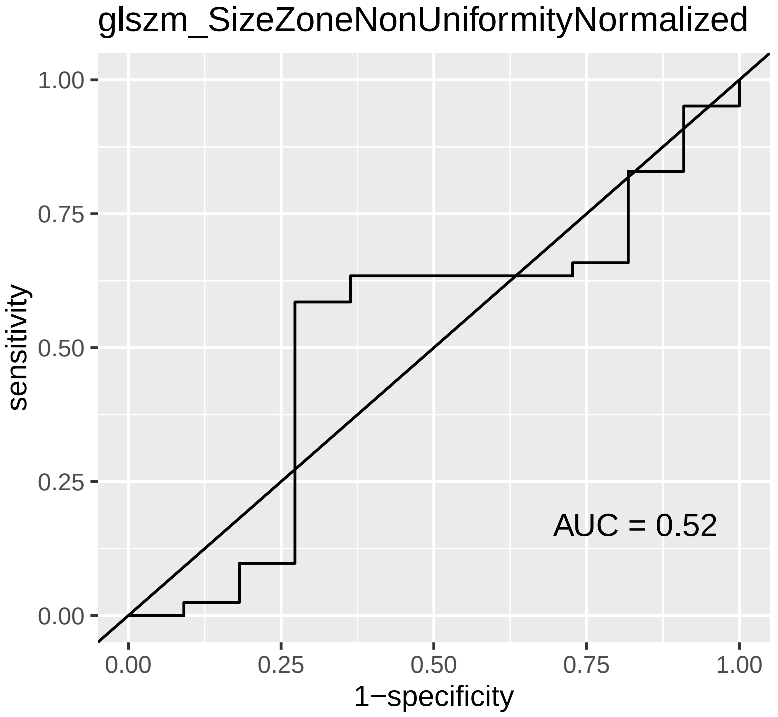
glszm_SizeZoneNonUniformityNormalized
sensitivity
1−specificity
0.00
0.25
0.50
0.75
1.00
AUC = 0.52

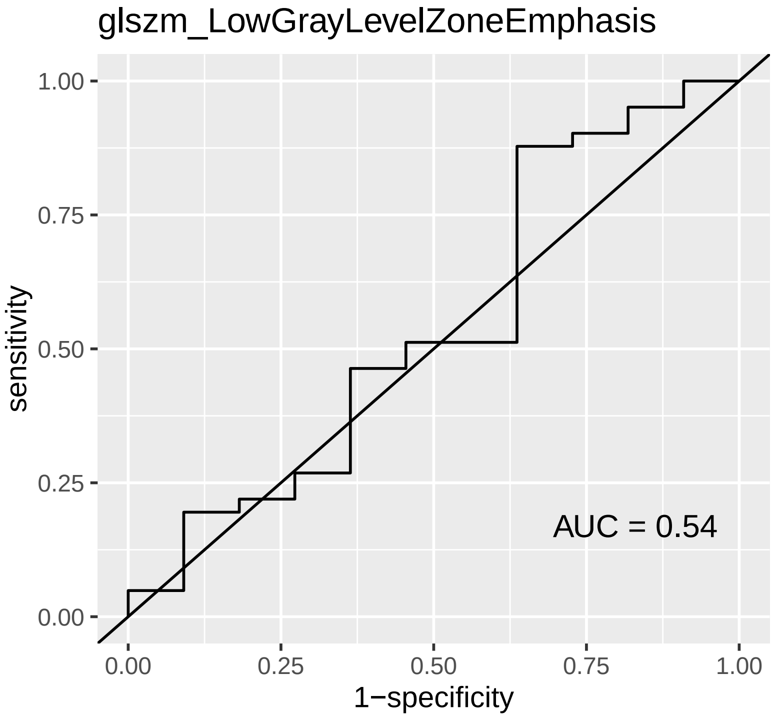
glszm_LowGrayLevelZoneEmphasis
sensitivity
1−specificity
0.00
0.25
0.50
0.75
1.00
AUC = 0.54

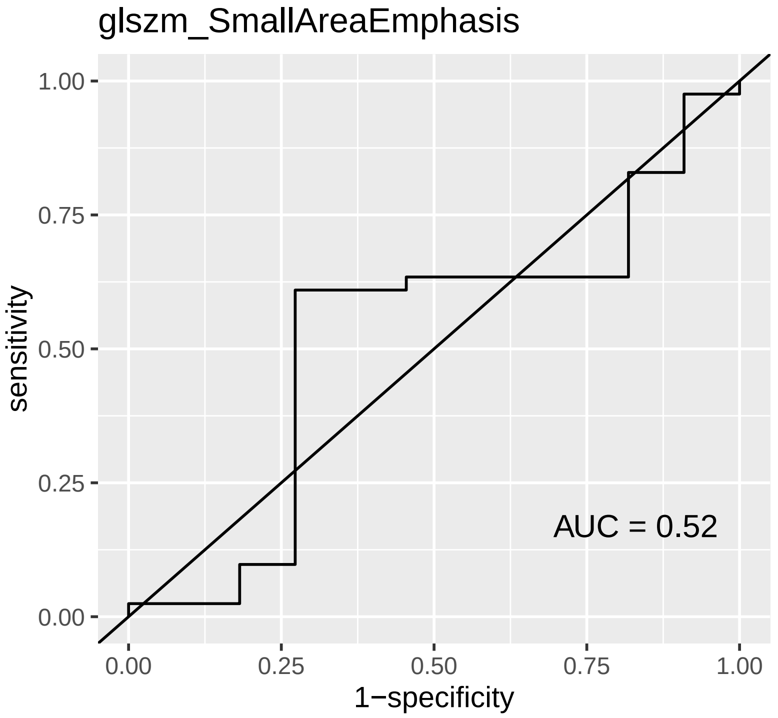
glszm_SmallAreaEmphasis
sensitivity
1−specificity
0.00
0.25
0.50
0.75
1.00
AUC = 0.52

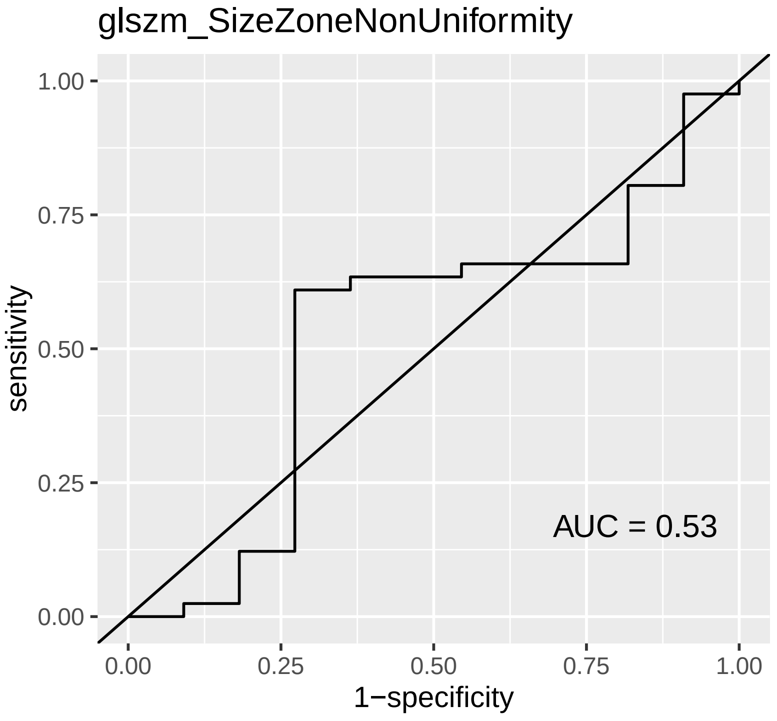
glszm_SizeZoneNonUniformity
sensitivity
1−specificity
0.00
0.25
0.50
0.75
1.00
AUC = 0.53

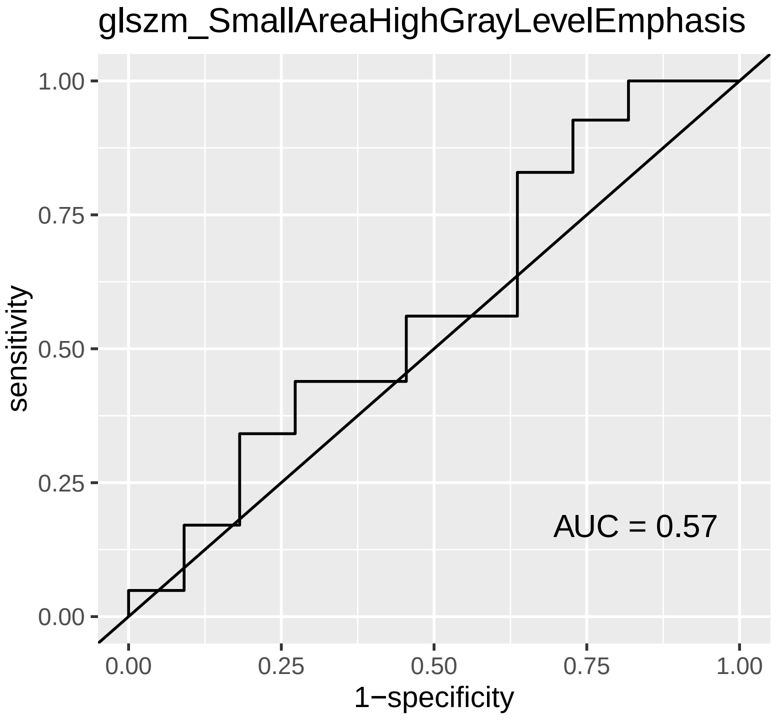
glszm_SmallAreaHighGrayLevelEmphasis
sensitivity
1−specificity
0.00
0.25
0.50
0.75
1.00
AUC = 0.57

glszm_SmallAreaLowGrayLevelEmphasis

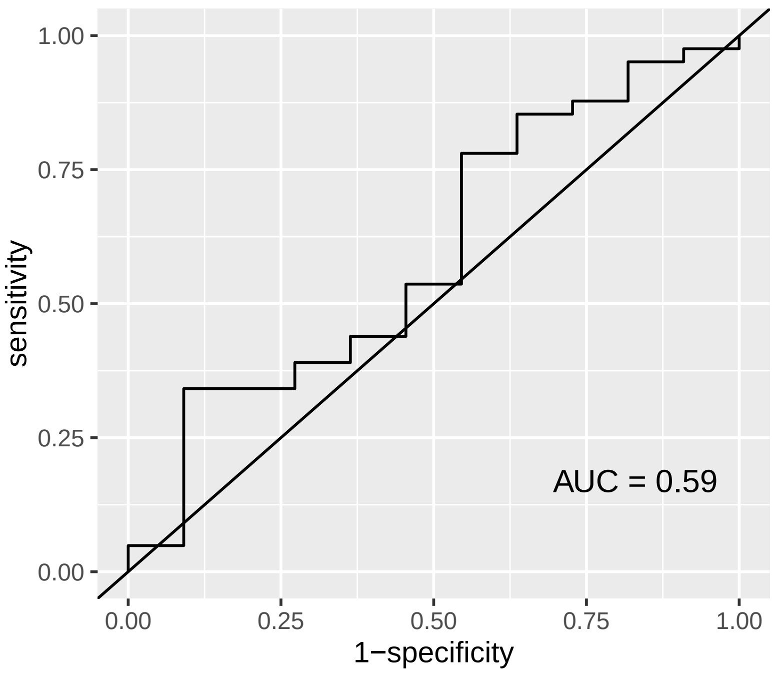

glszm_ZoneVariance

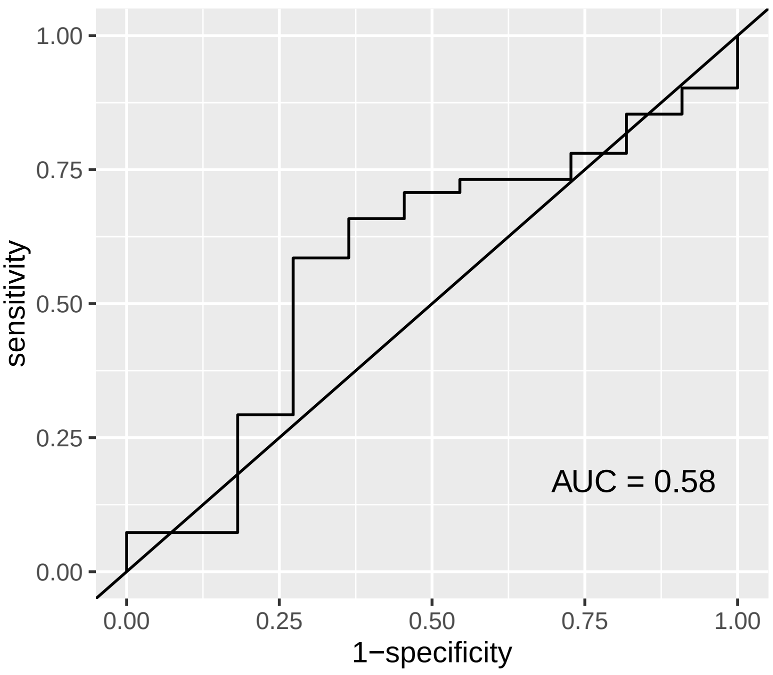

glszm_ZoneEntropy

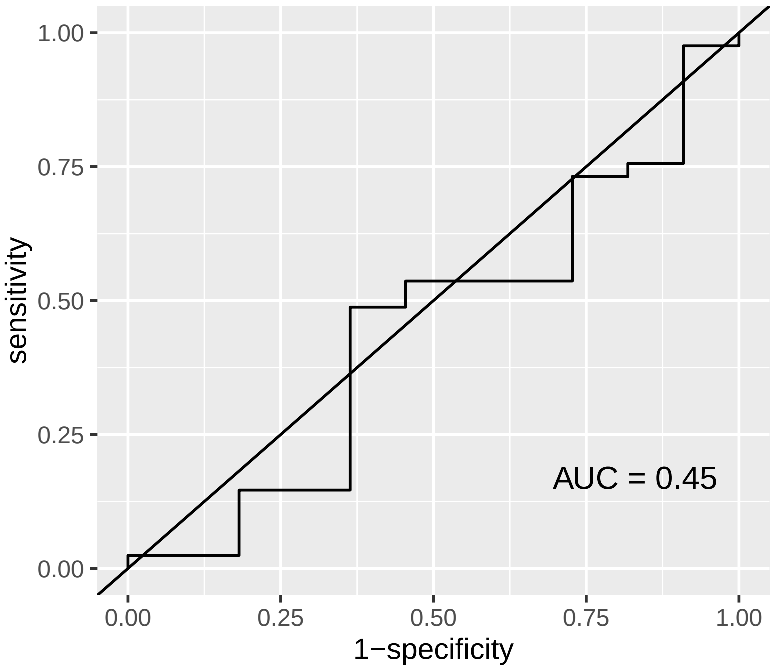

ngtdm_Busyness

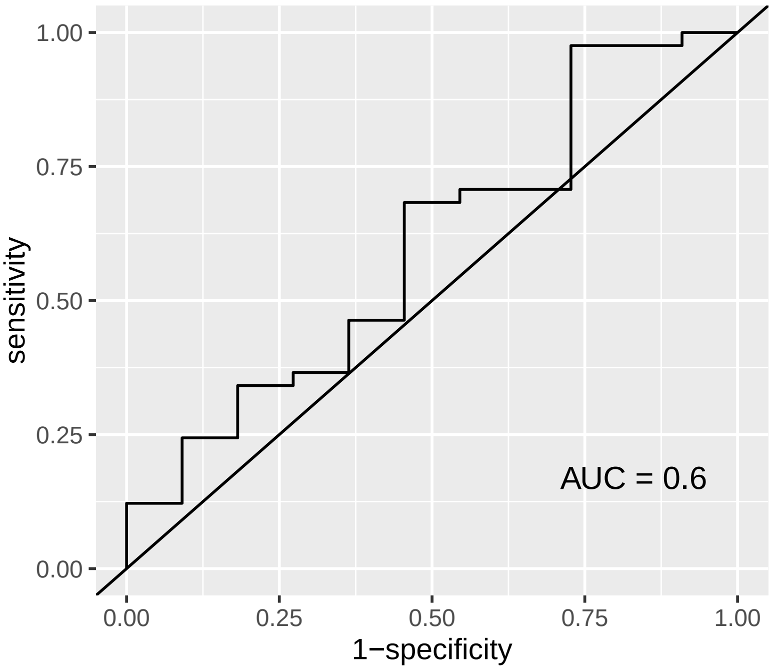

glszm_ZonePercentage

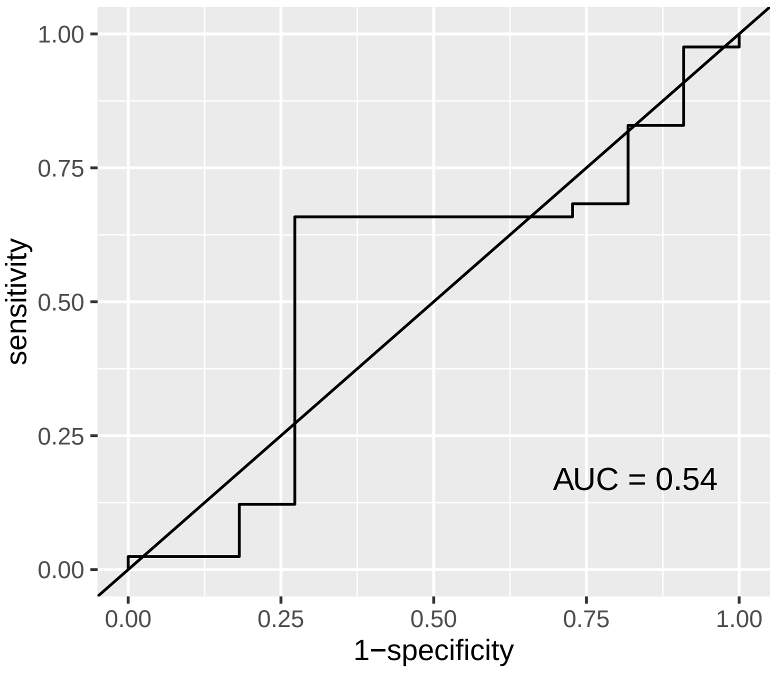

ngtdm_Coarseness

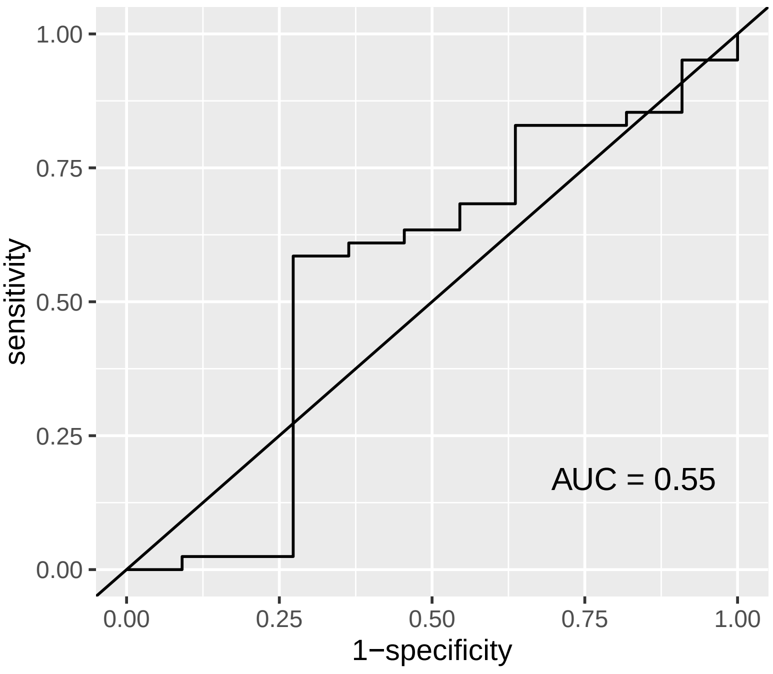

ngtdm_Complexity

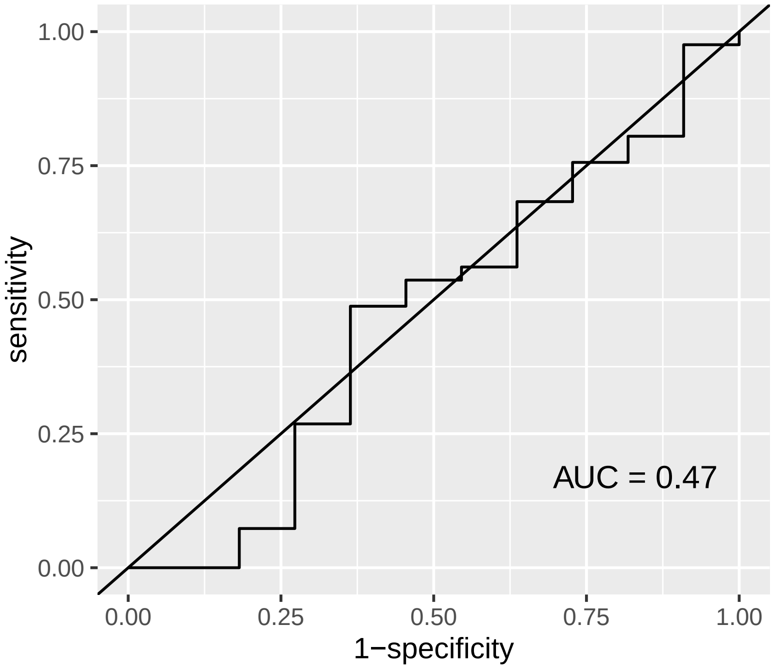

ngtdm_Contrast

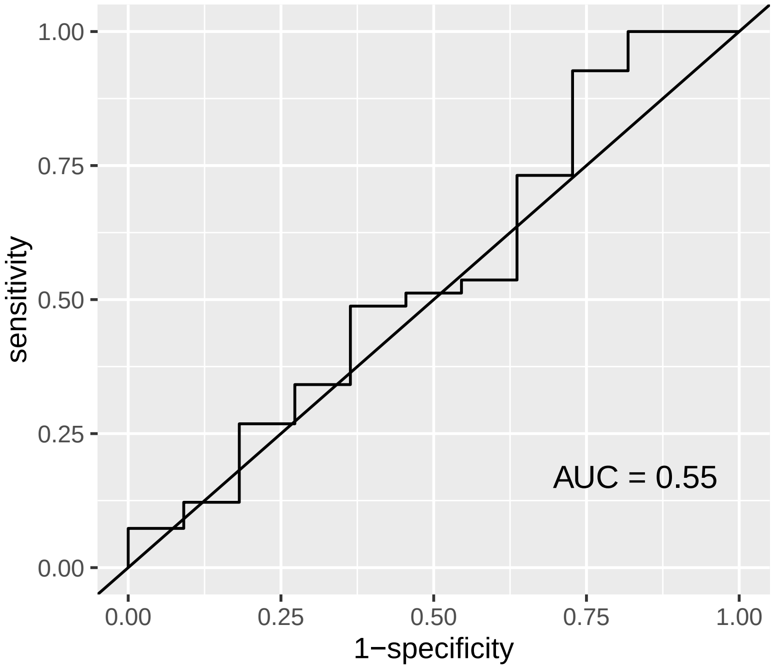

ngtdm_Strength

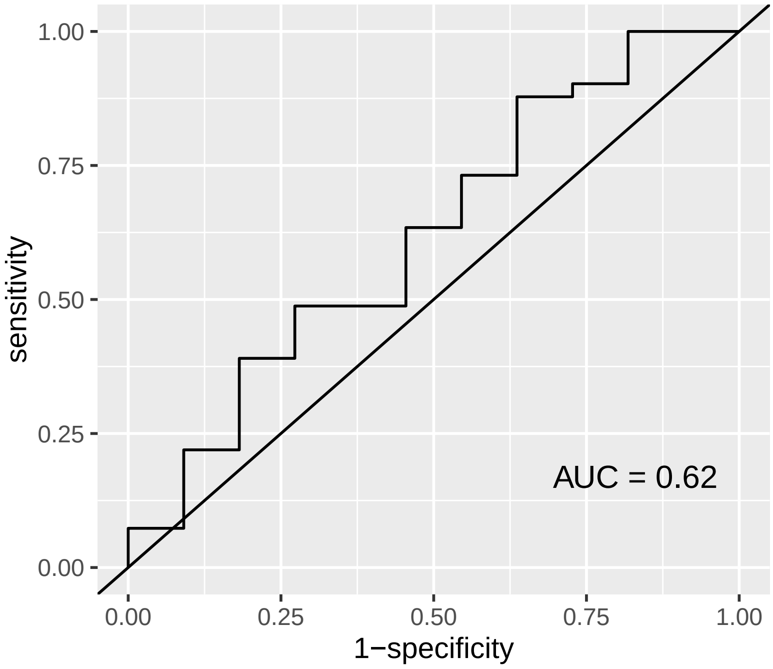

Supplement: Supplementary file 1 [file curroncol-31-00503-s001.zip › S9_ROCplots_feature_maps_all_zones.pdf]
